# An update of treatment modalities in children and adolescents with teeth affected by molar incisor hypomineralisation (MIH): a systematic review

**DOI:** 10.1007/s40368-021-00635-0

**Published:** 2021-06-10

**Authors:** C. Somani, G. D. Taylor, E. Garot, P. Rouas, N. A. Lygidakis, F. S. L. Wong

**Affiliations:** 1 Department of Paediatric Dentistry, Institute of Dentistry, Barts and The London School of Medicine & Dentistry, Queen Mary University of London, London, UK; 2 School of Dental Sciences, Faculty of Medical Sciences, Newcastle University, Newcastle upon Tyne, UK; 3 Univ. de Bordeaux, UFR Des Sciences Odontologiques, Bordeaux, France; 4 Private Paediatric Dental Clinic, 2 Papadiamantopoulou Street, 11528 Athens, Greece

**Keywords:** Molar incisor hypomineralisation, Developmental dental defect, Management, Children

## Abstract

**Purpose:**

To systematically review the treatment modalities for molar-incisor hypomineralisation for children under the age of 18 years. The research question was, ‘What are the treatment options for teeth in children affected by molar incisor hypomineralisation?’

**Methods:**

An electronic search of the following electronic databases was completed MEDLINE, EMBASE, Cochrane Central Register of Controlled Trials, LILACS, Google Scholar and Open Grey identifying studies from 1980 to 2020. The PRISMA guidelines were followed. The studies were screened, data extracted and calibration was completed by two independent reviewers.

**Results:**

Of 6220 potential articles, 34 studies were included. Twenty studies investigated management of molars with fissure sealants, glass ionomer cement, polyacid modified resin composite, composite resin, amalgam, preformed metal crowns, laboratory-manufactured crowns and extractions. In four articles management of incisors with microabrasion, resin-infiltration and a combination of approaches was reported. Eight studies looked at strategies to mineralise MIH-affected teeth and/or reduce hypersensitivity. Two studies investigated patient-centred outcomes following treatment. Due to the heterogeneity between the studies, meta-analysis was not performed.

**Conclusion:**

The use of resin-based fissure sealants, preformed metal crowns, direct composite resin restorations and laboratory-made restorations can be recommended for MIH-affected molars. There is insufficient evidence to support specific approaches for the management of affected incisors. Products containing CPP-ACP may be beneficial for MIH-affected teeth.

**Supplementary Information:**

The online version contains supplementary material available at 10.1007/s40368-021-00635-0.

## Introduction

Molar incisor hypomineralisation (MIH) is a well-recognised qualitative dental defect that involves demarcated enamel, hypomineralisation of one to four first permanent molars (FPM) and is frequently associated with similarly affected permanent incisors (Weerheijm et al. 2003). MIH is associated with hypersensitivity, difficulty gaining adequate anaesthesia, atypical carious lesions, post-eruptive breakdown (PEB), a reduction in resin bond strength, aesthetic concerns and a reduction in quality of life. It has been suggested that the presence of hypomineralised second primary molars (HSPM) is a predictive sign for MIH (Garot et al. 2018).

MIH has a reported global prevalence of 12.9% (11.7–14.3%) (Schwendicke et al. 2018; Zhao et al. 2018). The aetiology is multifactorial and is thought to be the result of systemic environmental factors that affect the developing enamel during the pre-, peri or early post-natal phases (up to three years) of life (Lygidakis et al. 2010). More recently, there have been suggestions that genetics and/or epigenetic changes are likely to be a main contributor to the development of MIH (Teixeira et al. 2017; Vieira and Manton 2019).

It has been reported that of MIH-affected teeth, 27.4% (23.5–31.7%) did or will require the need for treatment due to pain, sensitivity, or post-eruptive breakdown (Schwendicke et al. 2018). There are several available treatment options for both MIH-affected molars and incisors. Modalities range from prevention, restoration to extraction and possible post-extraction orthodontic treatment. However, deciding which approach is appropriate is complex. The main factors that need to be considered are patient cooperation, stage of dental development and defect severity; however, patient, and parental preferences, other anomalies and the psychosocial impact on the child must be taken into consideration. For MIH-affected molars with the potential for PEB or sensitivity, some type of early coverage must be undertaken to reduce sensitivity, prevent the development of adjunctive dental caries and minimise the risk of PEB, due to the increased porosity and decreased physical characteristics of the affected enamel. The rationale that underpins these philosophies allows the child to grow to the optimum age where a decision to definitively restore or extract these teeth can be made. Similarly, for MIH-affected incisors which require treatment, options should be minimally interventive to help reduce sensitivity, improve aesthetics whilst maintaining as much tooth tissue as possible.

Given the high treatment burden of treatment need for MIH-affected teeth and the range of options available, understanding the evidence-base for treatment options available is critical. More recently, there has been an increase in the number of studies investigating the management of MIH-affected teeth. Therefore, an update of previous systematic reviews (Lygidakis 2010; Elhennawy and Schwendicke 2016) is merited. The aim of this paper was to systematically review the success of treatment modalities for MIH-affected molars and incisors.

## Materials and methods

The systematic review protocol was registered with PROSPERO CRD42020196061. The PRISMA checklist was followed both in the planning and reporting of the review (Moher et al. 2009).

### Eligibility criteria

Studies were selected according to the defined criteria below:

#### Study design

Randomised controlled trials (RCTs), including cluster RCTs, controlled (non-randomised) trials (CCTs) with at least one data point before and after the intervention, case–control, cross-sectional, longitudinal/treatment prospective and retrospective studies will be considered. Case series, with a minimum of ten patients, were included based on consensus agreement from all authors. Studies on animal models, expert opinion and in vitro studies were excluded.

#### Participants

Studies examining human participants (age ≤ 18 years) who received treatment for MIH were included. Studies examining children and adults, or MIH and other diagnoses were included if data for participants ≤ 18 years with MIH was reported separately.

#### Interventions

Any interventions that managed MIH-affected teeth.

#### Comparators

Any other active intervention or treatment, pertaining to management of MIH-affected teeth, not similar to the intervention.

#### Outcomes

Due to the wide variety of treatment options and outcome measures used, the main outcome measure was success of the intervention. Success was defined based on the primary outcome measure used for each included study. Secondary outcome measures included longevity of the intervention, annual failure rate, quality of life, aesthetics, function, adverse events and patient, parent and dentist satisfaction with respect to the outcome.

#### Report characteristics

No restrictions on setting or geographical location were applied. Manuscripts in all languages were included and translations appropriately sought.

### Search strategy

The search strategy was developed by the project team, then peer-reviewed by a specialist librarian, using the Peer-Reviewed of Electronic Search Strategies (PRESS) standard (McGowan et al. 2016). The search strategy included the following terms:

- #1 (molar AND incisor AND hypominerali*ation) OR (demarcated AND opacities) OR (MIH) OR (mottled AND enamel) OR (developmental AND opacit*) OR (idiopathic OR nonfluoride) AND opacit*) OR (white AND opaque AND enamel) OR (Non-endemic AND mottling AND enamel) OR (hypominerali* AND t**th) OR (enamel AND opacit*) OR (enamel AND defect) OR (enamel AND (hypominerali*) OR (developmental AND dental AND defects) OR (calcification AND molar) OR (cheese AND molar) OR (developmental AND hypominerali*) OR (idiopathic AND hypominerali*) OR (enamel dysminerali*ation)
- *Medline:* Exp DENTAL ENAMEL HYPOPLASIA *EMBASE:* Exp ENAMEL HYPOPLASIA
- #2 (manage*) OR (treat*) OR (restor*) OR (extract*) OR (bleach*) OR (resin) OR (composite) OR (orthodont*) OR (seal*) OR (microabrasion) OR (crown) OR (veneer) OR (prevent*) OR (fluorid*) OR (SDF) OR (CPP-ACP) OR (casein phosphopeptide-amorphous calcium phosphate) OR (onlay) OR (inlay) OR (root canal) OR (pulp therapy) OR (pulpotomy) OR (pulpectomy) OR (endodontic) OR (infiltration) OR (reminerali*ation)
- *Medline:* Exp OPERATIVE DENTISTRY, TOOTH REMINERALIZATION, TOOTH PREPARATION, ORAL SURGERY, PREVENTIVE DENTISTRY, ORALSURGICAL PROCEDURES, ORTHODONTICS, DENTAL ESTHETICS, ENDODONTICS, DENTAL POLISHING, DENTAL BONDING, DENTAL ATRAUMATIC RESTORATIVE TREATMENT, DENTAL ANAESTHESIA *EMBASE:* Exp OPERATIVE DENTISTRY, PREVENTIVE DENTISTRY, ENDODONTICS, ORALSURGICAL PROCEDURES, RESTORATIVE DENTISTRY, ORTHODONTICS, DENTAL ANAESTHESIA, DENTAL BONDING, DENTAL POLISHING, ATRAUMATIC RESTORATIVE TREATMENT
- #1 & #2

The initial searches were completed through the electronic databases MEDLINE, EMBASE, Cochrane Central Register of Controlled Trials, LILACS, Google Scholar and Open Grey up until 26th August 2020. To ensure literature saturation, the electronic search was complemented by a search through the reference lists of included studies. Previous narrative and systematic reviews on the management of MIH-affected teeth were searched in order to identify any further suitable studies. Searches were limited to studies published from 1st January 1980 to 26th August 2020.

### Study selection

Search results were organised using Zotero™. Duplicate articles were removed. Title and abstract screening, against the inclusion and exclusion criteria, was carried out independently by two reviewers (CS & GT), with any disagreement resolved by consensus. If necessary, any unresolved differences were resolved by a consensus agreement by all of the authors.

Full texts were obtained for all titles that met these criteria. Two reviewers (CS & GT) assessed the full texts against the inclusion/exclusion criteria independently, with any disagreement resolved by consensus. If necessary, any unresolved differences were resolved by a consensus agreement by all of the authors. Reasons for exclusion were recorded.

A calibration exercise (using ten studies) was conducted with two reviewers (CS & GT) undertaking data extraction and risk of bias assessment. Cohen’s kappa (κ) was calculated as 0.75 for overall inter-rater agreement aafter which, data extraction and risk of bias assessment was carried out by one reviewer (CS). Cohen’s kappa (κ) was calculated as 0.84 for overall intra-rater agreement by randomly re-assessing 10% (3 studies) of included studies four weeks after initial data extraction.

### Data extraction and quality assessment

A standardised data extraction form was used to record the following details:

- Study characteristics (author, publication year, title, publication journal, study design, country, setting, funding)
- Study design
- Number of patients included
- Number of teeth studied
- Patient demographics (age, gender, ethnicity, socio-economic status)
- Patient selection (inclusion, exclusion criteria, controls included, presence of any other pathology such as dental caries)
- Primary and secondary outcome measures
- Severity of MIH (where this was not explicitly stated, an estimation was made using the EAPD criteria)
- Number and experience of clinicians providing treatment
- Intervention provided, including details on use of behaviour management techniques, local or general anaesthesia, use of sedation, technique and materials used
- Success of intervention (as per primary outcome measure used in that study)
- Annual failure rate and longevity (if applicable)
- Adverse events
- Dentist, patient and parent-reported outcomes

Any missing information was noted as not reported.

Risk of bias assessment was completed for each study using the ROBINS-I tool in non-randomised studies (Sterne et al. 2016) and RoB 2 tool for randomised trials (Higgins and Thomas 2020).

### Data synthesis

Narrative synthesis was used to explore the findings from the included studies due to the expected heterogeneity between studies.

## Results

**Fig. 1:**
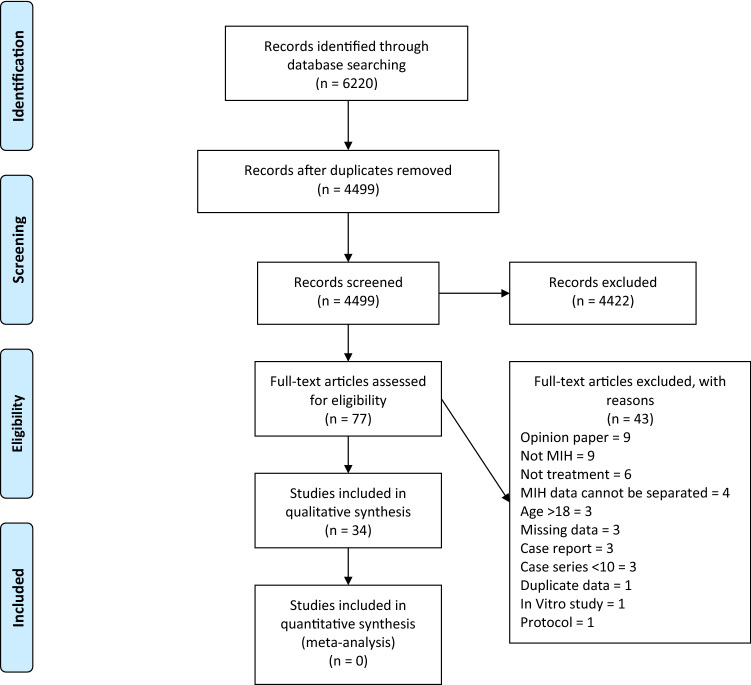
PRISMA flow diagram

A total of 6220 articles were identified from searching of electronic databases. After removing duplicates, 4499 were identified for title and abstract screening. Seventy-seven articles underwent full text review, of which 34 met the inclusion criteria. A summary of article selection is presented as a flowchart, based on PRISMA guidelines (Fig. 1).

### Findings for molars

Of the 34 included studies, 20 studies were on the management of MIH-affected molars and involved the management of 1711 teeth. There were ten broad categories of management strategies employed with significant variability in the techniques and materials used. Overall success, as defined by the primary outcome measure, and study-specific details are outlined in Table 1. Three studies investigated the use of resin-based fissure sealants (Kotsanos et al. 2005a, b; Lygidakis et al. 2009; Fragelli et al. 2017) in MIH-affected molars with only one study looking at the impact of resin adhesives (Lygidakis et al. 2009). Five studies used glass ionomer cement (GIC) as a restorative material (Mejare et al. 2005; Fragelli et al. 2015; Grossi et al. 2018; Linner et al. 2020; Durmus et al. 2020), although a significant variation in the success was noted across the studies. One study investigated polyacid modified resin composite restorations (Mejare et al. 2005) and two investigated amalgam (Kotsanos et al. 2005a, b; Mejare et al. 2005). Composite resin restorations were examined in eight studies (Lygidakis et al. 2003; N. Kotsanos et al. 2005a, b; Mejare et al. 2005; Sonmez and Saat 2017; de Souza et al. 2017; Gatón-Hernandéz et al. 2020; Linner et al. 2020; Rolim et al. 2020). Most studies adopted a traditional invasive approach where all hypomineralised enamel was removed and hardness determined (Lygidakis et al. 2003; Kotsanos et al. 2005a, b; Mejare et al. 2005; de Souza et al. 2017; Gatón-Hernandéz et al. 2020), but the remaining took less invasive approaches (Sonmez and Saat 2017; Linner et al. 2020; Rolim et al. 2020). Two of the composite resin restoration studies addressed the impact of bonding technique and found no difference between a total-etch adhesive approach and self-etch adhesive approach (de Souza et al. 2017; Rolim et al. 2020). Preformed metal crowns (PMC) were used in three studies (Kotsanos et al. 2005a, b; Koleventi et al. 2018; Oh et al. 2020) with consistent results noted. Four studies looked at laboratory-manufactured crowns (Gaardmand et al. 2013; Dhareula et al. 2018, 2019; Linner et al. 2020) with approaches such as cast adhesive gold copings (Gaardmand et al. 2013), indirect composite resin onlays (Dhareula et al. 2018) and ceramic restorations (Linner et al. 2020) tested. Only one study compared two techniques (Dhareula et al. 2019). Extraction of MIH-affected molars was investigated in three studies (Mejare et al. 2005; Jalevik and Moller 2007; Oliver et al. 2014).

**Table 1:** Included studies on treatment of molars

Study *Country*	Study design	Severity of MIH	Follow-up in months (range)	Age of participants in years	No. of participants (drop outs)	No. of teeth (drop outs)	Primary outcome measure	Intervention	Success
**Fissure sealants**									
Kotsanos et al. (2005a, b) *Greece*	Restrospective case–control	Mild & Severe	Mean 54	Mean 7.7	NR	35 restorations	Number of re-treatments needed	Fissure sealant (FS)	77.1% did not need retreatment
Lygidakis et al. (2009) *Greece*	Randomised trial	Mild	48	Mean 6.8 SD ± 0.4 Range 6–7	54 (7)	108	Success of fissure sealant	G1: resin-based FS applied with adhesive G2: resin-based FS applied without adhesive	G1: 70.2% fully sealed, 29.7% partially sealed and 0% lost G2: 25.5% fully sealed, 44.6% partially sealed and 29.7% lost
Fragelli et al. (2017) *Brazil*	Prospective cohort	Mild	18	Mean 7 Range 6–8	21 (0)	41	Success of restoration using USPHS-modified criteria	Resin-based FS G1: teeth affected by MIH G2: teeth unaffected by MIH	G1: 72.0% G2: 62.6% No difference between groups
**Glass ionomer cement (GIC) restorations**									
Mejare et al. (2005) *Sweden*	Restrospective cohort	Mild & Severe	NR	At referral: Mean 8.5 SD ± 2.16 Range 6–17 At follow-up: Mean 18.2	NR	63 restorations	Success of restoration	GIC restoration	49.2% acceptable
Fragelli et al. (2015) *Brazil*	Prospective cohort	Severe	12	Mean 7.7 Range 6.37–9.54	21 (0)	48	Success of restoration using USPHS-modified criteria	Non-invasive GIC restoration	78% cumulative survival
Grossi et al. (2018) *Brazil*	Prospective cohort	Severe	12	Mean 10.55 SD ± 1.25 Range 7–13	44 (1 incisor)	60 (6 restorations)	Success of restorations measured using modified ART criterion	Glass hybrid restoration using ART technique	98% cumulative survival
Durmus et al. (2020) *Turkey*	Prospective cohort	Severe	24	Mean 8.94 SD ± 1.41	58 (0)	134	Success of restoration using USPHS-modified criteria	Invasive high-viscocity GIC restoration	87.5% cumulative survival
Linner et al. (2020) *Germany*	Retrospective cohort	Severe	Mean 42.9	Mean 11.2 SD ± 2.9 Range 6.6–18.2	NR	28	Success of restoration using FDI criteria	Non-invasive GIC restoration	7.0% cumulative survival at 36 months
**Polyacid modified resin composite restorations**									
Mejare et al. (2005) *Sweden*	Retrospective cohort	Mild & Severe	NR	At referral: Mean 8.5 SD ± 2.16 Range 6–17 At follow-up: Mean 18.2	NR	14 restorations	Success of restoration	Polyacid modified resin composite restoration	64.3% acceptable
**Composite Resin Restorations**									
Lygidakis et al. (2003) *Greece*		Severe	48	Mean 8.84 SD ± 0.75 Range 8–10	46	52 (3 restorations)	Survival of restoration, hypersensitivity score using Cvar Ryge criteria	Composite resin restoration	100% survival and 100% non-sensitive
Kotsanos et al. (2005a, b) *Greece*	Retrospective case–control	Mild & Severe	Mean 54	Mean 7.7	NR	59 restorations	Number of re-treatments needed	Composite resin restoration	74.6% did not need retreatment Overall retreatment higher than control OREST = 3.10
Mejare et al. (2005) *Sweden*	Retrospective cohort	Mild & Severe	NR	At referral: Mean 8.5 SD ± 2.16 Range 6–17 At follow-up: Mean 18.2	NR	34 restorations	Success of restoration	Composite resin restoration	85.3% acceptable
de Souza et al. (2017) *Brazil*	Randomised trial	Severe	18	Mean 7 Range 6–8	18 (0)	41	Success of restoration using USPHS-modified criteria	Selective-etch adhesive (SEA) or total etch adhesive (TEA) composite resin restoration	SEA 68%, TEA 54% cumulative survival
Sonmez and Saat (2017) *Turkey*	Randomised trial	Severe	24	Mean 8.8 Range 8–12	30 (0)	95	Success of restoration using USPHS-modified criteria	Composite resin restoration G1: Invasive cavity preparation G2: Non-invasive cavity preparation G3: Non-invasive cavity preparation + pretreatment with 5% sodium hypochlorite G4: control, unaffected by MIH	Retention rate: G1: 93.7% G2: 80.7% G3: 93.5% G4: 100% No difference in success rates between G1, G3, and G4. Success rate group 2 significantly lower than other 3 groups
Gatón-Hernandéz et al. (2020) *Spain*	Prospective cohort	Severe	24	Mean 7.33 Range 6–8	326 (45)	326	Success of restoration, evidence of radiographic apexogenesis, absence of pulpal pathology	Selective caries removal and placement of GIC restoration. Replacement wtith composite resin restoration at 6 months	96.8% clinical and radiographic success
Linner et al. (2020) *Germany*	Retrospective cohort	Severe	Mean 42.9	Mean 11.2 SD ± 2.9 Range 6.6–18. 2	NR	126 27	Success of restoration using FDI criteria	Non-invasive composite resin restoration Conventional composite resin restoration	29.9% cumulative survival at 36 months 76.2% cumulative survival at 36 months
Rolim et al. (2020) *Brazil*	Randomised trial	Severe	12	Mean 10 Range 7–16	35	64 (14 teeth)	Success of restoration using USPHS-modified criteria	Bulk-fill composite resin restoration GI: TEA G2: SEA	G1: 80.8%, G2: 62.3% cumulative survival, no difference between groups
**Amalgam restorations**									
Kotsanos et al. (2005a, b) *Greece*	Retrospective case–control	Mild & Severe	Mean 54	Mean 7.7	NR	18 restorations	Number of re-treatments needed	Amalgam restoration	38.9% did not need retreatment Overall retreatment higher than control OREST = 3.10
Mejare et al. (2005) *Sweden*	Retrospective cohort	Mild & Severe	NR	At referral: Mean 8.5 SD ± 2.16 Range 6–17 At follow-up: Mean 18.2	NR	32 restorations	Success of restoration	Amalgam restoration	78.1% acceptable
**Preformed Metal Crowns (PMC)**									
Kotsanos et al. (2005a, b) *Greece*	Retrospective case–control	Mild & Severe	Mean 54	Mean 7.7	NR	24 restorations	Number of re-treatments needed	Placement of PMC	100% did not need retreatment Overall retreatment higher than control OREST = 3.10
Koleventi et al. (2018) *Greece*	Prospective cohort	Severe	6	Mean 10.6 SD ± 4.2	14 (0)	14	Multiple periodontal and microbiological outcome measures	Placement of PMC	100% survival. Increase in gingival index, periodontal depth, *P. Gingivalis* and *T. Forsythia* counts when compared with untreated teeth
Oh et al. (2020) *South Korea*	Retrospective cohort	Severe	44.3 mean (12–118)	Mean 9.27 Range 6–14 *mixed data	NR	50	Success of restoration	Placement of PMC	86% survival
**Laboratory manufactured restorations**									
Gaardmand et al. (2013) *Denmark*	Prospective cohort	Severe	38.5 mean	Mean 12 Range 8–18	33	57 (4 restorations)	Success of restoration	Cast adhesive gold coping	98.2% functioning at mean 38.6 months
Dhareula et al. (2018) *India*	Case series	Severe	34.8 mean, (30–36)	Mean 11.4 Range 8–14	10	10	Success of restoration using USPHS criteria	Indirect composite resin onlay	100% survival
Dhareula et al. (2019) *India*	Randomised trial	Severe	36	Mean 10.2 Range 8–13	30	42 (5 restorations)	Success of restoration, radiographics outcomes, Shiff's sensitivity status, gingival health (Loe and Sillness GI)	G1: minimally invasive cast metal onlay G2: indirect resin onlay	G1: 85% G2: 100% No difference between groups
Linner et al. (2020) *Germany*	Retrospective cohort	Severe	Mean 42.9	Mean 11.2 SD ± 2.9 Range 6.6–18.2	NR	23	Success of restorations using FDI criteria	CAD-CAM fabricated ceramic restoration	100% cumulative survival at 36 months
**Extractions**									
Mejare et al. (2005) *Sweden*	Retrospective cohort	Mild & Severe	NR	At referral: Mean 8.5 SD ± 2.16 Range 6–17 At follow-up: Mean 18.2	NR	76	Space closure	Extraction of FPM (between 1–4)	87% acceptable space closure
Jalevik and Moller (2007) *Sweden*	Prospective cohort	Severe	Median 68.4 (45.6–99.6)	Median 8.2 Range 5.6–12.7	33 (6)	77	Need for further orthodontic treatment	Extraction of FPM (between 1–4)	45% favourable development of dentition without need for orthodontic intervention
Oliver et al. (2014) *Spain*	Retrospective case series	Severe	Mean 44.4 (10–120 months) *mixed data	Mean 10.1	18	36	Completed space closure	Extraction of FPM (between 1–4)	61.2% complete space closure

KEY: *SD* standard deviation, *NR* not reported, G-group, *FS* fissure sealant, *USPHS* United States Public Health Service, *ART* atraumatic restorative treatment, *SEA* self-etch adhesive, *TEA* total-etch adhesive, *CAD-CAM* computer aided design and computer aided manufacture, *GI* gingival index, *DPT* dental panoramic tomograph

### Findings for incisors

Four of the included studies, including 105 incisors, focussed on management of MIH-affected incisors. Similar to the results of MIH-affected molars, the overall success, as defined by the primary outcome measure and study specific details, are outlined in Table 2. Three studies investigated resin infiltration (Kim et al. 2011; Elbaz and Mahfouz 2017; Bhandari et al. 2018) and one study investigated two different microabrasion approaches (Bhandari et al. 2019).

**Table 2:** Included studies on treatment of incisors

Study *Country*	Study Design	Severity of MIH	Follow-up in months (range)	Age of Participants	No. of participants (drop outs)	No. of teeth	Primary outcome measure	Intervention	Success
*Resin Infiltration*									
Kim et al. (2011) *South Korea*	Prospective cohort	Mild	0.25	Mean 12.5	12 (0)	20	Complete masking as detected by colour change using photographic evaluation, CIE L*a*b* scoring method	Resin infiltration	25% completely masked, 35% partially masked, 40% unchanged
Elbaz & Mahfouz (2017) *Egypt*	Prospective cohort	Mild	1	Range 9–14	10 (0)	20	Colour change using photos and image analysing programme, assessment of radiographs	G1: Resin infiltration G2: NaF 6% varnish	G1: Mean color difference between sound and white spots significantly decreased, improvement in radiodensity G2: no change following treatment
Bhandari et al. (2018) *India*	Prospective cohort	Mild	6	Range 7–16	NR	22	Colour change using photographic evaluation, CIE L*a*b* scoring method	Resin infiltration	Overall colour change following treatment
*Microabrasion*									
Bhandari et al. (2019) *India*	Randomised trial	Mild	6	Range 7–16	NR	43	Colour change using photographic evaluation, CIE L*a*b* scoring method	G1: microabrasion pumice slurry 37% phosphoric acid G2: microabrasion and CPP-ACP at home for 6 months	Overall colour change following treatment in both groups

KEY: *NR* not reported, G-group, CIE L*a*b*—Commission on Illumination, CPP-ACP – casein phosphopeptide-amorphous calcium phosphate, *OHRQoL *oral health-related quality of life

### Findings for managing hypersensitivity

Four studies focussed on reducing sensitivity in 402 MIH-affected molars and incisors. The overall success rates and pertinent study characteristics are displayed in Table 3. Four studies looked at the reduction in hypersensitivity with a variety of topical modalities used. One study compared a combination of options that included products including 5% fluoride varnish, 10% casein phosphopeptide-amorphous calcium phosphate crème (CPP-ACP), 10% CPP-ACP crème with 900 ppm fluoride and ozone (Ozgul et al. 2013). One study compared 10% CPP-ACP crème against a control of 1000 ppm fluoride toothpaste (Pasini et al. 2018) as a placebo crème wasn’t available. Another study used 8% arginine and calcium carbonate toothpaste applied once professionally and then used twice daily at home. (Bekes et al. 2017). The remaining study compared, in isolation and combined, 5% fluoride varnish and low-level laser therapy (Muniz et al. 2020).

**Table 3:** Included studies on treatment for reduction of hypersensitivity

Study *Country*	Study Design	Tooth	Severity of MIH	Follow-up (months)	Age of Participants	No. of participants (drop outs)	No. of teeth	Primary outcome measure	Intervention	Success
Ozgul et al. (2013) *Turkey*	Randomised trial	I	Mild	3	Range 7–12	33 (0)	92	Cold stimulus with VAS pain scale	G1A: 5% NaF varnish G1B: 5% NaF varnish & ozone G2A: 10% CPP-ACP creme G2B: Ozone & CPP-ACP creme G3A: 10% CPP-ACP creme containing 900 ppm fluoride G3B: 10% CPP-ACP containing 900 ppm fluoride & ozone	Reduction in hypersensitivity in all groups. No difference between groups
Bekes et al. (2017) *Germany*	Non-randomised trial	M	Mild & Severe	2	Mean 8.2	19 (4)	56	Cold and mechanical stimulus with SCASS and WBFS	8% arginine & calcium carbonate paste professionally applied	Reduction in hypersensitivity
Pasini et al. (2018) *Italy*	Randomised tiral	M	Mild & Severe	3	Range 8–13	40 (0)	40	Cold and mechanical stimulus with SCASS and VAS pain scale	G1: Control (1000 ppm fluoride TP) G2: 10% CPP-ACP creme in custom tray, twice daily for 2 h	Reduction in hypersensitivity in test group
Muniz et al. (2020) *Brazil*	Randomised trial	M&I (115 M/99I)	Mild & Severe	1	Mean 8.89 SD ± 2.13 Range 8–12	66 (6)	214	Cold stimulus and PIFS	G1: Laser G2: 5% NaF varnish G3: 5% NaF varnish and laser	Overall reduction in hypersensitivity in all groups. FV with laser better than laser alone but no difference between FV and FV with laser. Laser immediate effect and FV late onset effect

KEY: *M *molar, *I* incisor, *NR* not reported, G-group, *TP *toothpaste, *SCASS *Schiff cold air sensitivity scale, *WBFS *Wong Baker Faces Scale, *PPIFS *Pimenta Pain Intensity Face Scale, *VAS *visual analogue scale, *CPP-ACP *casein phosphopeptide-amorphous calcium phosphate

### Findings for increasing mineral content

A potential increase in mineral content was investigated in four studies and on 458 MIH-affected molars and incisors. The results are presented in Table 4. One study used 4% fluoride varnish (Restrepo et al. 2016) and another 10% CPP-ACP crème (Baroni and Marchionni 2011) only. Another compared 10% CPP-ACP crème with 10% CPP-ACP crème containing 900 ppm fluoride (Bakkal et al. 2017). Finally, one study compared three preparations, 5% fluoride varnish, 5% fluoride varnish containing tricalcium phosphate, and 10% CPP-ACP crème (Biondi et al. 2017).

**Table 4:** Included studies on treatment for increasing mineral content

Study *Country*	Study Design	Tooth	Severity of MIH	Follow-up (months)	Age of Participants	No. of participants (drop outs)	No. of teeth	Primary outcome measure	Intervention	Success
Baroni & Marchionni (2011) *Italy*	Prospective cohort	M	Severe	36	Range 6–9	30 (0)	30	In vivo replicas, in vitro biopsy with SEM and ESEM-EDX analysis	10% CPP-ACP creme in disposable trays, 20 min every evening	Improvement in mineralisation, morphology and porosities in enamel. Reduction in carbon and significant increase in calcium and phosphate
Restrepo et al. (2016) *Brazil*	Randomised trial	I	Mild & Severe	1	Mean 10.25 SD ± 1.14 Range 9–12	51 (0)	51	Quantitative light fluorescence imaging	G1: control G2: 4 × applications 4% NaF varnish	No difference in fluorescence between groups
Bakkal et al. (2017) *Turkey*	Prospective cohort	M&I (155 M/140I)	Mild	1	Mean 9.9 SD ± 1.6 Range 7–12	38 (0)	285	Laser fluorescence	G1: 10% CPP-ACP creme G2: 10% CPP-ACP containing 900 ppm fluoride	Both groups had a reduction in LF readings but no difference between the groups
Biondi et al. (2017) *Argentina*	Prospective cohort	M&I (teeth NR)	Mild & Severe	1.5	Range 6–17	55 (0)	92	Laser fluoresence	G1: 5% NaF varnish G2: 10% CPP-ACP creme G3: 5% NaF varnish containing tricalcium phosphate (TCP)	Reduction in LF scores for all three groups in mild lesions only. NaF better at remineralising severe lesions and NaF with TCP bettter at remineralising mild

KEY: M – molar, I – incisor, NR – not reported, G-group, LF—laser fluorescence, QLF—quantitative light fluorescence, SEM—scanning electron microscopy, ESEM-EDEX -environmental scanning electron microscopy and energy dispersive X-ray spectrometry, CPP-ACP – casein phosphopeptide-amorphous calcium phosphate

### Findings for patient-centred outcome measures

The remaining two studies used a variety of patient-centred outcome measures, following treatment, as either a primary outcome measure (Jalevik and Klingberg 2012; Hasmun et al. 2020). The results can be found in Table 5. Two studies which were included in the main analysis (Rolim et al. 2020; Mejare et al. 2005), were not included in this evaluation as they studied patient-centred outcomes as a secondary outcome measure. One study investigated patient satisfaction, dental anxiety and fear before and after treatment, and the need for need for additional behaviour management techniques (Jalevik and Klingberg 2012). The other study formally recorded changes in oral health-related quality of life following treatment (Hasmun et al. 2020).

**Table 5:** Included studies on patient-reported outcomes following treatment

Study	Study Design	Severity of MIH	Follow-up in months (range)	Age of participants	No. of participants (drop outs)	No. of teeth	Primary outcome measure	Intervention	Success
Jalevik and Klingberg (2012) *Sweden*	Retrospective case control	Severe	108	18 at time of review	72 (5)	NR	CFSS-DS to measure dental fear and anxiety, DVSS satisfaction with dental care, dental health and behaviour management problems by reviewing records. Measured at age 9 and 18 and compared with 41 controls	*Over 9-year period* G1 MIH: restorations 26 (86%), extractions 7 (23%), both restorations and extractions 27 (90%) G2 control: restorations 12 (32%), extractions 1 (3%), both restorations and extractions 12 32(%)	Increased dental fear and anxiety in MIH group at age 9 At age 9, 9 × more treatment in MIH group vs control. Overall 4.2 × more treatment vs control Behaviour management problems higher in MIH group. No difference in satisfaction between groups
Hasmun et al. (2020) *UK*	Prospective cohort	NR	6	Mean 11 Range 7–16	103 (17)	Mean 3.2 per participant	OHRQoL using C-OHIP-SF19, SPCC physical appearance subscale, social acceptance subscale, global self-worth	Microabrasion (4.65%), resin infiltration (4.65%), tooth whitening (4.65%), composite resin restoration (2.32%), microabrasion & resin infiltration (54%), microabrasion & tooth whitening (9.3%), tooth whitening & microabrasion and/or resin infiltration (7%)	Improvement C-OHIP-SF19 score from 47.4 to 59.8 Improvement in SPCC physical subscale appearance. No changes for social acceptance subscale or global self-worth

KEY: *NR* not reported, *CFSS-D *Children’s Fear Survey Schedule-Dental Subscale, *DVSS *Dental Visit Satisfaction Scale, *C-OHIP-SF19 *Child Oral Health Impact Profile-Short Form 19, *SPCC *Harter’s Self-Perception Profile for Children

### Risk of bias assessment

**Fig. 2:**
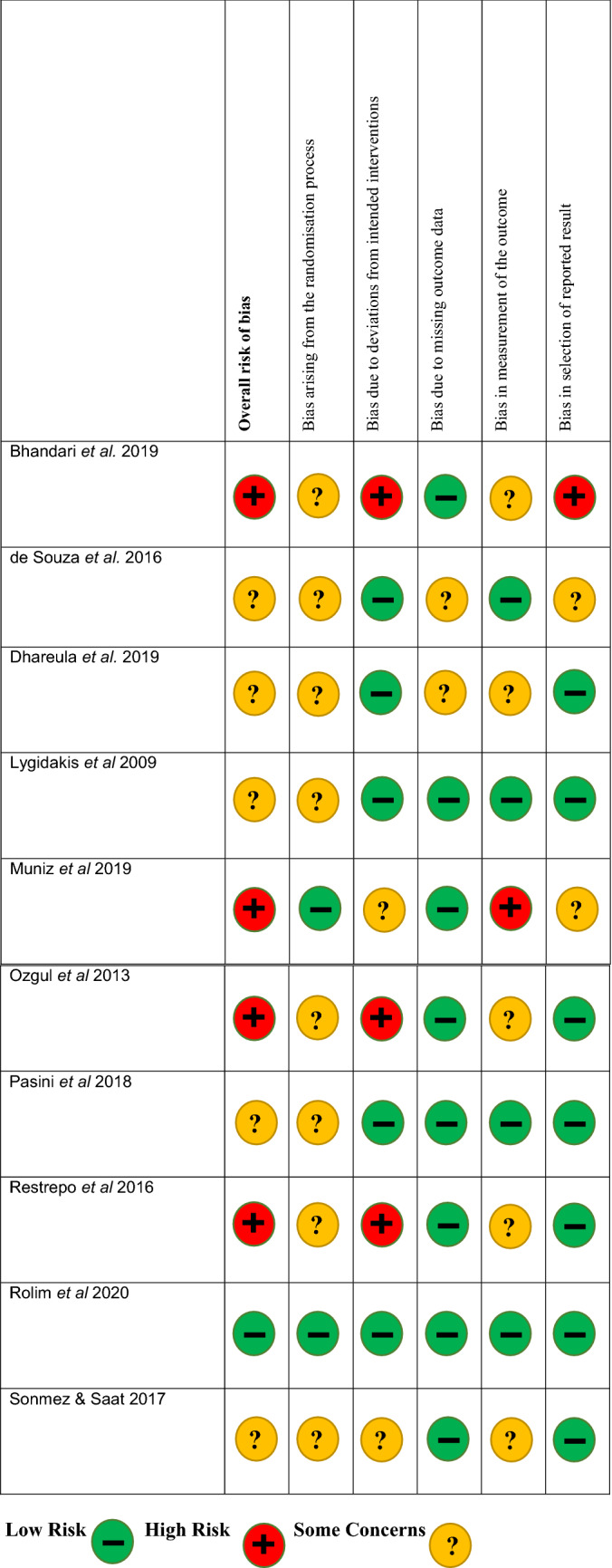
Rias of bias assessment for randomised trials

**Fig. 3:**
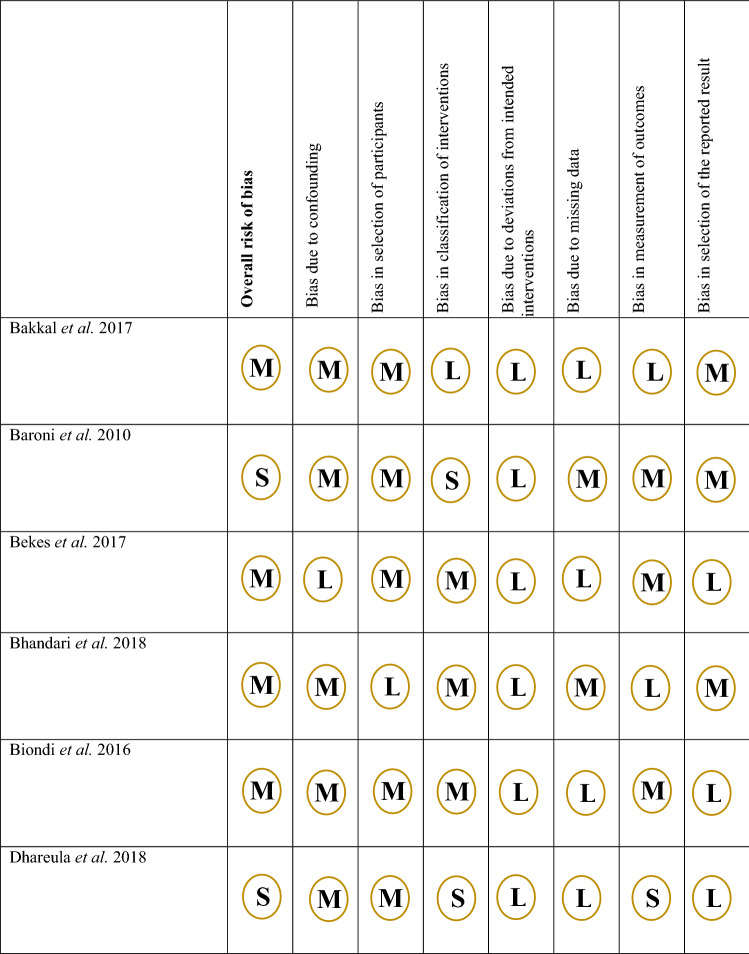

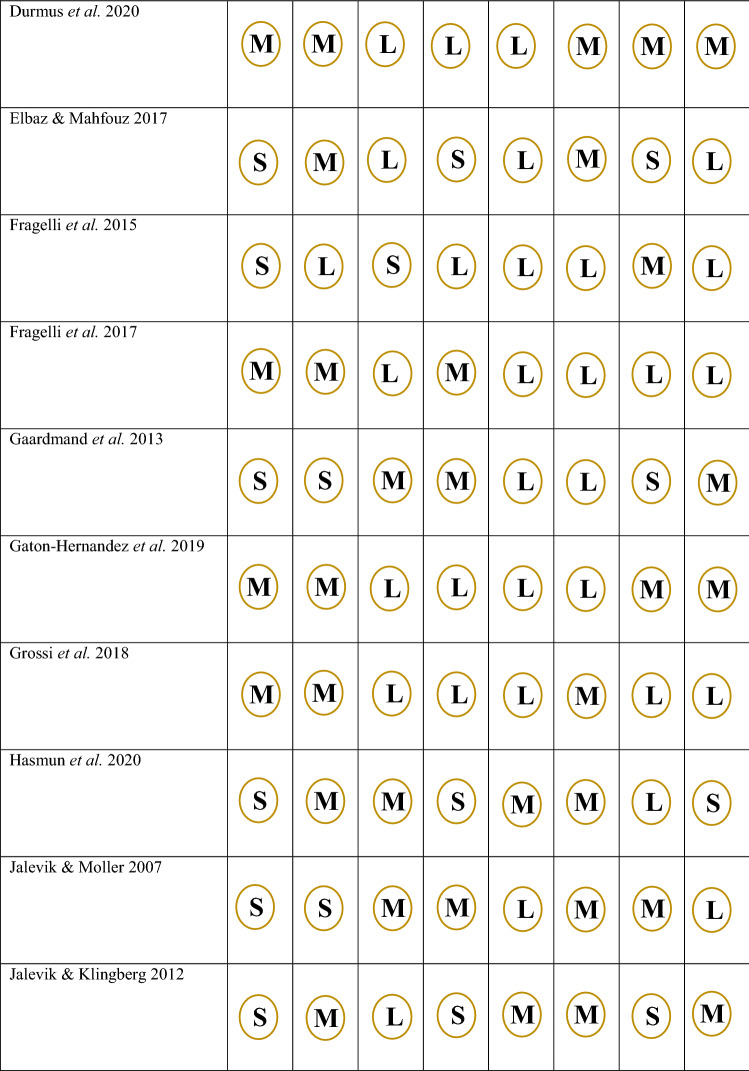

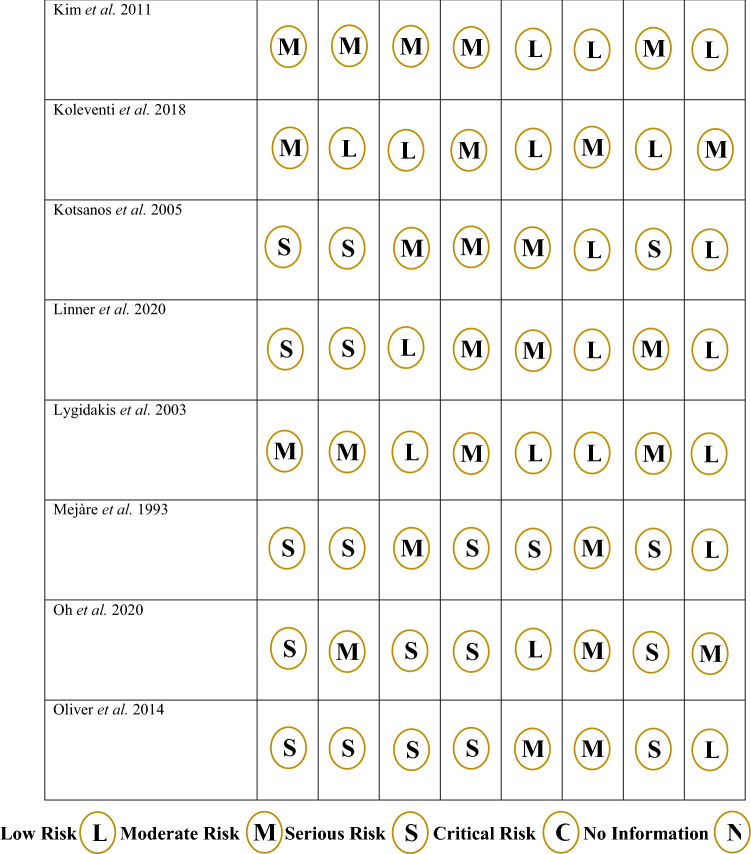
Rias of bias assessment for non-randomised trials

Risk of bias assessment was performed and a breakdown for each criterion is shown for non-randomised studies, in Fig. 2, and randomised studies in Fig. 3. For the 24 non-randomised studies, 13 were deemed to be high risk, 11 moderate risk and none were low risk. Bias due to confounding was a concern and 6 studies were judged to be high risk, 15 studies moderate risk and only 3 low risk. Conversely, the studies generally did not deviate from the intended interventions and 18 studies were judged to be low risk, 6 studies moderate risk and 1 study serious risk. Overall, for the randomised studies, only 1 of the 10 was judged to be low risk, 5 had some concerns and 4 were high risk. One domain where the studies had poor scores was in the randomisation process. Only 2 studies were ranked low and 8 showed some concerns. On the other hand, for missing outcome data, 8 studies were categorised as low and 2 studies showed some concerns.

## Discussion

There is a growing interest in addressing how best to manage MIH-affected teeth. Due to a lack of research in the area, previous systematic reviews discussing the management of MIH, (Lygidakis 2010) and a further review in 2016 (Elhennawy and Schwendicke) included studies which also investigated the management of other enamel defects. Both of these reviews included 14 studies, respectively. In comparison, this systematic review has only included studies that state they specifically managed teeth affected by MIH. A total of 34 studies were included thus confirming the recently increased number of studies and the expanding interest in this area of research. Furthermore, not only has the total number of studies more than doubled, there are also more randomised studies (n = 10) compared to those included in the previous reviews (Lygidakis 2010; Elhennawy and Schwendicke 2016). This demonstrates that researchers are looking to more methodologically robust approaches to understand the management of this condition better. Despite this increase, most included studies were at moderate or high risk of bias, suggesting that further well-designed randomised studies are needed.

### Management of posterior teeth

Fissure sealants are predominately used for fully erupted molars that have mild MIH. In this review, only three studies were included with a low total number of sealants placed (184). One of these did not state which material was used (Kotsanos et al. 2005a, b) whilst all three studies used different primary outcome measures. A significant difference in retention rate was noted in one study when an adhesive was applied prior to the placement of a resin-based fissure sealant (Lygidakis et al. 2009). This has been reported in non-MIH affected teeth (McCafferty and O’Connell 2016) although, further studies in relation to MIH would be merited. There was also variation in the technique with one study using a local anaesthetic infiltration and rubber dam isolation prior to the placement of the sealant (Fragelli et al. 2017) and another using cotton wool roll isolation (Lygidakis et al. 2009). Another possible confounding factor is that both aforementioned studies applied fluoride varnish to the treated teeth at different time intervals prior to the treatment. Despite showing moderate success rates, their application should be considered as a first line approach for these teeth given the potential for future PEB and/or caries lesion initiation. It is accepted that MIH-affected teeth are more prone to the development of carious lesions (Jeremias et al. 2013; Bullio Fragelli et al. 2015) and as such should form part of the preventive approach for any child at high risk (Ahovuo-Saloranta et al. 2016).

If an MIH-affected tooth is cavitated, due to PEB and/or dental caries, a restorative approach can be undertaken. However, consideration of the structure, chemical and mechanical properties of enamel and dentine as well as the extent of the lesion in MIH-affected teeth is essential when deciding which restorative material to use.

Two of the older studies used amalgam to restore MIH-affected molars, with 50 restorations being placed. Although one of the studies reported that 78% of the 32 restorations placed were acceptable, it has been suggested that amalgam should be avoided due a lack of adhesion and need for physical retention, which in conjunction with atypically shaped cavities are likely to increase further breakdown at the margins (Ghanim et al. 2017). Additionally, a European directive (Article 10 (2) of Regulation (EU) 2017/852 on Mercury) has advised against the use of amalgam in children under the age of 15 unless strictly necessary, which is likely to lead to a decline in its use in both research and clinical practice.

In contrast, eight studies used direct composite resin restorations in 793 molars, the majority of which were severely affected by MIH. In general, the success rates reported suggest this is an effective option for the management of MIH-affected molars where breakdown or carious lesions do not extend to the pulp, or do not present with irreversible pulpitis, even in the most severe of cases. Total or partial removal of the hypomineralised enamel, prior to restoring with composite resin under rubber dam isolation, remains a reliable technique in terms of success rates of the restorations. Linner et al. (2020) directly compared restoration with composite resin with and without removal of hypomineralised enamel, showing higher success rates of 78% when a conventional invasive approach was used over a more minimally invasive approach where only 29% of restorations where successful. However, the number of teeth managed by each approach in this study was vastly different, and therefore reduces the validity of the results (Linner et al. 2020). It is known that hypomineralised enamel has an increase in porosity, a reduction in hardness and elasticity and a change in carbon-carbonate ratios when compared to normal enamel (Elhennawy et al. 2017). Thus, if using a less invasive approach and leaving some MIH-affected enamel, adhesion will be poorer, with reduced bond strengths observed in vitro (Lagarde et al. 2020). However, recent advances in bonding techniques, and the suggestion of rinsing MIH-affected enamel with sodium hypochlorite before placing a composite resin restoration, are both likely to help increase bond strengths for these teeth (Sonmez and Saat 2017; Lagarde et al. 2020), although, some in vitro data supports the opposite (Ramakrishna et al. 2014; Krämer et al. 2018). Pre-treatment of MIH-affected enamel with an oxidative or proteolytic, prior to restoration, however, merits further investigation as a recent clinical study by Sonmez and Saat showed promising results when this was undertaken after a non-invasive cavity preparation (Sonmez and Saat 2017). A paradigm shift towards more minimally invasive approaches has been adopted in dental caries management (Banerjee 2017); however, such widespread change in attitudes has yet to be observed in treatment of MIH, as this approach may jeopardise the final result considering the nature of the enamel defect. Despite this, some clinicians have reported adopting minimally-invasive approaches in clinical practice for MIH-affected molars (Kopperud et al. 2016; Taylor et al. 2019; Dastouri et al. 2020), suggesting this is an area where more high-quality research studies are required to provide conclusive evidence for this approach (Alkhalaf et al. 2020). Differences in the restorative materials used across studies was observed. Whilst the majority of studies used conventional composite resin, a recent study used bulk-filled composite resin (Rolim et al. 2020) with another initially placing a provisional GIC restoration, which was later replaced with composite resin (de Souza et al. 2017). However, most of these studies did not report the number of surfaces restored or the presence or absence of caries lesions prior to restorative treatment and as such are significant confounding factors to consider when interpreting their results.

GIC restorations were used in five studies, where 333 restorations were placed with varying success rates noted. The heterogeneity in the included studies was high as all used different outcome measures. Two of the studies had a retrospective design, further increasing the likelihood of bias. The longevity of GIC restorations is influenced by factors such as the technique and type of GIC used, with those using high-viscosity (Durmus et al. 2020) or glass hybrid restorations (Grossi et al. 2018) reporting higher survival rates. One study, reporting a 98% cumulative survival, showed that MIH-affected molars can be restored with GIC using the atraumatic-restorative technique (ART) (Grossi et al. 2018), which negates the use of local anaesthesia. Fragelli et al. (2015) also reported a high cumulative survival of 78% using a non-invasive approach but Linner et al. (2020) only reported a 7% cumulative survival. The non-invasive approach may be beneficial in certain children with MIH due to the known difficulties in obtaining anaesthesia (Rodd et al. 2007) but the results are highly variable and dependant on patient co-operation. It has been suggested that GIC can be used as a permanent restorative material; however, the mean follow-up time in these studies was only 22.7 months so its long-term survival is not known. As other materials such as composite resin have been shown to be more reliable, in practice it is, therefore, often used as an interim measure until a definitive restoration can be placed when patient co-operation improves (Taylor et al. 2019; Wall and Leith 2020).

An alternative approach for severely affected molars is to use preformed metal crowns. Despite only three studies included in this review using PMC for MIH-affected molars, they outperformed all other restorative materials with one study reporting 86% success (Oh et al. 2020) and others reporting a 100% success rate (Kotsanos et al. 2005a, b; Koleventi et al. 2018). Preformed metal crowns may also be used occasionally as an interim measure until scheduled extraction (Taylor et al. 2019), especially as they have the advantage of maintaining the structural integrity of the tooth without causing any adverse symptoms (Lygidakis 2010). Across the three studies only 88 teeth were restored, with one of the studies also including patients with caries and other dental anomalies, thus increasing the risk of bias (Oh et al. 2020). Two of the three studies had a retrospective design (Kotsanos et al. 2005a, b; Oh et al. 2020) and all three studies used different outcome measures. Consideration should be given to the periodontal health when placing preformed metal crowns for MIH-affected molars. Koleventi et al. (2018) reported an increased periodontal pocket depth in the short-term. When used as an interim measure prior to scheduled extraction, this is not of clinical significance in children. However, more research is needed to understand the effects of preformed metal crowns on the periodontal tissues if they are to be used as a long-term option. Additionally, consideration should be given to the destructive nature when placed using a conventional technique that includes tooth preparation and the consequences of this, including reduced tooth tissue for bonding and retention of any future restorations. To overcome these issues, placing these crowns using the ‘Hall technique’ has been suggested (Innes et al. 2007), but further research is needed.

In general, high success rates were reported when indirect laboratory-made restorations were used, but the numbers included in these studies were relatively low, with 132 restorations placed. As four different restorative materials were used, (indirect composite resin onlays, minimally invasive cast metal or gold onlays, and CAD-CAM fabricated restorations) heterogeneity in these studies is high. All had a different technique for tooth preparation, restoration manufacture and cementation. On the other hand, in all studies, hypomineralised enamel was totally removed to ensure the restoration was bonded to clinically sound enamel. There was, however, variability in the margin finish with restorations being placed supra-, sub- and equi-gingivally depending on the defect. This may affect the periodontal tissues in the long-term; however, further research is required to confirm this. Interestingly, one of the studies successfully used nitrous oxide sedation with all of the participants in addition to using a putty or Essix retainer to cover the other affected teeth, to reduce tooth sensitivity, during tooth preparation (Gaardmand et al. 2013). Generally, indirect laboratory-made restorations could be considered as a better long-term option, especially in older children; however, studies included followed the participants up for no longer than 43 months, highlighting the need for longer studies. Drawbacks such as removal of more tooth tissue, difficulty to repair when damaged and higher cost have to be considered when making the decision to use such a technique (Zagdwon et al. 2003; Gaardmand et al. 2013; Dhareula et al. 2019).

For severely affected molars of poor long-term prognosis, extraction may be the only option. There is often an associated high burden of future treatment in a child with severely affected MIH-molars, and extraction should be considered (Lygidakis et al. 2010). In such cases, extraction at the optimum developmental stage between the ages of 8 and 10 has been advised (Ashley and Noar 2019). There were three extraction studies included this review, with variations on spontaneous space closure being the primary outcome measure used. Despite differences in measuring this, a wide range of success was reported, with complete space closure not guaranteed. The results should be interpreted with caution as all three studies had less than 80 participants with a wide variation in the age of the patients at extraction and orthodontic needs. In addition, two of three studies were retrospective. These results are, however, corroborated by Eichenberger et al. (2015﻿)﻿ who reported a 72% and 48% success rate of spontaneous space closure in the maxilla and mandible, respectively. Given this level of uncertainty, any scheduled extractions of MIH-affected molars are best planned following orthodontic evaluation. An assessment of the child’s underlying malocclusion, any hypodontia, the presence or absence of crowding, the presence of the third permanent molar and the dental developmental stage of the child is needed (Ashley and Noar 2019). However, should acute symptoms suggest immediate extraction is required, then a potential to develop a malocclusion must be accepted.

Pulp therapy in compromised first permanent molars is well documented; however, there is little evidence available specifically in MIH-affected molars. To our knowledge, no studies have yet been published and have, therefore, not been included in this review. A recent systematic review on compromised first permanent molars found that partial and coronal pulpotomies have high success rates, in the short- and long-term, but there is limited evidence available for conventional pulpectomy or regenerative techniques (Taylor et al. 2019). Clearly this is an area where further research is needed; however, partial or coronal pulpotomies may be considered as a potential treatment option in vital MIH-affected molars, while regenerative endodontics in non-vital immature molars, although a promising approach, needs further investigation (Tzanetakis et al. 2020).

It is evident from the included studies in this review that no one technique for the management of MIH-affected molars appears superior. There was significant heterogeneity amongst the studies mostly relating to the extent of MIH affecting each tooth. Variability (or non-recording) of the number of surfaces involved, extent of the breakdown, presence or absence of atypical or non-atypical caries lesions and presence or absence of previous restorations were often omitted. Furthermore, the outcome measures used across the studies significantly differed, some of which were not well-recognised or validated. Thus, comparing results across studies is difficult and introduces high levels of bias and is reflected in the risk of bias scores for these studies. Ultimately, deciding how to manage MIH-affected molars is challenging and complex, and management has been shown in various studies to differ greatly (Kopperud et al. 2016; Taylor et al. 2019; Dastouri et al. 2020; Wall and Leith 2020). Despite an increase, there still remains a lack of high-quality studies, and as such consideration to decide which approach to adopt should be based on the age of the child, co-operative ability, pulpal diagnosis, number of affected surfaces, number of teeth affected, underlying malocclusion, ability to access and pay for treatment and the need for general anaesthetic and the impact this may have on the treatment plan (Lygidakis et al. 2010).

### Management of anterior teeth

Due to the strict inclusion criteria of this review, studies on the management of MIH-affected anterior teeth were limited, despite the known psychosocial effects associated with these teeth (Rodd et al. 2011). Only four studies were included in this review, with the most common approach being resin infiltration. This is a non-invasive technique that aims to improve the translucency, optical properties and overall appearance of affected incisors (Crombie et al. 2014). Despite being a promising approach in MIH-mildly affected incisors, the results from the included studies show variable success rates based on colour change assessment alone. Using an outcome measure that addresses quality of life, or patient satisfaction, might be more appropriate to determine success. In addition, three of these included studies had small participant numbers with follow-up periods of less than 6 months (Kim et al. 2011; Elbaz and Mahfouz 2017; Bhandari et al. 2018). Similar methodological limitations were noted in the one study that used microabrasion (Bhandari et al. 2019). As a technique, microabrasion with either 18% hydrochloric acid or 37% phosphoric acid appears to be effective for improving the aesthetic appearance of anterior opacities (Wong and Winter 2002). It can be used in the management of children and adolescents with MIH-affected incisors; however, case selection is important (Lygidakis 2010; Wallace and Deery 2015). Despite the lack of evidence found from this review, anecdotally both of these options can be considered for the management of MIH-affected incisor opacities; however, it does highlight the need for more robust clinical studies with longer follow-up periods to be more confident in using these approaches. One included study looked at a combination of approaches for managing MIH-affected incisors (Hasmun et al. 2020). The outcome measures used in this study focussed on assessment of oral health-related quality of life (OHRQoL) and self-concept, rather than quantitative changes in colour and appearance. This pragmatic study used a combination of microabrasion, resin infiltration, home use tooth whitening and composite resin restoration, with each participant having a tailored approach depending on their clinical need. An overall improvement in OHRQoL and appearance was reported by the authors.

Studies that assessed treatment options such as external bleaching, the etch bleach and seal technique and composite resin restorations were not present, despite these being viable options for the management of these teeth (Lygidakis 2010; Wallace and Deery 2015). As previously mentioned, the strict inclusion criteria used in this review could explain such omissions. Several factors should be considered before choosing a treatment option, or a combination of treatment options for managing MIH-affected incisors. These include the age of the child, cooperative-ability, size, colour and number of opacities, and the psychological impact these have on the child, both in the short- and long-term (Lygidakis et al. 2010; Monteiro et al. 2020).

The colour of the lesion is a key factor when determining which management strategy to use as it relates to the chemical and physical properties of the enamel. An in-vitro study found that in molars, brown enamel had a higher protein content than yellow or chalky enamel (Farah et al. 2010). A further in-vitro study looking at the surface layer characteristics in hypomineralised molars found, as with other studies, the colour is likely to indicate the mechanical properties of the lesion (Kumar et al. 2017). In addition, they reported a marked variation in the depth of the lesions, which is difficult to determine clinically, and must be taken into consideration when trying to determine what approach to take. Brown opacities may be removed using microabrasion (Wallace and Deery 2015), whereas yellowish stains are more readily removed using an etch bleach and seal technique (Wright 2002). White opacities can be more challenging to manage. Vital bleaching can help camouflage the white opacity by increasing the overall brightness of the natural tooth and reducing the contrast between stains and normal enamel (Denis et al. 2013); however, this alone may not be sufficient (Wallace and Deery 2015). Nevertheless, a European directive (Directive 2011/84/EU of the European Commission, October, 29th 2011) indicates that professional vital bleaching is not allowed for patients under 18 (products over 0.1% of hydrogen peroxide). Resin infiltration techniques have shown some promise for white opacities (Kim et al. 2011; Crombie et al. 2014; Elbaz and Mahfouz 2017; Bhandari et al. 2018). However, incomplete removal of the surface layer with the etchant is known to prevent complete penetration of the resin, thus making it look like the technique has failed (Kim et al. 2011). Other methods such as judicious removal of surface enamel with a bur may be possible but should be undertaken with caution.

The shortcomings of the included studies are apparent, and as such, future studies need to consider these when designing their studies. In particular, participant recruitment and an appropriate follow-up strategy to adopt should be given due consideration. In addition, deciding which outcome measure is the most appropriate to improve the external validity of the results, thus making them more easily translated into clinical practice.

### Management of hypersensitivity

Despite differences in clinical protocols and options used in the four included studies, a reduction in sensitivity for all MIH-affected teeth was noted for all approaches. This offers the clinician managing MIH with a greater armamentarium when sensitivity is present. All included studies had prospective designs (three of which were randomised trials) and all used similar and comparable outcome measures. However, they had relatively small number of participants, with follow-up periods of less than 3 months, which does reduce their generalisability as the long-term efficacy is unknown (Ozgul et al. 2013; Bekes et al. 2017; Pasini et al. 2018; Muniz et al. 2020). In addition, there is a need for the continued use of the intervention which may limit the use to those that have access to the products or are able to afford regular use. Furthermore, CPP-ACP based products cannot be used in those who are allergic to milk proteins.

### Increasing mineral content

Topical preparations including 10% CPP-ACP, 10% CPP-ACP with 900 ppm fluoride and fluoride varnish, with and without tricalcium phosphate, all showed success in remineralising MIH-affected teeth (Baroni and Marchionni 2011; Restrepo et al. 2016; Bakkal et al. 2017; Biondi et al. 2017). Some of these studies, however, had serious methodological limitations; therefore, the results must be interpreted with caution. Three studies used optical measurement including quantitative light fluorescence (QLF) (Restrepo et al. 2016) and laser fluorescence (DIAGNOdent) (Bakkal et al. 2017; Biondi et al. 2017) as the primary outcome measure. As yet, these measurements have not been validated as to whether they accurately measure mineral density in MIH-affected teeth in-vivo, although QLF has been validated in-vitro (Gambetta-Tessini et al. 2017). Only one study investigated the mineral content, rather than the appearance of hypomineralised teeth. Baroni and Marchionni (2011) studied biopsied hypomineralised enamel following use of 10% CPP-ACP crème in-vivo, by analysing samples in-vitro using scanning electron microscopy and energy dispersive X-ray spectrometry. They found an increase in mineralisation, morphology and porosities in the affected enamel. Increasing the mineral content of MIH-affected teeth may lead to improved physical strength of the affected enamel, symptoms of sensitivity and appearance; however, none of these studies have demonstrated a clinical change as reported by the patient. Clearly validated outcome measures are needed to verify an increased mineral content, following application of these products, before a firm recommendation for the use of these products to increase the mineral content of hypomineralised teeth and crucially, a clinical improvement, can be made.

### Patient-reported outcomes following treatment

Only two studies used patient-centred outcomes as the primary outcome measure. One study reported an overall improvement in OHRQoL and appearance following a combination of approaches to manage anterior opacities (Hasmun et al. 2020). However, the participants were only followed up for 6 months, meaning that long-term sustained changes were unable to be established. The other study reported that patient satisfaction, following treatment plans that included restorations or extractions in isolation, or in combination, was found to be good overall (Jalevik and Klingberg 2012). Children with MIH had 4.2 times more treatment episodes in comparison with individuals without MIH as well as increased behaviour management problems (Jalevik and Klingberg 2012). High levels of dental fear and anxiety were reported. This is likely due to having younger participants and a wider range of treatments, including extraction, included in their study. However, there was no difference in the levels of dental fear and anxiety between participants with and without MIH (Jalevik and Klingberg 2012). Although not included in the present analysis, Rolim et al. (2020) found a significant reduction in anxiety 12 months after treatment, which may be due to a reduction in sensitivity following treatment. Additional factors such as increased operator experience, the child becoming more accepting of treatment or becoming older may also explain a reduction in dental fear and anxiety. Overall, many different outcome measures were used across these two studies making comparisons quite challenging. However, they provide a valuable insight into the patient’s perspective. There are many benefits to using patient centred outcome measures including a reduction in observer bias, providing information that is exclusive to participants, increased public accountability and the appreciation felt of participants by being involved (Gilchrist and Marshman 2021).

Significant heterogeneity still exists as the outcome measures used varied significantly. Consequently, most of the included studies were at a moderate or high risk of bias. Whilst research into the use of CPP-ACP based products for hypomineralised teeth is increasing, only four articles for anterior teeth were included. Additionally, the generalisability of the included studies is limited as most were carried out in a specialist environment by experienced paediatric dentists. Investigation into the long-term outcomes of treatment and survival data was also lacking. A reporting bias may exist and studies with poor outcomes may not be reported in the literature.

Whilst the number of included studies addressing the management of MIH-affected teeth has been substantially increased since previous reviews (Lygidakis 2010; Elhennawy and Schwendicke 2016), there remains a paucity of research for treatment options including pulpal involvement and therapy, the use of silver diamine fluoride, and alternative management options for anterior teeth, including composite resin masking and veneers. Overall, an increase in prospective randomised clinical trials, across all management options, is needed to increase the quality of evidence available and to help inform the decision-making process. In addition, future research would benefit by additionally assessing the psychosocial impact (Rodd et al. 2011) using patient reported outcome measures (Gilchrist and Marshman 2021) and economic impact of each approach (Taylor et al. 2019). Developing a core outcome measure set for MIH, and including other outcomes such as severity, presence of carious lesion, extent and colour of MIH-lesions and/or behaviour management strategies used, would be of great benefit (Elhennawy et al. 2019). This would permit data pooling and meta-analysis, which was not possible in this review. In addition, future studies would also benefit from being carried out across different countries, and different healthcare settings, to include those with and without free healthcare provision and reduced resources, so that effective and equitable treatment of MIH is available to all children that need it, regardless of their social status and inequalities.

## Conclusion

- There is convincing evidence to support the use of resin-based fissure sealants, preformed metal crowns, direct composite resin restorations and laboratory-made restorations for MIH-affected molars in specific clinical scenarios.
- There is little evidence to support any approaches for affected anterior teeth.
- There is some evidence to support the use of products containing CPP-ACP which may be beneficial for MIH-affected teeth.
- An increase in the existing number of studies addressing the management of MIH-affected teeth has been observed, however, the majority have several methodological limitations and are at moderate or high risk of bias, which reduces the external validity of the results.
- There is a need for further high-quality studies with more participants, longer follow up periods and more clinically relevant and appropriate outcome measures in the management of MIH-affected teeth.

## Supplementary Information

Below is the link to the electronic supplementary material.

Supplementary file1 (DOCX 658 KB)
