# Supplementary material for: An update of treatment modalities in children and adolescents with teeth affected by molar incisor hypomineralisation (MIH): a systematic review
Source: Eur Arch Paediatr Dent. 2021 Jun 10;23(1):39–64. doi: 10.1007/s40368-021-00635-0 (PMC8927013; doi:10.1007/s40368-021-00635-0)
Supplement: Supplementary file 1 — Supplementary file1 (DOCX 658 KB) [file 40368_2021_635_MOESM1_ESM.docx]

**Appendix 1 – Excluded studies following title and/or abstract review**

Aalderink M.T., Nguyen H.P., Arzi B., et al (2015) Dental and temporomandibular joint pathology of the northern fur seal (Callorhinus ursinus). Journal of Comparative Pathology 152:325–334. <https://doi.org/10.1016/j.jcpa.2015.02.002>

Aaron L., Torsten M., Patricia W. (2019) Autoimmunity in celiac disease: Extra-intestinal manifestations. Autoimmunity Reviews 18:241–246. <https://doi.org/10.1016/j.autrev.2018.09.010>

Abadi BJ, Kimmel NA, Falace DA (1982) Modified overdentures for the management of oligodontia and developmental defects. ASDC journal of dentistry for children 49:123–126

Abanto Alvarez J, Rezende KMPC, Marocho SMS, et al (2009) Dental fluorosis: exposure, prevention and management. Medicina oral, patologia oral y cirugia bucal 14:E103

Abbas S., Maurya S.K., Dubey J., et al (2013) Developmental exposure to As, Cd, and Pb mixture diminishes skeletal growth and causes osteopenia at maturity via osteoblast and chondrocyte malfunctioning in female rats. Toxicological Sciences 134:207–220. <https://doi.org/10.1093/toxsci/kft093>

Abbott C., Verstraete F.J.M. (2005) The dental pathology of northern elephant seals (Mirounga angustirostris). Journal of Comparative Pathology 132:169–178. <https://doi.org/10.1016/j.jcpa.2004.09.007>

Abd Alraheam I, Donovan T (2020) Management of amelogenesis imperfecta in an adult patient: a short review and clinical report. British Dental Journal 229:239–243

Abdalla HE, Abuaffan AH, Kemoli AM (2020) Prevalence, pattern and distribution of MIH defects in Sudanese children

Abdat M (2018) PREVALENSI KARIES GIGI PERMANEN MOLAR PERTAMA DAN INSISIVUS PADA SISWA MIN 1 BAITURRAHMAN BANDA ACEH USIA 8-10 TAHUN. Journal Of Syiah Kuala Dentistry Society 2:76–81

Abdul Halim R (2012) Identification of factors in the natal and neonatal period influencing enamel development in the permanent first molars and incisors

Abdul Majid Z., Zain R.B. (1988) The dental health of factory workers in Pasir Gudang, Johor (Malaysia). Dental journal of Malaysia 10:38–41

Abdul Razak I, Nik Hussein NN (1986) Enamel defects in 11-12 year-old subjects in a fluoridated area. Dental journal of Malaysia 9:23–28

Abdullah A (2014) In vitro studies on the effect of remineralising treatments on hypomineralised enamel and subsequent orthodontic bonding

Abdullatif M (2012) A pilot study of the genotype and phenotype in Amelogenesis Imperfecta and Molar Incisor Hypomineralization

Aboelenein A.Z., Riad M.I., Haridy M.F. (2019) Case Report: In-office bleaching, microabrasion, and resin infiltration for the correction of hypomineralized esthetic defects. F1000 Research 8:. <https://doi.org/10.12688/F1000RESEARCH.19697.1>

Abolfazli N, Saleh-Saber F, Eskandari A, Lafzi A (2009) A comparative study of the long term results of root coverage with connective tissue graft or enamel matrix protein: 24-month results. Medicina oral, patologia oral y cirugia bucal 14:E304‐9

Abramov R., Fu G., Zhang Y., Peng C. (2013) Expression and regulation of miR-17a and miR-430b in zebrafish ovarian follicles. General and Comparative Endocrinology 188:309–315. <https://doi.org/10.1016/j.ygcen.2013.02.012>

Abravanel J (1974) [3 cases of opalescent dentin]. Revue de stomatologie et de chirurgie maxillo-faciale 74:424–433

Abuhagr AM, Blindert JL, Nimitkul S, et al (2014) Molt regulation in green and red color morphs of the crab Carcinus maenas: gene expression of molt-inhibiting hormone signaling components. The Journal of experimental biology 217:796–808. <https://doi.org/10.1242/jeb.093385>

Abu-Ta’a M. (2016) Adjunctive Systemic Antimicrobial Therapy vs Asepsis in Conjunction with Guided Tissue Regeneration: A Randomized, Controlled Clinical Trial. The journal of contemporary dental practice 17:3–6

Abu-Zahra R, Antos N-J, Kump T, Angelopoulou M-V (2019) Oral health of cystic fibrosis patients at a north american center: A pilot study. Medicina oral, patologia oral y cirugia bucal 24:e379. <https://doi.org/10.4317/medoral.22756>

Acevedo A.C., Alves P.G., de Lima C.L., et al (2015) Variability of systemic and oro-dental phenotype in two families with non-lethal Raine syndrome with FAM20C mutations. BMC Medical Genetics 16:. <https://doi.org/10.1186/s12881-015-0154-5>

Achamallah N., Miller M., Hosea S., Kuklinski L. (2019) Disseminated cocci packs a punch. American Journal of Respiratory and Critical Care Medicine 199:

ACTRN12619000483156 (2019) Group-based delivery of a parent-mediated intervention for infants with social communication delay. http://www.who.int/trialsearch/Trial2.aspx?TrialID=ACTRN12619000483156

Acunha T, Ibáñez C, Pascual Reguera MI, et al (2015) Potential of prodendronic polyamines with modulated segmental charge density as novel coating for fast and efficient analysis of peptides and basic proteins by CE and CE-MS. Electrophoresis 36:1564–1571. <https://doi.org/10.1002/elps.201400576>

Adam PM CONTRIBUTIONS TO THE STUDY OF DENTAL DYSTROPHIES IN THE MIXED DENTITION

Adenubi J.O. (1982) The acid-etch technique in children: an experience in Lagos. Journal of dentistry 10:235–242. <https://doi.org/10.1016/0300-5712(82)90055-0>

Adhikari AN, Heggie AAC, Shand JM, et al (2016) Infant Mandibular Distraction for Upper Airway Obstruction: A Clinical Audit. Plastic and reconstructive surgery Global open 4:e812. <https://doi.org/10.1097/GOX.0000000000000822>

Adyani-Fard D, Kim T-S, Eickholz P (2011) Interproximal bone loss at contra-lateral teeth with and without root canal filling in periodontitis patients. Journal of clinical periodontology 38:269–275. <https://doi.org/10.1111/j.1600-051X.2010.01657.x>

Affaitati G, Giamberardino M, Lapenna D, Costantini R (2018) Diclofenac epolamine topical patch for the treatment of pain. J Biol Regul Homeost Agents 32:435–441

Agamy H, Bakry N, Mounir M, Avery D (2004) Comparison of mineral trioxide aggregate and formocresol as pulp-capping agents in pulpotomized primary teeth. Pediatric dentistry 26:302‐309

Aggarwal VR (2008) Critical Appraisal: Bringing Together the Academic World and the ‘Real’World

Agrali Ö, Kuru B, Yarat A, Kuru L (2016) Evaluation of gingival crevicular fluid transforming growth factor-β1 level after treatment of intrabony periodontal defects with enamel matrix derivatives and autogenous bone graft: a randomized controlled clinical trial. Nigerian journal of clinical practice 19:535‐543. <https://doi.org/10.4103/1119-3077.183306>

Aguilar M.B., Quackenbush L.S., Hunt D.T., et al (1992) Identification, purification and initial characterization of the vitellogenesis-inhibiting hormone from the Mexican crayfish Procambarus bouvieri (Ortmann). Comparative Biochemistry and Physiology - B Biochemistry and Molecular Biology 102:491–498. <https://doi.org/10.1016/0305-0491%2892%2990039-T>

Aguiló L, Gandía JL (1998) Dentigerous cyst of mandibular second premolar in a five-year-old girl, related to a non-vital primary molar removed one year earlier: a case report. The Journal of clinical pediatric dentistry 22:155–158

Ahmad S, Petrou M, Alhumrani A, et al (2019) Prevalence of Molar-Incisor Hypomineralisation in an Emerging Community, and a Possible Correlation with Caries, Fluorosis and Socioeconomic Status. Oral health & preventive dentistry 1‐5. <https://doi.org/10.3290/j.ohpd.a42725>

Ahmadi R, Ramazani N, Nourinasab R (2012) Molar incisor hypomineralization: a study of prevalence and etiology in a group of Iranian children. Iranian journal of pediatrics 22:245

Ahmed AM, Nagy D, Elkateb MA (2019) Etching Patterns of Sodium Hypochlorite Pretreated Hypocalcified Amelogenesis Imperfecta Primary Molars: SEM Study. The Journal of clinical pediatric dentistry 43:257–262. <https://doi.org/10.17796/1053-4625-43.4.6>

Ahmed ATM (2020) Prevalence, Clinical Presentation, and Associated Sociodemographic Characteristics of Molar Hypomineralization in Indiana, USA

Ahovuo-Saloranta A, Forss H, Walsh T Consultorio Dental M & L

Aimetti M, Ferrarotti F, Mariani G, et al Enamel Matrix Derivative Proteins in Combination with a Flapless Approach for Periodontal Regeneration of Intrabony Defects: A 2-Year Prospective Case Series. The International journal of periodontics & restorative dentistry 36:797–805. <https://doi.org/10.11607/prd.2842>

Aimetti M, Ferrarotti F, Mariani G, Romano F (2017) A novel flapless approach versus minimally invasive surgery in periodontal regeneration with enamel matrix derivative proteins: a 24-month randomized controlled clinical trial. Clinical oral investigations 21:327‐337. <https://doi.org/10.1007/s00784-016-1795-2>

Aimetti M, Pigella E, Romano F, Debernardi C (2005) Treatment of mandibular class II furcation defects by the use of amelogenins and autologous bone. Two case reports. Minerva stomatologica 54:583‐591

Aimetti M., Romano F., Pigella E., Piemontese M. (2007) Clinical evaluation of the effectiveness of enamel matrix proteins and autologous bone graft in the treatment of mandibular class II furcation defects: A series of 11 patients. International Journal of Periodontics and Restorative Dentistry 27:441–447

Aitken J, Kenilworth W (2004) Comment: Me too 2. management 31:9–12

Ajami S, Pakshir H, Samady H (2017) Prevalence and Characteristics of Developmental Dental Anomalies in Iranian Orofacial Cleft Patients. Journal of dentistry (Shiraz, Iran) 18:193–200

Ajlan SA (2015) Congenital adrenal hyperplasia with localized aggressive periodontitis and amelogenesis imperfecta. Congenital anomalies 55:186–190. <https://doi.org/10.1111/cga.12122>

Akhoon AB, Zameer S Scholars Journal of Dental Sciences (SJDS) ISSN 2394-4951 (Print)

Akin H, Tasveren S, Yeler DY (2007) Interdisciplinary approach to treating a patient with amelogenesis imperfecta: a clinical report. Journal of esthetic and restorative dentistry : official publication of the American Academy of Esthetic Dentistry . [et al] 19:131

AL DÍA P (2010) Hipomineralización incisivo-molar. Estado actual. Cient dent 7:19–28

Al Machot E, Hoffmann T, Lorenz K, et al (2014) Clinical outcomes after treatment of periodontal intrabony defects with nanocrystalline hydroxyapatite (Ostim) or enamel matrix derivatives (Emdogain): a randomized controlled clinical trial. Biomed research international 2014:786353. <https://doi.org/10.1155/2014/786353>

al-Nazhan S (1991) Two root canals in a maxillary central incisor with enamel hypoplasia. Journal of endodontics 17:469–471

Al Salem M, Ismail L (1987) Factors influencing visual outcome after cataract extraction among Arabs in Kuwait. The British journal of ophthalmology 71:458–461

Al Zain T.J., Khalili H.M., Aboud S.H., et al (2002) Multiple intracranial hydatidosis. Acta Neurochirurgica 144:1179–1185. <https://doi.org/10.1007/s00701-002-0987-5>

Alacam A (1989) Long term effects of primary teeth pulpotomies with formocresol, glutaraldehyde-calcium hydroxide and glutaraldehyde-zinc oxide eugenol on succudaneous teeth. Journal of pedodontics 13:

Alaluusua S (2010) Aetiology of Molar-Incisor Hypomineralisation: A systematic review. European archives of paediatric dentistry : official journal of the European Academy of Paediatric Dentistry 11:53–58

Alaluusua S (2012) Defining developmental enamel defect-associated childhood caries: where are we now? Journal of dental research 91:525–527

Alaluusua S, Calderara P, Gerthoux PM, et al (2004) Developmental dental aberrations after the dioxin accident in Seveso. Environmental health perspectives 112:1313–1318

Alaluusua S, Lukinmaa P (2006) Developmental dental toxicity of dioxin and related compounds—a review. International dental journal 56:323–331

Alanzi A, Faridoun A, Kavvadia K, Ghanim A (2018) Dentists’ perception, knowledge, and clinical management of molar-incisor-hypomineralisation in Kuwait: a cross-sectional study. BMC oral health 18:34. <https://doi.org/10.1186/s12903-018-0498-2>

Alaoui ML, El Khammal H, Chhoul H Pediatric Preformed Metal Crown: Pedodontist’s Best Friend

Alazmah A (2017) Early Childhood Caries: A Review. The journal of contemporary dental practice 18:732–737

Al-Azri K, Melita LN, Strange AP, et al (2016) Optical coherence tomography use in the diagnosis of enamel defects. Journal of Biomedical Optics 21:036004

Al-Azri KMH (2015) The investigation of Optical Coherence Tomography as a clinical tool to determine the extent of Molar Incisor Hypomineralisation (MIH) Lesions

Al-Bahar H, Kandiah P, Toumba K School of Dentistry

Al-Batayneh O, AlJamal G, AlTawashi E (2014) Pre-eruptive intracoronal dentine radiolucencies in the permanent dentition of Jordanian children. European Archives of Paediatric Dentistry 15:229–236

Al-Batayneh OB (2012) Tricho-dento-osseous syndrome: diagnosis and dental management. International journal of dentistry 2012:

Al-Batayneh O.B., AlJamal G.A., AlTawashi E.K. (2014) Pre-eruptive intracoronal dentine radiolucencies in the permanent dentition of Jordanian children. European archives of paediatric dentistry : official journal of the European Academy of Paediatric Dentistry 15:229–236. <https://doi.org/10.1007/s40368-013-0104-x>

Al-Batayneh OB, AlTawashi EK (2020) Pre-eruptive intra-coronal resorption of dentine: a review of aetiology, diagnosis, and management. European archives of paediatric dentistry : official journal of the European Academy of Paediatric Dentistry 21:1–11. <https://doi.org/10.1007/s40368-019-00470-4>

Alberth M., Nemes J., Radics T., Kiss C. (1997) Egy amegakaryocytas thrombocytas purpurahoz tarsult zomancfejlodesi rendellenesseg eseteMegakaryocytic thrombopenic purpura associated with enamel hypoplasia. Fogorvosi szemle 90:131–135

Albino LGB, Rodrigues JA, Kawano Y, Cassoni A (2011) Knoop microhardness and FT-Raman evaluation of composite resins: influence of opacity and photoactivation source. Brazilian oral research 25:267–273

Al-Buainain F., Al-Ghatam R. (2019) Oral-facial-digital syndrome type i. Bahrain Medical Bulletin 41:109–112

Alcântara AP da S (2014) Sensibilidade dolorosa em pacientes escolares portadores de hipomineralização do molar incisivo

Alcock NW, Rd JA (1969) A model for studying the initiation of normal calcification in vivo. The Biochemical journal 112:511–513

Alcorn C., Tirupathi S. (2017) Incontinenta pigmenti-a case series. Developmental Medicine and Child Neurology 59:74. <https://doi.org/10.1111/dmcn.13623>

Aldin P, Tougne JP, Fortier JP (1976) [A method of reconstitution of dyplastic permanent incisors in the child]. Pedodontie Francaise 10:137–147

Aldin P., Tougne J.P., Fortier J.P. (1976) Une methode de reconstitution des incisives permanentes dysplasiees chez l’enfantA method of reconstitution of dyplastic permanent incisors in the child. Pedodontie Francaise 10:137–147

Aldred MJ, Crawford PJ (1988) Variable expression in Amelogenesis imperfecta with taurodontism. Journal of oral pathology 17:327–333

Aldred M.J., Crawford P.J. (1988) Variable expression in Amelogenesis imperfecta with taurodontism. Journal of oral pathology 17:327–333

Aldred MJ, Savarirayan R, Lamandé SR, Crawford PJM (2002) Clinical and radiographic features of a family with autosomal dominant amelogenesis imperfecta with taurodontism. Oral diseases 8:62–68

Alex R., Panza G., Hakim H., et al (2019) Mild intermittent hypoxia increases loop gain and the arousal threshold in participants with obstructive sleep apnea. American Journal of Respiratory and Critical Care Medicine 199:

Alex R.M., Panza G.S., Hakim H., et al (2019) Exposure to mild intermittent hypoxia increases loop gain and the arousal threshold in participants with obstructive sleep apnoea. Journal of Physiology 597:3697–3711. <https://doi.org/10.1113/JP277711>

Alex R.M., Panza G.S., Mateika J.H., Lin H.-S. (2018) Mild intermittent hypoxia improves cardiovascular and neurocognitive function in obstructive sleep apnea patients. FASEB Journal 32:

Alexander SA (1984) The treatment of hypocalcified amelogenesis imperfecta in a young adolescent. The Journal of pedodontics 9:95–100

Alexandru O, Andreea I, Emilia O, Magda L Unconventional Non-Invasive Diagnostic Techniques and Treatment of White Spot Lesions in Paediatric Dentistry and Orthodontics

Alexiou A, Vouros I, Menexes G, Konstantinidis A (2017) Comparison of enamel matrix derivative (Emdogain) and subepithelial connective tissue graft for root coverage in patients with multiple gingival recession defects: a randomized controlled clinical study. Quintessence international (berlin, germany : 1985) 48:381‐389. <https://doi.org/10.3290/j.qi.a38058>

Alfaro Alfaro A, Castejón Navas I, Magán Sánchez R, Alfaro Alfaro M (2018) Síndrome de hipomineralización incisivo-molar. Pediatría Atención Primaria 20:183–188

Al-Hammad NS Journal Homepage:-www. journalijar. com

Al-Hammad NS, Al-Dhubaiban M, Alhowaish L, Bello LL (2018) Prevalence and Clinical Characteristics of Molar-Incisor-Hypomineralization in School Children in Riyadh, Saudi Arabia

Al-Hezaimi K., Al-Fahad H., O’Neill R., et al (2012) The effect of enamel matrix protein on gingival tissue thickness in vivo. Odontology 100:61–66. <https://doi.org/10.1007/s10266-011-0022-5>

Alhezaimi K., Al-Shalan T., O’Neill R., et al (2009) Connective tissue-cementum regeneration: a new histologic regeneration following the use of enamel matrix derivative in dehiscence-type defects. A dog model. The International journal of periodontics & restorative dentistry 29:425–433

Al-Hezaimi K, Rudek I, Al-Hamdan KS, et al (2013) Efficacy of acellular dermal matrix and coronally advanced flaps for the treatment of induced gingival recession defects: a histomorphometric study in dogs. Journal of periodontology 84:1172–1179. <https://doi.org/10.1902/jop.2012.120380>

Ali M, Okamoto M, Komichi S, et al (2019a) Lithium-containing surface pre-reacted glass fillers enhance hDPSC functions and induce reparative dentin formation in a rat pulp capping model through activation of Wnt/β-catenin signaling. Acta biomaterialia 96:594–604. <https://doi.org/10.1016/j.actbio.2019.06.016>

Ali N, Loughborough WW, Rodrigues JCL, et al (2019b) Computed tomographic and clinical features of pulmonary veno-occlusive disease: raising the radiologist’s awareness. Clinical radiology 74:655–662. <https://doi.org/10.1016/j.crad.2019.04.023>

Alifakioti E, Arhakis A, Oikonomidis S, Kotsanos N (2020) Structural and chemical enamel characteristics of hypomineralised second primary molars. European Archives of Paediatric Dentistry 1–6

Alkaabi A.M., AlHumaidan A.A., AlQarawi F.K., AlShahrani F.A. (2019) Esthetic smile rehabilitation of enamel hypomineralized teeth with E-max prosthesis: Case report. Saudi Dental Journal 31:. <https://doi.org/10.1016/j.sdentj.2019.02.017>

Alkan E, Parlar A (2011) EMD or subepithelial connective tissue graft for the treatment of single gingival recessions: a pilot study. Journal of periodontal research 46:637‐642. <https://doi.org/10.1111/j.1600-0765.2011.01381.x>

Alkan E, Parlar A (2013) Enamel matrix derivative (emdogain) or subepithelial connective tissue graft for the treatment of adjacent multiple gingival recessions: a pilot study. International journal of periodontics & restorative dentistry 33:619‐625. <https://doi.org/10.11607/prd.1337>

Alkhadra T (2017) A systematic review of the consequences of early extraction of first permanent first molar in different mixed dentition stages. Journal of International Society of Preventive & Community Dentistry 7:223

Alkhtib A, Ghanim A, Temple-Smith M, et al (2016) Prevalence of early childhood caries and enamel defects in four and five-year old Qatari preschool children. BMC oral health 16:73. <https://doi.org/10.1186/s12903-016-0267-z>

Allazzam SM, Alaki SM, El Meligy OAS (2014) Molar incisor hypomineralization, prevalence, and etiology. International journal of dentistry 2014:234508. <https://doi.org/10.1155/2014/234508>

Allwani V., Pawar M., Abrar S., et al (2017) Permanent maxillary central incisor with dilacerated crown and root and C-shaped root canal. Journal of Clinical and Diagnostic Research 11:. <https://doi.org/10.7860/JCDR/2017/27742.10191>

Al-Malik M.I. (2004) The dentofacial features of Sanjad-Sakati syndrome: A case report. International Journal of Paediatric Dentistry 14:136–140. <https://doi.org/10.1111/j.1365-263X.2004.00527.x>

Almaz ME, Sönmez IS, Oba AA (2017) Prevalence and distribution of developmental dental anomalies in pediatric patients. Meandros Medical and Dental Journal 18:130

Al-Mozany S (2012) Contemporary treatment of Class II maxillary deficiency malocclusion in growing patients. Orthodontic Journal 28:111

Almuallem Z, Busuttil-Naudi A (2018a) Molar incisor hypomineralisation (MIH) - an overview. British dental journal. <https://doi.org/10.1038/sj.bdj.2018.814>

Almuallem Z, Busuttil-Naudi A (2018b) Molar incisor hypomineralisation (MIH)–an overview. British dental journal 225:601–609

Al-Mullahi AM, Toumba KJ (2016) Regional Odontodysplasia with Generalised Enamel Defect. Case reports in dentistry 2016:4574673. <https://doi.org/10.1155/2016/4574673>

Al-Mutawa S, Mathews B, Salako N (2002) Oral findings in Alagille syndrome. A case report. Medical principles and practice : international journal of the Kuwait University, Health Science Centre 11:161–163

Al-Obaida M (2020) A Rare Case of Nonsyndromic Generalized Radiculomegaly with a Literature Review. Case Reports in Dentistry 2020:

Al-Obaidi R (2018) In vitro enamel subsurface lesions: characterization and treatment

Alonso V, Caserio M (2012) A clinical study of direct composite full-coverage crowns: long-term results. Operative dentistry 37:432–441. <https://doi.org/10.2341/11-229-S>

Alosaimy S., Abdul-Mutakabbir J.C., Kebriaei R., et al (2020) Evaluation of Eravacycline: A Novel Fluorocycline. Pharmacotherapy 40:221–238. <https://doi.org/10.1002/phar.2366>

Alqadi A, O’Connell AC (2018) Parental perception of children affected by amelogenesis imperfecta (AI) and dentinogenesis imperfecta (DI): a qualitative study. Dentistry journal 6:65

Alqarni M.A., Alharbi A., Merdad L. (2018) Dental management of a patient with 22q11.2 deletion syndrome (22q11.2DS). BMJ Case Reports 2018:. <https://doi.org/10.1136/bcr-2018-225765>

Al-Salehi SK, Dooley K, Harris IR (2009) Restoring function and aesthetics in a patient previously treated for amelogenesis imperfecta. The European journal of prosthodontics and restorative dentistry 17:170–176

Alshehhi A, Al Halabi M, Hussein I, et al (2020) Enamel defects and caries prevalence in preterm children aged 5-10 years in Dubai. Libyan Journal of Medicine 15:1705633

AlShehri A (2016) Effect of innovative double layer treatment on tooth color change and nitrate penetration

Altun C, Cehreli ZC, Güven G, Acikel C (2009) Traumatic intrusion of primary teeth and its effects on the permanent successors: a clinical follow-up study. Oral surgery, oral medicine, oral pathology, oral radiology, and endodontics 107:493–498. <https://doi.org/10.1016/j.tripleo.2008.10.016>

Altun C., Esenlik E., Tozum T.F. (2009) Hypoplasia of a permanent incisor produced by primary incisor intrusion: a case report. Journal (Canadian Dental Association) 75:215–218

Aluja Jaramillo F, Gutierrez FR, Díaz Telli FG, et al (2018) Approach to Pulmonary Hypertension: From CT to Clinical Diagnosis. Radiographics : a review publication of the Radiological Society of North America, Inc 38:357–373. <https://doi.org/10.1148/rg.2018170046>

Alvarez L, Hermida L (2009) Hipomineralización molar-incisiva (MIH): una patología emergente. Odontoestomatología 11:4–11

Alvarez Ochoa D, Robles Contreras I, Díaz Meléndez J, Sandoval Vidal P (2017) Abordaje Terapéutico de la Hipomineralización Molar-Incisal. Revisión Narrativa. International journal of odontostomatology 11:247–251

Alves N., Cantin M. (2014) Clinical and radiographic maxillofacial features of pycnodysostosis. International Journal of Clinical and Experimental Medicine 7:492–496

Amaral de Mascena Costa L., Aguiar Coelho Teixeira A., Cassio Silva de Lima F., et al (2020) Cytotoxic Activity of the Mesoionic Compound MIH 2.4Bl in Breast Cancer Cell Lines. Breast Cancer: Basic and Clinical Research 14:. <https://doi.org/10.1177/1178223420913330>

Amarante E, Leknes K, Skavland J, Lie T (2000) Coronally positioned flap procedures with or without a bioabsorbable membrane in the treatment of human gingival recession. Journal of periodontology 71:989‐998. <https://doi.org/10.1902/jop.2000.71.6.989>

Amend S, Nossol C, Bausback-Schomakers S, et al (2020) Prevalence of molar-incisor-hypomineralisation (MIH) among 6–12-year-old children in Central Hesse (Germany). Clinical Oral Investigations 1–8

American Academy of Pediatric Dentistry (2013) Guideline on dental management of heritable dental developmental anomalies. Pediatric dentistry 35:E179

American Academy on Pediatric Dentistry Council on Clinical Affairs (2008) Guideline on oral heath care/dental management of heritable dental development anomalies. Pediatric dentistry 30:

Americano GCA (2014) Hipomineralização de molares e incisivos e necessidade de tratamento operatório em dentes permanentes

Americano GCA, Jorge RC, Moliterno LF, Soviero VM (2016) Relating Molar Incisor Hypomineralization and Caries Experience Using the Decayed, Missing, or Filled Index. Pediatric dentistry 38:419–424

Aminabadi N, Huang B, Samiei M, et al (2016) A Randomized Trial Using 3Mixtatin Compared to MTA in Primary Molars with Inflammatory Root Resorption: a Novel Endodontic Biomaterial. Journal of clinical pediatric dentistry 40:95‐102. <https://doi.org/10.17796/1053-4628-40.2.95>

Aminabadi N, Taghizdeh Gangi A, Balayi E, Sadighi M (2007) Prevalence of Fluorosis in 5-12 Year-old Children in the North-Western Villages of Makoo in 2004. Journal of dental research, dental clinics, dental prospects 1:33–41. <https://doi.org/10.5681/joddd.2007.006>

Aminabadi NA, Farahani RMZ, Gajan EB (2008) Apgar index as a correlate of enamel defects of primary dentition. Oral health & preventive dentistry 6:331–335

Aminabadi N.A., Pourkazemi M., Oskouei S.G., Jamali Z. (2010) Dental management of oculodentodigital dysplasia: a case report. Journal of oral science 52:337–342. <https://doi.org/10.2334/josnusd.52.337>

Aminabadi NA, Sighari Deljavan A, Samiei M, Jamali Z (2013) Are referred inaccessible human primary molar teeth really inaccessible? Journal of oral science 55:167–173

Amine M, Benfaida S, Elfariati H, et al (2019) The effect of structural abnormalities on dental substrates adhesion: A systematic literature review. Oral Health 4:1–7

Anan Z., Haoxiao J., Jianmin X., et al (2018) Survival and late effects of comprehensive treatment in children with neuroblastoma. Pediatric Blood and Cancer 65:. <https://doi.org/10.1002/pbc.27455>

Anastasiou A.D., Brown A.P., Jha A., et al (2017) Use of femtosecond lasers for exogenous mineralization of dental enamel. European Biophysics Journal 46:. <https://doi.org/10.1007/s00249-017-1222-x>

Andaloro C, Sessa C, Bua N, Mantia IL (2018) Chronic kidney disease in children: Assessment of oral health status. Dental and medical problems 55:23–28. <https://doi.org/10.17219/dmp/81747>

Anderegg C, Metzler D, Nicoll B (1995) Gingiva thickness in guided tissue regeneration and associated recession at facial furcation defects. Journal of periodontology 66:397‐402. <https://doi.org/10.1902/jop.1995.66.5.397>

Anders K, Simmons C (2019) Growing evidence that the world mosquito program’s wolbachia method reduces dengue transmission. American journal of tropical medicine and hygiene 101:251‐252. <https://doi.org/10.4269/ajtmh.abstract2019>

Ando M, Masuda T, Yamasue M, et al (2018) A case of maoto-induced interstitial pneumonia. Journal of thoracic disease 10:E485. <https://doi.org/10.21037/jtd.2018.05.82>

Andrade NS, Pontes AS, de Sousa Paz HE, et al (2017) Molar incisor hypomineralization in HIV‐infected children and adolescents. Special Care in Dentistry 37:28–37

Andrade NS, Tenório JR, Gallottini M (2019) Supernumerary teeth in a patient with Turner syndrome: An unusual finding. Special care in dentistry : official publication of the American Association of Hospital Dentists, the Academy of Dentistry for the Handicapped, and the American Society for Geriatric Dentistry 39:538–542. <https://doi.org/10.1111/scd.12412>

Andreasen J.O., Riis I. (1978) Influence of pulp necrosis and periapical inflammation of primary teeth on their permanent successors. Combined macroscopic and histologic study in monkeys. International Journal of Oral Surgery 7:178–187

Andre-Frei V, Chevallay B, Orly I, et al (2000) Acellular mineral deposition in collagen-based biomaterials incubated in cell culture media. Calcified tissue international 66:204–211

Andreou A., Kradolfer D., Candinas D., et al (2019) Minimal-invasive versus open hepatectomy for colorectal liver metastases: Bicentric analysis of postoperative outcomes and long-term survival using propensity score matching analysis. Swiss Medical Weekly 149:

Andreou A., Struecker B., Raschzok N., et al (2018) Minimal-invasive versus open hepatectomy for hepatocellular carcinoma: Comparison of postoperative outcomes and long-term survivals using propensity score matching analysis. Surgical Oncology 27:751–758. <https://doi.org/10.1016/j.suronc.2018.10.005>

Andrews P, Levine N, Milnes A, et al (1992) Advances in the treatment of acquired and developmental defects of hard dental tissues. Current opinion in dentistry 2:66–71

Andryjowicz E., Reinaldo Ruiz V., Rudolf J., et al (2015) Safely Increase the Minimally Invasive Hysterectomy Rate: A Novel Three-Tiered Preoperative Categorization System Can Predict the Difficulty for Benign Disease. The Permanente journal 19:39–45. <https://doi.org/10.7812/TPP/15-023>

Anemone R.L., Watts E.S., Swindler D.R. (1991) Dental development of known-age chimpanzees, pan troglodytes (primates, pongidae). American Journal of Physical Anthropology 86:229–241

Angelillo IF, Romano F, Fortunato L, Montanaro D (1990) Prevalence of dental caries and enamel defects in children living in areas with different water fluoride concentrations. Community dental health 7:229–236

Angelovska B, Drakalska E, Gligorovska A, Cvetkovski A (2018) Treatment of hypertension and complications in the Emergency Department-Delchevo. Knowledge-International Journal, Scientific Papers

Angmar-Mansson B., de Josselin de Jong E., Sundstrom F., ten Bosch J.J. (1994) Strategies for improving the assessment of dental fluorosis: focus on optical techniques. Advances in dental research 8:75–79. <https://doi.org/10.1177/08959374940080011301>

Angulo Ballen PA, Meneses Torres LF Hipomineralización inciso molar revisión narrativa

anonymous (2008) Antibacterials are an essential component in the treatment of meticillin-resistant Staphylococcus aureus (MRSA) skin infections. Drugs and Therapy Perspectives 24:10–13. <https://doi.org/10.2165/00042310-200824070-00003>

anonymous (2012) Yaws, a non-venereal treponemal infection: Still endemic in some parts of the world. Prescrire International 21:217–219

Antalovská Z, Skalská H, Jüttnerová V (1986a) [Prevention of tetracycline changes in permanent teeth using sodium fluoride]. Sbornik vedeckych praci Lekarske fakulty Karlovy univerzity v Hradci Kralove Supplementum 29:343–357

Antalovská Z, Skalská H, Jüttnerová V (1986b) [Prevention of development of tetracycline-induced changes in the teeth of children with the use of sodium fluoride]. Casopis lekaru ceskych 125:1317–1320

Anthonappa RP, King NM (2015) Enamel defects in the permanent dentition: prevalence and etiology. In: Planning and care for children and adolescents with dental enamel defects. Springer, pp 15–30

Anthonappa RP, Yiu CKY, King NM (2008) A novel combination of dens evaginatus and dens invaginatus in a single tooth--review of the literature and a case report. The Journal of clinical pediatric dentistry 32:239–242

Antos N.J., Kump T.E., Abu-Zahra R., Angelopoulou M. (2016) Time to see the dentist: Oral health status in pediatric cystic fibrosis patients. Pediatric Pulmonology 51:297. <https://doi.org/10.1002/ppul.23576>

Antunes A, Pinho Ribeiro A, Sabino E, et al (2016) Benznidazole therapy for Chagas disease in asymptomatic Trypanosoma cruzi-seropositive former blood donors: evaluation of the efficacy of different treatment regimens. Revista da sociedade brasileira de medicina tropical 49:713‐720. <https://doi.org/10.1590/0037-8682-0165-2016>

Aoba T., Fejerskov O. (2002) Dental fluorosis: Chemistry and biology. Critical Reviews in Oral Biology and Medicine 13:155–170. <https://doi.org/10.1177/154411130201300206>

Aoki H. (1989) Ultrastructural changes induced in rat ameloblasts and enamel by NaF administration, especially the stages of transition and maturation. Shika gakuho Dental science reports 89:1605–1637

Apaydin A, Sermet B, Ureturk S, Kundakcioglu A (2014) Correction of malocclusion and oral rehabilitation in a case of amelogenesis imperfecta by insertion of dental implants followed by Le Fort I distraction osteogenesis of the edentulous atrophic maxilla. BMC oral health 14:116. <https://doi.org/10.1186/1472-6831-14-116>

Apicella L, Cassis PR, Balestra B (2016) [Tooth-pick? Picking the Right Tooth]. Praxis 105:99–102. <https://doi.org/10.1024/1661-8157/a002245>

Appleton J (1995) Changes in the plasma electrolytes and metabolites of the rat following acute exposure to sodium fluoride and strontium chloride. Archives of oral biology 40:265–268

Appleton J., Eisenmann D.R., Mishima H., Engel M.B. (1992) Dentinogenesis and the calciotraumatic response to the injection of lead or fluoride ions. Scanning Microscopy 6:1073–1081

Apponi R., Presti S., Giannetti L., Spinas E. (2020) Biological, genetic and aetiology aspects in molar incisor hypomineralization. Journal of biological regulators and homeostatic agents 34:1219–1222. <https://doi.org/10.23812/20-148-L-61>

Arab M, Al-Sarraf E, Al-Shammari M, Qudeimat M (2019) Microshear bond strength of different restorative materials to teeth with molar-incisor-hypomineralisation (MIH): a pilot study. European archives of paediatric dentistry : official journal of the European Academy of Paediatric Dentistry 20:47–51. <https://doi.org/10.1007/s40368-018-0384-2>

Araújo MG, Lindhe J (1998) GTR treatment of degree III furcation defects following application of enamel matrix proteins. An experimental study in dogs. Journal of clinical periodontology 25:524–530

Araújo MVS (2019) Hipomineralização molar incisivo: tratamento restaurador e estético.

Aravind N., Pandiyan S. (2016) Demineralisation around orthodontic brackets- A review. Research Journal of Pharmacy and Technology 9:1536–1540. <https://doi.org/10.5958/0974-360X.2016.00301.2>

Arce-Izaguirre M, Torres-Ramos G, Alvino-Vales M, Barzola-Loayza M (2020) Giómeros fluidos en la eliminación de sensibilidad en molar permanente afectada con Hipomineralización Incisivo Molar (HIM). Reporte de caso. International journal of interdisciplinary dentistry 13:95–98

Ardini YD, Ismail NN, Azni NDM, Harun N (2019) Molar incisor hypomineralisation: Prevalence and associated risk factors among children at the Polyclinic, Kulliyyah of Dentistry, IIUM. Materials Today: Proceedings 16:2351–2356

Ardu S, Duc O, Krejci I, Perroud R (2013a) Amelogenesis imperfecta: a conservative and progressive adhesive treatment concept. Operative dentistry 38:235–241. <https://doi.org/10.2341/11-437-S>

Ardu S, Duc O, Krejci I, Perroud R (2013b) The author’s respond. Operative dentistry 38:676

Ardu S, Feilzer AJ, Devigus A, Krejci I (2008) Quantitative clinical evaluation of esthetic properties of incisors. Dental materials : official publication of the Academy of Dental Materials 24:333–340

Arenaz B., Maestro M.M., Jorge-Herrero E., et al (2004) Effects of periodate and chondroitin 4-sulfate on proteoglycan stabilization of ostrich pericardium. Inhibition of calcification in subcutaneous implants in rats. Biomaterials 25:3359–3368. <https://doi.org/10.1016/j.biomaterials.2003.09.105>

Arendt E.K., Moroni A., Zannini E. (2011) Medical nutrition therapy: Use of sourdough lactic acid bacteria as a cell factory for delivering functional biomolecules and food ingredients in gluten free bread. Microbial Cell Factories 10:. <https://doi.org/10.1186/1475-2859-10-S1-S15>

Arévalo Bernal M del C (2015) Incidencia de la hipomineralización inciso-molar en los pacientes atendidos en la clínica de odontopediatría en el período lectivo 2014-2015

Arid J, Oliveira DB, Evangelista SS, et al (2019) Oestrogen receptor alpha, growth hormone receptor, and developmental defect of enamel. International journal of paediatric dentistry 29:29–35. <https://doi.org/10.1111/ipd.12434>

Ariyamuthu V.K., Nolph K.D., Ringdahl B.E. (2013) Periodontal disease in chronic kidney disease and end-stage renal disease patients: A review. CardioRenal Medicine 3:71–78. <https://doi.org/10.1159/000350046>

Arkutu N, Gadhia K, McDonald S, et al (2012) Amelogenesis imperfecta: the orthodontic perspective. British dental journal 212:485–489. <https://doi.org/10.1038/sj.bdj.2012.415>

Arnadóttir IB, Sigurjóns H, Holbrook WP (2005) Enamel opacities in 8-year-old Icelandic children in relation to their medical history as infants. Community dental health 22:279–281

Arnetzl GV, Arnetzl G (2011) Adhesive techniques and machineable high-performance polymer restorations for amelogenesis imperfecta in mixed dentition. International journal of computerized dentistry 14:129–138

Aroca S, Keglevich T, Nikolidakis D, et al (2010) Treatment of class III multiple gingival recessions: a randomized-clinical trial. Journal of clinical periodontology 37:88‐97. <https://doi.org/10.1111/j.1600-051X.2009.01492.x>

Arrow P (2017) Dental enamel defects, caries experience and oral health-related quality of life: a cohort study. Australian dental journal 62:165–172. <https://doi.org/10.1111/adj.12449>

Arrow P. (2005) Enamel hypoplasia of the primary dentition in a 4-year-old with intestinal lymphangiectasia. International Journal of Paediatric Dentistry 15:380–384. <https://doi.org/10.1111/j.1365-263X.2005.00662.x>

Arrow P. (2013) Child oral health-related quality of life (COHQoL), enamel defects of the first permanent molars and caries experience among children in Western Australia. Community Dental Health 30:183–188. <https://doi.org/10.1922/CDH_3117Arrow06>

Arrow P (2008) Prevalence of developmental enamel defects of the first permanent molars among school children in Western Australia. Australian dental journal 53:250–259

Arrow P (2009) Risk factors in the occurrence of enamel defects of the first permanent molars among schoolchildren in Western Australia. Community dentistry and oral epidemiology 37:405–415. <https://doi.org/10.1111/j.1600-0528.2009.00480.x>

Artigas G.Q., Lapebie P., Leclere L., et al (2020) A G protein-coupled receptor mediates neuropeptide-induced oocyte maturation in the jellyfish Clytia. PLoS Biology 18:. <https://doi.org/10.1371/journal.pbio.3000614>

Artun J., Thylstrup A. (1989) A 3-year clinical and SEM study of surface changes of carious enamel lesions after inactivation. American journal of orthodontics and dentofacial orthopedics : official publication of the American Association of Orthodontists, its constituent societies, and the American Board of Orthodontics 95:327–333

Asano N, Schlötzer-Schrehardt U, Dörfler S, Naumann GO (1995) Ultrastructure of contusion cataract. Archives of ophthalmology (Chicago, Ill : 1960) 113:210–215

Asfar S, Shuaib A, Al-Otaibi F, et al (2018) A New Technique to Induce Experimental Myointimal Hyperplasia. Medical principles and practice : international journal of the Kuwait University, Health Science Centre 27:415–419. <https://doi.org/10.1159/000492575>

Ashkenazi M, Blumer S, Eli I (2010) Effect of computerized delivery intraligamental injection in primary molars on their corresponding permanent tooth buds. International journal of paediatric dentistry 20:270–275. <https://doi.org/10.1111/j.1365-263X.2010.01049.x>

Ashkenazi M., Sarnat H. (2000) Microabrasion of teeth with discoloration resembling hypomaturation enamel defects: four-year follow up. The Journal of clinical pediatric dentistry 25:29–34

Aslan S, Buduneli N, Cortellini P (2020) Clinical outcomes of the entire papilla preservation technique with and without biomaterials in the treatment of isolated intrabony defects: a randomised-controlled clinical trial. Journal of clinical periodontology

Aslan S, Buduneli N, Cortellini P Entire Papilla Preservation Technique: A Novel Surgical Approach for Regenerative Treatment of Deep and Wide Intrabony Defects. The International journal of periodontics & restorative dentistry 37:227–233. <https://doi.org/10.11607/prd.2584>

Asokan S., Muthu M.S., Rathna Prabhu V. (2007) Noonan syndrome: A case report. Journal of Indian Society of Pedodontics and Preventive Dentistry 25:144–147. <https://doi.org/10.4103/0970-4388.36567>

Aspden R.M., Saunders F.R. (2019) Osteoarthritis as an organ disease: From the cradle to the grave. European Cells and Materials 37:74–87. <https://doi.org/10.22203/eCM.v037a06>

Aspinall S.L., Leboeuf-Yde C., Walker B.F., Etherington S.J. (2019) Manipulation-induced hypoalgesia in musculoskeletal pain populations: A systematic critical review and meta-analysis. Chiropractic and Manual Therapies 27:. <https://doi.org/10.1186/s12998-018-0226-7>

Aspriello S, Ferrante L, Rubini C, Piemontese M (2011) Comparative study of DFDBA in combination with enamel matrix derivative versus DFDBA alone for treatment of periodontal intrabony defects at 12 months post-surgery. Clinical oral investigations 15:225‐232. <https://doi.org/10.1007/s00784-009-0369-y>

Assunção CM, Girelli V, Sarti CS, et al (2014) Hipomineralização de molar-incisivo (HMI): relato de caso e acompanhamento de tratamento restaurador. Revista da associação paulista de cirurgiões dentistas São Paulo Vol 68, n 4 (out/dez 2014), p 346-350

Assunção WG, Barão VAR, Kanno CM, et al (2009) Overdenture as a restorative option for hypocalcified-hypoplastic amelogenesis imperfecta: a case report. The journal of contemporary dental practice 10:67–73

Aswath N, Ramakrishnan SN, Teresa N, Ramanathan A (2018) A novel ROGDI gene mutation is associated with Kohlschutter-Tonz syndrome. Oral surgery, oral medicine, oral pathology and oral radiology 125:e8. <https://doi.org/10.1016/j.oooo.2017.09.016>

Atanas D, Tanja B, Dijan S, et al (2018) Elaboration of temporary constructions in fixed prosthodontics-a survey. Научни трудове на Съюза на учените–Пловдив Серия Г: Медицина, фармация и дентална медицина 22:

Atar M, Körperich EJ (2010) Systemic disorders and their influence on the development of dental hard tissues: a literature review. Journal of dentistry 38:296–306. <https://doi.org/10.1016/j.jdent.2009.12.001>

Atar M., Yasmin R., Sharma R., et al (2008) Of mice and mutations: phenotypic effects of the diabetic db/db and ob/ob mutations on the skull and teeth of mice. European archives of paediatric dentistry : official journal of the European Academy of Paediatric Dentistry 9:37–40. <https://doi.org/10.1007/BF03321594>

Atar S., Demir G.U., Dogan O.A., et al (2019) KAT6B mutations in OHDO syndrome. Gazi Medical Journal 30:

Athayde GS, Jorge RC, Americano G, et al (2020) Esthetic Management of Incisors with Diffuse and Demarcated Opacities: 24 Month Follow-up Case Report. Operative dentistry. <https://doi.org/10.2341/19-206-S>

Atia G-S, May J (2013) Dental fluorosis in the paediatric patient. Dental update 40:836–839

Atlan A, Denis M, Tirlet G, Attal J-P (2017) Orthodontics and enamel spots. Benefits of a minimally invasive approach, guidelines for the orthodontist. Journal of Dentofacial Anomalies and Orthodontics 20:302

Attal J-P, Atlan A, Denis M, et al (2014) White spots on enamel: treatment protocol by superficial or deep infiltration (part 2). International orthodontics 12:1–31. <https://doi.org/10.1016/j.ortho.2013.12.011>

Attar NB, Phadnaik MB (2009) Bilateral cervicoenamel projection and its management: A case report with lingual involvement. Journal of Indian Society of Periodontology 13:168–171. <https://doi.org/10.4103/0972-124X.60233>

Auger N, Low N, Lee G, et al Maternal Mental Disorders before Delivery and the Risk of Dental Caries in Children. Caries Research 1–8

Auster P (2013) Conquering a difficult case, part 2. Lithium disilicate as a restorative solution for amelogenesis imperfecta. Dentistry today 32:61–64

Avdic E., Cosgrove S.E. (2008) Management and control strategies for community-associated methicillin-resistant Staphylococcus aureus. Expert Opinion on Pharmacotherapy 9:1463–1479. <https://doi.org/10.1517/14656566.9.9.1463>

Avisa PA, Mallineni SK, Kamatham R, Nuvvula S (2017) Deciduous molar hypomineralization (DMH)–a rare entity and its clinical management approach. RSBO 14:94–97

Avsar A., Kalayci A.G. (2008) The presence and distribution of dental enamel defects and caries in children with celiac disease. Turkish Journal of Pediatrics 50:45–50

Aydemir Turkal H, Demirer S, Dolgun A, Keceli H (2016) Evaluation of the adjunctive effect of platelet-rich fibrin to enamel matrix derivative in the treatment of intrabony defects. Six-month results of a randomized, split-mouth, controlled clinical study. Journal of clinical periodontology 43:955‐964. <https://doi.org/10.1111/jcpe.12598>

Ayers K.M., Drummond B.K. (2003) Novel dental anomalies associated with congenital contractural arachnodactyly: a case report. Pediatric dentistry 25:501–504

Ayers KMS, Drummond BK, Harding WJ, et al (2004) Amelogenesis imperfecta--multidisciplinary management from eruption to adulthood. Review and case report. The New Zealand dental journal 100:101–104

Ayna E, Celenk S, Kadiroglu ET (2007) Restoring function and esthetics in 2 patients with amelogenesis imperfecta: case report. Quintessence international (Berlin, Germany : 1985) 38:51–53

Azevedo T.D., Feijo G.C., Bezerra A.C. (2006) Presence of developmental defects of enamel in cystic fibrosis patients. Journal of dentistry for children (Chicago, Ill) 73:159–163

Azpeitia-Valadez M de L, Rodríguez-Frausto M, Sánchez-Hernández MA (2008) [Prevalence of dental fluorosis in children between 6 to 15 years old]. Revista medica del Instituto Mexicano del Seguro Social 46:67–72

Azpeitia-Valadez M.L., Rodriguez-Frausto M., Sanchez-Hernandez M.A. (2008) Prevalencia de fluorosis dental en escolares de 6 a 15 anos de edadPrevalence of dental fluorosis in children between 6 to 15 years old. Revista medica del Instituto Mexicano del Seguro Social 46:67–72

Azzi L., Maurino V., Croveri F., et al (2017) Adult syndrome: Dental features of a very rare condition. Journal of Biological Regulators and Homeostatic Agents 31:61–65

B Hasmun NN, Drummond BK, Milne T, et al (2017) Effects of environmental tobacco smoke on the oral health of preschool children. European archives of paediatric dentistry : official journal of the European Academy of Paediatric Dentistry 18:393–398. <https://doi.org/10.1007/s40368-017-0308-6>

Baart JA, van Hagen JM, Swart-van der Berg M (2000) [Syndromes 22. Rieger’s syndrome]. Nederlands tijdschrift voor tandheelkunde 107:332–333

Babaei M., Ramezanpour A. (2018) Papillon-Lefevre syndrome in 4 years old patient: A rare case report and 3 years follow-up. Journal of Zanjan University of Medical Sciences and Health Services 26:124–131

Babajko S, Gayrard V, Houari S, et al (2020) [Oral cavity as a target and a marker of environmental exposures: developmental dental defects]. Medecine sciences : M/S 36:225–230. <https://doi.org/10.1051/medsci/2020024>

Babajko S., Jedeon K., Houari S., et al (2015) Bisphenol a affects amelogenesis through steroid hormone receptors. Endocrine Reviews 36:

Babajko S, Jedeon K, Houari S, et al (2017) Disruption of steroid axis, a new paradigm for molar incisor hypomineralization (MIH). Frontiers in Physiology 8:343

Babina K, Turkina A, Sokhova I, et al (2019) Comparative assessment of various composite materials and natural tooth tissue translucencies. The international journal of esthetic dentistry 14:406–419

Babu P World Journal of Pharmaceutical Sciences

Baccetti T (1998) A controlled study of associated dental anomalies. The Angle orthodontist 68:267–274

Bach K, Manton D (2014) Early childhood caries: a New Zealand perspective. Journal of Primary Health Care 6:169–174

Bachmann J (1985) [Amelogenesis imperfecta--orthodontic problems and their solution using multiband appliances and temporary steel crowns]. Fortschritte der Kieferorthopadie 46:66–71

Bachmann L., Sena E.T., Stolf S.F., Zezell D.M. (2004) Dental discolouration after thermal treatment. Archives of Oral Biology 49:233–238. <https://doi.org/10.1016/j.archoralbio.2003.08.005>

Badavannavar A, Ajari S, Nayak K, Khijmatgar S (2020) Abfraction: etiopathogenesis, clinical aspect, and diagnostic-treatment modalities: a review. Indian journal of dental research 31:305‐311. <https://doi.org/10.4103/ijdr.IJDR_863_18>

Bae CH, Lee JY, Kim TH, et al (2013) Excessive Wnt/β-catenin signaling disturbs tooth-root formation. Journal of periodontal research 48:405–410. <https://doi.org/10.1111/jre.12018>

Bae JS, Kim I-H, Choi H-J, Song JS (2020) Status and Survey of Pulp Treatment by Korean Pediatric Dentists. THE JOURNAL OF THE KOREAN ACADEMY OF PEDTATRIC DENTISTRY 47:277–292

Baelum V., Manji F., Fejerskov O. (1986) Posteruptive tooth age and severity of dental fluorosis in Kenya. Scandinavian journal of dental research 94:405–410

Bahadure RN, Thosar N, Kriplani R, et al (2012) Dental aspect of distal tubular renal acidosis with genu valgum secondary to rickets: a case report. Case reports in dentistry 2012:374945. <https://doi.org/10.1155/2012/374945>

Bahrololoomi Z, Amrollahi N, Mostafaloo N (2020) The Prevalence and Extent of Molar-Incisor Hypo-Mineralization by Gender in a Group of Iranian Children. Iranian Journal of Public Health

Bahrololoomi Z, Mostafalu N, Shakib A (2017) Relationship between Molar Incisor Hypomineralization with Body Mass Index and Dental Caries Index in 7-11 Year Old Children in Yazd City. SSU_Journals 25:11–19

Baker CM (2007) A new smile for a new life. Dentistry today 26:70

Baker KL (1975) Tetracycline-induced tooth changes. Part 5. Incidence in extracted first permanent molar teeth: a resurvey after four years. The Medical journal of Australia 2:301–304

Baker KL, Storey E (1970) Tetracycline-induced tooth changes. 3. Incidence in extracted first permanent molar teeth. The Medical journal of Australia 1:109–113

Bakry AS, Abbassy MA, Alharkan HF, et al (2018) A novel fluoride containing bioactive glass paste is capable of re-mineralizing early caries lesions. Materials 11:1636

Balhaddad A.A., Kansara A.A., Weir M.D., et al (2019) Toward dental caries: Exploring nanoparticle-based platforms and calcium phosphate compounds for dental restorative materials. Bioactive Materials 4:43–55. <https://doi.org/10.1016/j.bioactmat.2018.12.002>

Ball JS (1972) Minor dental disorders in childhood. The Practitioner 208:239–247

Balmer R., Fayle S.A.F. (2007) Enamel defects and ectopic eruption in a child with Usher syndrome and a cochlear implant. International Journal of Paediatric Dentistry 17:57–61. <https://doi.org/10.1111/j.1365-263X.2006.00778.x>

Balmer R, Toumba J, Godson J, Duggal M (2012) The prevalence of molar incisor hypomineralisation in Northern England and its relationship to socioeconomic status and water fluoridation. International journal of paediatric dentistry 22:250–257. <https://doi.org/10.1111/j.1365-263X.2011.01189.x>

Balmer R, Toumba KJ, Munyombwe T, Duggal MS (2015) A comparison of the presentation of molar incisor hypomineralisation in two communities with different fluoride exposure. European archives of paediatric dentistry : official journal of the European Academy of Paediatric Dentistry 16:257–264. <https://doi.org/10.1007/s40368-014-0170-8>

Balmer RC, Laskey D, Mahoney E, Toumba KJ (2005) Prevalence of enamel defects and MIH in non-fluoridated and fluoridated communities. European journal of paediatric dentistry 6:209–212

Banerjee S, Kumar D, Mukherjee CG, et al (2018) A clinical case report on management of MIH in twins. Chief Patron 4:35

Baratella E, Fiorese I, Marrocchio C, et al (2019) Imaging Review of the Lung Parenchymal Complications in Patients with IPF. Medicina (Kaunas, Lithuania) 55:. <https://doi.org/10.3390/medicina55100613>

Baratieri C, Alves M, Mattos CT, et al (2013) Changes of pulp-chamber dimensions 1 year after rapid maxillary expansion. American journal of orthodontics and dentofacial orthopedics : official publication of the American Association of Orthodontists, its constituent societies, and the American Board of Orthodontics 143:471–478. <https://doi.org/10.1016/j.ajodo.2012.10.022>

Baratieri L, Canabarro S, Lopes G, Ritter A (2003) Effect of resin viscosity and enamel beveling on the clinical performance of Class V composite restorations: three-year results. Operative dentistry 28:482‐487

Barber AJ, King PA (2014) Management of the single discoloured tooth part 1: aetiology, prevention and minimally invasive restorative options. Dental Update 41:98–110

Barbosa C de B (2017) Sensibilidade dentária e caracterização do biofilme dentário e sua relação com a atividade de cárie em crianças portadoras de Hipomineralização Molar Incisivo

Barbosa De Paulo L.F., Rosa R.R., Neto S.S., et al (2013) Family heritage of dentin dysplasia type I: Case series. Gazzetta Medica Italiana Archivio per le Scienze Mediche 172:411–414

Barcroft B.D., Childers K.R., Harris E.F. (1990) Effects of acidulated and neutral NaF solutions on bond strengths. Pediatric dentistry 12:180–182

Bari A., Sadaqat N., Mehreen S., et al (2018) Autoimmune polyglandular syndrome type-1. Pakistan Paediatric Journal 42:213–216

Baron C, Houchmand-Cuny M, Enkel B, Lopez-Cazaux S (2018) Prevalence of dental anomalies in French orthodontic patients: A retrospective study. Archives de Pédiatrie 25:426–430

Baron C., Lopez-Cazaux S., Houchmand-Cuny M., Enkel B. (2018) Prevalence of dental anomalies in French orthodontic patients: A retrospective study. Archives de Pediatrie 25:426–430. <https://doi.org/10.1016/j.arcped.2018.07.002>

Baroni C, Mazzoni A, Breschi L (2019) Molar incisor hypomineralization: supplementary, restorative, orthodontic, and esthetic long-term treatment. Quintessence international (Berlin, Germany : 1985) 50:412–417. <https://doi.org/10.3290/j.qi.a42327>

Baroni C., Rimondini L. (1992) Space maintenance and endodontic follow-up: case reports. The Journal of clinical pediatric dentistry 16:94–97

Barreto GJ (2018) Hipomineralização molar-incisivo: uma análise sobre a gravidade das lesões

Barron M.J., McDonnell S.T., Mackie I., Dixon M.J. (2008) Hereditary dentine disorders: dentinogenesis imperfecta and dentine dysplasia. Orphanet journal of rare diseases 3:31. <https://doi.org/10.1186/1750-1172-3-31>

Barros R, Oliveira R, Novaes A, et al (2005) Treatment of Class II furcation defects with guided tissue regeneration or enamel matrix derivative proteins - a 12-month comparative clinical study. PERIO: periodontal practice today 2:275‐284

Barrutia-Borque A., Agesta-Sanchez N., Gonzalez-Hermosa M.R., et al (2017) Tooth enamel hypoplasia in PHACE syndrome. A Case Report. European Journal of Pediatric Dermatology 27:175

Bartlett F.F., Rapp J.H., Goldstone J. (1987) Recurrent carotid stenosis: Operative strategy and late results. Journal of Vascular Surgery 5:452–456. <https://doi.org/10.1067/mva.1987.avs0050452>

Bartoli A., Bossu A., Sfasciotti G., Polimeni A. (2006) Glycogen Storage Disease type Ib: a paediatric case report. European journal of paediatric dentistry : official journal of European Academy of Paediatric Dentistry 7:192–198

Bartolo A., Camilleri A., Camilleri S. (2010) Unerupted incisors - characteristic features and associated anomalies. European Journal of Orthodontics 32:297–301. <https://doi.org/10.1093/ejo/cjp094>

Basalamah M., Baroudi K. (2016) Prevalence des anomalies bucco-dentaires chez des ecoliers de la ville de Sanaa (Yemen)Prevalence of oro-dental anomalies among schoolchildren in Sana’a city, Yemen. Eastern Mediterranean Health Journal 22:34–39. <https://doi.org/10.26719/2016.2.1.34>

Basaran G., Hamamci N., Ozer T. (2007) Cervical vertebral and dental maturity in Turkish subjects. American Journal of Orthodontics and Dentofacial Orthopedics 131:447. <https://doi.org/10.1016/j.ajodo.2006.08.016>

Başaran G, Ozer T, Devecioğlu Kama J (2009) Comparison of a recently developed nanofiller self-etching primer adhesive with other self-etching primers and conventional acid etching. European journal of orthodontics 31:271‐275. <https://doi.org/10.1093/ejo/cjn103>

Basch Y (2016) [Molar Incisor Hypomineralisation (MIH): A literature review and case report]. Refu’at ha-peh veha-shinayim (1993) 33:16–26

Bassigny F (1990) [Orthodontic effects of tooth injury to the permanent and temporary incisors of children and the adolescent [corrected]. Revue d’odonto-stomatologie 19:511–538

Bassigny F. (1990) Les repercussions orthodontiques des traumatismes dentaires sur les incisives permanentes et temporaires chez l’enfant et l’adolescent [corrected]Orthodontic effects of tooth injury to the permanent and temporary incisors of children and the adolescent [corrected. Revue d’odonto-stomatologie 19:511–538

Basso AP, Ruschel HC, Gatterman A, Ardenghi TM (2007) Hipomineralização molar-incisivo. Rev odonto ciênc 371–376

Basu D, Navneet AK, Dasgupta S, Bhattacharya S (2004) Cdc2-cyclin B-induced G2 to M transition in perch oocyte is dependent on Cdc25. Biology of reproduction 71:894–900

Bates E.A. (2013) A potential molecular target for morphological defects of fetal alcohol syndrome: Kir2.1. Current Opinion in Genetics and Development 23:324–329. <https://doi.org/10.1016/j.gde.2013.05.001>

BAVISHA KA (2020) Failing Implant Restoration. Odell’s Clinical Problem Solving in Dentistry E-Book 427

Baygin O, Cakır M, Ucuncu N (2017) Oral and dental health in children with chronic liver disease in the Turkey Northeast. Nigerian journal of clinical practice 20:1182–1188. <https://doi.org/10.4103/1119-3077.183259>

Bds MJH Molar hypomineralization

Beauchesne P., Trombley T., Agarwal S.C., et al (2019) Timing is everything: Implementing a Life Course Perspective to Investigate Developmental Origins of Health and Disease in a Medieval Italian Skeletal Sample. American Journal of Physical Anthropology 168:14. <https://doi.org/10.1002/ajpa.23802>

Becam G., Chevalier T. (2018) Neandertal features of the deciduous and permanent teeth from Portel-Ouest Cave (Ariege, France). American Journal of Physical Anthropology 168:45–69. <https://doi.org/10.1002/ajpa.23719>

Bechor N, Finkelstein T, Shapira Y, Shpack N (2014) Conservative orthodontic treatment for skeletal open bite associated with amelogenesis imperfecta. Journal of dentistry for children (Chicago, Ill) 81:96–102

Beck J. (2013) Dental evidence of changes in female social status during the Middle to Late Woodland transition. American Journal of Physical Anthropology 150:76–77. <https://doi.org/10.1002/ajpa.22247>

Bedi R (1989) The management of children with amelogenesis imperfecta. Restorative dentistry 5:28

Beeley J, Yip H, Stevenson A (2000) Chemochemical caries removal: a review of the techniques and latest developments. British dental journal 188:427–430

Bekes K (2017) Molaren-Inzisiven-Hypomineralisation–Prävalenz, Ätiologie und Therapie. ZWR-Das Deutsche Zahnärzteblatt 126:32–36

Bekes K, Mitulović G, Meißner N, et al (2020) Saliva proteomic patterns in patients with molar incisor hypomineralization. Scientific reports 10:7560. <https://doi.org/10.1038/s41598-020-64614-z>

Bekes K, Steffen R (2016) Das Würzburger MIH-Konzept: Teil 1. Der MIH-Treatment Need Index (MIH-TNI)

Belcheva AB, Philipov IA, Tomov GT (2016) Scanning Еlectron Мicroscopy of Еnamel and Dentin of Тeeth with Hypocalcified Аmelogenesis Imperfecta. Folia medica 58:54–59. <https://doi.org/10.1515/folmed-2016-0008>

Belloli EA, Beckford R, Hadley R, Flaherty KR (2016) Idiopathic non-specific interstitial pneumonia. Respirology (Carlton, Vic) 21:259–268. <https://doi.org/10.1111/resp.12674>

Beltran-Aguilar E.D., Barker L.K., Canto M.T., et al (2005) Surveillance for dental caries, dental sealants, tooth retention, edentulism, and enamel fluorosis--United States, 1988-1994 and 1999-2002. MMWR Surveillance summaries : Morbidity and mortality weekly report Surveillance summaries / CDC 54:1–43

BENÁ BT, KOGA IYA, LONG SM, et al (2016) Hipomineralização Molar-Incisivo (HMI): Relato de Caso. Odonto 24:23–24

Benbachir N., Ardu S., Krejci I. (2007) Indications and limits of the microabrasion technique. Quintessence International 38:811–815

Benbachir-Hassani N, Ardu S, Krejci I (2007) Indications and limits of the microabrasion technique. Quintessence International 38:811–5

Ben-Bassat Y., Brin I., Zilberman Y. (1989) Effects of trauma to the primary incisors on their permanent successors: multidisciplinary treatment. ASDC journal of dentistry for children 56:112–116

Benitez-del-Castillo JM, Martinez-de-la-Casa JM, Pato-Cour E, et al (2005) Long-term treatment of refractory posterior uveitis with anti-TNFalpha (infliximab). Eye (London, England) 19:841–845

Benly P. (2014) Effect of fluorosis on enamel. Research Journal of Pharmacy and Technology 7:362–364

Bensi C, Costacurta M, Docimo R (2020) Oral health in children with cerebral palsy: A systematic review and meta‐analysis. Special Care in Dentistry 40:401–411

Berdouses ED, Oulis CJ, Michalaki M, et al (2019) Histological validation of the automated caries detection system (ACDS) in classifying occlusal caries with the ICDAS II system in vitro. European archives of paediatric dentistry : official journal of the European Academy of Paediatric Dentistry 20:249–255. <https://doi.org/10.1007/s40368-018-0389-x>

Beretta M Dental caries prevention: a review on the use of dental sealants

Beretta M, Federici Canova F, Moscati M, et al (2020) State-of-the-art on MIH. Part. 2 MIH clinical management using ozone. European journal of paediatric dentistry 21:163–166. <https://doi.org/10.23804/ejpd.2020.21.02.13>

Berg RA, Simon JC, Fried D, Darling CL (2017) Optical changes of dentin in the near-IR as a function of mineral content. Proceedings of SPIE--the International Society for Optical Engineering 10044:. <https://doi.org/10.1117/12.2256745>

Berger SJ (1974) Composite technic for amelogenesis imperfecta. Dental survey 50:52–53

Bergus GR, Levy BT, Levy SM, et al (1996) Antibiotic use during the first 200 days of life. Archives of family medicine 5:523–526

Berk N, Başaran G, Özer T (2008) Comparison of sandblasting, laser irradiation, and conventional acid etching for orthodontic bonding of molar tubes. The European Journal of Orthodontics 30:183–189

Berkowitz RJ, Neuman P, Spalding P, et al (1989) Developmental orofacial deficits associated with multimodal cancer therapy: case report. Pediatric dentistry 11:227–231

Berlucchi I, Francetti L, Del Fabbro M, et al (2005) The influence of anatomical features on the outcome of gingival recessions treated with coronally advanced flap and enamel matrix derivative: a 1-year prospective study. Journal of periodontology 76:899‐907

Berman L.H. (1982) Intrinsic staining and hypoplastic enamel: etiology and treatment alternatives. General dentistry 30:484–488

Betsinger T.K., Scott A.B. (2012) From popular culture to scientific inquiry: A bioarchaeological analysis of vampires in post-medieval Poland. American Journal of Physical Anthropology 147:99. <https://doi.org/10.1002/ajpa.22033>

Betz B.J., O’hara M.C., Oldershaw L. (2019) Assessing striae of Retzius periodicity nondestructively using perikymata counts and distribution in two new populations. American Journal of Physical Anthropology 168:20. <https://doi.org/10.1002/ajpa.23802>

Bharadwaj M., Dubey A.P., Kapoor S. (2019) Effect of gluten-free diet on nutrition of newly diagnosed children with celiac disease. Gut 68:. <https://doi.org/10.1136/gutjnl-2019-IDDFabstracts.115>

Bhaskar S.A., Hegde S. (2014) Molar-incisor hypomineralization: prevalence, severity and clinical characteristics in 8- to 13-year-old children of Udaipur, India. Journal of the Indian Society of Pedodontics and Preventive Dentistry 32:322–329. <https://doi.org/10.4103/0970-4388.140960>

Bhaskar SA, Hegde S (2012) Complications of untreated molar-incisor hypomineralization in a 12-year-old boy. Clinics and practice 2:e88. <https://doi.org/10.4081/cp.2012.e88>

Bhat M, Nelson KB, Swango PA (1989) Lack of stability in enamel defects in primary teeth of children with cerebral palsy or mental retardation. Pediatric dentistry 11:118–120

Bhatavadekar N.B., Paquette D.W. (2008) Long-term follow-up and tomographic assessment of an intrabony defect treated with enamel matrix derivative. Journal of Periodontology 79:1802–1808. <https://doi.org/10.1902/jop.2008.070636>

Bhatia SK, Goyal A, Dubey M, et al (2012) Congenital Rubella Syndrome: dental manifestations and management in a 5 year old child. The Journal of clinical pediatric dentistry 37:71–75

Bhatia SK, Hunter ML, Ashley PF (2015) Amelogenesis Imperfecta with Coronal Resorption: Report of Three Cases. Dental update 42:945

Bhattacharya S., Basu D., Ak N., Priyadarshini A. (2007) Molecular mechanism of oocyte maturation. Society of Reproduction and Fertility supplement 63:45–55

Bhattacharyya S, Sen U, Bhattacharyya SP, Mukherjee D (2002) High performance liquid chromatographic separation of steroids from ovarian follicles of fresh water perch Anabas testudineus: identification and characterization of the maturation-inducing hormone. The Journal of experimental zoology 292:565–572

Bhatti H, Girdhar A, Usman F, et al (2013) Approach to acute exacerbation of idiopathic pulmonary fibrosis. Annals of thoracic medicine 8:71–77. <https://doi.org/10.4103/1817-1737.109815>

Bhushan BA, Garg S, Sharma D, Jain M (2008) Esthetic and endosurgical management of Turner’s hypoplasia; a sequlae of trauma to developing tooth germ. Journal of the Indian Society of Pedodontics and Preventive Dentistry 26:

Bhutani V.K., Johnson L.H., Donn S.M. (2005) Risk management of severe neonatal hyperbilirubinemia to prevent kernicterus. Clinics in Perinatology 32:125–139. <https://doi.org/10.1016/j.clp.2004.11.002>

Bhutda G, Deo V (2013) Five years clinical results following treatment of human intra-bony defects with an enamel matrix derivative: a randomized controlled trial. Acta odontologica scandinavica 71:764‐770. <https://doi.org/10.3109/00016357.2012.728245>

Bica C, Ion V, Mártha K, et al (2017) The Evaluation of Caries Severity Index and Dental Hypoplasia in Children with Acute Lymphoblastic Leukemia. Results from a Romanian Medical Center. Journal of Interdisciplinary Medicine 2:31–35

Bigeard L, Hemmerle J, Sommermater JI (1996) Clinical and ultrastructural study of the natal tooth: enamel and dentin assessments. ASDC journal of dentistry for children 63:23–31

Biggemann J, Hoffmann P, Hristov I, et al (2020) Injection Molding of 3-3 Hydroxyapatite Composites. Materials (Basel, Switzerland) 13:. <https://doi.org/10.3390/ma13081907>

BİLGİN EŞ, ERDEM AP GELİŞİMSEL MİNE DEFEKTLERİ VE TEDAVİ YAKLAŞIMLARI. Atatürk Üniversitesi Diş Hekimliği Fakültesi Dergisi 26:

Billings R.J., Berkowitz R.J., Watson G. (2004) Teeth. Pediatrics 113:1120–1127

Bimler HP (1986) Bimler therapy. Part 3. Case report. Journal of clinical orthodontics : JCO 20:190–193

Bimstein E., Magliocca K., Cohen D., et al (2011) Hyperbilirubinemic stain: Location and extent in dental tissues. Journal of Clinical Pediatric Dentistry 36:75–78. <https://doi.org/10.17796/jcpd.36.1.8646325256141166>

Binois A, Bridault A, Pion G, Ducrocq T (2014) Dental development pathology in wild artiodactyls: Two prehistoric case studies from France. International journal of paleopathology 4:53–58. <https://doi.org/10.1016/j.ijpp.2013.11.002>

Biondi AM, Cortese SG, Ortolani AM, et al (2013) Prevalencia de hipomineralización molar incisiva en niños con y sin demanda de atención. Rev Asoc Odontol Argent 101:139–145

Biria M., Kamareh S., Torabzadeh H., et al (2019) Evaluation of topical pre-fluoride therapy effects on marginal microleakage of composite restorations in deciduous teeth: An in vitro study. Journal of Islamic Dental Association of Iran 31:109–116. <https://doi.org/10.30699/jidai.31.2.109>

Biskupiak J., Sainski A., Yoo M., et al (2017a) Utilization of an algorithm to identify individuals at risk for hypophosphatasia (HPP) within an electronic health record (EHR) database. Journal of Bone and Mineral Research 31:. <https://doi.org/10.1002/jbmr.3107>

Biskupiak J., Tak C., Brixner D., et al (2017b) Predictors of fracture risk in patients with probable hypophosphatasia identified within an electronic health record database. Endocrine Reviews 38:

Bisphenol A Why are white spots on teeth more common?

Bizenjima T, Osuka Y, Tomita S, Saito A (2019) Periodontal Regenerative Therapy with Enamel Matrix Derivative in Patient with Chronic Periodontitis: A 3.5-year Follow-up Report. The Bulletin of Tokyo Dental College 60:131–138. <https://doi.org/10.2209/tdcpublication.2018-0048>

Bjanid O., Roszkowska-Bjanid D., Paszyna-Grzeskowiak M., et al (2017) Rare case of nephrocalcinosis in a 14-year-old girl: Questions. Pediatric Nephrology 32:607–608. <https://doi.org/10.1007/s00467-016-3434-1>

Blanck-Lubarsch M, Dirksen D, Feldmann R, et al (2019) Tooth Malformations, DMFT Index, Speech Impairment and Oral Habits in Patients with Fetal Alcohol Syndrome. International journal of environmental research and public health 16:. <https://doi.org/10.3390/ijerph16224401>

Blezinski B., Richardson A., Lee L.M.J. (2017) To model or not to model embryonic heart development, that is the educational question. FASEB Journal 31:

Bloch-Zupan A, Goodman JR (2006) Otodental syndrome. Orphanet journal of rare diseases 1:5

Bloch-Zupan A, Rousseaux M, Laugel V, et al (2013) A possible cranio-oro-facial phenotype in Cockayne syndrome. Orphanet journal of rare diseases 8:9. <https://doi.org/10.1186/1750-1172-8-9>

Bloch-Zupan A., Stachtou J., Emmanouil D., et al (2007) Oro-dental features as useful diagnostic tool in rubinstein-taybi syndrome. American Journal of Medical Genetics, Part A 143:570–573. <https://doi.org/10.1002/ajmg.a.31622>

Block MS (2010) Use of living cell construct to enhance bone reconstruction: preliminary results. Journal of oral and maxillofacial surgery : official journal of the American Association of Oral and Maxillofacial Surgeons 68:2914–2919. <https://doi.org/10.1016/j.joms.2010.05.066>

Blumhof S, Danila M, Sun D, et al (2019) Survival of patients with acute exacerbations of rheumatoid arthritis associated interstitial lung disease. American journal of respiratory and critical care medicine 199:

Boaventura BS (2017) Relato de caso de diferentes técnicas restauradoras para tratamento da hipomineralização molar-incisivo (MIH) em gêmeas monozigóticas

Bocaege E, Hillson S (2016) Disturbances and noise: Defining furrow-form enamel hypoplasia. American journal of physical anthropology 161:744–751. <https://doi.org/10.1002/ajpa.23070>

Bocaege E, Humphrey LT, Hillson S (2010) Technical note: a new three-dimensional technique for high resolution quantitative recording of perikymata. American journal of physical anthropology 141:498–503. <https://doi.org/10.1002/ajpa.21233>

Bodrumlu EH, Demiriz L, Toprak S (2018) Relationship between Severe Early Childhood Caries and dental development. European journal of paediatric dentistry 19:156–160

Bogle G, Garrett S, Stoller NH, et al (1997) Periodontal regeneration in naturally occurring Class II furcation defects in beagle dogs after guided tissue regeneration with bioabsorbable barriers. Journal of periodontology 68:536–544

Bogosavljević A, Misina V, Jordacević J, et al (2016) Treatment of teeth in the esthetic zone in a patient with amelogenesis imperfecta using composite veneers and the clear matrix technique: A case report. Vojnosanitetski pregled 73:288–292

Bogusiak K, Arkuszewski P, Skorek-Stachnik K, Kozakiewicz M (2014) Treatment strategy in Goldenhar syndrome. The Journal of craniofacial surgery 25:177–183. <https://doi.org/10.1097/SCS.0000000000000387>

Bohlen Delgado A.P., Rodrigues B., Moreira D., et al (2013) A new multimarkers strategy for risk stratification in patients with acute pulmonary thromboembolism. European Heart Journal: Acute Cardiovascular Care 2:159. <https://doi.org/10.1177/2048872613501458>

Boj J.R., Conde S., Plasencia P., et al (2019) Odontopediatria en la primera infanciaPediatric dentistry in early childhood. Pediatria Catalana 79:48–53

Bokan I, Bill J, Schlagenhauf U (2006) Primary flap closure combined with Emdogain alone or Emdogain and Cerasorb in the treatment of intra-bony defects. Journal of clinical periodontology 33:885‐893. <https://doi.org/10.1111/j.1600-051X.2006.01010.x>

Boksman L, Jordan RE (1983) Conservative treatment of the stained dentition: vital bleaching. Australian dental journal 28:67–72

Bolean M., Ciancaglini P., Simao A.M., et al (2011) Standardization of proteoliposome systems containing alkaline phosphatase and annexin V: Kinetic characterization at physiological pH. Journal of Bone and Mineral Research 26:

Bollu IP, Hari A, Thumu J, et al (2016) Comparative Evaluation of Microleakage Between Nano-Ionomer, Giomer and Resin Modified Glass Ionomer Cement in Class V Cavities- CLSM Study. Journal of clinical and diagnostic research : JCDR 10:ZC66. <https://doi.org/10.7860/JCDR/2016/18730.7798>

Bona A, Boscato N (2000) Clinical evaluation of allografts and homografts for restoration of missing tooth structure. Journal of prosthetic dentistry 84:163‐168. <https://doi.org/10.1067/mpr.2000.108575>

BÖNECKER M, HADDAD AE, DE CARVALHO P, ARIMA LY (2019) EMANUELLA PINHEIRO DASILVA OLIVEIRA. A Global Compendium of Oral Health: Tooth Eruption and Hard Dental Tissue Anomalies 62

Bonmassar A., Frati L., Fioretti M.C., et al (1979) Changes of the immunogenic properties of K36 lymphoma treated in vivo with 5(3,3-dimethyl-1-triazeno) imidazole-4-carboxamide (DTIC). European Journal of Cancer and Clinical Oncology 15:933–939

Bontsevich RA, Filinichenko TS, Vovk YR, et al (2019) Comparative assessment of physicians’ and senior medical students’ basic knowledge in treatment of chronic obstructive pulmonary disease

Bonucci E, Lozupone E, Silvestrini G, et al (1994) Morphological studies of hypomineralized enamel of rat pups on calcium-deficient diet, and of its changes after return to normal diet. The Anatomical record 239:379–395

Booij J, Livas C (2016) Unilateral Maxillary First Molar Extraction in Class II Subdivision: An Unconventional Treatment Alternative. Case Reports in Dentistry 2016:

Booij JW, Marie Kuijpers‐Jagtman A, Bronkhorst EM, et al (2020) Class II Division 1 malocclusion treatment with extraction of maxillary first molars: Evaluation of treatment and post‐treatment changes by the PAR Index. Orthodontics & Craniofacial Research

Booth JM, Mitropoulos CM, Worthington HV (1992) A comparison between the dental health of 3-year-old children living in fluoridated Huddersfield and non-fluoridated Dewsbury in 1989. Community dental health 9:151–157

Boran E., Balci S., Bardakci H. (2010) Post-axial hexodactyly and single atrium-indicating ellis-van creveld syndrome: Anesthetic management? Interactive Cardiovascular and Thoracic Surgery 10:. <https://doi.org/10.1510/icvts.2010.0000S1>

Borghetti A, Glise J, Monnet-Corti V, Dejou J (1999) Comparative clinical study of a bioabsorbable membrane and subepithelial connective tissue graft in the treatment of human gingival recession. Journal of periodontology 70:123‐130. <https://doi.org/10.1902/jop.1999.70.2.123>

Borghetti A, Novakovitch G, Louise F, et al (1993) Cryopreserved cancellous bone allograft in periodontal intraosseous defects. Journal of periodontology 64:

Borovskiĭ EV, Leont’ev VK, Maksimovskaia LN, Suntsov VG (1984) [Disordered mineralization process in hard dental tissues and the principles of its regulation]. Stomatologiia 63:19–22

Borsting T., Fagerhaug T.N., Stafne S.N., et al (2019) Associations between maternal vitamin D status in second and third trimester of pregnancy and offspring enamel hypomineralisation at 7-9 years: A longitudinal study. Norsk Epidemiologi 28:45

Borysewicz-Lewicka M. (2009) Aspekty stomatologiczne w praktyce lekarza rodzinnegoImportant dental aspects in family physician practice. Family Medicine and Primary Care Review 11:99–101

Bossens M. (2001) Antibiotiques et grossesseAntibiotics and pregnancy. Revue Medicale de Bruxelles 22:

Bossù M, Bartoli A, Orsini G, et al (2007) Enamel hypoplasia in coeliac children: a potential clinical marker of early diagnosis. European journal of paediatric dentistry 8:31–37

Boston DW, al-bargi H, Bogert M (1999) Abrasion, erosion, and abfraction combined with linear enamel hypoplasia: a case report. Quintessence international (Berlin, Germany : 1985) 30:683–687

Botelho FM (2019) Fatores etiológicos associados à hipomineralização molar incisivo: um estudo transversal

Boutboul R (1979) [Restorations of dysplasias and coronal fractures of enamel with the Nuva method (Caulk)]. Pedodontie Francaise 13:133–144

Boutboul R. (1979) Restauration des dysplasies et fractures coronaires de l’email par la methode Nuva (Caulk)Restorations of dysplasias and coronal fractures of enamel with the Nuva method (Caulk). Pedodontie Francaise 13:133–144

Bouvier D, Duprez JP, Bois D (1996) Rehabilitation of young patients with amelogenesis imperfecta: a report of two cases. ASDC journal of dentistry for children 63:443–447

Bowden S.A., Adler B.H. (2018) Asfotase alfa treatment for 1 year in a 16 year-old male with severe childhood hypophosphatasia. Osteoporosis International 29:511–515. <https://doi.org/10.1007/s00198-017-4267-x>

Bowen J.A., Mellonig J.T., Gray J.L., Towle H.T. (1989) Comparison of decalcified freeze-dried bone allograft and porous particulate hydroxyapatite in human periodontal osseous defects. Journal of periodontology 60:647–654. <https://doi.org/10.1902/jop.1989.60.12.647>

Bowen RL, George LA, Eichmiller FC, Misra DN (1993) An esthetic glass-ceramic for use in composite restoration inserts. Dental materials : official publication of the Academy of Dental Materials 9:290–294

Bowen WH (2002) Fluorosis: is it really a problem? Journal of the American Dental Association (1939) 133:1405–1407

Boy S, Crossley D, Steenkamp G (2016) Developmental Structural Tooth Defects in Dogs - Experience From Veterinary Dental Referral Practice and Review of the Literature. Frontiers in veterinary science 3:9. <https://doi.org/10.3389/fvets.2016.00009>

Boyde A (1972) Influence of normal and abnormal enamel structure on cavity margins. British dental journal 133:421–427

Boyne P.J. (1974) Osseous grafts and implants in the restoration of large oral defects. Journal of Periodontology 45:378–384

Bozal CB, Kaplan A, Ortolani A, et al (2015) Ultrastructure of the surface of dental enamel with molar incisor hypomineralization (MIH) with and without acid etching. Acta odontologica latinoamericana : AOL 28:192–198. <https://doi.org/10.1590/S1852-48342015000200016>

Bozorgmehr E, Malek Mohammadi T, Hajizamani A, et al (2012) Knowledge, attitude, and practices of pediatricians about children’s oral health. Journal of Oral Health and Oral Epidemiology 1:93–98

Bozzuto L., Mastroyannis S.A., Salva C., Haggerty A. (2018) Enhanced recovery pathway for minimally invasive hysterectomy: Outcomes of a quality improvement initiative. Obstetrics and Gynecology 131:

Bradley J.S. (2014) Which antibiotic for resistant Gram-positives, and why? Journal of Infection 68:. <https://doi.org/10.1016/j.jinf.2013.09.016>

Brancher A Aluhê Lopes Fatturi, Bruna Leticia Menoncin, Magdalena Torres Reyes, Michelle Meger, Rafaela Scariot, João

Brandsten C., Alatli I., Wurtz T., Hammarstrom L. (1995) Coronal dentinal nodules induced by single or multiple injections of HEBP in young rats. Connective tissue research 32:275–279

Brannstrom M, Lindskog S, Nordenvall KJ (1984) Enamel hypoplasia in permanent teeth induced by periodontal ligament anesthesia of primary teeth. Journal of the American Dental Association (1939) 109:735–736

Brennan MT, O’Connell BC, Rams TE, O’Connell AC (1999) Management of gingival overgrowth associated with generalized enamel defects in a child. The Journal of clinical pediatric dentistry 23:97–101

Brignall I, Mehta SB, Banerji S, Millar BJ (2011) Aesthetic composite veneers for an adult patient with amelogenesis imperfecta: a case report. Dental update 38:594

Brignardello-Petersen R (2017) Molar–incisor hypomineralization seems to be associated with caries experience, but the magnitude of this association is yet to be reliably determined. The Journal of the American Dental Association 148:e197

Briner WW, Rosen S (1967) Effect of fluoride on hypomineralized areas in the molars of rats fed a cariogenic diet. Archives of oral biology 12:1077–1084

Broadbent JM, Thomson WM, Williams SM (2005) Does caries in primary teeth predict enamel defects in permanent teeth? A longitudinal study. Journal of dental research 84:260–264

Brogardh-Roth S. (2010) The preterm child in dentistry. Behavioural aspects and oral health. Swedish dental journal Supplement 11–85

Brogårdh-Roth S, Matsson L, Klingberg G (2011) Molar-incisor hypomineralization and oral hygiene in 10- to-12-yr-old Swedish children born preterm. European journal of oral sciences 119:33–39. <https://doi.org/10.1111/j.1600-0722.2011.00792.x>

Bromley KM, Lakshminarayanan R, Thompson M, et al (2012) Amelogenin processing by MMP-20 prevents protein occlusion inside calcite crystals. Crystal growth & design 12:4897–4905

Bronckers A.L. (2017) Ion Transport by Ameloblasts during Amelogenesis. Journal of dental research 96:243–253. <https://doi.org/10.1177/0022034516681768>

Bronckers ALJJ, Jalali R, Lytton J (2017) Reduced Protein Expression of the Na+/Ca2++K+-Exchanger (SLC24A4) in Apical Plasma Membranes of Maturation Ameloblasts of Fluorotic Mice. Calcified tissue international 100:80–86. <https://doi.org/10.1007/s00223-016-0197-4>

Bronckers ALJJ, Lyaruu D, Jalali R, et al (2015) Ameloblast Modulation and Transport of Cl^−^, Na^+^, and K^+^ during Amelogenesis. Journal of dental research 94:1740–1747. <https://doi.org/10.1177/0022034515606900>

Bronckers ALJJ, Lyaruu DM, DenBesten PK (2009) The impact of fluoride on ameloblasts and the mechanisms of enamel fluorosis. Journal of dental research 88:877–893. <https://doi.org/10.1177/0022034509343280>

Brook A (2009) Multilevel complex interactions between genetic, epigenetic and environmental factors in the aetiology of anomalies of dental development. Archives of oral biology 54:S3–S17

Brook J (2019) Top 10 most viewed BDJ articles of 2018.

Brookes SJ, Barron MJ, Boot-Handford R, et al (2014) Endoplasmic reticulum stress in amelogenesis imperfecta and phenotypic rescue using 4-phenylbutyrate. Human molecular genetics 23:2468–2480. <https://doi.org/10.1093/hmg/ddt642>

Brooks JK, Bare LC, Davidson J, et al (2008) Junctional epidermolysis bullosa associated with hypoplastic enamel and pervasive failure of tooth eruption: Oral rehabilitation with use of an overdenture. Oral surgery, oral medicine, oral pathology, oral radiology, and endodontics 105:e24. <https://doi.org/10.1016/j.tripleo.2007.12.038>

Bröseler F, Tietmann C, Hinz A-K, Jepsen S (2017) Long-term results of periodontal regenerative therapy: A retrospective practice-based cohort study. Journal of clinical periodontology 44:520–529. <https://doi.org/10.1111/jcpe.12723>

Browne D, Whelton H, O’Mullane D, et al (2011) The aesthetic impact of enamel fluorosis on Irish adolescents. Community dentistry and oral epidemiology 39:127–136. <https://doi.org/10.1111/j.1600-0528.2010.00577.x>

Browning W, Cho S, Deschepper E (2012) Effect of a nano-hydroxyapatite paste on bleaching-related tooth sensitivity. Journal of esthetic and restorative dentistry 24:268‐276. <https://doi.org/10.1111/j.1708-8240.2011.00437.x>

Bruhn T.O., Taplin J.H., Jackson I.M.D. (1991) Hypothyroidism reduces content and increases in vitro release of pro-thyrotropin-releasing hormone peptides from the median eminence. Neuroendocrinology 53:511–515

Bruserud O., Oftedal B.E., Bratland E., et al (2016) A longitudinal follow-up of autoimmune polyendocrine syndrome type 1. Journal of Clinical Endocrinology and Metabolism 101:2975–2983. <https://doi.org/10.1210/jc.2016-1821>

Brusevold IJ, Bie TMG, Baumgartner CS, et al (2017) Molar incisor malformation in six cases: description and diagnostic protocol. Oral surgery, oral medicine, oral pathology and oral radiology 124:52–61

Bruzda-Zwiech A, Filipińska R, Borowska-Strugińska B, et al (2015) Caries experience and distribution by tooth surfaces in primary molars in the pre-school child population of Lodz, Poland. Oral Health Prev Dent 13:557–66

Bučević P (2014) Molarno incizivna hipomineralizacija:: etiologija, dijagnostika i terapija

Büchel K, Gerwig P, Weber C, et al (2011) Prevalence of enamel fluorosis in 12-year-olds in two Swiss cantons. Schweizer Monatsschrift fur Zahnmedizin = Revue mensuelle suisse d’odonto-stomatologie = Rivista mensile svizzera di odontologia e stomatologia 121:647–656

Buchet R., Magne D., Millan J.L. (2013) Multisystemic functions of alkaline phosphatases. 1053:27–51. <https://doi.org/10.1007/978-1-62703-562-0_3>

Büchi D, Fehmer V, Sailer I, et al (2014) Minimally invasive rehabilitation of a patient with amelogenesis imperfecta. The international journal of esthetic dentistry 9:134–145

Bulani M, Shetiya SH, Agarwal D, Mathur A (2020) Severe Early Childhood Caries, Hypoplasia-Associated Severe Early Childhood Caries and Deciduous Molar Hypomineralization amongst 3 to 6 Years Old Anganwadi Children in Pune, Maharashtra: A Cross-Sectional Study. Indian Journal of Public Health Research & Development 11:174–179

Buldur B, Güvendi ON (2020) Conceptual modelling of the factors affecting oral health‐related quality of life in children: A path analysis. International Journal of Paediatric Dentistry 30:181–192

Bulus D., Tayfur A.C., Yilmaz D. (2018) Carbonic anhydrase deficiency: Three siblings. Hormone Research in Paediatrics 90:189–190. <https://doi.org/10.1159/000492307>

Buonfrate D, Vazquez-Villegas J, Munoz J, et al (2018) Strong treat 1 to 4: a randomized, open-label clinical trial on multiple versus single dose of ivermectin for the treatment of strongyloidiasis. American journal of tropical medicine and hygiene 99:399‐

Burke A., Boyce A., Collins M. (2017) Dental findings from the national institutes of health fibrous dysplasia/mccune-albright syndrome cohort. Journal of Bone and Mineral Research 31:. <https://doi.org/10.1002/jbmr.3107>

Burns J, Hollands K (2015) Association between developmental defects of enamel and dental caries. Evidence-based dentistry 16:72–73

Busch M., Sooreide E. (2009) Good outcome in octogenarians after ventricular fibrillation out-of-hospital cardiac arrest. Critical Care 13:. <https://doi.org/10.1186/cc7230>

Busch M., Soreide E. (2009) Mild induced hypothermia in in-hospital cardiac arrest survivors - It is worthwhile? Acta Anaesthesiologica Scandinavica, Supplement 53:52. <https://doi.org/10.1111/j.1399-6576.2009.02004.x>

Bussadori SK, Roth F, Guedes CC, et al (2006) Bleaching non vital primary teeth: case report. The Journal of clinical pediatric dentistry 30:179–182

Bussell RM, Deery C (2010) Case report: Blue chromogenic dental staining in child with West syndrome. European archives of paediatric dentistry : official journal of the European Academy of Paediatric Dentistry 11:298–300

Butler I.J., Gadoth N. (1976) Kearns Sayre syndrome. A review of a multisystem disorder of children and young adults. Archives of Internal Medicine 136:1290–1293. <https://doi.org/10.1001/archinte.136.11.1290>

Büttner W (1979) [Sealing]. Zahnarztliche Mitteilungen 69:1274

Buttner W. (1979) Die VersiegelungSealing. Zahnarztliche Mitteilungen 69:1274

Bystrom E.B., Sanger R.G., Stewart R. (1975) The syndrome of ectrodactyly, ectodermal dysplasia, and clefting (EEC). Journal of Oral Surgery 33:192–198

CAbASS C, MARIe-COUSI A, Huet A, Sixou J (2015) Computer-assisted intraosseous anaesthesia for molar and incisor hypomineralisation teeth. Odonto-Stomatologie Tropicale

Cabasse C., Marie-Cousin A., Huet A., Sixou J.L. (2015) Computer-assisted intraosseous anaesthesia for molar and incisor hypomineralisation teeth. A preliminary study. Odonto-stomatologie tropicale = Tropical dental journal 38:5–9

Cabay RJ (2014) An overview of molecular and genetic alterations in selected benign odontogenic disorders. Archives of pathology & laboratory medicine 138:754–758. <https://doi.org/10.5858/arpa.2013-0057-SA>

Cabral RN (2017) Novo sistema de detecção para a hipomineralização molar incisivo: diagnóstico, progressão dos defeitos e decisão de tratamento

Caffesse RG, Nasjleti CE, Morrison EC, Sanchez R (1994) Guided tissue regeneration: comparison of bioabsorbable and non-bioabsorbable membranes. Histologic and histometric study in dogs. Journal of periodontology 65:583–591

Cagetti MG, Angelino E (2020) Could SARS‐CoV‐2 burst the use of Non‐Invasive and Minimally Invasive treatments in paediatric dentistry? International journal of paediatric dentistry

Cagetti MG, Cattaneo S, Hu YQ, Campus G (2017) Amelogenesis Imperfecta: A Non-Invasive Approach to Improve Esthetics in Young Patients. Report of Two Cases. The Journal of clinical pediatric dentistry 41:332–335. <https://doi.org/10.17796/1053-4628-41.5.332>

Cairns AM (2011) Reality bites. Journal of Family Health Care 21:31

Cakir E, Aksoy F, Cakır FB, Ertem T (2010) Chronic eosinophilic pneumonia with mucous plugs in a child. Pediatric pulmonology 45:1040–1042. <https://doi.org/10.1002/ppul.21299>

Calabrese F, Giacometti C, Rea F, et al (2002) Recurrence of idiopathic pulmonary hemosiderosis in a young adult patient after bilateral single-lung transplantation. Transplantation 74:1643–1645

Calabrò A, Calabrò S, Busà A (1991) [Clinical evaluation of a hybrid composite]. Attualita dentale 7:14

Calamari L, Morera P, Bani P, et al (2018) Effect of hot season on blood parameters, fecal fermentative parameters, and occurrence of Clostridium tyrobutyricum spores in feces of lactating dairy cows. Journal of dairy science 101:4437–4447. <https://doi.org/10.3168/jds.2017-13693>

Calvano Küchler E, Maschietto Pucinelli C, Carpio Horta K, et al (2020) Dental Caries, Developmental Defects of Enamel and Enamel Microhardness Associated with Genetic Polymorphisms in the RANK/RANKL/OPG System. The Journal of clinical pediatric dentistry 44:35–40. <https://doi.org/10.17796/1053-4625-44.1.6>

Camargo P.M., Lekovic V., Weinlaender M., et al (2001) The effectiveness of enamel matrix proteins used in combination with bovine porous bone mineral in the treatment of intrabony defects in humans. Journal of clinical periodontology 28:1016–1022

Camesasca F.I., Vinciguerra P., Seiler T. (2011) Bilateral ring-shaped intrastromal opacities after corneal cross-linking for keratoconus. Journal of Refractive Surgery 27:913–915. <https://doi.org/10.3928/1081597X-20110812-02>

Campos A., Sanchez-Quevedo M.C., Garcia J.M., et al (2000) Mineralization of human premolar occlusal fissures. A quantitative histochemical microanalysis. Histology and Histopathology 15:499–502

Campos de Freitas A, Mussolino Ribeiro Z, Tambasco de Oliveira MC, Assed S (1986) [Clinical management of a case of epidermolysis bullosa]. Revista da Faculdade de Odontologia de Ribeirao Preto 23:71–78

Campos RE, Miranda Valdivia ADC, Santos-Filho PC de F, et al (2014) Conservative treatment for amelogenesis imperfecta: a case report. General dentistry 62:74–78

Campos-Lara P, Santos-Diaz MA, Ruiz-Rodríguez MS, et al (2012) Orofacial findings and dental management of Williams-Beuren syndrome. The Journal of clinical pediatric dentistry 36:401–404

Camps J., de Franceschi H., Idir F., et al (2007) Time-Course Diffusion of Hydrogen Peroxide Through Human Dentin: Clinical Significance for Young Tooth Internal Bleaching. Journal of Endodontics 33:455–459. <https://doi.org/10.1016/j.joen.2006.12.006>

Camps J, Pommel L, Aubut V, About I (2010) Influence of acid etching on hydrogen peroxide diffusion through human dentin. American journal of dentistry 23:168–170

Canger EM, Celenk P, Yenísey M, Odyakmaz SZ (2010) Amelogenesis imperfecta, hypoplastic type associated with some dental abnormalities: a case report. Brazilian dental journal 21:170–174

Cangini F, Cornelini R (2005) A comparison between enamel matrix derivative and a bioabsorbable membrane to enhance healing around transmucosal immediate post-extraction implants. Journal of periodontology 76:1785‐1792. <https://doi.org/10.1902/jop.2005.76.10.1785>

Cannon HM, Broffitt B, Levy SM, Warren JJ (2010) Longitudinal changes in parental satisfaction: mixed dentition esthetics. Journal of dentistry for children (Chicago, Ill) 77:166–173

Cannon M CLINICAL TECHNIQUE

Cantekin K, Arslan D, Delikan E (2015a) Presence and distribution of dental enamel defects, recurrent aphthous lesions and dental caries in children with celiac disease. Pakistan journal of medical sciences 31:606–609. <https://doi.org/10.12669/pjms.313.6960>

Cantekin K., Gumus H., Torun Y.A., Sahin H. (2015) The evaluation of developmental enamel defects and dental treatment conditions in a group of Turkish children with congenital heart disease. Cardiology in the Young 25:312–316. <https://doi.org/10.1017/S1047951113002308>

Cantekin K, Gumus H, Torun YA, Sahin H (2015b) The evaluation of developmental enamel defects and dental treatment conditions in a group of Turkish children with congenital heart disease. Cardiology in the Young 25:

Cao F-L, Liu M-G, Hao J, et al (2007) Different roles of spinal p38 and c-Jun N-terminal kinase pathways in bee venom-induced multiple pain-related behaviors. Neuroscience letters 427:50–54

Carbonell Pérez A, Duarte Solano M, Prada Correa M Conocimiento del diagnóstico y tratamiento para la HIM por los estudiantes de la clínica del niño en la Universidad Santo Tomás en el ii 2018

Cárdenas Flores A, Flores Reyes H, Gordillo Moscoso A, et al (2009) Clinical efficacy of 5% sodium hypochlorite for removal of stains caused by dental fluorosis. The Journal of clinical pediatric dentistry 33:187–191

Cardiel Rios S.A. (2005) Class II correction in a severe hyperdivergent growth pattern, bilateral open bite and oral compromise. Angle Orthodontist 75:870–880

Cares Henriquez A., Oxenham M.F. (2019) New distance-based exponential regression method and equations for estimating the chronology of linear enamel hypoplasia (LEH) defects on the anterior dentition. American Journal of Physical Anthropology 168:510–520. <https://doi.org/10.1002/ajpa.23764>

Carlos R., Contreras-Vidaurre E., Almeida O.P., et al (2008) Regional odontodysplasia: morphological, ultrastructural, and immunohistochemical features of the affected teeth, connective tissue, and odontogenic remnants. Journal of dentistry for children (Chicago, Ill) 75:144–150

Carlyle S.W., Parr R.L., Geoffrey Hayes M., O’Rourke D.H. (2000) Brief communication: The timing of linear hypoplasias on human anterior teeth. American Journal of Physical Anthropology 113:135–139

Carneiro DP, Reis LM, Gouvêa GR, et al (2020) Enamel development defects and oral symptoms: A hierarchical approach. Community Dental Health 37:1–6

Carneiro PA (1974) [Hypoplasias and enamel pigmentations treated with adhesive resins]. Revista odontologica 20:88–94

Carneiro P.A. (1974) Hipoplasias y pigmentaciones adamantinas tratadas por resinas adhesivasHypoplasias and enamel pigmentations treated with adhesive resins. Revista odontologica 20:88–94

Carraro J.J., Sznajder N., Alonso C.A. (1976) Intraoral cancellous bone autografts in the treatment of infrabony pockets. Journal of Clinical Periodontology 3:104–109. <https://doi.org/10.1111/j.1600-051X.1976.tb01856.x>

Carrasco Jaramillo VM (2016) Tratamientos que los odontólogos indican para los diferentes estadíos de la hipomineralización molar incisal en niños entre 6 a 12 años de edad

Carrasco Muñoz M, Toncio Salazar H, Valdés P (2018) Prevalencia de lesiones de caries vinculadas a defectos del desarrollo del esmalte tipo MIH/HSPM en dientes índice de MIH y HSPM, en pacientes de 6 a 12 años atendidos en el Centro de Clínicas Odontológicas, Universidad de Talca, 2018.

Carrillo CM, Corrêa FNP, Lopes NNF, et al (2014) Dental anomalies in children submitted to antineoplastic therapy. Clinics (Sao Paulo, Brazil) 69:433–437

Carrotte P. (2005) Endodontic treatment for children. British Dental Journal 198:9–15. <https://doi.org/10.1038/sj.bdj.4811946>

Carrotte P (2007) Do Your Children or Patients Chew Gum? Dental Update 34:640–640

Caruso F, Ciccarese F, Cesana G, et al (2017) Massive Incisional Hernia Repair with Parietex: Monocentric Analysis on 500 Cases Treated with a Laparoscopic Approach. Journal of laparoendoscopic & advanced surgical techniques Part A 27:388–392. <https://doi.org/10.1089/lap.2016.0623>

Carvalho JC (2014) Caries process on occlusal surfaces: evolving evidence and understanding. Caries research 48:339–346

Carvalho JC, Silva EF, Gomes RR, et al (2011) Impact of enamel defects on early caries development in preschool children. Caries research 45:353–360. <https://doi.org/10.1159/000329388>

Carvalho LD, Bernardon JK, Bruzi G, et al (2013) Hypoplastic enamel treatment in permanent anterior teeth of a child. Operative dentistry 38:363–368. <https://doi.org/10.2341/12-284-T>

Carvalho V., Jacomo D.R., Campos V. (2010) Frequency of intrusive luxation in deciduous teeth and its effects. Dental Traumatology 26:304–307. <https://doi.org/10.1111/j.1600-9657.2010.00893.x>

Carvalho VR (2019) Hipomineralização molar incisivo: correlação com fluorose e cárie dentária, impacto do não tratamento e influência na qualidade de vida

Casarin R, Del Peloso Ribeiro E, Nociti F, et al (2008) A double-blind randomized clinical evaluation of enamel matrix derivative proteins for the treatment of proximal class-II furcation involvements. Journal of clinical periodontology 35:429‐437. <https://doi.org/10.1111/j.1600-051X.2008.01202.x>

Casarin RCV, Ribeiro EDP, Ribeiro FV, et al (2009) Influence of anatomic features on the effectiveness of enamel matrix derivative proteins in the treatment of proximal Class II furcation involvements. Quintessence international (Berlin, Germany : 1985) 40:753–761

Casas Araya M Comparación de las caracteristicas dentales, faciales y esqueleticas presentes en individuos con hipomineralización inciso-molar (HIM) y Amelogenesis Imperfecta (AI): estudio exploratorio

Casati MZ, Sallum EA, Nociti FH, et al (2002) Enamel matrix derivative and bone healing after guided bone regeneration in dehiscence-type defects around implants. A histomorphometric study in dogs. Journal of periodontology 73:789–796

Casella E (1976) [Management of a case of amelogenesis imperfecta and open bite using a composite resin (Nuova System)]. Odontostomatologia E Implantoprotesi 2:20–21

Caspersen I, Warburg M (1968) Hallermann-Streiff syndrome. Acta ophthalmologica 46:385–390

Cassin A.M., Pearson G.J., Picton D.C.A. (1991) Fissure sealants as a means of prolonging longevity of amalgam restorations - An in-vitro feasibility study. Clinical Materials 7:203–207

Castaño Lopez LJ, Mejia Roldan JD, Escobar Rojas A (2015) Prevalencia y severidad de la hipomineralizacion molarincisivo (HMI) en pacientes escolarizados de la ciudad de Medellin.

Castellanos A, de la Rosa M, de la Garza M, Caffesse R (2006) Enamel matrix derivative and coronal flaps to cover marginal tissue recessions. Journal of periodontology 77:7‐14. <https://doi.org/10.1902/jop.2006.77.1.7>

Castellazzi L, Patria MF, Frati G, et al (2016) Idiopathic pulmonary haemosiderosis in paediatric patients: how to make an early diagnosis. Italian journal of pediatrics 42:86. <https://doi.org/10.1186/s13052-016-0296-x>

CASTELO BRANCO MBS (2020) CONHECIMENTO E CONDUTAS DOS CIRURGIÕES-DENTISTAS DA ESTRATÉGIA SAÚDE DA FAMÍLIA SOBRE A HIPOMINERALIZAÇÃO MOLAR-INCISIVO.

Castilla Pajares F del R Evaluación del manejo terapéutico en estudiantes de pregrado, egresados y docentes en piezas con hipomineralización incisivo-molar (HIM) en una Universidad privada de Lima

Castro I del RA (2014) HIPOMINERALIZACIÓN INCISO MOLAR (HIM). Revista Odontológica Basadrina 2:39–43

Castro K, Ferreira A, Duarte R, et al (2014) Acceptability, efficacy and safety of two treatment protocols for dental fluorosis: a randomized clinical trial. Journal of dentistry 42:938‐944. <https://doi.org/10.1016/j.jdent.2014.01.011>

Castro-Rebollo M, Montes-Mollón MA, Pérez-Rico C, Teus MA (2011) [Effectiveness of topical bevacizumab in bilateral primary lipid keratopathy]. Archivos de la Sociedad Espanola de Oftalmologia 86:374–376. <https://doi.org/10.1016/j.oftal.2011.05.029>

Catalá M, Bonafé N, García M, et al (2012) Hipomineralización en primeros molares permanentes: protocolos preventivo y restaurador. ODONTOL PEDIÁTR (Madrid) 20:123–133

Catalán Gamonal BP (2016) Prevalencia y distribución del grado de severidad de hipomineralización incisivo-molar en niños de 6 a 12 años de la Provincia de Santiago

Cattabriga M, Rotundo R, Muzzi L, et al (2001) Retrospective evaluation of the influence of the interleukin-1 genotype on radiographic bone levels in treated periodontal patients over 10 years. Journal of periodontology 72:767–773

Caufield PW, Li Y, Bromage TG (2012) Hypoplasia-associated severe early childhood caries--a proposed definition. Journal of dental research 91:544–550. <https://doi.org/10.1177/0022034512444929>

Cavalcanti Monteiro de Oliveira S, Campos Burigo R, Araujo de Lucena Lira G, et al (2020) Rubinstein-Taybi syndrome: A case report with 7-year follow-up. Revista de Odontopediatría Latinoamericana 10:102–110

Cavalheiro JP, Bussaneli DG, Restrepo M, et al (2017a) Aspectos clínicos de la fluorosis dental de acuerdo con las características histológicas: una revisión del Índice de Thylstrup Fejerskov. CES Odontología 30:41–50

Cavalheiro JP, Girotto Bussaneli D, Restrepo M, et al (2017b) Clinical aspects of dental fluorosis according to histological features: a Thylstrup Fejerskov Index review. CES Odontología 30:41–50

Cavalheiro JP, Souza MIAV, Duque CCO, et al (2020) Esthetic rehabilitation of anterior teeth with molar-incisor hypomineralization and dental fluorosis: a case report. General dentistry 68:34–39

Cazzolla AP, De Franco AR, Lacaita M, Lacarbonara V (2018) Efficacy of 4-year treatment of icon infiltration resin on postorthodontic white spot lesions. Case Reports 2018:

Cehajic E (2014) Molar-Incisiv-Hypomineralisering (MIH) i en gruppe 8-og 9-åringer i byen Kljuc, Bosnia-Hercegovina

Celik EU, Yazkan B, Yildiz G, Tunac AT (2017) Clinical performance of a combined approach for the esthetic management of fluorosed teeth: Three-year results. Nigerian journal of clinical practice 20:943–951. <https://doi.org/10.4103/1119-3077.180066>

Celik EU, Yildiz G, Yazkan B (2013a) clinical evaluation of enamel microabrasion for the aesthetic management of mild-to-severe dental fluorosis. Journal of esthetic and restorative dentistry : official publication of the American Academy of Esthetic Dentistry . [et al] 25:422–430. <https://doi.org/10.1111/jerd.12052>

Celik EU, Yıldız G, Yazkan B (2013b) Comparison of enamel microabrasion with a combined approach to the esthetic management of fluorosed teeth. Operative dentistry 38:E134. <https://doi.org/10.2341/12-317-C>

Cengiz E, Kurtulmus-Yilmaz S, Karakaya I, Aktore H (2018) Color difference of composite resins after cementation with different shades of resin luting cement. Odontology 106:181–186. <https://doi.org/10.1007/s10266-017-0311-8>

Ceppi E, Dall’Oca S, Rimondini L, et al (2006) Cementoenamel junction of deciduous teeth: SEM-morphology. European journal of paediatric dentistry 7:131–134

Cerri P.S., Pizzol-Junior J.P., Sasso-Cerri E., et al (2015) Immunolocalization of osteocalcin in matrix-vesicles in the early developing alveolar process of rat molars. Molecular Biology of the Cell 26:. <https://doi.org/10.1091/mbc.E15-09-0674>

Cetiner D., Uraz A., Cetiner S., et al (2019) Oral and dental alterations and growth disruption following chemotherapy in long-term survivors of childhood malignancies. Supportive Care in Cancer 27:1891–1899. <https://doi.org/10.1007/s00520-018-4454-0>

Ceyhan D, Kirzioglu Z, Emek T (2019) A long-term clinical study on individuals with amelogenesis imperfecta. Nigerian journal of clinical practice 22:1157–1162. <https://doi.org/10.4103/njcp.njcp_227_18>

Chacon L.F., Lopez M.L., Frechero N.M. (2009) Prevalencia de fluorosis dental y consumo de fluoruros ocultos en escolares del municipio de NezahualcoyotlPrevalence of dental fluorosis and consumption of hidden fluoride in school children in the municipality of Nezahualcoyotl. Gaceta medica de Mexico 145:263–267

Chadwick BL, White DA, Morris AJ, et al (2006) Non-carious tooth conditions in children in the UK, 2003. British dental journal 200:379–384

Chadwick J, Mills M, Mealey B (2016) Clinical and Radiographic Evaluation of Demineralized Freeze-Dried Bone Allograft Versus Platelet-Rich Fibrin for the Treatment of Periodontal Intrabony Defects in Humans. Journal of periodontology 87:1253‐1260. <https://doi.org/10.1902/jop.2016.160309>

Chafaie A (2004) Minimally invasive aesthetic treatment for discolored and fractured teeth in adolescents: a case report. Practical procedures & aesthetic dentistry : PPAD 16:319–324

Chafaie A (2016) Esthetic management of anterior dental anomalies: A clinical case. International orthodontics 14:357–365. <https://doi.org/10.1016/j.ortho.2016.07.005>

Chaini K, Georgopoulou MK (2016) General pulp calcification: Literature review and. ENDO (Lond Engl) 10:69–75

Chałas R, Jurczykowska M, Marczyński R, Pels E (2014) Composite inlays as a modern way of posterior restorations in the dental arch. Polish Journal of Public Health 124:99–102

Chalifoux PR (2004) Composite tints: mixing composite materials to alter color and techniques to simulate hypocalcification and craze lines. Compendium of continuing education in dentistry (Jamesburg, NJ : 1995) 25:583

Chalisserry EP, Nam SY, Park SH, Anil S (2017) Therapeutic potential of dental stem cells. Journal of tissue engineering 8:2041731417702531. <https://doi.org/10.1177/2041731417702531>

Chambrone D, Pasin I, Chambrone L, et al (2010) Treatment of infrabony defects with or without enamel matrix proteins: a 24-month follow-up randomized pilot study. Quintessence international (berlin, germany : 1985) 41:125‐134

Chambrone D, Pasin I, Conde M, et al (2007) Effect of enamel matrix proteins on the treatment of intrabony defects: a split-mouth randomized controlled trial study. Brazilian oral research 21:241‐246. <https://doi.org/10.1590/s1806-83242007000300009>

Chambrone L, Salinas Ortega M, Sukekava F, et al (2018) Root coverage procedures for treating localised and multiple recession‐type defects. Cochrane Database of Systematic Reviews. <https://doi.org/10.1002/14651858.CD007161.pub3>

Champlin TL, Mallory SB (1989) Hypohidrotic ectodermal dysplasia: a review. The Journal of the Arkansas Medical Society 86:115–117

Chan C., Kaur R. (2017) Unforgettable 7 steps & 5 moments"-critique on staff’s technique and accuracy. Antimicrobial Resistance and Infection Control 6:. <https://doi.org/10.1186/s13756-017-0176-1>

Chan KHC, Ho EHT, Botelho MG, Pow EHN (2011) Rehabilitation of amelogenesis imperfecta using a reorganized approach: a case report. Quintessence international (Berlin, Germany : 1985) 42:385–391

Chan YL, Ngan AHW, King NM (2010) Degraded prism sheaths in the transition region of hypomineralized teeth. Journal of dentistry 38:237–244. <https://doi.org/10.1016/j.jdent.2009.11.003>

Chandna P., Adlakha V.K. (2010) Oral health in children - Guidelines for pediatricians. Indian Pediatrics 47:323–327. <https://doi.org/10.1007/s13312-010-0061-y>

Chang S.K.Y., Tay C.W., Iyer S.G., et al (2016) Long-term oncological safety of minimally invasive hepatectomy in patients with hepatocellular carcinoma: A case-control study. Annals of the Academy of Medicine Singapore 45:91–97

Chapple JR, Nunn JH (2001) The oral health of children with clefts of the lip, palate, or both. The Cleft palate-craniofacial journal : official publication of the American Cleft Palate-Craniofacial Association 38:525–528

Chattoraj A., Bhattacharyya S., Basu D., et al (2005) Melatonin accelerates maturation inducing hormone (MIH): Induced oocyte maturation in carps. General and Comparative Endocrinology 140:145–155. <https://doi.org/10.1016/j.ygcen.2004.10.013>

Chau SS, King NM (1989) An in vitro investigation of developmental defects of enamel under wet and dry conditions. The New Zealand dental journal 85:78–82

Chau S.S., King N.M. (1989) An in vitro investigation of developmental defects of enamel under wet and dry conditions. The New Zealand dental journal 85:78–82

Chávez Jaramillo NV (2018) Prevalencia de hipomineralización incisivo–molar (HIM) en niños entre 9-12 años de edad pertenecientes a dos escuelas de Quito, Ecuador entre febrero y marzo de 2018

Chawla N, Messer LB, Silva M (2008) Clinical studies on molar-incisor-hypomineralisation part 1: distribution and putative associations. European archives of paediatric dentistry : official journal of the European Academy of Paediatric Dentistry 9:180–190

Chawla N., Messer L.B., Silva M. (2008) Clinical studies on molar-incisor-hypomineralisation part 2: development of a severity index. European archives of paediatric dentistry : official journal of the European Academy of Paediatric Dentistry 9:191–199. <https://doi.org/10.1007/BF03262635>

Chay PL, Manton DJ, Palamara JE (2014a) The effect of resin infiltration and oxidative pre‐treatment on microshear bond strength of resin composite to hypomineralised enamel. International journal of paediatric dentistry 24:252–267

Chay PL, Manton DJ, Palamara JEA (2014b) The effect of resin infiltration and oxidative pre-treatment on microshear bond strength of resin composite to hypomineralised enamel. International journal of paediatric dentistry 24:252–267. <https://doi.org/10.1111/ipd.12069>

Chellappah NK, Lo GL, Vignehsa H (1986) Enamel defects in permanent maxillary incisors of Singaporean children. Annals of the Academy of Medicine, Singapore 15:280–283

Chen B, Szabo D, Shen Y, et al (2020) Removal of calcifications from distal canals of mandibular molars by a non-instrumentational cleaning system: A micro-CT study. Australian endodontic journal : the journal of the Australian Society of Endodontology Inc 46:11–16. <https://doi.org/10.1111/aej.12376>

Chen C-F, Hu JCC, Estrella MRP, et al (2013a) Assessment of restorative treatment of patients with amelogenesis imperfecta. Pediatric dentistry 35:337–342

Chen E., Yuan Z.-A., Li Y., et al (2003) The Small Bovine Amelogenin LRAP Fails to Rescue the Amelogenin Null Phenotype. Calcified Tissue International 73:487–495. <https://doi.org/10.1007/s00223-002-0036-7>

Chen H., Gu X., Zeng Q., et al (2019) Carbamazepine disrupts molting hormone signaling and inhibits molting and growth of Eriocheir sinensis at environmentally relevant concentrations. Aquatic Toxicology 208:138–145. <https://doi.org/10.1016/j.aquatox.2019.01.010>

Chen H, Pediatric Diffuse Parenchymal Lung Disease/Pediatric Interstitial Lung Disease Cooperative Group, Subspecialty Group of Respiratory Diseases, Society of Pediatrics CMA (2011) [Clinical study on interstitial lung disease in children of China]. Zhonghua er ke za zhi = Chinese journal of pediatrics 49:734–739

Chen H.-L., Zhao C.-C., Sun Y., et al (2012) Guided bone regeneration repairs human mandibular bone defects: Histological observation. Chinese Journal of Tissue Engineering Research 16:8589–8592. <https://doi.org/10.3969/j.issn.2095-4344.2012.46.008>

Chen J, Hu H, Guo J, et al (2010) Correlation between dental maturity and cervical vertebral maturity. Oral surgery, oral medicine, oral pathology, oral radiology, and endodontics 110:777–783. <https://doi.org/10.1016/j.tripleo.2010.08.006>

Chen S, Li B, Lin S, et al (2013b) Change of urinary fluoride and bone metabolism indicators in the endemic fluorosis areas of southern China after supplying low fluoride public water. BMC public health 13:156. <https://doi.org/10.1186/1471-2458-13-156>

Chen S.-J., Lin X., Liu H., Zheng X.-W. (2016) Ameloblastic fibro-odontosarcoma of the mandible in a pediatric patient. European Annals of Otorhinolaryngology, Head and Neck Diseases 133:419–421. <https://doi.org/10.1016/j.anorl.2015.11.010>

Chen Y, Zhou F, Peng Y, et al (2019) Non-syndromic occurrence of true generalized microdontia with hypodontia: A case report. Medicine 98:e16283. <https://doi.org/10.1097/MD.0000000000016283>

Cheng L., Kuehn M.H., Cring M.R. (2019) Modeling oculodentodigital dysplasia syndrome using human induced pluripotent stem cells. Investigative Ophthalmology and Visual Science 60:

Cheng S.-X., Zhang S., Sun H.-T., Tu Y. (2013) Effects of mild hypothermia treatment on rat hippocampal beta-amyloid expression following traumatic brain injury. Therapeutic Hypothermia and Temperature Management 3:132–139. <https://doi.org/10.1089/ther.2013.0005>

Cherry N, Burstyn I, Beach J (2012) Mental ill-health and second claims for work-related injury. Occupational medicine (Oxford, England) 62:462–465. <https://doi.org/10.1093/occmed/kqs137>

Chesterman J, Durey K (2017) Management of enamel defects: a case report of identical twins with enamel defects of the permanent dentition. Dental Update 44:1049–1056

Chi N-C, Huang S-T, Liu H-Y, et al (2016) A Four-year Case Analysis of Dental Treatment with Office-Based Intravenous Sedation in Taiwan. 臺灣兒童牙醫學雜誌 16:39–45

Chiantella G, Sculean A, Windisch P (2000) Treatment of intrabony defects with enamel matrix proteins and a bovine derived xenograft. A controlled clinical study. Journal of clinical periodontology 27:62, Abstract no: 176

Chiarantin G., Neves R., Carmona A., et al (2013) PHEX substrate protein osteopontin and its asarm peptide decrease NAPT2A expression. Journal of Bone and Mineral Research 28:

ChiCTR1800016195 (2018) Multi-center clinical application of high intensity laser, weak laser and photodynamic therapy for oral soft and hard tissue diseases. http://www.who.int/trialsearch/Trial2.aspx?TrialID=ChiCTR1800016195

Chikte U.M., Louw A.J., Stander I. (2001) Perceptions of fluorosis in northern Cape communities. SADJ : journal of the South African Dental Association = tydskrif van die Suid-Afrikaanse Tandheelkundige Vereniging 56:528–532

Chindasombatjaroen J., Poomsawat S., Boonsiriseth K. (2014) Two unique cases of calcifying cystic odontogenic tumor in the maxillary posterior region. Oral surgery, oral medicine, oral pathology and oral radiology 118:497–504. <https://doi.org/10.1016/j.oooo.2014.06.006>

Chitsazi M, Mostofi Zadeh Farahani R, Pourabbas M, Bahaeddin N (2007) Efficacy of open flap debridement with and without enamel matrix derivatives in the treatment of mandibular degree II furcation involvement. Clinical oral investigations 11:385‐389. <https://doi.org/10.1007/s00784-007-0134-z>

Cho S-Y, Ki Y, Chu V (2008) Molar incisor hypomineralization in Hong Kong Chinese children. International journal of paediatric dentistry 18:348–352. <https://doi.org/10.1111/j.1365-263X.2008.00927.x>

Cho Y.-A., Yoon H.-J., Hong S.-P., et al (2011) Multiple calcifying hyperplastic dental follicles: Comparison with hyperplastic dental follicles. Journal of Oral Pathology and Medicine 40:243–249. <https://doi.org/10.1111/j.1600-0714.2010.00968.x>

Choea N., Bragera R., Weeb W., et al (2018) A case of KAT6A mutation associated with immunodeficiency and granulomatous lymphocytic interstitial lung disease. LymphoSign Journal 5:146–148. <https://doi.org/10.14785/lymphosign-2018-0014>

Chong C.C.-N., Lee K.-F., Lai P.B.-S., et al (2020) Laparoscopic Hepatectomy (with or without Robotic Assistance) versus Radiofrequency Ablation as a Minimally Invasive Treatment for Very Early-Stage or Early-Stage Hepatocellular Carcinoma. Digestive surgery 37:65–71. <https://doi.org/10.1159/000497112>

Choubisa S.L. (2010) Osteo-dental fluorosis in domestic horses and donkeys in Rajasthan, India. Fluoride 43:5–12

Choudhary P, Tandon S, Ganesh M, Mehra A (2012) Evaluation of the remineralization potential of amorphous calcium phosphate and fluoride containing pit and fissure sealants using scanning electron microscopy. Indian journal of dental research 23:157‐163. <https://doi.org/10.4103/0970-9290.100419>

Choung H.-W., Lee D.-S., Lee H.-K., et al (2016) Preameloblast-Derived Factors Mediate Osteoblast Differentiation of Human Bone Marrow Mesenchymal Stem Cells by Runx2-Osterix-BSP Signaling. Tissue Engineering - Part A 22:93–102. <https://doi.org/10.1089/ten.tea.2015.0272>

Chow MH, Peterson DS (1979) Dental management for children with chronic renal failure undergoing hemodialysis therapy. Oral surgery, oral medicine, and oral pathology 48:34–38

Christensen AE, Knappskog PM, Midtbø M, et al (2010) Brittle cornea syndrome associated with a missense mutation in the zinc-finger 469 gene. Investigative ophthalmology & visual science 51:47–52. <https://doi.org/10.1167/iovs.09-4251>

Chtioui F, Marouane O, Douki N White Spot Lesions: A New Topographic Classification

Chu EY, Tamasas B, Fong H, et al (2016) Full Spectrum of Postnatal Tooth Phenotypes in a Novel Irf6 Cleft Lip Model. Journal of dental research 95:1265–1273. <https://doi.org/10.1177/0022034516656787>

Chu FCS (2009) Clinical considerations in managing severe tooth discoloration with porcelain veneers. Journal of the American Dental Association (1939) 140:442–446

Chuchmai LD, Gumetskii RA (1973) [Incidence of dental enamel hypoplasia and fluorosis in school children of Lvov and their need for orthodontic treatment]. Stomatologiia 52:79–80

Chung J.S., Webster S.G. (2005) Dynamics of in vivo release of molt-inhibiting hormone and crustacean hyperglycemic hormone in the shore crab, carcinus maenas. Endocrinology 146:5545–5551. <https://doi.org/10.1210/en.2005-0859>

Ciarrocchi I, Masci C, Spadaro A, et al (2012) Dental enamel, fluorosis and amoxicillin. La Pediatria medica e chirurgica : Medical and surgical pediatrics 34:148–154

Cicero G., Cicero B. (2019) Predictable surgical planning for implant placement and alveolar ridge augmentation- utilization of three-dimensional printed cone beam radiographic series using ORAL3D software to enhance surgical and prosthetic outcomes. International Journal of Oral and Maxillofacial Surgery 48:48. <https://doi.org/10.1016/j.ijom.2019.03.145>

Cisneros C, Gómez M, Vaca M, et al (2017) Temporary restorative treatment in children and adolescents with amelogenesis imperfecta: Scoping review. Journal of Oral Research 6:324–330

Ciusa G., Malagoli A., Milic J., et al (2019) Modelling 2018 AHA cholesterol guidelines in HIV. Antiviral Therapy 24:

Clarissa B, Daniela H (2018) Гипоминерализованные молочные вторые моляры (HSPM) и молярно-резцовая гипоминерализация (MIH): лечение с применением готовых металлических коронок. Стоматология детского возраста и профилактика 17:47–51

Clark E.J., Chesnutt S.R., Verstraete F.J.M., et al (2017) Dental and Temporomandibular Joint Pathology of the American Black Bear (Ursus americanus). Journal of Comparative Pathology 156:240–250. <https://doi.org/10.1016/j.jcpa.2016.11.267>

Clarkson J (1989) Review of terminology, classifications, and indices of developmental defects of enamel. Advances in dental research 3:104–109

Clarkson J, O’Mullane D (1989) A modified DDE Index for use in epidemiological studies of enamel defects. Journal of dental research 68:445–450

Clarkson JJ, O’Mullane DM (1992) Prevalence of enamel defects/fluorosis in fluoridated and non-fluoridated areas in Ireland. Community dentistry and oral epidemiology 20:196–199

Clayton CJ (2015) The John Kay Williams Gold Medal of the Royal College of Surgeons of England and Glasgow 2013. Journal of orthodontics 42:159–171. <https://doi.org/10.1179/1465313314Y.0000000124>

Clelland E.S., Tan Q., Balofsky A., et al (2007) Inhibition of premature oocyte maturation: A role for bone morphogenetic protein 15 in zebrafish ovarian follicles. Endocrinology 148:5451–5458. <https://doi.org/10.1210/en.2007-0674>

Cobourne M.T., Goodman J.R., Spencer T. (1996) Oral manifestations seen in association with a case of trisomy for the short arm of chromosome 9. Pediatric dentistry 18:465–468

Cobourne MT, Williams A, Harrison M (2014) National clinical guidelines for the extraction of first permanent molars in children. British dental journal 217:643–648. <https://doi.org/10.1038/sj.bdj.2014.1053>

Cocco A, Lund R, Torre E, Martos J (2016) Treatment of fluorosis spots using a resin infiltration technique: 14-month follow-up. Operative dentistry 41:357–362

Cochran DL, Jones A, Heijl L, et al (2003a) Periodontal regeneration with a combination of enamel matrix proteins and autogenous bone grafting. Journal of periodontology 74:1269–1281

Cochran DL, King GN, Schoolfield J, et al (2003b) The effect of enamel matrix proteins on periodontal regeneration as determined by histological analyses. Journal of periodontology 74:1043–1055

Cochran JA, Ketley CE, Arnadóttir IB, et al (2004) A comparison of the prevalence of fluorosis in 8-year-old children from seven European study sites using a standardized methodology. Community dentistry and oral epidemiology 32:

Cockburn F., Belton N.R., Purvis R.J., et al (1980) Maternal vitamin D intake and mineral metabolism in mothers and their newborn infants. British Medical Journal 281:11–14

Coelho Jr. L.G.T.M., Caldas Jr. A.F., Soriano E.P., et al (2009) Christ Siemens Touraine syndrome: A case report. Cases Journal 2:. <https://doi.org/10.1186/1757-1626-2-38>

Coffield K.D., Brady M., Roberts M.W., et al (2005) The psychosocial impact of developmental dental defects in people with hereditary amelogenesis imperfecta. Journal of the American Dental Association 136:620–630. <https://doi.org/10.14219/jada.archive.2005.0233>

Cogo E., Sibilla P., Turrini R. (2018) Correlazione tra pH dei prodotti sbiancanti e danni sullo smalto post-sbiancamentoRelationship between whitening gels pH and damages on enamel after bleaching. Dental Cadmos 86:142–146. <https://doi.org/10.19256/d.cadmos.02.2018.10>

Cogulu D, Becerik S, Emingil G, et al (2009) Oral rehabilitation of a patient with amelogenesis imperfecta. Pediatric dentistry 31:523–527

Cogulu D., Cagirir F.D., Hazan F. (2015) Orofacial findings and dental management of Williams syndrome. Genetic Counseling 26:437–442

Cogulu D, Han B, Yetkiner E, Ertuğrul F (2007) Combined apexification and orthodontic extrusion of a hypoplastic permanent canine. Journal of dentistry for children (Chicago, Ill) 74:221–223

Cohen S, Becker GL (1976) Origin, diagnosis, and treatment of the dental manifestations of vitamin D-resistant rickets: review of the literature and report of case. Journal of the American Dental Association (1939) 92:120–129

Colaco M.V., Barroso R.C., Porto I.M., et al (2012) Synchrotron X-ray diffraction characterization of healthy and fluorotic human dental enamel. Radiation Physics and Chemistry 81:1578–1585. <https://doi.org/10.1016/j.radphyschem.2012.05.011>

Colares Neto G., De Souza S., Antequera R., et al (2012) Dental abnormalities in children with X-linked hypophosphatemic rickets confirmed by the presence of PHEX mutations. Hormone Research in Paediatrics 78:37

Colella RF (1968) Dental care. Pediatric clinics of North America 15:325–336

Coley-Smith A, Brown CJ (1996) Case report: radical management of an adolescent with amelogenesis imperfecta. Dental update 23:434–435

Collard M., Azzopardi K.A., Hall S.J., et al (2016) Oral health in children with cystic fibrosis. Journal of Cystic Fibrosis 15:

Collins MA, Mauriello SM, Tyndall DA, Wright JT (1999) Dental anomalies associated with amelogenesis imperfecta: a radiographic assessment. Oral surgery, oral medicine, oral pathology, oral radiology, and endodontics 88:358–364

Colombo S, Ferrazzan G, Beretta M, Paglia L Dental Caries Prevention: A Review on the Use of Dental Sealants

Colquhoun J. (1984) Disfiguring dental fluorosis in Auckland, New Zealand. Fluoride - Quarterly Reports 17:234–242

Comes Martínez Á, Puente Ruiz C de la, Rodríguez Salvanés F (2007) Prevalencia de Hipomineralización en primeros molares permanentes (MIH) en población infantil del Área 2 de Madrid. RCOE 12:129–134

Compañías De Salud Y Clinica Dental Elvira Martinez

Compañías De Salud Y Consultorio Dental M & L

Compañías De Salud Y Odontopediatra Carla Cabrejas

Condò R, Perugia C, Maturo P, Docimo R (2012) MIH: epidemiologic clinic study in paediatric patient. ORAL & implantology 5:58–69

Conklin WW (1978) Long-term follow-up and evaluation of transplantation of fully developed teeth. Oral surgery, oral medicine, and oral pathology 46:477–485

Cooke MS, Neesome PR (1990) Combined orthodontic and restorative correction of severe anterior open bite. Quintessence international (Berlin, Germany : 1985) 21:729–736

Coplan M.J., Bachman M.S., Patch S.C., Masters R.D. (2007) Confirmation of and explanations for elevated blood lead and other disorders in children exposed to water disinfection and fluoridation chemicals. NeuroToxicology 28:1032–1042. <https://doi.org/10.1016/j.neuro.2007.02.012>

Corazza G.R., Gasbarrini G. (1995) Coeliac disease in adults. Bailliere’s Clinical Gastroenterology 9:329–350. <https://doi.org/10.1016/0950-3528%2895%2990034-9>

Corbella S, Alberti A, Calciolari E, et al (2019) Enamel matrix derivative for the treatment of partially contained intrabony defects: 12-month results. Australian dental journal 64:27‐34

Cordaro L, di Torresanto V, Torsello F (2012) Split-mouth comparison of a coronally advanced flap with or without enamel matrix derivative for coverage of multiple gingival recession defects: 6- and 24-month follow-up. International journal of periodontics & restorative dentistry 32:e10‐20

Cordeiro R (2013) JF Souza, F. Jeremias, CM Costa-Silva, L. Santos-Pinto, ACC Zuanon &. Eur Arch Paediatr Dent 14:233–238

CORONA TABARES MG, AGUIAR FUENTES EG, ROBLES ROMERO D, et al (2016) Pasta triantibiotica en pulpotomías de dientes permanentes. Reporte de un caso clínico. CONACYT

Corral-Núñez C, Rodríguez H, Cabello R, et al (2016) Impacto de la hipomineralización incisivo molar en la experiencia de caries en escolares de 6-12 años en Santiago, Chile. Revista clínica de periodoncia, implantología y rehabilitación oral 9:277–283

Corral-Núnez C, Rodríguez H, Cabello R, et al (2016) Revista Clínica de Periodoncia, Implantología y Rehabilitación Oral

Corrêa MG, Campos MLG, Benatti BB, et al (2010) The impact of cigarette smoke inhalation on the outcome of enamel matrix derivative treatment in rats: histometric analysis. Journal of periodontology 81:1820–1828. <https://doi.org/10.1902/jop.2010.100200>

Correa M.G., Gomes Campos M.L., Casati M.Z., et al (2016) Alcohol intake may impair bone density and new cementum formation after enamel matrix derivative treatment: histometric study in rats. Journal of periodontal research 51:60–69. <https://doi.org/10.1111/jre.12279>

Correa M.G., Gomes Campos M.L., Marques M.R., et al (2014) Outcome of enamel matrix derivative treatment in the presence of chronic stress: histometric study in rats. Journal of periodontology 85:. <https://doi.org/10.1902/jop.2013.130383>

Corrêa MG, Gomes Campos ML, Marques MR, et al (2013) Histometric analysis of the effect of enamel matrix derivative on the healing of periodontal defects in rats with diabetes. Journal of periodontology 84:1309–1318. <https://doi.org/10.1902/jop.2012.120354>

Corrêa-Faria P, Paixão-Gonçalves S, Paiva SM, et al (2015) Association between developmental defects of enamel and early childhood caries: a cross-sectional study. International journal of paediatric dentistry 25:103–109. <https://doi.org/10.1111/ipd.12105>

Corrêa-Faria P, Paixão-Gonçalves S, Ramos-Jorge ML, et al (2019) Developmental enamel defects are associated with early childhood caries: Case-control study. International journal of paediatric dentistry. <https://doi.org/10.1111/ipd.12574>

Corruccini RS, Townsend GC (2003) Decline in enamel hypoplasia in relation to fluoridation in Australians. American journal of human biology : the official journal of the Human Biology Council 15:795–799

Corruccini R.S., Townsend G.C., Schwerdt W. (2005) Correspondence between enamel hypoplasia and odontometric bilateral asymmetry in Australian twins. American Journal of Physical Anthropology 126:177–182. <https://doi.org/10.1002/ajpa.20113>

Cortellini P, Nieri M, Prato GP, Tonetti MS (2008) Single minimally invasive surgical technique with an enamel matrix derivative to treat multiple adjacent intra-bony defects: clinical outcomes and patient morbidity. Journal of clinical periodontology 35:605–613. <https://doi.org/10.1111/j.1600-051X.2008.01242.x>

Cortellini P, Stalpers G, Mollo A, Tonetti M (2011) Periodontal regeneration versus extraction and prosthetic replacement of teeth severely compromised by attachment loss to the apex: 5-year results of an ongoing randomized clinical trial. Journal of clinical periodontology 38:915‐924. <https://doi.org/10.1111/j.1600-051X.2011.01768.x>

Cortellini P, Tonetti M (2005) Clinical performance of a regenerative strategy for intrabony defects: scientific evidence and clinical experience. Journal of periodontology 76:341‐350. <https://doi.org/10.1902/jop.2005.76.3.341>

Cortellini P, Tonetti M (2011) Clinical and radiographic outcomes of the modified minimally invasive surgical technique with and without regenerative materials: a randomized-controlled trial in intra-bony defects. Journal of clinical periodontology 38:365‐373. <https://doi.org/10.1111/j.1600-051X.2011.01705.x>

Cortellini P., Tonetti M.S. (2007a) A minimally invasive surgical technique with an enamel matrix derivative in the regenerative treatment of intra-bony defects: A novel approach to limit morbidity. Journal of Clinical Periodontology 34:87–93. <https://doi.org/10.1111/j.1600-051X.2006.01020.x>

Cortellini P., Tonetti M.S. (2007b) Minimally invasive surgical technique and enamel matrix derivative in intra-bony defects. I: Clinical outcomes and morbidity. Journal of Clinical Periodontology 34:1082–1088. <https://doi.org/10.1111/j.1600-051X.2007.01144.x>

Côrtes LCA (2015) Protocolo de tratamento de hipomineralização molar-incisivo em odontopediatria: relato de caso clínico

Cortese A, Vita G, Obici L, et al (2015) Monitoring safety and effectiveness of Tafamidis in transthyretin amyloidosis in Italy: a 3-year longitudinal multicenter study in a non-endemic area. Orphanet journal of rare diseases Conference: 1st european congress on hereditary ATTR amyloidosis France Conference start: 20151102 Conference end: 20151103 10:. <https://doi.org/10.1186/1750-1172-10-S1-P6>

Cossellu G., Angiero F., Farronato G. (2014) Complete pre-eruptive idiopathic crown resorption. Pediatric dentistry 36:147–150

Costa DC, Dourado MR, Figueiredo de Carvalho MF, et al Enamel Renal Syndrome: A Case History Report. The International journal of prosthodontics 30:22–24. <https://doi.org/10.11607/ijp.4916>

Cottin V (2007) [Interstitial lung disease in connective tissue diseases]. La Revue du praticien 57:2235–2242

Cottin V, Cordier J-F (2012) Cryptogenic organizing pneumonia. Seminars in respiratory and critical care medicine 33:462–475

Cottin V, Loire R, Chalabreysse L, et al (2001) [Nonspecific interstitial pneumonitis: a new anatomoclinical entity among idiopathic diffuse interstitial pneumonias]. Revue des maladies respiratoires 18:25–33

Courson F Violaine Smaïl-Faugeron, Michèle Muller-Bolla, 3, 4 Jean-Louis Sixou, 5

Coutinho T, Lenzi M, Simões M, Campos V (2011) Duplication of a permanent maxillary incisor root caused by trauma to the predecessor primary tooth: clinical case report. International endodontic journal 44:688–695. <https://doi.org/10.1111/j.1365-2591.2010.01842.x>

Couto LV, del Pozo EP, Escobar JIS, et al (2020) Oral manifestations in premature infants. A systematic review

Coventry J (2010) Book Review: Oral and Maxillofacial Medicine: The Basis of Diagnosis and Treatment

Covi JA, Chang ES, Mykles DL (2009) Conserved role of cyclic nucleotides in the regulation of ecdysteroidogenesis by the crustacean molting gland. Comparative biochemistry and physiology Part A, Molecular & integrative physiology 152:470–477. <https://doi.org/10.1016/j.cbpa.2008.12.005>

Crabb JJ (1975) The restoration of hypoplastic anterior teeth using an acid-etched technique. Journal of dentistry 3:121–124

Crabb J.J. (1975) The restoration of hypoplastic anterior teeth using an acid-etched technique. Journal of dentistry 3:121–124

Craig SA, Baker SR, Rodd HD (2015) How do children view other children who have visible enamel defects? International Journal of Paediatric Dentistry 25:399–408

Craveia J, Rouas P, Carat T, et al (2020) Knowledge and Management of First Permanent Molars with Enamel Hypomineralization among Dentists and Orthodontists. The Journal of clinical pediatric dentistry 44:20–27. <https://doi.org/10.17796/1053-4625-44.1.4>

Crawford P.J., Aldred M., Bloch-Zupan A. (2007) Amelogenesis imperfecta. Orphanet journal of rare diseases 2:17. <https://doi.org/10.1186/1750-1172-2-17>

Crawford PJM, Aldred M, Bloch-Zupan A (2007) Amelogenesis imperfecta. Orphanet journal of rare diseases 2:17

Crea A, Dassatti L, Hoffmann O, et al (2008) Treatment of intrabony defects using guided tissue regeneration or enamel matrix derivative: a 3-year prospective randomized clinical study. Journal of periodontology 79:2281‐2289. <https://doi.org/10.1902/jop.2008.080135>

Créton MA, Cune MS (2004) [An enamel disorder in two siblings]. Nederlands tijdschrift voor tandheelkunde 111:400–402

Creton M.A., Cune M.S. (2004) Een glazuurafwijking bij twee zusjesAn enamel disorder in two siblings. Nederlands tijdschrift voor tandheelkunde 111:400–402

Crincoli V, Di Bisceglie MB, Scivetti M, et al (2010) Dens invaginatus: a qualitative-quantitative analysis. Case report of an upper second molar. Ultrastructural pathology 34:7–15. <https://doi.org/10.3109/01913120903506595>

Croll TP (1990) Enamel microabrasion for removal of superficial dysmineralization and decalcification defects. Journal of the American Dental Association (1939) 120:411–415

Croll TP (1991) Creating the appearance of white enamel dysmineralization with bonded resins. Journal of esthetic dentistry 3:30–33

Croll TP (1993) Bonded composite resin restoration of a smooth-surface enamel hypoplasia lesion. Practical periodontics and aesthetic dentistry : PPAD 5:25

CROMBIE F (2011) An investigation into developmentally hypomineralised enamel in first permanent molar teeth

Crombie F, Manton D, Kilpatrick N (2009a) Aetiology of molar–incisor hypomineralization: a critical review. International Journal of Paediatric Dentistry 19:73–83

Crombie F, Manton D, Kilpatrick N (2009b) Aetiology of molar-incisor hypomineralization: a critical review. International journal of paediatric dentistry 19:73–83. <https://doi.org/10.1111/j.1365-263X.2008.00966.x>

Crombie F, Manton DJ (2014) Managing the Prevention of Dental Caries and Sensitivity in Teeth. Planning and Care for Children and Adolescents with Dental Enamel Defects: Etiology, Research and Contemporary Management 113

Crombie F, Manton DJ (2015) Managing the Prevention of Dental Caries and Sensitivity in Teeth with Enamel Defects. In: Planning and Care for Children and Adolescents with Dental Enamel Defects. Springer, pp 113–122

Crombie FA, Cochrane NJ, Manton DJ, et al (2013a) Mineralisation of developmentally hypomineralised human enamel in vitro. Caries research 47:259–263. <https://doi.org/10.1159/000346134>

Crombie FA, Manton DJ, Palamara JEA, et al (2013b) Characterisation of developmentally hypomineralised human enamel. Journal of dentistry 41:611–618. <https://doi.org/10.1016/j.jdent.2013.05.002>

Crombie FA, Manton DJ, Weerheijm KL, Kilpatrick NM (2008) Molar incisor hypomineralization: a survey of members of the Australian and New Zealand Society of Paediatric Dentistry. Australian dental journal 53:160–166. <https://doi.org/10.1111/j.1834-7819.2008.00026.x>

Cros P, Achard R, Dumas P (1976) [Hereditary enamel dysplasia. Therapeutic approach apropos of 2 cases]. Revue de stomatologie et de chirurgie maxillo-faciale 77:1021–1027

Cross KJ, Huq NL, Reynolds EC (2007) Casein phosphopeptides in oral health--chemistry and clinical applications. Current pharmaceutical design 13:793–800

Cruvinel VRN, Gravina DBL, Azevedo TDPL, et al (2012) Prevalence of enamel defects and associated risk factors in both dentitions in preterm and full term born children. Journal of applied oral science : revista FOB 20:310–317

Cruz PF, de Lima M de DM CONDIÇÕES DE SAÚDE BUCAL E NECESSIDADES DE TRATAMENTO DE INDIVÍDUOS COM HIPOMINERALIZAÇÃO DE MOLARES E INCISIVOS.

Cruz PF, Neta NBD, de Aguiar AS, Castro G FÓRUM CIENTÍFICO–EPIDEMIOLOGIA–Acadêmico–Pesquisa Científica 28 PERCEPÇÃO DOS PAIS SOBRE A QUALIDADE DE VIDA DE ESCOLARES COM HIPOMINERALIZAÇÃO MOLAR-INCISIVO

CTRI/2011/12/002208 (2011) To compare the usefulness and side effects of Deflazacort and Prednisolone in Nephrotic syndrome in children. http://www.who.int/trialsearch/Trial2.aspx?TrialID=CTRI/2011/12/002208

CTRI/2016/09/007232 (2016) To Compare the effect of two fluoride varnishes on dental enamel demineralization in patients undergoing Braces treatment. http://www.who.int/trialsearch/Trial2.aspx?TrialID=CTRI/2016/09/007232

CTRI/2016/10/007379 (2016) Comparative evaluation of cast metal and indirect composite inlays for first permanent molars affected with molar incisor hypomineralization. http://www.who.int/trialsearch/Trial2.aspx?TrialID=CTRI/2016/10/007379

CTRI/2018/02/012223 (2018) Comparing the effect of three new materials for the treatment of deep cavities. http://www.who.int/trialsearch/Trial2.aspx?TrialID=CTRI/2018/02/012223

CTRI/2018/05/013674 (2018) Treatment of dental fluorosis using combined techniques. http://www.who.int/trialsearch/Trial2.aspx?TrialID=CTRI/2018/05/013674

CTRI/2018/07/014785 (2018) A clinical study to evaluate the effect of new medicament to save the primary teeth. http://www.who.int/trialsearch/Trial2.aspx?TrialID=CTRI/2018/07/014785

CTRI/2019/01/017345 (2019) Effectiveness Of Recent Remineralizing Agents On Defects Of Permanent Incisors. http://www.who.int/trialsearch/Trial2.aspx?TrialID=CTRI/2019/01/017345

CTRI/2019/02/017482 (2019) Comparing two fillings for teeth with soft enamel. http://www.who.int/trialsearch/Trial2.aspx?TrialID=CTRI/2019/02/017482

CTRI/2019/03/018005 (2019) Comparison of two restoration techniques for molar teeth affected with hypominerlization. http://www.who.int/trialsearch/Trial2.aspx?TrialID=CTRI/2019/03/018005

Cubukçu CE, Güneş AM (2008) Caries experience of leukemic children during intensive course of chemotherapy. The Journal of clinical pediatric dentistry 32:155–158

Cuesta M, JC AL, AM GG, JM BG (2018) Prevalencia de hipomineralización incisivo-molar y características clínico-epidemiológicas en dos cohortes de edad de población infantil. RCOE: Revista del Ilustre Consejo General de Colegios de Odontólogos y Estomatólogos de España 23:6–13

Cui J, Yang K, Yu X, et al (2016) Contents Vol. 25, 2016. Medical Principles and Practice 25:I–VI

Cunha NAJ (2015) Alternativas terapêuticas na abordagem da Hipomineralização Incisivo-Molar

Cunha R.F., de Oliveira D.C., Favretto C.O. (2015) Molar incisor hypomineralization: considerations about treatment in a controlled longitudinal case. Journal of the Indian Society of Pedodontics and Preventive Dentistry 33:152–155. <https://doi.org/10.4103/0970-4388.155133>

Curzon M, Ogden A, Williams-Ward M, Cleaton-Jones P (2015) Case report: A medieval case of molar-incisor-hypomineralisation. British Dental Journal 219:583–587

Curzon M, Roberts J, Toumba J (2017) Too many cooks…?

Curzon M.E.J., Spector P.C. (1977) Enamel mottling in a high strontium area of the USA. Community Dentistry and Oral Epidemiology 5:243–247. <https://doi.org/10.1111/j.1600-0528.1977.tb01648.x>

Cutress TW, Suckling GW (1990) Differential diagnosis of dental fluorosis. Journal of dental research 69:

Czarnetzki A, Pusch CM (2001) [Classification of a 300,000-year-old dental crown of the upper loamy deposit of the Bad Canstatter travertine zone]. Anthropologischer Anzeiger; Bericht uber die biologisch-anthropologische Literatur 59:289–307

Czarnetzki A., Pusch C.M. (2001) Klassifizierung einer 300.000 Jahre alten Zahnkrone aus dem oberen Lehmhorizont des Bad-Cannstatter TravertinClassification of a 300,000-year-old dental crown of the upper loamy deposit of the Bad Canstatter travertine zone. Anthropologischer Anzeiger; Bericht uber die biologisch-anthropologische Literatur 59:289–307

Da Conceicao Pedro Pais J.A., Picarra B., Guerreiro R.A., et al (2019) New predictive score of mortality and cardiogenic shock in patients with acute myocardial infarction. European Heart Journal: Acute Cardiovascular Care 8:102–103. <https://doi.org/10.1177/2048872619829424>

da Costa VS, Wanderley MT, Haddad AE, et al (2020) Currículo de Odontopediatria nos cursos brasileiros de graduação em Odontologia. Revista da ABENO 20:93–101

Da Costa-Silva C.M., Ambrosano G.M.B., Mialhe F.L., et al (2011) Increase in severity of molar-incisor hypomineralization and its relationship with the colour of enamel opacity: A prospective cohort study. International Journal of Paediatric Dentistry 21:333–341. <https://doi.org/10.1111/j.1365-263X.2011.01128.x>

da Cruz Rocha R, dos Santos AFL Hipomineralização Molar-Incisivo (HMI): relato de caso

da Cunha Coelho ASE, Mata PCM, Lino CA, et al (2019) Dental hypomineralization treatment: A systematic review. Journal of esthetic and restorative dentistry : official publication of the American Academy of Esthetic Dentistry . [et al] 31:26–39. <https://doi.org/10.1111/jerd.12420>

da Fonseca M, Oueis HS, Casamassimo PS (2007) Sickle cell anemia: a review for the pediatric dentist. Pediatric dentistry 29:159–169

Da Silva Assunção LR, Ferelle A, Iwakura MLH, Cunha RF (2009) Effects on permanent teeth after luxation injuries to the primary predecessors: a study in children assisted at an emergency service. Dental traumatology : official publication of International Association for Dental Traumatology 25:165–170. <https://doi.org/10.1111/j.1600-9657.2008.00759.x>

da Silva Dalben G, Costa B, Gomide MR (2006) Prevalence of dental anomalies, ectopic eruption and associated oral malformations in subjects with Treacher Collins syndrome. Oral surgery, oral medicine, oral pathology, oral radiology, and endodontics 101:588–592

DA SILVA FMF (2016) HIPOMINERALIZAÇÃO MOLAR-INCISIVO: PERCEPÇÃO ESTÉTICA DE CRIANÇAS/ADOLESCENTES E DE SEUS RESPOSÁVEIS

da Silva F.M.F., Vieira F.G.F., Costa M.C., et al (2020) Defining the prevalence of molar incisor hypomineralization in Brazil. Pesquisa Brasileira em Odontopediatria e Clinica Integrada 20:. <https://doi.org/10.1590/pboci.2020.021>

Da Silva Santos P.S., Esperidiao A.P., De Freitas R.R. (2009) Maxillofacial aspects in malignant osteopetrosis. Cleft Palate-Craniofacial Journal 46:388–390. <https://doi.org/10.1597/07-203.1>

da Silva T, de Oliveira H, Severino D, et al (2014) Direct spectrometry: a new alternative for measuring the fluorescence of composite resins and dental tissues. Operative dentistry 39:407–415. <https://doi.org/10.2341/12-464-L>

da Silva-Júnior IF, de Oliveira CR, da Silva Berwig P, Schardosim LR (2018) Reabilitação de dentes afetados pela Hipomineralização Molar-Incisivo (HMI): um relato de caso com 16 meses de acompanhamento. Revista da Faculdade de Odontologia-UPF 23:

Dahl E (1976) Genetic aspects of some orofacial anomalies. Acta oto-laryngologica 82:226–229

Dahllof G., Barr M., Bolme P., et al (1988) Disturbances in dental development after total body irradiation in bone marrow transplant recipients. Oral surgery, oral medicine, and oral pathology 65:41–44

Dahllof G., Rozell B., Forsberg C.M., Borgstrom B. (1994) Histologic changes in dental morphology induced by high dose chemotherapy and total body irradiation. Oral surgery, oral medicine, and oral pathology 77:56–60. <https://doi.org/10.1016/S0030-4220(06)80107-6>

Dahllöf G, Ussisoo-Joandi R, Ideberg M, Modeer T (1989) Caries, gingivitis, and dental abnormalities in preschool children with cleft lip and/or palate. The Cleft palate journal 26:233

Dahlstrom J.E., Wong P. (2015) A t] in a tooth: An invaginated odontome. Pediatric and Developmental Pathology 18:. <https://doi.org/10.2350/14-08-1534-MISC.1>

Daito M., Tanaka T., Hieda T. (1992) Clinical observations on the development of third molars. Journal of Osaka Dental University 26:91–104

Dalben G da S, das Neves LT, Gomide MR (2006) Oral findings in patients with Apert syndrome. Journal of applied oral science : revista FOB 14:465–469

Daly D., Waldron J.M. (2009) Molar incisor hypomineralisation: clinical management of the young patient. Journal of the Irish Dental Association 55:83–86

Daly D, Waldron JM (2009) Molar incisor hypomineralisation: clinical management of the young patient. Journal of the Irish Dental Association 55:83–86

Daneshkazemi A.R., Davari A. (2005) Assessment of DMFT and enamel hypoplasia among junior high school children in Iran. Journal of Contemporary Dental Practice 6:85–92

Dantas Neta NB (2017) Hipomineralização molar-incisivo: prevalência, fatores associados e impacto na qualidade de vida relacionada à saúde bucal de escolares

Dantas-Neta N.B., Soares Figueiredo M., Lima C.C.B., et al (2018) Factors associated with molar-incisor hypomineralisation in schoolchildren aged 8-10 years: a case-control study. International journal of paediatric dentistry 28:570–577. <https://doi.org/10.1111/ipd.12412>

Daou MH, Eden E, El Osta N (2016) Age and reasons of the first dental visit of children in Lebanon. Lebanese Medical Journal 103:1–5

Darmstadt G.L. (1998) Antibiotics in the management of pediatric skin disease. Dermatologic Clinics 16:509–525. <https://doi.org/10.1016/S0733-8635%2805%2970249-7>

Das UM, Prashanth ST (2009) A comparative study to evaluate the effect of fluoride releasing sealant cured by visible light, argon lasers, and light emitting diode curing units: an in vitro study. Journal of the Indian Society of Pedodontics and Preventive Dentistry 27:139–144. <https://doi.org/10.4103/0970-4388.57093>

Dashash M, Yeung CA, Jamous I, Blinkhorn A (2013) Interventions for the restorative care of amelogenesis imperfecta in children and adolescents. The Cochrane database of systematic reviews CD007157. <https://doi.org/10.1002/14651858.CD007157.pub2>

Dastouri M, Kowash M, Al-Halabi M, et al (2020) United Arab Emirates dentists’ perceptions about the management of broken down first permanent molars and their enforced extraction in children: a questionnaire survey. European Archives of Paediatric Dentistry 21:31–41

Dave M, Taylor G (2018) Global prevalence of molar incisor hypomineralisation. Evidence-based dentistry 19:78–79. <https://doi.org/10.1038/sj.ebd.6401324>

Dave M, Thomson F, Barry S, et al (2019) The use of localised CBCT to image inflammatory collateral cysts: a retrospective case series demonstrating clinical and radiographic features. European Archives of Paediatric Dentistry 1–9

Davenport M, Welles AD, Angelopoulou MV, et al (2019) Prevalence of molar-incisor hypomineralization in Milwaukee, Wisconsin, USA: a pilot study. Clinical, cosmetic and investigational dentistry 11:109

Davey, Hall, Willis, et al (2000) Five Crustacean Hyperglycemic Family Hormones of Penaeus monodon: Complementary DNA Sequence and Identification in Single Sinus Glands by Electrospray Ionization-Fourier Transform Mass Spectrometry. Marine biotechnology (New York, NY) 2:80–91

Davidovich E, Dagon S, Tamari I, et al (2020) An Innovative Treatment Approach Using Digital Workflow and CAD-CAM Part 2: The Restoration of Molar Incisor Hypomineralization in Children. International journal of environmental research and public health 17:. <https://doi.org/10.3390/ijerph17051499>

Davidovich E., Davidovits M., Eidelman E., et al (2005) Pathophysiology, therapy, and oral implications of renal failure in children and adolescents: an update. Pediatric dentistry 27:98–106

Davidovich E, Kooby E, Shapira J, Ram D (2013) The traditional practice of canine bud removal in the offspring of Ethiopian immigrants. BMC oral health 13:34. <https://doi.org/10.1186/1472-6831-13-34>

Davies GM, Pretty IA, Neville JS, Goodwin M (2012) Investigation of the value of a photographic tool to measure self-perception of enamel opacities. BMC oral health 12:41. <https://doi.org/10.1186/1472-6831-12-41>

Davila V., Gil N., Marino A., et al (2019) Multifocal infantile hemangioma with hepatic involvement, the importance of a multidisciplinary approach and management of a rare disease. Pediatric Dermatology 36:. <https://doi.org/10.1111/pde.13846>

Davit-Beal T., Gabay J., Antoniolli P., et al (2014) Dental complications of rickets in early childhood: Case report on 2 young girls. Pediatrics 133:. <https://doi.org/10.1542/peds.2013-0733>

de Alencar CDRB, Cavalcanti AL (2018) Hipomineralización de incisivos molares:¿ un desafío de la odontología pediátrica? Journal of Oral Research 7:84–85

De Almeida C.M., Andre S.J., Toscano A., Petersen P.E. (2003) Changing oral health status of 6- And 12-year-old schoolchildren in Portugal. Community Dental Health 20:211–216

De Araújo AMP (2020) Hipomineralização Molar-incisivo-Uma revisão de literatura. Revista Cathedral 2:

de Araujo EB, Zis V, Dutra CA (2000) Enamel color change by microabrasion and resin-based composite. American journal of dentistry 13:6–7

de Araújo LG DIAGNÓSTICO E TRATAMENTO DA HIPOMINERALIZAÇÃO MOLAR INCISIVO (HMI)

de Baat P., Heijboer M.P., de Baat C. (2005) Osteopetrose. Classificatie, etiologie, behandelmogelijkheden en implicaties voor de mondgezondheidOsteopetrosis. Classification, etiology, treatment options and implications for oral health. Nederlands tijdschrift voor tandheelkunde 112:497–503

de Camargo MGA (2011) Otomastoiditis y su posible relación a hipomineralización molar-incisivo. Reporte de caso. Acta Odontologica Venezolana 49:

de Camargo MGA, Natera A (2020a) Level of knowledge concerning enamel defects and their treatment among pediatric dentists. Revista de Odontopediatría Latinoamericana 7:25–35

de Camargo MGA, Natera A (2020b) Nivel de conocimiento de defectos de esmalte y su tratamiento entre odontopediatras. Revista de Odontopediatría Latinoamericana 7:25–35

De Coster P, Martens L (2013) 29 Behandelen van gebitselementen met ontwikkelingsstoornissen. In: Kindertandheelkunde deel 2. Springer, pp 13–24

De Coster P.J., Martens L.C., De Paepe A. (2002) Oral manifestations of patients with Marfan syndrome: a case-control study. Oral surgery, oral medicine, oral pathology, oral radiology, and endodontics 93:564–572

de Crousaz P (1982) Observations on enamel opacities in Switzerland in relation to water or salt fluoridation. Schweizerische monatsschrift fur zahnheilkunde 92:332‐344

de Crousaz P. (1982) Observations sur les opacites d’email en Suisse, en rapport avec la fluoration de l’eau ou du selObservations on enamel opacities in Switzerland in relation to water or salt fluoridation. Schweizerische Monatsschrift fur Zahnheilkunde = Revue mensuelle suisse d’odonto-stomatologie / SSO 92:332–344

De Fatima Guedes de Amorim L., Da Costa L.R.R.S., Estrela C. (2011) Effects of traumatic dental injuries to primary teeth on permanent teeth - a clinical follow-up study. Dental Traumatology 27:117–121. <https://doi.org/10.1111/j.1600-9657.2010.00959.x>

De La Dure-Molla M, Naulin-Ifi C, Jedeon K, et al (2013) Spots on tooth enamel: what’s new? Journal of Dentofacial Anomalies and Orthodontics 16:404

de la Dure-Molla M, Quentric M, Yamaguti PM, et al (2014) Pathognomonic oral profile of Enamel Renal Syndrome (ERS) caused by recessive FAM20A mutations. Orphanet journal of rare diseases 9:84. <https://doi.org/10.1186/1750-1172-9-84>

De La Pena V.A., Valea M.C. (2011) Treatment of enamel hypoplasia in a patient with Usher syndrome. Journal of the American Dental Association 142:938–941. <https://doi.org/10.14219/jada.archive.2011.0300>

De Leeuw N.H., Bowe J.R., Rabone J.A.L. (2007) A computational investigation of stoichiometric and calcium-deficient oxy- and hydroxy-apatites. Faraday Discussions 134:195–214. <https://doi.org/10.1039/b602012g>

De Leonardis D, Paolantonio M (2013) Enamel matrix derivative, alone or associated with a synthetic bone substitute, in the treatment of 1- to 2-wall periodontal defects. Journal of periodontology 84:444‐455. <https://doi.org/10.1902/jop.2012.110656>

de Liefde B. (1988) Longitudinal survey of enamel defects in a cohort of New Zealand children. Community dentistry and oral epidemiology 16:218–221

de Liefde B, Herbison GP (1985) Prevalence of developmental defects of enamel and dental caries in New Zealand children receiving differing fluoride supplementation. Community dentistry and oral epidemiology 13:164–167

de Liefde B., Herbison G.P. (1989) The prevalence of development defects of enamel and dental caries in New Zealand children receiving differing fluoride supplementation, in 1982 and 1985. The New Zealand dental journal 85:2–8

De Oliveira C.A., Cirelli J.A., Marcantonio R.A.C., Spolidorio L.C. (2005) Acellular dermal matrix allograft used alone and in combination with enamel matrix protein in gingival recession: Histologic study in dogs. International Journal of Periodontics and Restorative Dentistry 25:595–603

de Oliveira DC, Favretto CO, Cunha RF (2015a) Molar incisor hypomineralization: considerations about treatment in a controlled longitudinal case. Journal of Indian Society of Pedodontics and Preventive Dentistry 33:152

de Oliveira FV, Silva MFA, Nogueira RD, Geraldo-Martins VR (2015b) Hipoplasia de esmalte em paciente hebiátrico: relato de caso clínico. Revista Odontológica do Brasil Central 24:

de Oliveira LL, dos Santos Freiras FR, de Sá HC, et al (2020) A influência dos fatores genéticos sobre a etiologia da hipomineralização molar-incisivo: revisão de Literatura. Revista Eletrônica Acervo Saúde 12:e3336–e3336

DE PEDODONŢIE D-OC MIH–STUDIU EPIDEMIOLOGIC PE UN LOT DE COPII

de Souza J.F., Giovanini A.F., Gramasco M., et al (2016) Amoxicillin diminishes the thickness of the enamel matrix that is deposited during the secretory stage in rats. International journal of paediatric dentistry 26:199–210. <https://doi.org/10.1111/ipd.12184>

de Souza JF, Gramasco M, Jeremias F, et al (2016) Amoxicillin diminishes the thickness of the enamel matrix that is deposited during the secretory stage in rats. International journal of paediatric dentistry 26:199–210

De Souza N, Vaz A, Chalakkal P (2017) Intracoronal Radiolucency in An Unerupted Premolar: A Rare Occurrence. Journal of clinical and diagnostic research : JCDR 11:ZD04. <https://doi.org/10.7860/JCDR/2017/22791.9135>

de Trabajos R Concurso de Pósters

de Villiers CJ, Phillips VM (1998) Person identification by means of a single unique dental feature. The Journal of forensic odonto-stomatology 16:17–19

De Wit R., Bettarel Y., Roques C., et al (2015) Viruses occur incorporated in biogenic high-Mg calcite from hypersaline microbial mats. PLoS ONE 10:. <https://doi.org/10.1371/journal.pone.0130552>

Dean JA (2015) McDonald and Avery’s Dentistry for the Child and Adolescent-E-Book. Elsevier Health Sciences

Dean J.A., Jones J.E., Vash B.W. (1986) Dental management of oculodentodigital dysplasia: report of case. ASDC journal of dentistry for children 53:131–134

Debnath K., Couthino A., Chatterjee A., Shenoy S. (2019) Enamel renal gingival syndrome: A rare case report. Journal of Indian Society of Periodontology 23:69–72. <https://doi.org/10.4103/jisp.jisp_532_18>

Deen J.L., Von Seidlein L., Dondorp A. (2008) Therapy of uncomplicated malaria in children: A review of treatment principles, essential drugs and current recommendations. Tropical Medicine and International Health 13:1111–1130. <https://doi.org/10.1111/j.1365-3156.2008.02117.x>

del Carmen LJM, Licet Á, Inés S Prevalencia de la Hipomineralización Molar-Incisiva (MIH) en niños con diferente cobertura asistencial (privada y pública) en Montevideo, Uruguay

Del Fabbro M, Karanxha L, Panda S, et al (2018) Autologous platelet concentrates for treating periodontal infrabony defects. Cochrane Database of Systematic Reviews. <https://doi.org/10.1002/14651858.CD011423.pub2>

Delbem A.C., Faraco Junior I.M., Percinoto C. (1996) Natal teeth: case report. The Journal of clinical pediatric dentistry 20:325–327

Delgado M., Clegg N., Sparagana S., et al (2009) Epilepsy in holoprosencephaly: A ten-year study. Annals of Neurology 66:. <https://doi.org/10.1002/ana.21853>

della Vella F., Fucile R., Dibello V., et al (2019) ORO-Dental manifestations in west syndrome. Current Topics in Medicinal Chemistry 19:2824–2828. <https://doi.org/10.2174/1568026619666191114122732>

Demir T, Kecik D, Cehreli ZC (2007) Kenny-Caffey Syndrome: oral findings and 4-year follow-up of overlay denture therapy. Journal of dentistry for children (Chicago, Ill) 74:236–240

Den Besten PK (1999) Mechanism and timing of fluoride effects on developing enamel. Journal of public health dentistry 59:247–251

DenBesten P, Li W (2011) Chronic fluoride toxicity: dental fluorosis. Monographs in oral science 22:81–96. <https://doi.org/10.1159/000327028>

DenBesten P.K., Thariani H. (1992) Biological mechanisms of fluorosis and level and timing of systemic exposure to fluoride with respect to fluorosis. Journal of dental research 71:1238–1243

DenBesten PK, Zhu L, Li W, et al (2011) Fluoride incorporation into apatite crystals delays amelogenin hydrolysis. European journal of oral sciences 119:. <https://doi.org/10.1111/j.1600-0722.2011.00903.x>

Denes L., Bori Z., Csonka E., et al (2008) Reverse regulation of endothelial cells and myointimal hyperplasia on cell proliferation by a heatshock protein-coinducer after hypoxia. Stroke 39:1022–1024. <https://doi.org/10.1161/STROKEAHA.107.495754>

Denis M., Atlan A., Tirlet G., et al (2013) Taches blanches de l’email : diagnostic et anatomopathologie : deux donnees indispensables pour bien les traiter (partie 1)White defects on enamel: Diagnosis and anatomopathology: Two essential factors for proper treatment (part 1). International Orthodontics 11:139–165. <https://doi.org/10.1007/s10856-008-3441-2>

Denis M, Atlan A, Vennat E, et al (2013) White defects on enamel: diagnosis and anatomopathology: two essential factors for proper treatment (part 1). International orthodontics 11:139–165. <https://doi.org/10.1016/j.ortho.2013.02.014>

Densiri-agsorn W, Vitwatanatipa N, Santiwong P, et al Bisphenol-A, Dioxins and Orthodontics

Dent EAP (2015) R. Krishnan, M. Ramesh & P. Chalakkal. Eur Arch Paediatr Dent 16:455–460

Dent EAP PK Musale & S. Kothare

Dent EAP R. Steffen, N. Krämer & K. Bekes

Dent JP (2013) Correction to: Periodontal care in general practice: 20 important FAQs–Part two. children 14:323–327

Dentists AP (2016) Postrzeganie i świadomość hipomineralizacji trzonowcowo-siekaczowej (MIH) wśród polskich lekarzy dentystów. Dent Med Probl 53:382–393

Department of Oral and Maxillofacial Surgery TDC and H Chennai, Tamil Nadu, India, Aarthi Nisha V., Asokan G.S., et al (2014) Oral manifestations in a renal osteodystrophy patient - A case report with review of literature. Journal of Clinical and Diagnostic Research 8:. <https://doi.org/10.7860/JCDR/2014/8879.4722>

DeRocher KA, Smeets PJM, Goodge BH, et al (2020) Chemical gradients in human enamel crystallites. Nature 583:66–71. <https://doi.org/10.1038/s41586-020-2433-3>

DeSantis LC (2015) Options for Treating Teeth Affected with Developmental Defects of Enamel A survey of dentists and dental hygienists in Ohio

Deshpande AN, Joshi NH, Pradhan NR, Raol RY (2017) Microabrasion-remineralization (MAb-Re): An innovative approach for dental fluorosis. Journal of the Indian Society of Pedodontics and Preventive Dentistry 35:384–387. <https://doi.org/10.4103/JISPPD.JISPPD_216_16>

Devi R, Dixit J (2016) Clinical evaluation of insulin like growth factor-i and vascular endothelial growth factor with alloplastic bone graft material in the management of human two wall intra-osseous defects. Journal of clinical and diagnostic research 10:ZC41‐ZC46. <https://doi.org/10.7860/JCDR/2016/21333.8476>

Devireddy SK, Senthil Murugan M, Kishore Kumar RV, et al (2015) Evaluation of Non-vascular Fibula Graft for Mandibular Reconstruction. Journal of maxillofacial and oral surgery 14:299–307. <https://doi.org/10.1007/s12663-014-0657-1>

Dharmani C.K.K. (2018) Management of children with special health care needs (SHCN) in the dental office. JMS - Journal of Medical Society 32:1–6. <https://doi.org/10.4103/jms.jms_115_16>

Di Domenico G.L., Loddo E., Guglielmi D. (2019) Trattamento di recessioni singole associate alla presenza di nccl con approccio conservativo-parodontaleTreatment of single gingival recessions associated with a nccl with a restorative-periodontal approach. Dental Cadmos 87:441–449. <https://doi.org/10.19256/d.cadmos.07.2019.07>

Di Tullio M, Femminella B, Pilloni A, et al (2013) Treatment of supra-alveolar-type defects by a simplified papilla preservation technique for access flap surgery with or without enamel matrix proteins. Journal of periodontology 84:1100‐1110. <https://doi.org/10.1902/jop.2012.120075>

Diedrich P., Fritz U., Kinzinger G., Angelakis J. (2003) Die bewegung von parodontal geschadigten Zahnen nach guided tissue regeneration (GTR) - Eine tierexperimentelle pilotstudieMovement of periodontally affected teeth after guided tissue regeneration (GTR) - An experimental pilot study in animals. Journal of Orofacial Orthopedics 64:214–227. <https://doi.org/10.1007/s00056-003-0240-8>

Diedrich P, Fritz U, Kinzinger G, Angelakis J (2003) Movement of periodontally affected teeth after guided tissue regeneration (GTR)--an experimental pilot study in animals. Journal of orofacial orthopedics = Fortschritte der Kieferorthopadie : Organ/official journal Deutsche Gesellschaft fur Kieferorthopadie 64:214–227

Diemert D, Lobato L, Styczynski A, et al (2017) A Comparison of the Quality of Informed Consent for Clinical Trials of an Experimental Hookworm Vaccine Conducted in Developed and Developing Countries. Plos neglected tropical diseases 11:. <https://doi.org/10.1371/journal.pntd.0005327>

Dieterich M., Schroter V., Stubert J., et al (2019) Oncologic Outcome of Patients with (Low-Risk) Endometrial Carcinoma Undergoing Laparotomy versus Minimally Invasive Hysterectomy: A Retrospective Analysis. Oncology Research and Treatment 42:636–648. <https://doi.org/10.1159/000502757>

Dietschi D. (1995) Free-hand composite resin restorations: a key to anterior aesthetics. Practical periodontics and aesthetic dentistry : PPAD 7:

Dietschi D, Ardu S, Krejci I (2006) A new shading concept based on natural tooth color applied to direct composite restorations. Quintessence international (Berlin, Germany : 1985) 37:91–102

Dietschi D, Devigus A (2011) Prefabricated composite veneers: historical perspectives, indications and clinical application. The European journal of esthetic dentistry : official journal of the European Academy of Esthetic Dentistry 6:178–187

Dietschi D, Herzfeld D (1998) In vitro evaluation of marginal and internal adaptation of class II resin composite restorations after thermal and occlusal stressing. European journal of oral sciences 106:1033–1042

Dietschi D, Monasevic M, Krejci I, Davidson C (2002) Marginal and internal adaptation of class II restorations after immediate or delayed composite placement. Journal of dentistry 30:259‐269. <https://doi.org/10.1016/s0300-5712(02)00041-6>

Dilsiz A, Aydın T, Emrem G (2010a) Effects of the combined desensitizing dentifrice and diode laser therapy in the treatment of desensitization of teeth with gingival recession. Photomedicine and laser surgery 28:S-69

Dilsiz A, Canakci V, Aydin T (2010b) The combined use of Nd: YAG laser and enamel matrix proteins in the treatment of periodontal infrabony defects. Journal of periodontology 81:1411‐1418. <https://doi.org/10.1902/jop.2010.100031>

Dimova C, Papakoca K, Ristoska S, Kovacevska I (2012) Treatment modalities of palatal impacted canines

Dinçer B, Hazar S, Sen BH (2002) Scanning electron microscope study of the effects of soft drinks on etched and sealed enamel. American journal of orthodontics and dentofacial orthopedics : official publication of the American Association of Orthodontists, its constituent societies, and the American Board of Orthodontics 122:135–141

Dincer E. (2008) Why do I have white spots on my front teeth? The New York state dental journal 74:58–60

Diner H., Chou M.D., Sobel E.H. (1978) The effects of neonatal stunting on the development of rats: Effects of early single dose cortisone on dental development and maturation of nursling and mature rats. Pediatric Research 12:948–951

Dini EL, Holt RD, Bedi R (2000) Prevalence of caries and developmental defects of enamel in 9-10 year old children living in areas in Brazil with differing water fluoride histories. British dental journal 188:146–149

Diniz M.B., Coldebella C.R., Zuanon A.C.C., Cordeiro R.C.L. (2011) Alteracoes orais em criancas prematuras e de baixo peso ao nascer: A importancia da relacao entre pediatras e odontopediatrasOral abnormalities in preterm and low birth weight infants: The importance of the relationship between pediatricians and pediatric dentists. Revista Paulista de Pediatria 29:449–455

Diniz-Santos D.R., Silva L.R., Silva N. (2006) Antibiotics for the empirical treatment of acute infectious diarrhea in children. Brazilian Journal of Infectious Diseases 10:217–227. <https://doi.org/10.1590/S1413-86702006000300011>

Dioguardi M., Troiano G., Giannatempo G., et al (2016) Oral manifestations in chronic uremia patients. Renal Failure 38:1–6. <https://doi.org/10.3109/0886022X.2015.1103639>

Discepolo KE, Baker S (2011) Adjuncts to traditional local anesthesia techniques in instance of hypomineralized teeth. The New York state dental journal 77:22–27

Ditto D, Ajoor T, Al-Khashram M, et al Journal of the Bahrain Medical Society

Divaris K, Preisser J, Slade G (2013) Surface-specific efficacy of fluoride varnish in caries prevention in the primary dentition: results of a community randomized clinical trial. Caries research 47:78‐87. <https://doi.org/10.1159/000344015>

Divine RD, Rankin SA (2013) Short communication: Reducing agents attenuate methylglyoxal-based browning in Parmesan cheese. Journal of dairy science 96:6242–6247. <https://doi.org/10.3168/jds.2013-6890>

Divyapriya G, Yavagal PC, Veeresh D (2016) Casein phosphopeptide-amorphous calcium phosphate in dentistry: An update. International Journal of Oral Health Sciences 6:18

Dixit UB, Joshi AV (2018) Efficacy of Intraosseous Local Anesthesia for Restorative Procedures in Molar Incisor Hypomineralization-Affected Teeth in Children. Contemporary clinical dentistry 9:S272. <https://doi.org/10.4103/ccd.ccd_252_18>

Dixon DA (1968) Defects of structure and formation of the teeth in persons with cleft palate and the effect of reparative surgery on the dental tissues. Oral surgery, oral medicine, and oral pathology 25:435–446

Doertbudak O, Durstberger G, Bernhart T, Haas R (2000) Treatment of periodontal defects with an enamel matrix derivative (Emdogain). Journal of clinical periodontology 27:

Doméjean-Orliaguet S, Gansky SA, Featherstone JD (2006) Caries risk assessment in an educational environment. Journal of dental education 70:1346–1354

Dominguez Torres A (2018) Hipomineralização Incisivo-Molar

Domsa E.-M., Para I., Berindan-Neagoe I., et al (2020) Celiac disease: A multi-faceted medical condition. Journal of Physiology and Pharmacology 71:1–12. <https://doi.org/10.26402/jpp.2020.1.01>

Donly KJ (1990) Posterior composite resin: use for anterior restorations. ASDC journal of dentistry for children 57:260–262

Donly KJ, Liu JA (2016) Restoration of Pulp-Treated Teeth. In: Pediatric Endodontics. Springer, pp 103–116

Donos N., Glavind L., Karring T., et al (2003) Wound healing of degree III furcation involvements following guided tissue regeneration and/or Emdogain: A histologic study. Journal of Clinical Periodontology 30:1061–1068. <https://doi.org/10.1046/j.0303-6979.2003.00429.x>

Donos N, Glavind L, Karring T, Sculean A (2004) Clinical evaluation of an enamel matrix derivative and a bioresorbable membrane in the treatment of degree III mandibular furcation involvement: a series of nine patients. International journal of periodontics & restorative dentistry 24:362‐369

Donos N., Lang N.P., Karoussis I.K., et al (2004) Effect of GBR in combination with deproteinized bovine bone mineral and/or enamel matrix proteins on the healing of critical-size defects. Clinical oral implants research 15:101–111

Doolan BJ, Cranwell WC, Nicolopoulos J, Dolianitis C (2019) Secondary organizing pneumonia (bronchiolitis obliterans with organizing pneumonia) associated with adalimumab for treatment of chronic plaque psoriasis. Dermatology online journal 25:

Dori F (2009) [Effect of combined therapeutic methods on healing of periodontal vertical bone defects in regenerative surgery]. Orvosi hetilap 150:517–522. <https://doi.org/10.1556/OH.2009.28500>

Döri F, Arweiler N, Gera I, Sculean A (2005) Clinical evaluation of an enamel matrix protein derivative combined with either a natural bone mineral or beta-tricalcium phosphate. Journal of periodontology 76:2236‐2243. <https://doi.org/10.1902/jop.2005.76.12.2236>

Döri F, Arweiler N, Húszár T, et al (2013a) Five-year results evaluating the effects of platelet-rich plasma on the healing of intrabony defects treated with enamel matrix derivative and natural bone mineral. Journal of periodontology 84:1546‐1555. <https://doi.org/10.1902/jop.2013.120501>

Döri F, Arweiler N, Szántó E, et al (2013b) Ten-year results following treatment of intrabony defects with an enamel matrix protein derivative combined with either a natural bone mineral or a β-tricalcium phosphate. Journal of periodontology 84:749‐757. <https://doi.org/10.1902/jop.2012.120238>

Döri F, Nikolidakis D, Húszár T, et al (2008) Effect of platelet-rich plasma on the healing of intrabony defects treated with an enamel matrix protein derivative and a natural bone mineral. Journal of clinical periodontology 35:44‐50. <https://doi.org/10.1111/j.1600-051X.2007.01161.x>

dos Anjos B, Novaes A, Meffert R, Barboza E (1998) Clinical comparison of cellulose and expanded polytetrafluoroethylene membranes in the treatment of class II furcations in mandibular molars with 6-month re-entry. Journal of periodontology 69:454‐459. <https://doi.org/10.1902/jop.1998.69.4.454>

dos Santos CT, Picini C, Czlusniak GD, Alves FBT (2014) Anomalias do esmalte dentário-revisão de literatura. Archives of Health Investigation 3:

DOS SANTOS RF, MARINHO SA, AGRIPINO GG, et al (2015) Focal Epithelial Hyperplasia: A Case Report. Oral Surgery, Oral Medicine, Oral Pathology and Oral Radiology 120:e80

Dougall A, Fiske J (2008) Access to special care dentistry, part 6. Special care dentistry services for young people. British dental journal 205:235–249. <https://doi.org/10.1038/sj.bdj.2008.734>

Downer MC (1987) Craniofacial anomalies--are they a public health problem? International dental journal 37:193–196

Downer M.C. (1995) The 1993 national survey of children’s dental health. British dental journal 178:407–412

Downer M.C., Blinkhorn A.S., Holt R.D., et al (1994) Dental caries experience and defects of dental enamel among 12-year-old children in north London, Edinburgh, Glasgow and Dublin. Community dentistry and oral epidemiology 22:283–285

Drawanz Hartwig A, Sousa Azevedo M, Anschau Pauli L, et al (2019) An observational study of dental abnormalities in the primary teeth. RSBO: Revista Sul-Brasileira de Odontologia 16:

Dreano E., Hatton A., Vignaud L., et al (2019) Characterization of two rat models of cystic fibrosis: F508del and KO CFTR generated by CRISPR-Cas9. Pediatric Pulmonology 54:318–319. <https://doi.org/10.1002/ppul.22495>

Drew R.H. (2007) Emerging options for treatment of invasive, multidrug-resistant Staphylococcus aureus infections. Pharmacotherapy 27:227–249. <https://doi.org/10.1592/phco.27.2.227>

DRKS00005600 (2013) Oral Health of patients with stem cell transplantation in childhood. http://www.who.int/trialsearch/Trial2.aspx?TrialID=DRKS00005600

DRKS00006841 (2015) Maintenance of oral health in preterm children by preventive oral care. http://www.who.int/trialsearch/Trial2.aspx?TrialID=DRKS00006841

DRKS00009760 (2016) Randomized clinical study on the infiltration of enamel mineralization disorders. http://www.who.int/trialsearch/Trial2.aspx?TrialID=DRKS00009760

DRKS00010465 (2016) Clinical trial for masking of dental fluorosis by infiltration. http://www.who.int/trialsearch/Trial2.aspx?TrialID=DRKS00010465

DRKS00011882 (2017) The impact of molar-incisor hypomineralization (MIH) on the child’s oral health-related quality of life in children before and after dental management. http://www.who.int/trialsearch/Trial2.aspx?TrialID=DRKS00011882

DRKS00018845 (2019) Vitalamputation of maxillary molars with furcation involvement degree II and / or III - a randomized clinically controlled study. http://www.who.int/trialsearch/Trial2.aspx?TrialID=DRKS00018845

DRKS00020359 (2019) Efficacy of desensitizing toothpastes containing hydroxylapatite vs fluoride for hypersensitivity relief in MIH (Molar-Incisor-Hypomineralisation)-affected molars in children and adolescents. http://www.who.int/trialsearch/Trial2.aspx?TrialID=DRKS00020359

Drum MA, Kaiser-Kupfer MI, Guckes AD, Roberts MW (1985) Oral manifestations of the Rieger syndrome: report of case. Journal of the American Dental Association (1939) 110:343–346

Drummond BK, Harding W (2014) Examination and Treatment Planning for Hypomineralized. Planning and Care for Children and Adolescents with Dental Enamel Defects: Etiology, Research and Contemporary Management 99

Drummond BK, Harding W (2015) Examination and treatment planning for hypomineralized and/or hypoplastic teeth. In: Planning and Care for Children and Adolescents with Dental Enamel Defects. Springer, pp 99–112

Drummond BK, Kilpatrick N (2014) 13 Final Comments. Planning and Care for Children and Adolescents with Dental Enamel Defects: Etiology, Research and Contemporary Management 169

Drummond BK, Kilpatrick N (2015) Final Comments. In: Planning and Care for Children and Adolescents with Dental Enamel Defects. Springer, pp 169–170

Du J-K, Wu J-H, Chen P-H, et al (2020) Influence of cavity depth and restoration of non-carious cervical root lesions on strain distribution from various loading sites. BMC oral health 20:98. <https://doi.org/10.1186/s12903-020-01083-w>

Duarte A, Santos M, Cordeiro A (2018) Nintedanib-A new oportunity for systemic sclerosis patients? Journal of scleroderma and related disorders 3:267‐268. <https://doi.org/10.1177/2397198317753493>

Duggal MS (2003) Paediatric dentistry in the new millennium: I. Quality care for children. Dental update 30:230–234

Dujmović V (2019) Etiologija i terapija molarno incizivne hipomineralizacije

Dukic W., Delija B., Lesic S., et al (2013) Radiopacity of flowable composite by a digital technique. Operative Dentistry 38:299–308. <https://doi.org/10.2341/12.166-L>

Dummer PM, Kingdon A, Kingdon R (1990) Prevalence and distribution by tooth type and surface of developmental defects of dental enamel in a group of 15- to 16-year-old children in South Wales. Community dental health 7:369–377

Duncan WK, Silberman SL, Trubman A (1988) Labial hypoplasia of primary canines in black Head Start children. ASDC journal of dentistry for children 55:423–426

Dunkel L, Quinton R (2014) Transition in endocrinology: induction of puberty. European journal of endocrinology 170:R229. <https://doi.org/10.1530/EJE-13-0894>

Durmus B., Abbasoglu Z., Peker S., Kargul B. (2013) Moguci medicinski etioloski cimbenici i znacajke molarno incizivne hipomineralizacije u skupini turske djecePossible medical aetiological factors and characteristics of molar incisor hypomineralisation in a group of turkish children. Acta Stomatologica Croatica 47:297–305. <https://doi.org/10.15644/asc47/4/1>

Dursun E, Savard E, Vargas C, et al Management of Amelogenesis Imperfecta: A 15-Year Case History of Two Siblings. Operative dentistry 41:567–577

Duval B, Maynard J, Gunsolley J, Waldrop T (2000) Treatment of human mucogingival defects utilizing a bioabsorbable membrane with and without a demineralized freeze-dried bone allograft. Journal of periodontology 71:1687‐1692. <https://doi.org/10.1902/jop.2000.71.11.1687>

Eastman J, Raibley S, Schwartz LJ (1979) An esthestic management of the amelogenesis imperfecta patient. Illinois dental journal 48:379–381

Eaton K (2008) Book Review: Critical Thinking: Understanding and Evaluating Dental Research

Ebel M, Bekes K, Klode C, Hirsch C (2018) The severity and degree of hypomineralisation in teeth and its influence on oral hygiene and caries prevalence in children. International journal of paediatric dentistry 28:648–657. <https://doi.org/10.1111/ipd.12425>

Echeverría S, Sassi JC, Simunović V (1974) [Restoration with composite in fractures and hypoplasia of young permanent teeth]. Odontologia chilena 22:24–25

Echeverria S., Sassi J.C., Simunovic V. (1974) Restauraciones con composite en fracturas e hipoplasias de piezas permanentes jovenesRestoration with composite in fractures and hypoplasia of young permanent teeth. Odontologia chilena 22:24–25

Eckstein M., Lacruz R.S. (2018) CRAC channels in dental enamel cells. Cell Calcium 75:14–20. <https://doi.org/10.1016/j.ceca.2018.07.012>

Edds A.C., Walden J.E., Scheetz J.P., et al (2005) Pilot study of correlation of pulp stones with cardiovascular disease. Journal of Endodontics 31:504–506. <https://doi.org/10.1097/01.don.0000168890.42903.2b>

Edmeads JG, Gawel MJ, Vickers J (1997) Strategies for diagnosing and managing medication-induced headache. Canadian family physician Medecin de famille canadien 43:1249–1254

Edward S, Nord CE (1974) A case of amelogenesis imperfecta. Svensk tandlakare tidskrift Swedish dental journal 67:229–237

Effinger K.E., Migliorati C.A., Hudson M.M., et al (2014) Oral and dental late effects in survivors of childhood cancer: A Children’s Oncology Group report. Supportive Care in Cancer 22:2009–2019. <https://doi.org/10.1007/s00520-014-2260-x>

Eichenberger M, Erb J, Zwahlen M, Schätzle M (2015) The timing of extraction of non-restorable first permanent molars: a systematic review Introduction. Eur J Paediatr Dent 16:8–272

Eichner K. (1982) Lucken, Zahnwanderung und Versorgung im FrontzahngebietGaps, tooth migration and treatment in the anterior dentition. Deutsche zahnarztliche Zeitschrift 37:223–226

Eickholz P., Benn D.K., Staehle H.J. (1996) Radiographic evaluation of bone regeneration following periodontal surgery with or without expanded polytetrafluoroethylene barriers. Journal of periodontology 67:379–385

Eickholz P, Hörr T, Klein F, et al (2004) Radiographic parameters for prognosis of periodontal healing of infrabony defects: two different definitions of defect depth. Journal of periodontology 75:399–407

Eickholz P, Kim T, Holle R (1998a) Regenerative periodontal surgery with non-resorbable and biodegradable barriers: results after 24 months. Journal of clinical periodontology 25:666‐676. <https://doi.org/10.1111/j.1600-051x.1998.tb02504.x>

Eickholz P., Kim T.S., Holle R. (1997) Guided tissue regeneration with non-resorbable and biodegradable barriers: 6 months results. Journal of clinical periodontology 24:92–101

Eickholz P., Krigar D.-M., Kim T.-S., et al (2007) Stability of clinical and radiographic results after guided tissue regeneration on infrabony defects. Journal of Periodontology 78:37–46. <https://doi.org/10.1902/jop.2007.060097>

Eickholz P, Lenhard M, Benn D, Staehle H (1998b) Periodontal surgery of vertical bony defects with or without synthetic bioabsorbable barriers. 12-month results. Journal of periodontology 69:1210‐1217. <https://doi.org/10.1902/jop.1998.69.11.1210>

Eickholz P, Röllke L, Schacher B, et al (2014) Enamel matrix derivative in propylene glycol alginate for treatment of infrabony defects with or without systemic doxycycline: 12- and 24-month results. Journal of periodontology 85:669‐675. <https://doi.org/10.1902/jop.2013.130290>

Ekambaram M, Anthonappa RP, Govindool SR, Yiu CKY (2017) Comparison of deproteinization agents on bonding to developmentally hypomineralized enamel. Journal of dentistry 67:94–101. <https://doi.org/10.1016/j.jdent.2017.10.004>

Ekambaram M, Yiu C (2016) Bonding to hypomineralized enamel–A systematic review. International Journal of Adhesion and Adhesives 69:27–32

Ekanayake L, van der Hoek W (2003) Prevalence and distribution of enamel defects and dental caries in a region with different concentrations of fluoride in drinking water in Sri Lanka. International dental journal 53:243–248

Ekanayake L., van der Hoek W. (2002) Dental caries and developmental defects of enamel in relation to fluoride levels in drinking water in an arid area of Sri Lanka. Caries research 36:398–404

El Haj AJ, Tamone SL, Peake M, et al (1997) An ecdysteroid-responsive gene in a lobster - a potential crustacean member of the steroid hormone receptor superfamily. Gene 201:127–135

EL MELIGY O, ELKHODARY H, SABBAGH H, BAGHER S (2019) THE KINGDOM OF SAUDI ARABIA OMAR EL MELIGY1, 1, 2, HEBA ELKHODARY1, 3, HEBA SABBAGH1 AND SARA BAGHER1 1DEPARTMENT OF PEDIATRIC DENTISTRY, FACULTY OF. A Global Compendium of Oral Health: Tooth Eruption and Hard Dental Tissue Anomalies 350

Elcock C, Lath DL, Luty JD, et al (2006) The new Enamel Defects Index: testing and expansion. European journal of oral sciences 114:

Elfrink M (2014) Kaasmolaren, en dan? Standby 28:4–7

Elfrink M (2012a) SOURCE (OR PART OF THE FOLLOWING SOURCE): Type PhD thesis Title Deciduous molar hypomineralisation, its nature and nurture

Elfrink M (2015) Kaaskiezen in het melkgebit en blijvende gebit. JGZ Tijdschrift voor jeugdgezondheidszorg 47:24–28

Elfrink M, Ghanim A, Manton D, Weerheijm K (2015) Standardised studies on molar incisor hypomineralisation (MIH) and hypomineralised second primary molars (HSPM): a need. European Archives of Paediatric Dentistry 16:247–255

Elfrink M, Ten Cate J, Jaddoe V, et al (2012a) Deciduous molar hypomineralization and molar incisor hypomineralization. Journal of dental research 91:551–555

Elfrink M, ten Cate J, van Ruijven L, Veerkamp J (2012b) MicroCt study on Deciduous Molar Hypomineralisation. Deciduous Molar Hypomineralisation, its nature and nurture 69

Elfrink M, Weerheijm K (2012) Molaren Inzisiven Hypomineralisation und Milchmolaren Hypomineralisation—Klinisches Erscheinungsbild, Prävalenz und deren Ursachen. Oralprophylaxe und Kinderzahnheilkunde 34:166

Elfrink ME, Moll HA (2013) Jessica C. Kiefte-de Jong, Hanan El Marroun, Vincent WV Jaddoe, Albert Hofman, et al. Drug Saf 36:627–633

Elfrink ME, Moll HA, Kiefte-de Jong JC, et al (2013a) Is maternal use of medicines during pregnancy associated with deciduous molar hypomineralisation in the offspring? A prospective, population-based study. Drug safety 36:627–633

Elfrink ME, Weerheijm KL (2020) Hypomineralized Second Primary Molars. In: Molar Incisor Hypomineralization. Springer, pp 71–85

Elfrink MEC (2012b) Deciduous molar hypomineralisation, its nature and nurture

Elfrink MEC, Schuller AA, Veerkamp JSJ, et al (2010) Factors increasing the caries risk of second primary molars in 5-year-old Dutch children. International journal of paediatric dentistry 20:151–157. <https://doi.org/10.1111/j.1365-263X.2009.01026.x>

Elfrink MEC, ten Cate JM, van Ruijven LJ, Veerkamp JSJ (2013b) Mineral content in teeth with deciduous molar hypomineralisation (DMH). Journal of dentistry 41:974–978. <https://doi.org/10.1016/j.jdent.2013.08.024>

Elger W, Illge C, Kiess W, et al (2020) Relationship between deciduous molar hypomineralisation and parameters of bone metabolism in preschool children. International Dental Journal

Elhennawy K, Jost-Brinkmann P-G, Manton DJ, et al (2017a) Managing molars with severe molar-incisor hypomineralization: A cost-effectiveness analysis within German healthcare. Journal of dentistry 63:65–71

Elhennawy K, Jost-Brinkmann P-G, Zaslansky P, et al (2019a) Was wissen wir über MIH-Schmelz? Eine systematische Literaturübersicht

Elhennawy K, Krois J, Jost-Brinkmann P-G, Schwendicke F (2019b) Outcome and comparator choice in molar incisor hypomineralisation (MIH) intervention studies: a systematic review and social network analysis. BMJ open 9:e028352

Elhennawy K, Manton DJ, Crombie F, et al (2017b) Structural, mechanical and chemical evaluation of molar-incisor hypomineralization-affected enamel: A systematic review. Archives of oral biology 83:272–281

Elhennawy K., Reda S., Finke C., et al (2017) Oral manifestations, dental management, and a rare homozygous mutation of the PRDM12 gene in a boy with hereditary sensory and autonomic neuropathy type VIII: A case report and review of the literature. Journal of Medical Case Reports 11:. <https://doi.org/10.1186/s13256-017-1387-z>

Elhennawy K, Schwendicke F (2016) Managing molar-incisor hypomineralization: A systematic review. Journal of dentistry 55:16–24

Elhussein M, Jamal H (2020) Molar Incisor Hypomineralisation-To Extract or to Restore beyond the Optimal Age? Children (Basel, Switzerland) 7:. <https://doi.org/10.3390/children7080091>

Ellakwa A.E., Shortall A.C., Burke F.J., Marquis P.M. (2003) Effects of grit blasting and silanization on bond strengths of a resin luting cement to Belleglass HP indirect composite. American journal of dentistry 16:53–57

Ellwood R, O’Mullane D, Clarkson J, Driscoll W (1994) A comparison of information recorded using the Thylstrup Fejerskov index, Tooth Surface Index of Fluorosis and Developmental Defects of Enamel index. International dental journal 44:628–636

Ellwood R.P., Cortea D.F., O’Mullane D.M. (1996) A photographic study of developmental defects of enamel in Brazilian school children. International dental journal 46:69–75

Ellwood RP, O’Mullane D (1995a) Enamel opacities and dental esthetics. Journal of public health dentistry 55:171–176

Ellwood RP, O’Mullane D (1996) The association between developmental enamel defects and caries in populations with and without fluoride in their drinking water. Journal of public health dentistry 56:76–80

Ellwood RP, O’Mullane DM (1994) Tooth brushing behaviour in twelve year old children and dental enamel. Journal of the Irish Dental Association 40:12–15

Ellwood RP, O’Mullane DM (1995b) Dental enamel opacities in three groups with varying levels of fluoride in their drinking water. Caries research 29:137–142

Ellwood R.P., O’Mullane D.M. (1994a) Association between dental enamel opacities and dental caries in a north Wales population. Caries research 28:383–387

Ellwood R.P., O’Mullane D.M. (1994b) The demographic and social variation in the prevalence of dental enamel opacities in north Wales. Community dental health 11:192–196

El-Sayed W, Shore RC, Parry DA, et al (2011) Hypomaturation amelogenesis imperfecta due to WDR72 mutations: a novel mutation and ultrastructural analyses of deciduous teeth. Cells, tissues, organs 194:60–66. <https://doi.org/10.1159/000322036>

Eltair M, Pitchika V, Standl M, et al (2020) Prevalence of traumatic crown injuries in German adolescents. Clinical oral investigations 24:867–874

Elzein R, Chouery E, Abdel-Sater F, et al (2019) Molar incisor hypomineralisation in Lebanon: prevalence and clinical characteristics. European Archives of Paediatric Dentistry 1–8

Emil T, Yavor K, Ilia N, Atanas B (2015) Dental incrustation-a survey. Научни трудове на Съюза на учените–Пловдив Серия Г: Медицина, фармация и дентална медицина 18:

Eminkahyagil N., Korkmaz Y., Gokalp S., Baseren M. (2005) Shear bond strength of orthodontic brackets with newly developed antibacterial self-etch adhesive. Angle Orthodontist 75:843–848

Eminkahyagil N, Korkmaz Y, Gokalp S, Baseren M (2005) Shear bond strength of orthodontic brackets with newly developed antibacterial self-etch adhesive. The Angle orthodontist 75:843–848

Emmatty TB, Eby A, Joseph MJ, et al (2020) The prevalence of molar incisor hypomineralization of school children in and around Muvattupuzha, Kerala. Journal of the Indian Society of Pedodontics and Preventive Dentistry 38:14–19. <https://doi.org/10.4103/JISPPD.JISPPD_152_18>

en Odontología L (2018) FACULTAD DE MEDICINA ESCUELA DE ODONTOLOGÍA

Engstrom C, Noren JG (1986) Effects of orthodontic force on enamel formation in normal and hypocalcemic rats. Journal of oral pathology 15:78–82

Enomoto N, Chida K, Suda T, et al (2016) An exploratory trial of intravenous immunoglobulin therapy for idiopathic pulmonary fibrosis: a preliminary multicenter report. Clinical respiratory journal 10:746‐755. <https://doi.org/10.1111/crj.12281>

Enzenauer R, Freeman H, Larson M, Williams T (2000) Photoscreening for amblyogenic factors by public health personnel: the Eyecor Camera System. Ophthalmic epidemiology 7:1‐12

Epasinghe DJ, Yiu CKY (2018) Effect of etching on bonding of a self-etch adhesive to dentine affected by amelogenesis imperfecta. Journal of investigative and clinical dentistry 9:. <https://doi.org/10.1111/jicd.12276>

Erdem GB, Uzamiş M, Olmez S, Sargon MF (2001) Primary incisor triplication defect. ASDC journal of dentistry for children 68:301

Ergun G, Kaya BM, Egilmez F, Cekic-Nagas I (2013) Functional and esthetic rehabilitation of a patient with amelogenesis imperfecta. Journal (Canadian Dental Association) 79:d38

Erika V, Modrić, Verzak Ž, Karlović Z (2016) Developmental Defects of Enamel in Children with Intellectual Disability. Acta stomatologica Croatica 50:65–71. <https://doi.org/10.15644/asc50/1/9>

Ermis R, Temel U, Cellik E, Kam O (2010) Clinical performance of a two-step self-etch adhesive with additional enamel etching in Class III cavities. Operative dentistry 35:147‐155. <https://doi.org/10.2341/09-089-C>

Ermis R, Van Landuyt K, Cardoso M, et al (2012) Clinical effectiveness of a one-step self-etch adhesive in non-carious cervical lesions at 2 years. Clinical oral investigations 16:889‐897. <https://doi.org/10.1007/s00784-011-0565-4>

Ernst CP, Weckmüller C, Willershausen B (1995) [Deciduous tooth reconstruction with composite polymers. The ITN care of amelogenesis imperfecta (first dentition) with composite polymeric materials as well as steel crowns--a case report]. Schweizer Monatsschrift fur Zahnmedizin = Revue mensuelle suisse d’odonto-stomatologie = Rivista mensile svizzera di odontologia e stomatologia 105:664–671

Ertas U, Ataol M, Kiki A, Uğurlu M (2020) Orthognathic surgery with two-segment le fort i and sagittal split ramus osteotomies of open bite deformity in an amelogenesis imperfecta patient via virtual planning: A case report. Nigerian journal of clinical practice 23:577–580. <https://doi.org/10.4103/njcp.njcp_316_19>

Ertugrul F., Elbek-Cubukcu C., Sabah E., Mir S. (2003) The oral health status of children undergoing hemodialysis treatment. Turkish Journal of Pediatrics 45:108–113

Esan T TA Oyedele, MO Folayan, CA Adekoya-Sofowora, EO Oziegbe

Escalante H., Castro L., Amaya M.P., et al (2018) Anaerobic digestion of cheese whey: Energetic and nutritional potential for the dairy sector in developing countries. Waste Management 71:711–718. <https://doi.org/10.1016/j.wasman.2017.09.026>

Esposito M, Grusovin M, Papanikolaou N, et al (2009) Enamel matrix derivative (Emdogain®) for periodontal tissue regeneration in intrabony defects. Cochrane Database of Systematic Reviews. <https://doi.org/10.1002/14651858.CD003875.pub3>

Eton D., Borhani M., Spero K., et al (1995) Photodynamic therapy: Cytotoxicity of aluminum phthalocyanine on intimal hyperplasia. Archives of Surgery 130:1098–1103

EUCTR2008-004405-34-GB (2009) ARTEMIS-IPF: a Phase 3, Randomized, Double Blind, Placebo Controlled, Multi Center, Parallel Group, Event Driven Study to Evaluate the Efficacy and Safety of Ambrisentan in Subjects with Early Idiopathic Pulmonary Fibrosis (IPF) - ARTEMIS-IPF. http://www.who.int/trialsearch/Trial2.aspx?TrialID=EUCTR2008-004405-34-GB

EUCTR2009-011169-98-IT (2009) A Phase 3, Randomized, Double Blind, Placebo Controlled, Multi Center, Parallel Group Study to Evaluate the Efficacy and Safety of Ambrisentan in Subjects with Idiopathic Pulmonary Fibrosis and Pulmonary Hypertension - ND. http://www.who.int/trialsearch/Trial2.aspx?TrialID=EUCTR2009-011169-98-IT

EUCTR2011-002784-24-GB (2013) Strong Treat 1 to 4. http://www.who.int/trialsearch/Trial2.aspx?TrialID=EUCTR2011-002784-24-GB

EUCTR2014-004782-24-NL (2014) A study to test intravenous PRM-151 for safety and to see how PRM-151 acts in the body and blood of people with idiopathic pulmonary fibrosis (IPF) - a disorder where lung tissue becomes damaged and scarred making it difficult to breathe. http://www.who.int/trialsearch/Trial2.aspx?TrialID=EUCTR2014-004782-24-NL

EUCTR2016-000600-29-IE (2016) A Randomized, Open-Label, Phase 3 Study to Assess the Efficacy and Safety of KRN23 Versus Oral Phosphate and Active Vitamin D Treatment in Pediatric Patients with X-linked Hypophosphatemia (XLH). http://www.who.int/trialsearch/Trial2.aspx?TrialID=EUCTR2016-000600-29-IE

EUCTR2018-002632-24-FR (2018) to evaluate a triple therapy: plasma exchange, rituximab, intravenous immunoglobulin (IVIg) and corticosteroid administration compared to standard corticosteroid therapy in patients for severe acute exacerbation of idiopathic pulmonary. http://www.who.int/trialsearch/Trial2.aspx?TrialID=EUCTR2018-002632-24-FR

Evans R.W., Beck D.J., Brown R.H. (1980) Dental health of 5-year-old children: A report from the Dunedin multidisciplinary child development study. New Zealand Dental Journal 76:179–186

Facchinetti F., Martignoni E., Gallai V., et al (1988) Neuroendocrine evaluation of central opiate activity in primary headache disorders. Pain 34:29–33. <https://doi.org/10.1016/0304-3959%2888%2990178-9>

Fadeyibi I.O., Ademiluyi S., Sorunke M.E., et al (2011) Oral health status of individuals with cleft lip, cleft palate or both in a Nigerian population. Macedonian Journal of Medical Sciences 4:265–270. <https://doi.org/10.3889/MJMS.1857-5773.2011.0175>

Fagenson A.M., Karhadkar S.S., Di Carlo A., et al (2020) 1056 MINIMALLY INVASIVE HEPATECTOMY IN NORTH AMERICA: LAPAROSCOPIC VERSUS ROBOTIC. Gastroenterology 158:1551. <https://doi.org/10.1016/S0016-5085%2820%2934557-1>

Faggella A, Guadagni MG, Cocchi S, et al (2006) Dental features in patients with Turner syndrome. European journal of paediatric dentistry 7:165–168

Fagrell S, Melin N, Svensson H, et al (2001) Swedish Dental Journal. swedish dental journal 25:

Fagrell T. (2011) Molar incisor hypomineralization. Morphological and chemical aspects, onset and possible etiological factors. Swedish dental journal Supplement 5–83

Fagrell TG, Dietz W, Jälevik B, Norén JG (2010) Chemical, mechanical and morphological properties of hypomineralized enamel of permanent first molars. Acta odontologica Scandinavica 68:215–222. <https://doi.org/10.3109/00016351003752395>

Fagrell TG, Lingström P, Olsson S, et al (2008) Bacterial invasion of dentinal tubules beneath apparently intact but hypomineralized enamel in molar teeth with molar incisor hypomineralization. International journal of paediatric dentistry 18:333–340. <https://doi.org/10.1111/j.1365-263X.2007.00908.x>

Fagrell TG, Ludvigsson J, Ullbro C, et al (2011) Aetiology of severe demarcated enamel opacities-an evaluation based on prospective medical and social data from 17,000 children. Swed Dent J 35:57–67

Fagrell T.G., Salmon P., Melin L., Noren J.G. (2013) Onset of Molar Incisor Hypomineralization (MIH). Swedish Dental Journal 37:61–70

Fahl Jr. N. (2007) A polychromatic composite layering approach for solving a complex Class IV/direct veneer/diastema combination: Part II. Practical procedures & aesthetic dentistry : PPAD 19:17–22

Fahl N, Denehy GE, Jackson RD (1998) Protocol for predictable restoration of anterior teeth with composite resins. Oral health 88:15–22

Fairaq MM, Naghi KM, Alshouibi EN (2019) Minimally Invasive Dentistry. INDO AMERICAN JOURNAL OF PHARMACEUTICAL SCIENCES 6:1422–1428

Falcon HC, Richardson P, Shaw MJ, et al (2001) Developing an index of restorative dental treatment need. British dental journal 190:479–486

Falkowska S, Stawiecka M, Milewska R, et al (2019a) Hipomineralizacja trzonowcowo-siekaczowa (MIH)–etiologia, obraz kliniczny, leczenie

Falkowska S, Stawiecka M, Milewska R, et al (2019b) Molar-incisor hypomineralisation (MIH)–aetiology, clinical picture, treatment

Fang X.-X., Fan C.-H., Xu T., et al (2019) Association between cervical vertebral maturation stages and dental calcification stages in patients with unilateral complete cleft lip and palate. Hua xi kou qiang yi xue za zhi = Huaxi kouqiang yixue zazhi = West China journal of stomatology 37:180–186. <https://doi.org/10.7518/hxkq.2019.02.010>

Farah R (2011) The role of blood and serum proteins in the pathogenesis of Molar-Incisor Hypomineralisation

Farah R, Drummond B, Swain M, Williams S (2010a) Linking the clinical presentation of molar-incisor hypomineralisation to its mineral density. International journal of paediatric dentistry 20:353–360. <https://doi.org/10.1111/j.1365-263X.2010.01061.x>

Farah R, Swain M, Drummond B, et al (2010b) Mineral density of hypomineralised enamel. Journal of Dentistry 38:50–58

Farah RA, Drummond BK, Swain MV, Williams S (2008) Relationship between laser fluorescence and enamel hypomineralisation. Journal of dentistry 36:915–921. <https://doi.org/10.1016/j.jdent.2008.07.012>

Faria-e-Silva AL, De Moraes RR, Menezes MDS, et al (2011) Hardness and microshear bond strength to enamel and dentin of permanent teeth with hypocalcified amelogenesis imperfecta. International journal of paediatric dentistry 21:314–320. <https://doi.org/10.1111/j.1365-263X.2011.01129.x>

Farias L, Laureano ICC, de Alencar CRB, Cavalcanti AL (2018) Hipomineralização molar-incisivo: etiologia, características clínicas e tratamento. Revista de Ciências Médicas e Biológicas 17:211–219

Farias L, Laureano ICC, de Alencar CRB, Cavalcanti AL (2019) Analysis of prevalence and diagnostic criteria of molar-incisor hipomineralization. Journal of Oral Research 8:254–262

Farid H., Khan F.R. (2012) Clinical management of severe fluorosis in an adult. BMJ Case Reports. <https://doi.org/10.1136/bcr-2012-007138>

Farina R., Itro A., Ferrieri I., Trombelli L. (2007) Disease recurrence following reconstructive procedures: a 6- to 8-year follow-up observational study. Oral health & preventive dentistry 5:307–312

Farjoudi F., Hupfeld C.J., Chi N.-W. (2018) Digeorge syndrome in a 43-year-old man presenting for diabetes management. Endocrine Reviews 39:

Farmakis E., Puntis J.W., Toumba K.J. (2005) Enamel defects in children with coeliac disease. European journal of paediatric dentistry 6:129–132

Farmer V, Townsend G (1993) Crown size variability in the deciduous dentition of South Australian children. American journal of human biology : the official journal of the Human Biology Council 5:681–690. <https://doi.org/10.1002/ajhb.1310050610>

Fathi F. (2017) Production of knockout mice with FAM83H gene modifications by CRISPR/Cas9 mediated genome engineering. Iranian Journal of Biotechnology 15:77–78. <https://doi.org/10.15171/ijb.2017.s1-334>

Fatturi AL, Menoncin BL, Reyes MT, et al (2020) The relationship between molar incisor hypomineralization, dental caries, socioeconomic factors, and polymorphisms in the vitamin D receptor gene: a population-based study. Clinical oral investigations 1–10

Fatusi OA, Ogunbodede E, Sowole CA, Folayan MO (2018) Gaps in oral health-care service provision systems for children in Nigeria: A case study of a tertiary health institution. Indian Journal of Dental Research 29:622

Faunce F. (1983) Management of discolored teeth. Dental clinics of North America 27:657–670

Faustino-Silva DD, Rocha AF, da Rocha BS, Stein C (2020) Use of Antibiotics in early Childhood and Dental Enamel Defects in 6- to 12-year-old Children in Primary Health Care. Acta odontologica latinoamericana : AOL 33:6–13

Fava M, Frascino AV, Balducci I, Ramos CJ (2018) Comparative analysis of different prophylatic methods on primary teeth enamel roughness. Brazilian Dental Science 21:335–340

Favia G., Lacaita M.G., Limongelli L., et al (2014) Hyperphosphatemic familial tumoral calcinosis: Odontostomatologic management and pathological features. American Journal of Case Reports 15:569–575. <https://doi.org/10.12659/AJCR.892113>

Fayle S, Kandiah P (2012) Treatment of dental caries in the preschool child. Paediatric Dentistry 3:133–146

Fayle SA (2003) Molar incisor hypomineralisation: restorative management. European journal of paediatric dentistry 4:121–126

Fearne J., Anderson P., Davis G.R. (2004) 3D X-ray microscopic study of the extent of variations in enamel density in first permanent molars with idiopathic enamel hypomineralisation. British Dental Journal 196:634–638. <https://doi.org/10.1038/sj.bdj.4811282>

FEDERAL GDD Protocolo de Atenção à Saúde

Feher A., Ogodescu A.S., Luca M.M., et al (2016) The treatment of white spot lesions in pediatric dentistry-infltration with Icon. Clujul Medical 89:

Fei A, Udin R, Johnson R (1991) A clinical study of ferric sulfate as a pulpotomy agent in primary teeth. Pediatric dentistry 13:327‐332

Feierabend S (2014) Behandlungskonzepte bei Strukturanomalien des Zahnschmelzes und des Dentins. Stomatologie 111:341–353

Feierabend S Eine besondere Herausforderung jenseits des Praxisalltags

Feierabend S, Bekes K Der Fall: Verdacht auf Fluorose

Feierabend S, Halbleib K, Klaiber B, Hellwig E (2012) 2. Quintessence international (Berlin, Germany : 1985) 43:305–311

Fejerskov O, Manji F, Baelum V (1990) The nature and mechanisms of dental fluorosis in man. Journal of dental research 69:

Fejerskov O., Yaeger J.A., Thylstrup A. (1979) Microradiography of the effect of acute and chronic administration of fluoride on human and rat dentine and enamel. Archives of Oral Biology 24:123–130. <https://doi.org/10.1016/0003-9969%2879%2990060-8>

Felberg R.A., Burgin W.S., Hickenbottom S.L., et al (2001) Hypothermia after cardiac arrest: Feasibility and safety of an external cooling protocol. Circulation 104:1799–1804. <https://doi.org/10.1161/hc4001.097037>

Fernandes A.S., Mesquita P., Vinhas L. (2012) Hipomineralizacao incisivo-molar: Uma revisao da literaturaMolar-incisor-hypomineralization: A literature review. Revista Portuguesa de Estomatologia, Medicina Dentaria e Cirurgia Maxilofacial 53:258–262. <https://doi.org/10.1016/j.rpemd.2012.07.004>

Fernandes Gomes M., Rodrigues Kohlemann K., Plens G., et al (2005) Oral manifestations during chemotherapy for acute lymphoblastic leukemia: A case report. Quintessence International 36:307–313

Fernandes JMA, Rego ROCC, Spolidorio LC, et al (2005) Enamel matrix proteins associated with GTR and bioactive glass in the treatment of class III furcation in dogs. Brazilian oral research 19:169–175

Fernández CC, Rodríguez AL, Martínez SS, et al Hipomineralización incisivo-molar (MIH). A propósito de un caso.

Fernández M, Marbella D Clinica Dental Elvira Martinez

Fernando de Almeida Barros Mourão C (2019) Autologous platelet concentrates for treating periodontal infrabony defects. Evidence-based dentistry 20:54–55. <https://doi.org/10.1038/s41432-019-0031-8>

Ferrarotti F., Romano F., Quirico A., et al (2018) Effectiveness of Enamel Matrix Derivative in Conjunction with Particulate Autologous Bone in the Treatment of Noncontained Intrabony Defects: A 2-Year Prospective Case Series. The International journal of periodontics & restorative dentistry 38:673–680. <https://doi.org/10.11607/prd.3003>

Ferrazzano G, Sangianantoni G, Cantile T, et al (2012a) Dental enamel defects in Italian children with cystic fibrosis: an observational study. Community Dent Health 29:106–9

Ferrazzano GF, Sangianantoni G, Cantile T, et al (2013) Oral health status in liver transplant Italian children. European journal of paediatric dentistry 14:323–327

Ferrazzano GF, Sangianantoni G, Cantile T, et al (2012b) Dental health in asthmatic children: a South Italy study. Journal of dentistry for children (Chicago, Ill) 79:170–175

Ferreira JCM (2017) Hipomineralização incisivo-molar: Abordagem Terapêutica

Ferreira LT, Paiva E, Ríos H, et al (2005) Hipomineralización incisivo molar: su importancia en Odontopediatría. Odontología Pediátrica, 2005, vol 13, num 2, p 54-59

Ferreira S.-B.-P., Maia N.-G.-F., Martelli-Junior H., et al (2018) Oral findings in Williams-Beuren syndrome. Medicina Oral, Patologia Oral y Cirugia Bucal 23:. <https://doi.org/10.4317/medoral.21834>

Ferring V., Preusser S.E., Wetzel W.-E., Wleklinski C. (2007) Prevalence and severity of molar incisor hypomineralization in a region of Germany - A brief communication. Journal of Public Health Dentistry 67:148–150. <https://doi.org/10.1111/j.1752-7325.2007.00040.x>

Ferrini F, Lorenzo L-E, Godin AG, et al (2017) Enhancing KCC2 function counteracts morphine-induced hyperalgesia. Scientific reports 7:3870. <https://doi.org/10.1038/s41598-017-04209-3>

Ferrini F.R.D., Marba S.T.M., Gaviao M.B.D. (2008) Oral conditions in very low and extremely low birth weight children. Journal of Dentistry for Children 75:235–242

Fete T.J., Fete M. (2016) International research symposium on Goltz syndrome. American Journal of Medical Genetics, Part C: Seminars in Medical Genetics 172:3–6. <https://doi.org/10.1002/ajmg.c.31475>

Fickl S, Thalmair T, Kebschull M, et al (2009) Microsurgical access flap in conjunction with enamel matrix derivative for the treatment of intra-bony defects: a controlled clinical trial. Journal of clinical periodontology 36:784‐790. <https://doi.org/10.1111/j.1600-051X.2009.01451.x>

Field R (2019) Aesthetic management of white lesions in the permanent dentition. Dental Update 46:313–322

Filipova D, Dostalova T, Filipi V, Kaminek M (2019) Proclination-induced changes in the labial cortical bone thickness of lower incisors. Bratislavske lekarske listy 120:155–160. <https://doi.org/10.4149/BLL_2019_025>

Filitis D.C., Graber E.M. (2013) Minocycline-induced hyperpigmentation involving the oral mucosa after short-term minocycline use. Cutis 92:46–48

Fine J.-D., Mellerio J.E. (2009) Extracutaneous manifestations and complications of inherited epidermolysis bullosa. Part II. Other organs. Journal of the American Academy of Dermatology 61:387–402. <https://doi.org/10.1016/j.jaad.2009.03.053>

Finkelstein T, Shapira Y, Shpack N (2012) Nonsurgical treatment of severe open bite associated with amelogenesis imperfecta. Journal of clinical orthodontics : JCO 46:427

Firmino RT, Ferreira FM, Paiva SM, et al (2017) Oral health literacy and associated oral conditions: A systematic review. Journal of the American Dental Association (1939) 148:604–613. <https://doi.org/10.1016/j.adaj.2017.04.012>

Firooz F, Heidari B, Vafaei F, et al (2017) Association between Enamel Abrasion and Surface Roughness of Dental Ceramics. Journal of Mashhad Dental School 41:51–60

Firth FA (2017) A Mechanical Strain Model for the Assessment of Periodontal Ligament Cell Endoplasmic Reticulum Stress in Three-dimensional Culture: A Thesis Submitted in Partial Fulfilment of the Requirements for the Degree of Doctor of Clinical Dentistry (Orthodontics), Department of Oral Sciences, University of Otago,[Dunedin,] New Zealand

Fischer P (2016a) Molar Incisor Hypomineralisation-Overview, Clinical Aspects, Treatment Recommendations. INFORMATIONEN AUS ORTHODONTIE UND KIEFERORTHOPAEDIE 48:195–199

Fischer P (2016b) Molaren-Inzisiven-Hypomineralisation–Überblick, klinische Aspekte, Behandlungsempfehlungen. Informationen aus Orthodontie & Kieferorthopädie 48:195–199

Fish L.M., Foster J.S., Kestler D.P., et al (2012) Odontogenic ameloblast-associated protein (ODAM) alters the growth and migration of human melanoma cells. Cancer Research 72:. <https://doi.org/10.1158/1538-7445.AM2012-4302>

Fishbein M.C., Levy R.J., Ferrans V.J. (1982) Calcification of cardiac valve bioprostheses. Biochemical, histologic, and ultrastructural observations in a subcutaneous implantation model system. Journal of Thoracic and Cardiovascular Surgery 83:602–609

FitzGerald C.M., Saunders S.R. (2005) Test of histological methods of determining chronology of accentuated striae in deciduous teeth. American Journal of Physical Anthropology 127:277–290. <https://doi.org/10.1002/ajpa.10442>

Fitzgerald J, Verveniotis SJ (1998) Morquio’s syndrome. A case report and review of clinical findings. The New York state dental journal 64:48–50

FitzGerald K, Fleming P, Franklin O (2010) Dental health and management for children with congenital heart disease. Primary Dental Care 17:21–25

Fitzpatrick L, O’Connell A (2007) First permanent molars with molar incisor hypomineralisation. Journal of the Irish Dental Association 53:32–37

Fırat MG, Bulut D Continuation of Apexogenesis around Fracture Endodontic File

Flaherty KR, Toews GB, Lynch JP, et al (2001) Steroids in idiopathic pulmonary fibrosis: a prospective assessment of adverse reactions, response to therapy, and survival. The American journal of medicine 110:278–282

Fleischer-Peters A (1987) [Pediatric aspects of orthodontics]. Fortschritte der Kieferorthopadie 48:475–485

Fleischer-Peters A. (1987) Padiatrische Aspekte in der KieferorthopadiePediatric aspects of orthodontics. Fortschritte der Kieferorthopadie 48:475–485

Flett AM, Sandler J (2016) The role of the GDP in assessment and management of the early orthodontic referral. Dental Update 43:706–720

Flexeder C, Hassan LK, Standl M, et al (2020) Is There an Association between Asthma and Dental Caries and Molar Incisor Hypomineralisation? Caries Research 54:86–94

Flores M.T., Onetto J.E. (2019) How does orofacial trauma in children affect the developing dentition? Long-term treatment and associated complications. Dental traumatology : official publication of International Association for Dental Traumatology 35:312–323. <https://doi.org/10.1111/edt.12496>

Florès-de-Jacoby L, Zimmermann A, Tsalikis L (1994) Experiences with guided tissue regeneration in the treatment of advanced periodontal disease. A clinical re-entry study. Part I. Vertical, horizontal and horizontal periodontal defects. Journal of clinical periodontology 21:113–117

Flores-Mir C, McGrath L, Heo G, Major P (2013) Efficiency of molar distalization with the XBow appliance related to second molar eruption stage. European journal of orthodontics 35:745–751

Floyd B (2007) Focused life history data and linear enamel hypoplasia to help explain intergenerational variation in relative leg length within Taiwanese families. American journal of human biology : the official journal of the Human Biology Council 19:358–375

Fnaish MM, Alawneh AM, Da’ameh M, Al-Share AA (2011) Dental anomalies in children in North Jordan. Pakistan Oral and Dental Journal 31:

Folayan MO (2019) A Global Compendium of Oral Health: Tooth Eruption and Hard Dental Tissue Anomalies. Cambridge Scholars Publishing

Folayan MO, Alade M, Adeniyi A, et al (2019) Association between developmental dental anomalies, early childhood caries and oral hygiene status of 3-5-year-old children in Ile-Ife, Nigeria. BMC oral health 20:1. <https://doi.org/10.1186/s12903-019-0991-2>

Folayan MO, Chukwumah NM, Popoola BO, et al (2018a) Developmental defects of the enamel and its impact on the oral health quality of life of children resident in Southwest Nigeria. BMC oral health 18:160

Folayan MO, Oyedele TA, Oziegbe E (2018b) Time expended on managing molar incisor hypomineralization in a pediatric dental clinic in Nigeria. Brazilian oral research 32:e79. <https://doi.org/10.1590/1807-3107bor-2018.vol32.0079>

Folwaczny M, Loher C, Mehl A, et al (2001) Class V lesions restored with four different tooth-colored materials--3-year results. Clinical oral investigations 5:31‐39. <https://doi.org/10.1007/s007840000098>

FONSECA BB, CAVALCANTI LG, FURQUIM CP, et al (2015) Herpes Zoster of the Trigeminal Nerve: A Case Report. Oral Surgery, Oral Medicine, Oral Pathology and Oral Radiology 120:e80

Font A., Casas J.I., Koller T., et al (2013) Anaesthetic management of a child affected by Lenz-Majewski syndrome: A case report. European Journal of Anaesthesiology 30:159–160

Foo S.H., Moss C., Goodwin R., et al (2016) Terminal osseous dysplasia with pigmentary defects. British Journal of Dermatology 175:84–85. <https://doi.org/10.1111/bjd.14578>

Foo S.H., Moss C., Goodwin R., et al (2018) Blaschkoid pigmentation, keloids and disabling digital fibromas in Borjeson-Forssman-Lehmann syndrome. British Journal of Dermatology 179:35

Ford D, Seow WK, Kazoullis S, et al (2009) A controlled study of risk factors for enamel hypoplasia in the permanent dentition. Pediatric dentistry 31:382–388

Fornaini C., Rocca J.-P., Merigo E., Xu N. (2019) Er:YAG laser for the aesthetic treatment of developmental enamel defects in frontal teeth: A case report. Laser Therapy 28:199–202. <https://doi.org/10.5978/islsm.28_19-CR-02>

Foroughi R (1978) [Acid etch and enamel bond composite. Restoration of permanent anterior teeth affected by enamel hypoplasia]. Journal of the Dental School, National University of Iran 9:37–45

Forrest JR, James PM (1965) A blind study of enamel opacities and dental caries prevalence after eight years of fluoridation of water. British dental journal 119:319–322

Foster J, Pan T, Rokadia H (2019) AN UNUSUAL CAUSE OF ACUTE EOSINOPHILIC PNEUMONIA. Chest 156:A1544‐. <https://doi.org/10.1016/j.chest.2019.08.1362>

Foteva K, Zarkova J, Zlatanovska K (2014) Treatment modalities of fractured maxillary incisors with pulp exposure (Case report)

Foulds H (2017) Developmental defects of enamel and caries in primary teeth. Evidence-based dentistry 18:72–73. <https://doi.org/10.1038/sj.ebd.6401252>

Fowler EB, Breault LG (2001) Ridge augmentation with a folded acellular dermal matrix allograft: a case report. The journal of contemporary dental practice 2:31–40

Fox DJ, Pappas P (1975) Use of an acid etch resin in hereditary enamel hypoplasia: report of case. ASDC journal of dentistry for children 42:137–139

Fragelli CMB (2012) Hipomineralização molar-inciso: avaliação longitudinal em crianças de 6 a 9 anos

Fragelli CMB (2016) Hipomineralização molar incisivo: qualidade de vida relacionada à saúde bucal e percepção estética em escolares de 8 a 12 anos

Fragelli CMB, Jeremias F, Santos-Pinto LAM dos (2013) Manifestation of molar-incisor hypomineralisation in twins: clinical case reports. Brazilian Dental Science 90–96

Franca R., Goodman A. (2012) A regression method for the timing of micro enamel defects. American Journal of Physical Anthropology 147:146. <https://doi.org/10.1002/ajpa.22033>

Franca-Grohmann I.L., Sangiorgio J.P.M., Bueno M.R., et al (2020) Treatment of dehiscence-type defects with collagen matrix and/or enamel matrix derivative: Histomorphometric study in minipigs. Journal of Periodontology 91:967–974. <https://doi.org/10.1002/JPER.19-0107>

Francetti L., Del Fabbro M., Basso M., et al (2004) Enamel matrix proteins in the treatment of intra-bony defects. A prospective 24-month clinical trial. Journal of clinical periodontology 31:52–59

Francetti L., Del Fabbro M., Trombelli L., et al (2005) Evaluation of efficacy of enamel matrix derivative in the treatment of intrabony defects: A 24-month multicenter study. International Journal of Periodontics and Restorative Dentistry 25:461–473

Franek E, Blaschyk R, Kolonko A, et al (2006) [Oral hygiene in haemodialyzed patients with chronic renal failure]. Wiadomosci lekarskie (Warsaw, Poland : 1960) 59:184–188

Franklin D., Senior N., James I., Roberts G. (2000) Oral health status of children in a paediatric intensive care unit. Intensive Care Medicine 26:319–324. <https://doi.org/10.1007/s001340051156>

Franz-Odendaal TA, Lee-Thorp JA, Chinsamy A (2003) Insights from stable light isotopes on enamel defects and weaning in Pliocene herbivores. Journal of biosciences 28:765–773

Fraser SJ, Natarajan AK, Clark AS, et al (2015) A Raman spectroscopic study of teeth affected with molar–incisor hypomineralisation. Journal of Raman Spectroscopy 46:202–210

Fratto G, Lucchetti MC, Ruspoli CG, Di Giorgio R (1984) [Composites: clinical use of a new material in the esthetic reconstruction of anterior teeth]. Dental Cadmos 52:85–94

Frazao P., Peverari A.C., Forni T.I., et al (2004) Fluorose dentaria: comparacao de dois estudos de prevalenciaDental fluorosis: comparison of two prevalence studies. Cadernos de saude publica / Ministerio da Saude, Fundacao Oswaldo Cruz, Escola Nacional de Saude Publica 20:1050–1058

Frazão P, Peverari AC, Forni TIB, et al (2004) [Dental fluorosis: comparison of two prevalence studies]. Cadernos de saude publica 20:1050–1058

Frazier AA, Galvin JR, Franks TJ, Rosado-De-Christenson ML (2000) From the archives of the AFIP: pulmonary vasculature: hypertension and infarction. Radiographics : a review publication of the Radiological Society of North America, Inc 20:491

Frenkel B, Blinder D, Penn M (2010) [Isolated oligodontia: a case presentation and review of the literature]. Refu’at ha-peh veha-shinayim (1993) 27:6–10

Friedman I.S., Shelton R.M., Phelps R.G. (2002) Minocycline-induced hyperpigmentation of the tongue: Successful treatment with the Q-switched ruby laser. Dermatologic Surgery 28:205–209. <https://doi.org/10.1046/j.1524-4725.2002.01083.x>

Frisk F, Kvist T, Axelsson S, et al (2013) Pulp exposures in adults–choice of treatment among Swedish dentists. Swed Dent J 37:153–60

Fron H, Vergnes J, Moussally C, et al (2011) Effectiveness of a new one-step self-etch adhesive in the restoration of non-carious cervical lesions: 2-year results of a randomized controlled practice-based study. Dental materials 27:304‐312. <https://doi.org/10.1016/j.dental.2010.11.006>

Froum S., Weinberg M., Novak J., et al (2004) A multicenter study evaluating the sensitization potential of enamel matrix derivative after treatment of two infrabony defects. Journal of Periodontology 75:1001–1008. <https://doi.org/10.1902/jop.2004.75.7.1001>

Froum S, Weinberg M, Rosenberg E, Tarnow D (2001) A comparative study utilizing open flap debridement with and without enamel matrix derivative in the treatment of periodontal intrabony defects: a 12-month re-entry study. Journal of periodontology 72:25‐34. <https://doi.org/10.1902/jop.2001.72.1.25>

Froum SJ, Froum SH, Rosen PS (2012) Successful management of peri-implantitis with a regenerative approach: a consecutive series of 51 treated implants with 3- to 7.5-year follow-up. The International journal of periodontics & restorative dentistry 32:11–20

Fryer CE, Brown RS, Osborne SD (2015) Uncontrolled Dental Caries in a Young Adult: A Therapeutic Perspective and Case Report. Dentistry today 34:46–47

Fteita D., Ali A., Alaluusua S. (2006) Molar-incisor hypomineralization (MIH) in a group of school-aged children in Benghazi, Libya. European archives of paediatric dentistry : official journal of the European Academy of Paediatric Dentistry 1:92–95

Fu Q., Wang Y., Li X., Lei X. (2006) Comparison of the enamel structure of human severely fluorotic teeth between water-drinking endemic fluorosis area and coal-burning endemic fluorosis area. Medical Journal of Wuhan University 27:326–329

Fuchs C, Buske G, Krämer N (2009) Schmelzbildungsstörungen–Fallbericht einer generalisierten Schmelzbildungsstörung in der 1. Dentition. Oralprophylaxe Kinderzahnheilkd 31:178–186

Fujinami K., Hayakawa H., Ota K., et al (2011) Two-year follow-up of treatment of intrabony periodontal defect with enamel matrix derivative. The Bulletin of Tokyo Dental College 52:215–221. <https://doi.org/10.2209/tdcpublication.52.215>

Fukui N, Amano A, Akiyama S, et al (2000) Oral findings in DiGeorge syndrome: clinical features and histologic study of primary teeth. Oral surgery, oral medicine, oral pathology, oral radiology, and endodontics 89:208–215

Fukui N., Amano A., Akiyama S., et al (2000) Oral findings in DiGeorge syndrome: clinical features and histologic study of primary teeth. Oral surgery, oral medicine, oral pathology, oral radiology, and endodontics 89:208–215

Fukuta Y., Totsuka M., Takeda Y., et al (2001) Histological and analytical studies of a tooth in a patient with cleidocranial dysostosis. Journal of oral science 43:85–89. <https://doi.org/10.2334/josnusd.43.85>

Fulton A, Amlani M, Parekh S (2020) Oral manifestations of vitamin D deficiency in children. British dental journal 228:515–518. <https://doi.org/10.1038/s41415-020-1424-y>

Fulton A.J., Fiani N., Verstraete F.J.M. (2014) Canine pediatric dentistry. Veterinary Clinics of North America - Small Animal Practice 44:303–324. <https://doi.org/10.1016/j.cvsm.2013.11.004>

Fumagalli Romario U, Puccetti F, Elmore U, et al (2013) Self-gripping mesh versus staple fixation in laparoscopic inguinal hernia repair: a prospective comparison. Surgical endoscopy 27:1798‐1802. <https://doi.org/10.1007/s00464-012-2683-8>

Gabardo G, Maciel JVB, Franco A, et al (2020) Radiographic analysis of dental maturation in children with amelogenesis imperfecta: A case-control study. Special care in dentistry : official publication of the American Association of Hospital Dentists, the Academy of Dentistry for the Handicapped, and the American Society for Geriatric Dentistry 40:267–272. <https://doi.org/10.1111/scd.12456>

GABRIEL D (2011) Molar-incisor hypomineralisation (MIH) (current data and epidemiological study in two schools of Montpellier)

Gabriel LAR, Sachdeva R, Marcotty A, et al (2011) Oculodentodigital dysplasia: new ocular findings and a novel connexin 43 mutation. Archives of ophthalmology (Chicago, Ill : 1960) 129:781–784. <https://doi.org/10.1001/archophthalmol.2011.113>

Gadhia K, McDonald S, Arkutu N, Malik K (2012) Amelogenesis imperfecta: an introduction. British dental journal 212:377–379. <https://doi.org/10.1038/sj.bdj.2012.314>

Gaffaney TE (2004) Guided tissue regeneration using a bioabsorbable membrane: a 21-case series. Journal of periodontology 75:1728–1733

Gaillard T, Briolant S, Madamet M, Pradines B (2017) The end of a dogma: the safety of doxycycline use in young children for malaria treatment. Malaria journal 16:148. <https://doi.org/10.1186/s12936-017-1797-9>

Gainey CE, Brown HA, Gerard WC (2018) Utilization of Mobile Integrated Health Providers During a Flood Disaster in South Carolina (USA). Prehospital and disaster medicine 33:432–435. <https://doi.org/10.1017/S1049023X18000572>

Gait TC (2017) LIFE IN ACADEMIA

Galgut P, Verrier J, Waite I, et al (1991) Computerized densitometric analysis of interproximal bone levels in a controlled clinical study into the treatment of periodontal bone defects with ceramic hydroxyapatite implant material. Journal of periodontology 62:44‐50. <https://doi.org/10.1902/jop.1991.62.1.44>

Galhotra V, Ahluwalia P, Jodhka S, et al (2015) Effect of nutritional rickets on dental development in North Indian children: A prospective study. Journal of Pediatric Dentistry 3:88

Gallacher AA, Pemberton MN, Waring DT (2018) The dental manifestations and orthodontic implications of hypoparathyroidism in childhood. Journal of orthodontics 45:46–50. <https://doi.org/10.1080/14653125.2017.1406685>

Gallusi G, Libonati A, Campanella V (2006) SEM-morphology in dentinogenesis imperfecta type II: microscopic anatomy and efficacy of a dentine bonding system. European journal of paediatric dentistry 7:9–17

Gambetta-Tessini K (2017) Comparative studies of demarcated hypomineralised lesions of dental enamel and their fluorescence properties

Gambetta-Tessini K, Mariño R, Ghanim A, et al (2016) Knowledge, experience and perceptions regarding Molar-Incisor Hypomineralisation (MIH) amongst Australian and Chilean public oral health care practitioners. BMC oral health 16:75. <https://doi.org/10.1186/s12903-016-0279-8>

Gambetta-Tessini K, Mariño R, Ghanim A, et al (2019) The impact of MIH/HSPM on the carious lesion severity of schoolchildren from Talca, Chile. European Archives of Paediatric Dentistry 20:417–423

Gambetta‐Tessini K, Marino R, Ghanim A, et al (2018) Carious lesion severity and demarcated hypomineralized lesions of tooth enamel in schoolchildren from Melbourne, Australia. Australian dental journal 63:365–373

Gamble J.A. (2016) Exploring stress thresholds through dental enamel defects and skeletal evidence for life history trade-offs in adults. American Journal of Physical Anthropology 159:150–151. <https://doi.org/10.1002/ajpa.22955>

Gamble J.A. (2019) The bioarchaeology of urbanisation in Denmark. American Journal of Physical Anthropology 168:81. <https://doi.org/10.1002/ajpa.23802>

Gamboa G, Lee G, Ekambaram M, Yiu C (2018) Knowledge, perceptions, and clinical experiences on molar incisor hypomineralization among dental care providers in Hong Kong. BMC oral health 18:217‐. <https://doi.org/10.1186/s12903-018-0678-0>

Gandhi S, Crawford P, Shellis P (2012) The use of a “bleach-etch-seal” deproteinization technique on MIH affected enamel. International journal of paediatric dentistry 22:427–434. <https://doi.org/10.1111/j.1365-263X.2011.01212.x>

Gandolfi B, Liu H, Griffioen L, Pedersen NC (2013) Simple recessive mutation in ENAM is associated with amelogenesis imperfecta in Italian Greyhounds. Animal genetics 44:569–578. <https://doi.org/10.1111/age.12043>

Gangwar A, Singal D, Giri KY, et al (2014) An Immature Type II Dens Invaginatus in a Mandibular Lateral Incisor with Talon’s Cusp: A Clinical Dilemma to Confront. Case reports in dentistry 2014:826294. <https://doi.org/10.1155/2014/826294>

Gao J, Li X, Gao L, et al (2020) Effects of applying amoxicillin in juvenile mice on enamel mineralization and the expression of kallikrein‑related peptidase 4 and tight junction proteins in ameloblasts. International Journal of Molecular Medicine 46:179–190

Gao J., Ruan J., Gao L. (2014) Excessive fluoride reduces Foxo1 expression in dental epithelial cells of the rat incisor. European journal of oral sciences 122:317–323. <https://doi.org/10.1111/eos.12148>

Gao J., Ruan J., Li X., et al (2020) Effects of applying amoxicillin in juvenile mice on enamel mineralization and the expression of kallikrein-related peptidase 4 and tight junction proteins in ameloblasts. International Journal of Molecular Medicine 46:179–190. <https://doi.org/10.3892/ijmm.2020.4598>

Gao X-J, Lü P, Zhuang H (2009) [Problem solving in endodontic diseases: I. Enamel developmental defects: clinical classification and molecular biologic mechanism]. Zhonghua kou qiang yi xue za zhi = Zhonghua kouqiang yixue zazhi = Chinese journal of stomatology 44:314–317

Garcés EAD, Pérez DCG, Pensabene G, et al Dental Fase

Garcia Ballesta C, Perez Lajarin L (1985) [Use of dentin adhesives in the conservative treatment of amelogenesis imperfecta]. Revista espanola de estomatologia 33:473–480

Garcia M., Trindade-Suedam I. (2018) Ideal vs late secondary alveolar bone graft surgery: Long-term clinical follow-up and tomographic assessment. Cleft Palate-Craniofacial Journal 55:21. <https://doi.org/10.1177/1055665618761376>

Garcia M.F., Racciatti G. (2016) Comparative study of pulp calcifications incidence in rats molars: Diabetics vs controls. Biocell 40:

García-Briseño M, Martens L, Cauwels R No. 11-June 2015

Garcia-Margarit M., Catala-Pizarro M., Montiel-Company J.M., Almerich-Silla J.M. (2014) Epidemiologic study of molar-incisor hypomineralization in 8-year-old Spanish children. International journal of paediatric dentistry / the British Paedodontic Society [and] the International Association of Dentistry for Children 24:14–22. <https://doi.org/10.1111/ipd.12020>

Garg C, Shrimanker I, Goel S, et al (2020) Extraintestinal Manifestations of Crohn’s Disease in the Form of Pulmonary Nodules: A Case Report. Cureus 12:e7161. <https://doi.org/10.7759/cureus.7161>

Garg D, Mahabala K, Lewis A, et al (2019) Comparative evaluation of sealing ability, penetration and adaptation of a self etching pit and fissure sealant-stereomicroscopic and scanning electron microscopic analyses. Journal of Clinical and Experimental Dentistry 11:e547

Garg N, Jain AK, Saha S, Singh J (2012) Essentiality of early diagnosis of molar incisor hypomineralization in children and review of its clinical presentation, etiology and management. International journal of clinical pediatric dentistry 5:190–196. <https://doi.org/10.5005/jp-journals-10005-1164>

GAROT E (2012) Study of molars incisors hypomineralisation etiological hypotheses on 130 patients

Garot E, Couture-Veschambre C, Manton D, et al (2017a) Analytical evidence of enamel hypomineralisation on permanent and primary molars amongst past populations. Scientific reports 7:1–10

Garot E, Couture-Veschambre C, Manton D, et al (2017b) Diagnostic guide enabling distinction between taphonomic stains and enamel hypomineralisation in an archaeological context. Archives of Oral Biology 74:28–36

Garot E, Couture‐Veschambre C, Manton DJ, et al (2019) Differential diagnoses of enamel hypomineralisation in an archaeological context: A postmedieval skeletal collection reassessment. International Journal of Osteoarchaeology 29:747–759

Garot E, Denis A, Delbos Y, et al (2018) Are hypomineralised lesions on second primary molars (HSPM) a predictive sign of molar incisor hypomineralisation (MIH)? A systematic review and a meta-analysis. Journal of dentistry 72:8–13. <https://doi.org/10.1016/j.jdent.2018.03.005>

Garot E, Manton D, Rouas P (2016a) Peripartum events and molar-incisor hypomineralisation (MIH) amongst young patients in southwest France. European Archives of Paediatric Dentistry 17:245–250

Garot E, Rouas P, D’Incau E, et al (2016b) Mineral density of hypomineralised and sound enamel. Bulletin du Groupement international pour la recherche scientifique en stomatologie & odontologie 53:e33

Gasque KCS, Foster BL, Kuss P, et al (2015) Improvement of the skeletal and dental hypophosphatasia phenotype in Alpl-/- mice by administration of soluble (non-targeted) chimeric alkaline phosphatase. Bone 72:137–147. <https://doi.org/10.1016/j.bone.2014.11.017>

Gautam B, Sreedharan S, Seetha SM (2019) Photographic Profile Analysis in Preschool Children of Thiruvananthapuram, Kerala. International journal of clinical pediatric dentistry 12:111–115. <https://doi.org/10.5005/jp-journals-10005-1605>

Gawade P.L., Hudson M.M., Robison L.L., et al (2014) A systematic review of dental late effects in survivors of childhood cancer. Pediatric Blood and Cancer 61:407–416. <https://doi.org/10.1002/pbc.24842>

Gay SE, Kazerooni EA, Toews GB, et al (1998) Idiopathic pulmonary fibrosis: predicting response to therapy and survival. American journal of respiratory and critical care medicine 157:1063–1072

Ge Z, Ma R, Li G, et al (2015) Age estimation based on pulp chamber volume of first molars from cone-beam computed tomography images. Forensic science international 253:133. <https://doi.org/10.1016/j.forsciint.2015.05.004>

Gedalia I, Braustein E, Lewinstein I, et al (1996) Fluoride and hard cheese exposure on etched enamel in neck-irradiated patients in situ. Journal of dentistry 24:365–368

Gedalia I, Shapira L (1989) Effect of prenatal and postnatal fluoride on the human deciduous dentition. A literature review. Advances in dental research 3:168–176

Geerinckx T., de Kegel B., Adriaens D., et al (2012) Soft dentin results in unique flexible teeth in scraping catfishes. Physiological and Biochemical Zoology 85:481–490. <https://doi.org/10.1086/667532>

Geetha Priya PR, John JB, Elango I (2010) Turner’s hypoplasia and non-vitality: a case report of sequelae in permanent tooth. Contemporary clinical dentistry 1:251–254. <https://doi.org/10.4103/0976-237X.76395>

Gemalmaz D, Isik F, Keles A, Küker D (2003) Use of adhesively inserted full-ceramic restorations in the conservative treatment of amelogenesis imperfecta: a case report. The journal of adhesive dentistry 5:235–242

Gençer MDG, KIRZIOĞLU Z (2019) A comparison of the effectiveness of resin infiltration and microabrasion treatments applied to developmental enamel defects in color masking. Dental materials journal 2018–074

GENÇER MDG, KIRZIOĞLU Z Evaluation of Color Masking Effectiveness by Visual and Instrumental Methods of Various Materials Used In the Treatment of Developmental Enamel Defects. Black Sea Journal of Health Science 1:46–53

GENÇER MDG, KIRZIOĞLU Z Resin Infiltration Systems’ Color Masking Effect on Developmental Enamel Defects. Black Sea Journal of Health Science 1:70–76

Gerdolle D, Mortier E, Richard A, Vailati F (2015) Full-mouth adhesive rehabilitation in a case of amelogenesis imperfecta: a 5-year follow-up case report. The international journal of esthetic dentistry 10:12–31

Gerlach RF, de Souza AP, Cury JA, Line SR (2000) Fluoride effect on the activity of enamel matrix proteinases in vitro. European journal of oral sciences 108:48–53

Gerlach R.F., Jorge Jr. J., de Almeida O.P., et al (1998) Regional odontodysplasia. Report of two cases. Oral surgery, oral medicine, oral pathology, oral radiology, and endodontics 85:308–313

Gerreth K, Zaorska K, Zabel M, et al (2018) Significance of genetic variations in developmental enamel defects of primary dentition in Polish children. Clinical oral investigations 22:321–329

Geyer M., Fairchild J., Tham E., et al (2014) Recalcitrant hypocalcaemia in autoimmune enteropathy. Pediatrics 134:. <https://doi.org/10.1542/peds.2013-3308>

Ghanim A, Elfrink M, Weerheijm K, et al (2015) A practical method for use in epidemiological studies on enamel hypomineralisation. European archives of paediatric dentistry 16:235–246

Ghanim A, Manton D, Bailey D, et al (2013a) Risk factors in the occurrence of molar–incisor hypomineralization amongst a group of Iraqi children. International Journal of Paediatric Dentistry 23:197–206

Ghanim A, Manton D, Marino R, et al (2013b) Prevalence of demarcated hypomineralisation defects in second primary molars in Iraqi children. International journal of paediatric dentistry 23:48–55

Ghanim A., Marino R., Manton D.J. (2019) Validity and reproducibility testing of the Molar Incisor Hypomineralisation (MIH) Index. International journal of paediatric dentistry 29:6–13. <https://doi.org/10.1111/ipd.12433>

Ghanim A, Marino R, Morgan M, et al (2013c) An in vivo investigation of salivary properties, enamel hypomineralisation, and carious lesion severity in a group of Iraqi schoolchildren. International journal of paediatric dentistry 23:2–12

Ghanim A, Morgan M, Mariño R, et al (2011a) Perception of molar-incisor hypomineralisation (MIH) by Iraqi dental academics. International journal of paediatric dentistry 21:261–270. <https://doi.org/10.1111/j.1365-263X.2011.01118.x>

Ghanim A, Morgan M, Mariño R, et al (2011b) Molar-incisor hypomineralisation: prevalence and defect characteristics in Iraqi children. International journal of paediatric dentistry 21:413–421. <https://doi.org/10.1111/j.1365-263X.2011.01143.x>

Ghanim A, Morgan M, Marino R, et al (2012) Risk factors of hypomineralised second primary molars in a group of Iraqi schoolchildren. European Archives of Paediatric Dentistry 13:111–118

Ghanim A, Silva MJ, Elfrink MEC, et al (2017) Molar incisor hypomineralisation (MIH) training manual for clinical field surveys and practice. European archives of paediatric dentistry : official journal of the European Academy of Paediatric Dentistry 18:225–242. <https://doi.org/10.1007/s40368-017-0293-9>

Ghanim A.M., Manton D.J., Morgan M.V., et al (2012) Trends of oral health care and dental treatment needs in relation to molar incisor hypomineralisation defects: A study amongst a group of Iraqi schoolchildren. European Archives of Paediatric Dentistry 13:171–178. <https://doi.org/10.1007/BF03262866>

Ghasemlou M, Khodaiyan F, Jahanbin K, et al (2012) Structural investigation and response surface optimisation for improvement of kefiran production yield from a low-cost culture medium. Food chemistry 133:383–389. <https://doi.org/10.1016/j.foodchem.2012.01.046>

Ghezzi C, Ferrantino L, Bernardini L, et al (2016) Minimally Invasive Surgical Technique in Periodontal Regeneration: a Randomized Controlled Clinical Trial Pilot Study. International journal of periodontics & restorative dentistry 36:475‐482. <https://doi.org/10.11607/prd.2550>

Ghodsi S, Rasaeipour S, Vojdani M (2012) Oral rehabilitation of a patient with amelogenesis imperfecta using removable overlay denture: a clinical report. The journal of contemporary dental practice 13:227–231

Ghoneim MM, Aboushelib MN, Dawod H, et al (2020) Effect of preheating and ultrasonic energy on penetration of low viscosity enamel resin infiltrant. Egyptian Dental Journal 66:423–427

Ghosh J., Baguneid M., Khwaja N., et al (2006) Reduction of myointimal hyperplasia after arterial anastomosis by local injection of transforming growth factor beta3. Journal of Vascular Surgery 43:142–149. <https://doi.org/10.1016/j.jvs.2005.08.041>

Ghougassian SS, Ghafari JG (2014) Association between mandibular third molar formation and retromolar space. The Angle orthodontist 84:946–950. <https://doi.org/10.2319/120113-883.1>

Giannetti L, Apponi R (2020) Combined Orthodontic and Restorative Minimally Invasive Approach to Diastema and Morphology Management in the Esthetic Area. Clinical Multidisciplinary Case Report with 3-Year Follow-Up. Case Reports in Dentistry 2020:

Giannetti L, Murri Dello Diago A, Corciolani E, Spinas E (2018a) Deep infiltration for the treatment of hypomineralized enamel lesions in a patient with molar incisor hypomineralization: a clinical case. Journal of biological regulators and homeostatic agents 32:751–754

Giannetti L, Murri Dello Diago A, Silingardi G, Spinas E (2018c) " Superficial infiltration to treat white hypomineralized defects of enamel: clinical trial with 12-month follow-up. Journal of biological regulators and homeostatic agents 32:1335–1338

Gibb H, Grossman BF, Dickman CR, et al (2019) Long-term responses of desert ant assemblages to climate. The Journal of animal ecology 88:1549–1563. <https://doi.org/10.1111/1365-2656.13052>

Gibbard PD (1974) The management of children and adolescents suffering from amelogenesis imperfecta and dentinogenesis imperfecta. International journal of orthodontics 12:15–25

Gibson C.W., Yuan Z.-A., Chen E., et al (2001) Amelogenin-deficient Mice Display an Amelogenesis Imperfecta Phenotype. Journal of Biological Chemistry 276:31871–31875. <https://doi.org/10.1074/jbc.M104624200>

Gigli S., Ko J., Hoffman W., Oberoi S. (2019) Three-dimensional analysis of buccal cortical bone thickness in individuals with nonsyndromic unilateral cleft lip and palate. Cleft Palate-Craniofacial Journal 56:85. <https://doi.org/10.1177/1055665619829661>

Gioacchini G., Carnevali O., Bizzaro D., et al (2010) Oocytes maturation induction by lactobacillus rhamnosus in Danio rerio: In vivo and in vitro studies. Human Reproduction 25:. <https://doi.org/10.1093/humrep/de.25.s1.140>

Giri J, Shrestha BK, Yadav R, Ghimire TR (2016) Assessment of skeletal maturation with permanent mandibular second molar calcification stages among a group of Nepalese orthodontic patients. Clinical, cosmetic and investigational dentistry 8:57–62. <https://doi.org/10.2147/CCIDE.S93561>

Gisler V, Enkling N, Zix J, et al (2010) A multidisciplinary approach to the functional and esthetic rehabilitation of amelogenesis imperfecta and open bite deformity: a case report. Journal of esthetic and restorative dentistry : official publication of the American Academy of Esthetic Dentistry . [et al] 22:282–293. <https://doi.org/10.1111/j.1708-8240.2010.00354.x>

Giuca M.R., Cei G., Gigli F., Gandini P. (2010) Oral signs in the diagnosis of celiac disease: review of the literature. Minerva stomatologica 59:33–43

Giuca MR, Lardani L, Pasini M, et al (2020) State-of-the-art on MIH. Part. 1 Definition and aepidemiology. European journal of paediatric dentistry 21:80–82. <https://doi.org/10.23804/ejpd.2020.21.01.16>

Giuffra V, Panetta D, Salvadori PA, Fornaciari G (2014) A historical case of amelogenesis imperfecta: Giovanna of Austria, Grand Duchess of Tuscany (1547-1578). European journal of oral sciences 122:1–6. <https://doi.org/10.1111/eos.12097>

Gjørup H, Haubek D, Hintze H, et al (2009) Hypocalcified type of amelogenesis imperfecta in a large family: clinical, radiographic, and histological findings, associated dento-facial anomalies, and resulting treatment load. Acta odontologica Scandinavica 67:240–247. <https://doi.org/10.1080/00016350902973685>

Gkranias ND, Graziani F, Sculean A, Donos N (2012) Wound healing following regenerative procedures in furcation degree III defects: histomorphometric outcomes. Clinical oral investigations 16:239–249. <https://doi.org/10.1007/s00784-010-0478-7>

Gleicher H, Fuks A, Sela J (1998) Adaptation of Class II Vitremer restorations with and without primer: a morphometric study. Pediatric dentistry 20:263‐266

Glodkowska N., Emerich K. (2016) Postrzeganie i swiadomosc hipomineralizacji trzonowcowo-siekaczowej (MIH) wsrod polskich lekarzy dentystowPerception and awareness of molar incisor hypomineralisation among polish dentists. Dental and Medical Problems 53:382–393. <https://doi.org/10.17219/dmp/62523>

Goda Y, Shoji T, Katakura H (2020) Unilateral antibiotic-induced acute eosinophilic pneumonia on the operative side after surgery for primary lung cancer: a case report. Surgical case reports 6:40. <https://doi.org/10.1186/s40792-020-00803-2>

Goel M, Sachdeva GS, Bala S, et al (2013) Letter to the editor. Operative dentistry 38:676. <https://doi.org/10.2341/1559-2863-38.6.676>

Goel S., Minz R., Sehgal S., et al (2014) PCR array based approach to find IL-6/STAT3 mediated downstream target genes involved in the skeletal pathogenesis of hyper-IGE syndrome. Journal of Clinical Immunology 34:. <https://doi.org/10.1007/s10875-014-0101-9>

Goh N, Raghu G, Wells A, et al (2016) Consistent effect of nintedanib on decline in FVC in patients across subgroups based on highresolution computed tomography diagnostic criteria: results from the INPULSIS trials in idiopathic pulmonary fibrosis. Respirology 21:148. <https://doi.org/10.1111/resp.12755>

Goho C. (1993) Chemoradiation therapy: effect on dental development. Pediatric dentistry 15:6–12

Gokce K, Canpolat C, Ozel E (2007) Restoring function and esthetics in a patient with amelogenesis imperfecta: a case report. The journal of contemporary dental practice 8:95–101

Göland U, Koch G, Paulander J, Rasmussen CG (1974) [Principles for “self-retaining” plastic materials, and their application]. Tandlakartidningen 66:109–114

Goldbaum MH, McCuen BW, Hanneken AM, et al (1998) Silicone oil tamponade to seal macular holes without position restrictions. Ophthalmology 105:2140

Goldberg NL (1971) Biological considerations for a functional esthetic restoration of dysplastic and fractured anterior teeth in children. ASDC journal of dentistry for children 38:239–245

Golkari A, Sabokseir A, Pakshir H-R, et al (2011) A comparison of photographic, replication and direct clinical examination methods for detecting developmental defects of enamel. BMC oral health 11:16. <https://doi.org/10.1186/1472-6831-11-16>

Golombeski G.L., Kalscheur K.F., Hippen A.R., Schingoethe D.J. (2006) Slow-release urea and highly fermentable sugars in diets fed to lactating dairy cows. Journal of Dairy Science 89:4395–4403. <https://doi.org/10.3168/jds.S0022-0302(06)72486-9>

Gomes A.C., Messias L.P., Delbem A.C., Cunha R.F. (2010) Developmental disturbance of an unerupted permanent incisor due to trauma to its predecessor. Journal (Canadian Dental Association) 76:

Gomes AC, Neves LT das, Gomide MR (2009) Enamel defects in maxillary central incisors of infants with unilateral cleft lip. The Cleft palate-craniofacial journal : official publication of the American Cleft Palate-Craniofacial Association 46:420–424. <https://doi.org/10.1597/08-044.1>

Gomes B.C., Gold S.B., Baer P.N., Pesta S. (1979) Importance of combined periodontal and acid-etch composite treatment in restoration of anterior teeth and periodontal health. Journal of the American Dental Association (1939) 99:834–835. <https://doi.org/10.14219/jada.archive.1979.0395>

Gómez JF, López MMH (2012) Diagnóstico y Tratamiento de la hipomineralización incisivo molar. Revista Latinoamericana de Ortodoncia y Odontopediatría 19:

Gonçalves F de BDS (2011) Hipomineralização Incisivo-molar: prevalência e etiologia

Gonçalves SCD, Díaz-Serrano KV, de Queiroz AM, et al (2008) Gingival overgrowth in a renal transplant recipient using cyclosporine A. Journal of dentistry for children (Chicago, Ill) 75:313–317

Gong Y. (2008) The developmental history of the dental filling materials. Zhonghua yi shi za zhi (Beijing, China : 1980) 38:227–230

GONTAREVA K, KOSTYAKOVA T, AFANASYEVA V (2018) ANALYSIS OF INTERCONNECTION BETWEEN LOCAL HYPOPLASIA DEVELOPMENT AND METHODS FOR TREATING PERIODONTITIS IN TEMPORARY MOLARS. HEALTH CARE 33

Goodman AH, Armelagos GJ (1985) Factors affecting the distribution of enamel hypoplasias within the human permanent dentition. American journal of physical anthropology 68:479–493

Goodman A.H., Armelagos G.J. (1985) The chronological distribution of enamel hypoplasia in human permanent incisor and canine teeth. Archives of oral biology 30:503–507

Goodman AH, Martinez C, Chavez A (1991) Nutritional supplementation and the development of linear enamel hypoplasias in children from Tezonteopan, Mexico. The American journal of clinical nutrition 53:773–781

Goodman A.H., Rose J.C. (1990) Assessment of systemic physiological perturbations from dental enamel hypoplasias and associated histological structures. American Journal of Physical Anthropology 83:59–110

Goodwin AF, Tidyman WE, Jheon AH, et al (2014) Abnormal Ras signaling in Costello syndrome (CS) negatively regulates enamel formation. Human molecular genetics 23:682–692. <https://doi.org/10.1093/hmg/ddt455>

Gopalakrishnan SB, Viswanathan G (2012) Assessment of fluoride-induced changes on physicochemical and structural properties of bone and the impact of calcium on its control in rabbits. Journal of bone and mineral metabolism 30:154–163. <https://doi.org/10.1007/s00774-011-0312-6>

Gopinath VK, Al-Salihi KAM, Yean CY, et al (2004) Amelogenesis imperfecta: enamel ultra structure and molecular studies. The Journal of clinical pediatric dentistry 28:319–322

Gopinath VK, Yoong TP, Yean CY, Ravichandran M (2008) Identifying polymorphism in enamelin gene in amelogenesis imperfecta (AI). Archives of oral biology 53:937–940. <https://doi.org/10.1016/j.archoralbio.2008.03.007>

Gore DM, Iovieno A, Connell BJ, et al (2013) Peripheral hypertrophic subepithelial corneal degeneration: nomenclature, phenotypes, and long-term outcomes. Ophthalmology 120:892–898. <https://doi.org/10.1016/j.ophtha.2012.10.037>

Gorgani N., Sullivan R.E., DuBois L. (1990) A radiographic investigation of third-molar development. ASDC journal of dentistry for children 57:106–110

Gorski B, Gorska R, Wysokinska-Miszczuk J, Kaczynski T (2020) Tunnel technique with enamel matrix derivative in addition to subepithelial connective tissue graft compared with connective tissue graft alone for the treatment of multiple gingival recessions: a randomized clinical trial. Clinical oral investigations. <https://doi.org/10.1007/s00784-020-03312-6>

Goswami M, Bhushan U, Pandiyan R, Sharma S (2019) Molar Incisor Hypomineralization-An Emerging Burden: A Short Study on Prevalence and Clinical Characteristics in Central Delhi, India. International journal of clinical pediatric dentistry 12:211–214. <https://doi.org/10.5005/jp-journals-10005-1624>

Gotler M., Ratson T. (2010) [Molar incisor hypomineralization (MIH)--a literature review]. Refuat ha-peh Veha-shinayim (1993) 27:10–60

Goto G, Zhang Y (1996) Kinetic cavity preparation: protection of the cavo-surface enamel. The Journal of clinical pediatric dentistry 21:61–65

Gottberg B., Berne J., Quinonez B., Solorzano E. (2014) Prenatal effects by exposing to amoxicillin on dental enamel in wistar rats. Medicina Oral, Patologia Oral y Cirugia Bucal 19:. <https://doi.org/10.4317/medoral.18807>

Gottesfeld Z, Silverman PB (1990) Developmental delays associated with prenatal alcohol exposure are reversed by thyroid hormone treatment. Neuroscience letters 109:42–47

Goultschin J., Gazit D., Bichacho N., Bab I. (1988) Changes in teeth and gingiva of dogs following laser surgery: A block surface light microscopy study. Lasers in Surgery and Medicine 8:402–408. <https://doi.org/10.1002/lsm.1900080411>

Gound T.G., Maixner D. (2004) Nonsurgical management of a dilacerated maxillary lateral incisor with type III dens invaginatus: A case report. Journal of Endodontics 30:448–451. <https://doi.org/10.1097/00004770-200406000-00018>

Goward PE (1976) Enamel mottling in a non-fluoride community in England. Community dentistry and oral epidemiology 4:111–114

Goyal A, Dhareula A, Gauba K, Bhatia SK (2019) Prevalence, defect characteristics and distribution of other phenotypes in 3- to 6-year-old children affected with Hypomineralised Second Primary Molars. European archives of paediatric dentistry : official journal of the European Academy of Paediatric Dentistry 20:585–593. <https://doi.org/10.1007/s40368-019-00441-9>

Goyal S., Gugnani N. (2014) Assessment of skeletal maturation using mandibular second molar maturation stages. The Journal of clinical pediatric dentistry 39:79–84

Gräf W, Beimler HJ (1979) [Rate of dental caries in youths from communities of both naturally high and low, resp., fluoride content in drinking water (author’s transl)]. Zentralblatt fur Bakteriologie, Parasitenkunde, Infektionskrankheiten und Hygiene Erste Abteilung Originale Reihe B: Hygiene, Betriebshygiene, praventive Medizin 169:409–426

Grahnén H, Lysell L, Myrberg N, Ollinen P (1974) Fluoride, mineralisation defects of the enamel, and tooth width. Acta paediatrica Scandinavica 63:188–192

Granja MP (2018) Nathaly Vanessa Chávez Jaramillo

Grawish ME-A, Zaher AR, Elsabaa HM, Hegazy D (2011) Vasculitis of dental pulp associated with cryoglobulinemia in hepatitis C virus patients: case report. Journal of endodontics 37:1593–1595. <https://doi.org/10.1016/j.joen.2011.06.033>

Graziani F, Peric M, Marhl U, et al (2020) Local application of enamel matrix derivative prevents acute systemic inflammation after periodontal regenerative surgery: a randomized controlled clinical trial. Journal of clinical periodontology

Greco A., Podrini C., Williams R., Vinciguerra M. (2013) Histone variant macroH2A1 isoform have distinct effects on lipid accumulation in hepatocytes. Journal of Hepatology 58:. <https://doi.org/10.1016/S0168-8278%2813%2961287-8>

Greenberg MS, Brightman VJ, Lynch MA, Ship II (1969) Idiopathic hypoparathyroidism, chronic candidiasis, and dental hypoplasia. Oral surgery, oral medicine, and oral pathology 28:42–53

Greenfield B.A. (2012) Enamel defect restoration of the left mandibular first molar tooth. Journal of veterinary dentistry 29:36–43

Greenfield R, Iacono V, Zove S, Baer P (1992) Periodontal and prosthodontic treatment of amelogenesis imperfecta: a clinical report. The Journal of prosthetic dentistry 68:572–574

Greenwall L (2017) 11 MOLAR INCISOR HYPOPLASIA. Tooth Whitening Techniques 11

Greenwall L Bright opportunities–Dr Linda Greenwall–The British Dental Conference and Dentistry Show 2019

Greenwall-Cohen J, Greenwall L, Haywood V, Harley K (2018) Tooth whitening for the under-18-year-old patient. British dental journal 225:19–26

Gregg A., Crawford W., Crowther M., et al (2019) Systematic Review of Community Paramedicine and EMS Mobile Integrated Health Care Interventions in the United States. Population Health Management 22:213–222. <https://doi.org/10.1089/pop.2018.0114>

Grenier P, Brauner M (2010) [Imaging of fibrosing interstitial pneumonias]. Bulletin de l’Academie nationale de medecine 194:353

Gresky J. (2011) First examinations on neolithic human bones from Gobekli Tepe, Turkey. American Journal of Physical Anthropology 144:150. <https://doi.org/10.1002/ajpa.21502>

Grgic A, Lausberg H, Heinrich M, et al (2008) Progression of fibrosis in usual interstitial pneumonia: serial evaluation of the native lung after single lung transplantation. Respiration; international review of thoracic diseases 76:139–145

Grimard B, Hoidal M, Mills M, et al (2009) Comparison of clinical, periapical radiograph, and cone-beam volume tomography measurement techniques for assessing bone level changes following regenerative periodontal therapy. Journal of periodontology 80:48‐55. <https://doi.org/10.1902/jop.2009.080289>

Grin D (2000) Creating esthetic composite restorations: Part II, Crown fabrication. Journal of dental technology : the peer-reviewed publication of the National Association of Dental Laboratories 17:16–19

Grin D. (2000) Creating esthetic composite restorations. Journal of dental technology : the peer-reviewed publication of the National Association of Dental Laboratories 17:13–16

Grošelj M, Jan J (2013) Molar incisor hypomineralisation and dental caries among children in Slovenia. European journal of paediatric dentistry 14:241–245

Grossi J de A (2016) Tratamento restaurador atraumático como opção restauradora para hipomineralização molar-incisivo

Grossi J.A., Cabral R.N., Leal S.C. (2017) Caries Experience in Children with and without Molar-Incisor Hypomineralisation: A Case-Control Study. Caries research 51:419–424. <https://doi.org/10.1159/000477099>

Grusovin M.G., Esposito M. (2009) The efficacy of enamel matrix derivative (Emdogain) for the treatment of deep infrabony periodontal defects: a placebo-controlled randomised clinical trial. European journal of oral implantology 2:43–54

Gu PL, Yu KL, Chan SM (2000) Molecular characterization of an additional shrimp hyperglycemic hormone: cDNA cloning, gene organization, expression and biological assay of recombinant proteins. FEBS letters 472:122–128

Guandalini S., Gupta P. (2002) Celiac disease - A diagnostic challenge with many facets. Clinical and Applied Immunology Reviews 2:293–305. <https://doi.org/10.1016/S1529-1049%2802%2900052-1>

Guangzhi F., Hongyu F., Penghua M. (2019) Different materials and different methods for repairing an isolated tooth defect model: A three-dimensional finite element analysis of stress distribution. Chinese Journal of Tissue Engineering Research 23:870–876. <https://doi.org/10.3969/j.issn.2095-4344.1557>

Guatelli-Steinberg D (2000) Linear enamel hypoplasia in gibbons (Hylobates lar carpenteri). American journal of physical anthropology 112:395–410

Guatelli-Steinberg D (2003) Macroscopic and microscopic analyses of linear enamel hypoplasia in Plio-Pleistocene South African hominins with respect to aspects of enamel development and morphology. American journal of physical anthropology 120:309–322

Guatelli-Steinberg D, Ferrell RJ, Spence J (2012) Linear enamel hypoplasia as an indicator of physiological stress in great apes: reviewing the evidence in light of enamel growth variation. American journal of physical anthropology 148:191–204. <https://doi.org/10.1002/ajpa.21619>

Guatelli-Steinberg D, Larsen CS, Hutchinson DL (2004) Prevalence and the duration of linear enamel hypoplasia: a comparative study of Neandertals and Inuit foragers. Journal of human evolution 47:65–84

Guatelli-Steinberg D, Lukacs JR (1998) Preferential expression of linear enamel hypoplasia on the sectorial premolars of rhesus monkeys (Macaca mulatta). American journal of physical anthropology 107:179–186

Guatelli-Steinberg D., Lukacs J.R. (1999) Interpreting sex differences in enamel hypoplasia in human and non-human primates: Developmental, environmental, and cultural considerations. American journal of physical anthropology 73–126

Guatelli-Steinberg D., O’Hara M.C., McGraw W.S., et al (2018) Do Mid-Crown Enamel Formation Front Angles Reflect Factors Linked to the Pace of Primate Growth and Development? Anatomical Record 301:125–139. <https://doi.org/10.1002/ar.23703>

Guatelli-Steinberg D, Skinner M (2000) Prevalence and etiology of linear enamel hypoplasia in monkeys and apes from Asia and Africa. Folia primatologica; international journal of primatology 71:115–132

Guclu M., Cangul H., Ersoy C. (2015) APECED sendromu tanisi konulan turk ve avrupali hastalarda guclu benzerliklerStrong similarities in Turkish and European patients diagnosed with APECED syndrome. Turkish Journal of Endocrinology and Metabolism 19:89–92. <https://doi.org/10.4274/tjem.2987>

Guedes K.M.A., Guimaraes A.M.D.N., Bastos A.S., et al (2015) Stomatognathic evaluation at five years of age in children born premature and at term. BMC Pediatrics 15:. <https://doi.org/10.1186/s12887-015-0343-6>

Guentsch A, Seidler K, Nietzsche S, et al (2012) Biomimetic mineralization: long-term observations in patients with dentin sensitivity. Dental materials 28:457‐464. <https://doi.org/10.1016/j.dental.2012.01.003>

Guergolette RP, Dezan CC, Frossard WTG, et al (2009a) Prevalence of developmental defects of enamel in children and adolescents with asthma. Jornal Brasileiro de Pneumologia 35:295–300

Guergolette RP, Dezan CC, Frossard WTG, et al (2009b) Prevalência do desenvolvimento de defeitos no esmalte dentário em crianças e adolescentes com asma. Jornal Brasileiro de Pneumologia 35:295–300

Guergolette R.P., Ferreira F.B.A., Fernandes K.B.P., et al (2009) Prevalencia de defeitos do desenvolvimento do esmalte dentario em criancas e adolescentes com asmaPrevalence of developmental defects of enamel in children and adolescents with asthma. Jornal Brasileiro de Pneumologia 35:295–300

Gugnani N, Pandit I, Gupta M, et al (2017) Comparative evaluation of esthetic changes in nonpitted fluorosis stains when treated with resin infiltration, in-office bleaching, and combination therapies. Journal of esthetic and restorative dentistry 29:317‐324. <https://doi.org/10.1111/jerd.12312>

Gugnani N, Pandit IK, Goyal V, et al (2014) Esthetic improvement of white spot lesions and non-pitted fluorosis using resin infiltration technique: series of four clinical cases. Journal of the Indian Society of Pedodontics and Preventive Dentistry 32:176–180. <https://doi.org/10.4103/0970-4388.130996>

Gugnani N, Pandit IK, Gupta M, Josan R (2012) Caries infiltration of noncavitated white spot lesions: A novel approach for immediate esthetic improvement. Contemporary clinical dentistry 3:S199. <https://doi.org/10.4103/0976-237X.101092>

Guida L, Annunziata M, Belardo S, et al (2007) Effect of autogenous cortical bone particulate in conjunction with enamel matrix derivative in the treatment of periodontal intraosseous defects. Journal of periodontology 78:231‐238. <https://doi.org/10.1902/jop.2007.060142>

Guimaraes L.F., Dias P.F., Janini M.E., de Souza I.P. (2008) Langerhans cell histiocytosis: impact on the permanent dentition after an 8-year follow-up. Journal of dentistry for children (Chicago, Ill) 75:64–68

Guinee TP, O’Callaghan DJ (2013) Effect of increasing the protein-to-fat ratio and reducing fat content on the chemical and physical properties of processed cheese product. Journal of dairy science 96:6830–6839. <https://doi.org/10.3168/jds.2013-6685>

Guler C, Keskin G (2014) Dental findings in Hamamy syndrome. Genetic counseling 25:383

Guler C., Keskin G. (2015) Dental findings in hamamy syndrome. Genetic Counseling 25:383–388

Gunen Yilmaz S, Yilmaz F, Bayrakdar IS, Harorli A (2019) The Relationship between carotid artery calcification and pulp stone among hemodialysis patients: A retrospective study. Saudi journal of kidney diseases and transplantation : an official publication of the Saudi Center for Organ Transplantation, Saudi Arabia 30:755–763. <https://doi.org/10.4103/1319-2442.265449>

Guner S, Salcioglu D (2016) Current View on the Diagnosis and Treatment of Molar Incisor Hypomineralization. CLINICAL AND EXPERIMENTAL HEALTH SCIENCES 6:28–34

Güner Ş, Salcıoğlu D (2016) Büyük azı keser hipomineralizasyonu’na güncel bakış: Teşhis ve tedavi yaklaşımları

Güngör HC, Püşman E, Uysal S (2011) Eruption delay and sequelae in permanent incisors following intrusive luxation in primary dentition: a case report. Dental traumatology : official publication of International Association for Dental Traumatology 27:156–158. <https://doi.org/10.1111/j.1600-9657.2011.00981.x>

Gungor O, Erdogan Y, Gungor A, Alkis H (2016) In vitro evaluation of microleakage of class V cavities restored with new flowable compomers on the primary teeth. International journal of artificial organs 39:132‐135. <https://doi.org/10.5301/ijao.5000486>

Guo Z-L, Gan S-L, Cao C-Y, et al (2019) Advanced glycosylated end products restrain the osteogenic differentiation of the periodontal ligament stem cell. Journal of dental sciences 14:146–151. <https://doi.org/10.1016/j.jds.2019.03.007>

Gupta D (2018) Dentofacial Medicine and Diagnosis. The open dentistry journal 12:696

Gupta G, Rana V, Srivastava N, Chandna P (2015) Laser pulpotomy-an effective alternative to conventional techniques: a 12 months clinicoradiographic study. Jaypees international journal of clinical pediatric dentistry 8:18‐21

Gupta KP, Grewal PS, Garg S (2011) Clinical challenges in managing minor developmental anomalies in children. J of Oral Health Community Dentistry 5:136–141

Gupta R.K., Archambeault D.R., Singh J.M., et al (2010) Exposure to low dose bisphenol-a affects ovarian development in the mouse. Biology of Reproduction 83:

Gupta S., Das A., Murphy C., Dunn L. (2011) A case of massive obstetric haemorrhage controlled by hydrostatic rusch balloon. Irish Journal of Medical Science 180:. <https://doi.org/10.1007/s11845-011-0697-1>

Gupta S, Jhingran R, Gupta V, et al (2014a) Efficacy of platelet-rich fibrin vs. enamel matrix derivative in the treatment of periodontal intrabony defects: a clinical and cone beam computed tomography study. Journal of the international academy of periodontology 16:86‐96

Gupta S.J., Jhingran R., Gupta V., et al (2014) Efficacy of platelet-rich fibrin vs. enamel matrix derivative in the treatment of periodontal intrabony defects: a clinical and cone beam computed tomography study. Journal of the International Academy of Periodontology 16:86–96

Gupta SP, Shetty PP, Reddy K, Sancheti P (2014b) Enamel Hypoplasia: A Case Report. Journal of Advanced Oral Research 5:10–13

Gurinsky B, Mills M, Mellonig J (2004) Clinical evaluation of demineralized freeze-dried bone allograft and enamel matrix derivative versus enamel matrix derivative alone for the treatment of periodontal osseous defects in humans. Journal of periodontology 75:1309‐1318. <https://doi.org/10.1902/jop.2004.75.10.1309>

Gusmão TP de L, Faria ABS de, Leão Filho JC, et al (2020) Dental changes in children with congenital Zika syndrome. Oral diseases 26:457–464. <https://doi.org/10.1111/odi.13238>

Güth J-F, Kauling AEC, Schweiger J, et al Virtual Simulation of Periodontal Surgery Including Presurgical CAD/CAM Fabrication of Tooth-Colored Removable Splints on the Basis of CBCT Data: A Case Report. The International journal of periodontics & restorative dentistry 37:e310. <https://doi.org/10.11607/prd.2769>

Gutierrez Gossweiler A., Martinez-Mier E.A. (2020) Chapter 6: Vitamins and Oral Health. Monographs in oral science 28:59–67. <https://doi.org/10.1159/000455372>

Gutiérrez HM, Barrientos AP MANEJO DE PACÍENTES CON TRASTORNO DEL ESPECTRO AUTÍSTA DURANTE LA ATENCÍó N DENTAL. IV Jornadas Odontológicas del Archipiélago de Chiloé 27

Gutierrez M, Mellonig J, Cochran D (2003) Evaluation of enamel matrix derivative as an adjunct to non-surgical periodontal therapy. Journal of clinical periodontology 30:739‐745. <https://doi.org/10.1034/j.1600-051x.2003.00374.x>

Gutiérrez TV, Ortega CCB, Pérez NP, Pérez AG (2019) Impact of Molar Incisor Hypomineralization on Oral Health-Related Quality of Life in Mexican Schoolchildren. Journal of Clinical Pediatric Dentistry 43:324–330

Guven Y. (2020) Ectodermal dysplasias and treatment approaches: A dentist’s perspective. Gazi Medical Journal 31:

Gwal K., Mallon M., States L. (2011) Radiologic survey of brittle bones in neonates. Pediatric Radiology 41:. <https://doi.org/10.1007/s00247-011-2025-3>

Haak R, Hähnel M, Schneider H, et al (2019) Clinical and OCT outcomes of a universal adhesive in a randomized clinical trial after 12 months. Journal of dentistry 90:103200. <https://doi.org/10.1016/j.jdent.2019.103200>

Haak R, Nake T, Park K-J, et al (2018a) Internal and marginal adaptation of high-viscosity bulk-fill composites in class II cavities placed with different adhesive strategies. Odontology. <https://doi.org/10.1007/s10266-018-0402-1>

Haak R, Schmidt P, Park K, et al (2018b) OCT for early quality evaluation of tooth-composite bond in clinical trials. Journal of dentistry 76:46‐51

Haavikko K, Helle A (1974) The prevalence and distribution of enamel defects in four districts with different fluoride contents in the drinking water. Proceedings of the Finnish Dental Society Suomen Hammaslaakariseuran toimituksia 70:178–185

Habib SR, Vohra FA, MClinDent M Treatment Modalities For Ectodermal Dysplasia Patients At A Tertiary Care Hospital

Hafer M, Jentsch H, Haak R, Schneider H (2015) A three-year clinical evaluation of a one-step self-etch and a two-step etch-and-rinse adhesive in non-carious cervical lesions. Journal of dentistry 43:350‐361

Hägewald S, Spahr A, Rompola E, et al (2002) Comparative study of Emdogain and coronally advanced flap technique in the treatment of human gingival recessions. A prospective controlled clinical study. Journal of clinical periodontology 29:35‐41. <https://doi.org/10.1034/j.1600-051x.2002.290106.x>

Haghnegahdar AA, Bronoosh P, Khojastepour L, Tahmassebi P (2014) Prevalence of bifid mandibular condyle in a selected population in South of iran. Journal of dentistry (Shiraz, Iran) 15:156–160

Hahn C, Cahuana A, da Silva JM, et al (2013) Exodoncia terapéutica del primer molar permanente con hipomineralización incisivo molar severa. Revisión de la literatura. Odontología pediátrica 21:131–138

Hahn C, Palma C (2012) Hipomineralización incisivo-molar: de la teoría a la práctica. Odontología pediátrica 11:136–144

Hahn Chacón C, Cahuana Cárdenas AB, Mendes da Silva J, et al (2013) Exodoncia terapéutica del primer molar permanente con hipomineralización incisivo molar severa. Revisión de la literatura. Odontología Pediátrica, 2013, vol 21, num 2, p 131-138

Hahn J.S., Hahn S.M., Kammann H., et al (2005) Endocrine disorders associated with holoprosencephaly. Journal of Pediatric Endocrinology and Metabolism 18:935–941. <https://doi.org/10.1515/JPEM.2005.18.10.935>

Haidary S (2014) Comparison of the Prevalence of Molar Incisor Hypomineralization in Dubai/United Arab Emirate and Greifswald/Germany

Hakki SS, Celenligil-Nazliel H, Karaduman A, et al (2001) Epidermolysis bullosa acquisita: clinical manifestations, microscopic findings, and surgical periodontal therapy. A case report. Journal of periodontology 72:550–558

Halabi R.S., Miller K.B., Grossman C.E., et al (2010) A fatal case of pulmonary Kaposi Sarcoma-associated immune reconstitution syndrome in the absence of mucocutaneous lesions. Chest 138:. <https://doi.org/10.1378/chest.10915>

Halal F, Raslan N (2020) Prevalence of hypomineralised second primary molars (HSPM) in Syrian preschool children. European Archives of Paediatric Dentistry: Official Journal of the European Academy of Paediatric Dentistry

Halal R, Nohra J, Akel H (2018) Conservative anterior treatment with CAD-CAM technology and polymer-infiltrated ceramic for a child with amelogenesis imperfecta: A 2-year follow-up. The Journal of prosthetic dentistry 119:710–712. <https://doi.org/10.1016/j.prosdent.2017.07.018>

Halbleib K Labor-Kompositversorgungen bei einer sechsjährigen Patientin

Hali H., Nahvi A. (2016) Hypoplastic amelogenesis imperfecta: A case report. Journal of Mazandaran University of Medical Sciences 25:346–351

Halim RA (2017) Molar-Incisor Hypomineralisa-tion: What Do We Know?

Hallas D, Herman NG, Benichou L, et al (2015) Management of a child with nutritional rickets, multiple cavities, enamel hypoplasia, and reactive attachment disorder. Journal of pediatric health care : official publication of National Association of Pediatric Nurse Associates & Practitioners 29:283–288. <https://doi.org/10.1016/j.pedhc.2014.11.010>

Haller B, Trojanski A (1998) Effect of multi-step dentin bonding systems and resin-modified glass ionomer cement liner on marginal quality of dentin-bonded resin composite Class II restorations. Clinical oral investigations 2:130–136

Hallet J., Soler L., Mutter D., et al (2015) Trans-thoracic minimally invasive segment 8 liver resection guided by augmented reality. HPB 17:3. <https://doi.org/10.1111/hpb.12399_2/abstract>

Hallett K.B., Hall R.K. (1995) Congenital adrenal hyperplasia and enamel hypoplasia: case report. Pediatric dentistry 17:54–59

Hallett KB, Lucas JO, Johnston T, et al (1995) Dental health of children with cerebral palsy following sialodochoplasty. Special care in dentistry : official publication of the American Association of Hospital Dentists, the Academy of Dentistry for the Handicapped, and the American Society for Geriatric Dentistry 15:234–238

Hallett K.B., Radford D.J., Seow W.K. (1992) Oral health of children with congenital cardiac diseases: a controlled study. Pediatric dentistry 14:224–230

Hals E, Norderval IT (1973) Histopathology of experimental in vivo caries around silicate fillings. Acta odontologica Scandinavica 31:357–367

Hamza M, Chlyah A, Bousfiha B, et al (2019) Pathology and Abnormality of the First Permanent Molar among Children. In: Human Teeth-Key Skills and Clinical Illustrations. IntechOpen

Han XL, Liu M, Voisey A, et al (2011) Post-natal effect of overexpressed DKK1 on mandibular molar formation. Journal of dental research 90:1312–1317. <https://doi.org/10.1177/0022034511421926>

Handzel J (1979) [Treatment of teeth damaged by tetracycline antibiotics]. Ceskoslovenska pediatrie 34:528–531

Haney JM, Nilvéus RE, McMillan PJ, Wikesjö UM (1993) Periodontal repair in dogs: expanded polytetrafluoroethylene barrier membranes support wound stabilization and enhance bone regeneration. Journal of periodontology 64:883–890

Haney K.L. (2007) Trauma to the primary dentition. Journal - Oklahoma Dental Association 99:42–44

Hanlin SM, Burbridge LA, Drummond BK (2015) Restorative management of permanent teeth enamel defects in children and adolescents. In: Planning and Care for Children and Adolescents with Dental Enamel Defects. Springer, pp 139–155

Hanna B., Rajagopalan S. (2010) Reiger anomaly due to a FOXC1 gene deletion: Look beyond the eyes. Twin Research and Human Genetics 13:642

Hanna L.M.O., De Araujo R.J.G., Gomes L.C.O., et al (2009) Microabrasion of the enamel to soften the hypoplasia. Annals of Tropical Medicine and Public Health 2:53–56

Hannibal DL (2017) Prevalence of an unusual hypoplastic defect of the permanent maxillary lateral incisor in great apes. American journal of primatology 79:1–9. <https://doi.org/10.1002/ajp.22594>

Hansrani M, Stanford J, McIntyre G, et al (2010) Immunotherapy for the prevention of myointimal hyperplasia after experimental balloon injury of the rat carotid artery. Angiology 61:437–442. <https://doi.org/10.1177/0003319710366128>

Harika R, Dutta B, Arun P, Teja RP (2016) A novel clinical approach for long-term retention and durability of resin restorations bonded to multiple developmental defects of enamel. Journal of International Society of Preventive & Community Dentistry 6:597–601. <https://doi.org/10.4103/2231-0762.195507>

Harley K (2014) The restorative management of developmental enamel defects. Annals of the Royal Australasian College of Dental Surgeons 22:64

Harley KE, Ibbetson RJ (1993) Dental anomalies--are adhesive castings the solution? British dental journal 174:15–22

Harne PS, Rao S, Malik M, Shepherd Z (2020) Acute Eosinophilic Pneumonia Secondary to Menthol Cigarette Use: A Rare Phenomenon With a Review of Literature. Journal of investigative medicine high impact case reports 8:2324709620925978. <https://doi.org/10.1177/2324709620925978>

Haro Durand L.A., Gorustovich A.A., Mesones R.V., Nielsen F.H. (2010) Histomorphometric and microchemical characterization of maturing dental enamel in rats fed a boron-deficient diet. Biological Trace Element Research 135:242–252. <https://doi.org/10.1007/s12011-009-8512-9>

Harrel SK, Wilson TG, Nunn ME (2005) Prospective assessment of the use of enamel matrix proteins with minimally invasive surgery. Journal of periodontology 76:380–384

Harris RJ, Harris LE, Harris CR, Harris AJ (2007) Clinical evaluation of a combined regenerative technique with enamel matrix derivative, bone grafts, and guided tissue regeneration. The International journal of periodontics & restorative dentistry 27:171–179

Harryparsad A, Rahman L, Bunn BK (2013) Amelogenesis imperfecta: a diagnostic and pathological review with case illustration. SADJ : journal of the South African Dental Association = tydskrif van die Suid-Afrikaanse Tandheelkundige Vereniging 68:404–407

Hart TC, Hart PS (2009) Genetic studies of craniofacial anomalies: clinical implications and applications. Orthodontics & craniofacial research 12:212–220. <https://doi.org/10.1111/j.1601-6343.2009.01455.x>

Harte C., Juggins K. (2013) Triplets with bilateral palatally displaced canines in association with third molar agenesis: An example of biologically related dental anomaly patterns? Journal of Orthodontics 40:172–177. <https://doi.org/10.1179/1465312513Z.00000000081>

Hartsfield Jr JK, Cameron AC (2016) Acquired and Developmental Disturbances of the Teeth and Associated Oral Structures. In: McDonald and Avery’s Dentistry for the Child and Adolescent. Elsevier, pp 39–79

Hartsock L.A., Burnheimer J., Modesto A., Vieira A.R. (2020) A Snapshot of the Prevalence of Molar Incisor Hypomineralization and Fluorosis in Pittsburgh, Pennsylvania, USA. Pediatric dentistry 42:36–40

Hartwig AD, Azevedo MS, Pauli LA, et al (2019) An observational study of dental abnormalities in the primary teeth. RSBO 16:86–93

Harutunian K., Figueiredo R., Gay-Escoda C. (2011) Tuberous sclerosis complex with oral manifestations: A case report and literature review. Medicina Oral, Patologia Oral y Cirugia Bucal 16:. <https://doi.org/10.4317/medoral.16.e478>

Harvey A.R. (2012) The dog days of stress: A comparison of methods for determining age at occurrence of enamel hypoplasias. American Journal of Physical Anthropology 147:161. <https://doi.org/10.1002/ajpa.22033>

Hasan MMB Self-etching Adhesive Systems in Operative Dentistry: A Literature. system 22:23

Hasenauer L, Vogelsberger M, Bürkle V, et al (2010) Prävalenz und Ausprägung der Molar Incisor Hypomineralisation (MIH) in Salzburg und Tirol und ein Beitrag zur Erforschung der Ursachen. Stomatologie 107:43–50

Hashimoto M. (1990) Effects of Nd:YAG laser irradiation on acid resistance of defective rat enamel. Shoni shikagaku zasshi The Japanese journal of pedodontics 28:956–967

Hassan A.K., Saifi M.A.-B., Najim Z.N. (2002) Prevalence of dental flourosis in Fazan (South Libya). Journal of the Bahrain Medical Society 14:85–90

Hassett BR (2014) Missing defects? A comparison of microscopic and macroscopic approaches to identifying linear enamel hypoplasia. American journal of physical anthropology 153:463–472. <https://doi.org/10.1002/ajpa.22445>

Haukali G, Lundeberg S, Ostergaard BH (2017) Paediactric dentistry—a clinical approach

Hawas RA, Taha SE, Fouad WA (2014) Prevalence of visible enamel defects in permanent dentition among a group of Egyption children. DENTAL JOURNAL 60:981

Hayashi Y., Morinaga S., Zhang J., et al (2016) BK channels in microglia are required for morphine-induced hyperalgesia. Nature Communications 7:. <https://doi.org/10.1038/ncomms11697>

Hayashi-Sakai S., Numa-Kinjoh N., Sakamoto M., et al (2016) Hypophosphatasia: Evaluation of Size and Mineral Density of Exfoliated Teeth. The Journal of clinical pediatric dentistry 40:496–502. <https://doi.org/10.17796/1053-4628-40.6.496>

Hazar B, Avşar A (2019) Hipomineralizacija kutnjaka i sekutića kod dece sa sistemskim oboljenjima. Srpski arhiv za celokupno lekarstvo 147:19–24

Heasley J (2004) General field isolation rubber dams without operative inserts which isolate the dental alveolar arch for dental treatment

Heden G, Wennström J, Lindhe J (1999) Periodontal tissue alterations following Emdogain treatment of periodontal sites with angular bone defects. A series of case reports. Journal of clinical periodontology 26:855–860

Heden G., Wennstrom J., Lindhe J. (1999) Periodontal tissue alterations following Emdogain treatment of periodontal sites with angular bone defects. A series of case reports. Journal of clinical periodontology 26:855–860

Heden G., Wennstrom J.L. (2006) Five-year follow-up of regenerative periodontal therapy with enamel matrix derivative at sites with angular bone defects. Journal of Periodontology 77:295–301. <https://doi.org/10.1902/jop.2006.050071>

Hegde S. (2012) Multiple unerupted teeth with amelogenesis imperfecta in siblings. North American Journal of Medical Sciences 4:235–237. <https://doi.org/10.4103/1947-2714.95908>

Heijl L (1997) Periodontal regeneration with enamel matrix derivative in one human experimental defect. A case report. Journal of clinical periodontology 24:693–696

Heijl L., Heden G., Svardstrom G., Ostgren A. (1997) Enamel matrix derivative (EMDOGAIN) in the treatment of intrabony periodontal defects. Journal of clinical periodontology 24:705–714

Hein CM, Noack MJ, Roulet JF (1989) [Opacity of composites and hard tooth substance on X-ray]. Deutsche zahnarztliche Zeitschrift 44:536–539

Hein C.M., Noack M.J., Roulet J.F. (1989) Die Rontgenopazitat von Kompositmaterialien und ZahnhartsubstanzenOpacity of composites and hard tooth substance on X-ray. Deutsche zahnarztliche Zeitschrift 44:536–539

Heinrich-Weltzien R, Kühnisch J, van der Veen M, et al (2003) Quantitative light-induced fluorescence (QLF)--a potential method for the dental practitioner. Quintessence international (Berlin, Germany : 1985) 34:181–188

Heithersay GS (1994) External root resorption. Annals of the Royal Australasian College of Dental Surgeons 12:46–59

Heithersay G.S. (1999) Clinical, radiologic, and histopathologic features of invasive cervical resorption. Quintessence international (Berlin, Germany : 1985) 30:27–37

Heitmueller D, Thiering E, Hoffmann U, et al (2013) Is there a positive relationship between molar incisor hypomineralisations and the presence of dental caries? International journal of paediatric dentistry 23:116–124

Hejlesen J, Underbjerg L, Gjørup H, et al (2020) Dental anomalies and orthodontic characteristics in patients with pseudohypoparathyroidism. BMC oral health 20:1–9

Heldt JP, Zito MF, Seroussi A, et al (2019) A Medical Incapacity Hold Policy Reduces Inappropriate Use of Involuntary Psychiatric Holds While Protecting Patients From Harm. Psychosomatics 60:37–46. <https://doi.org/10.1016/j.psym.2018.06.002>

Hellemons ME, Moor CC, von der Thüsen J, et al (2020) Desquamative interstitial pneumonia: a systematic review of its features and outcomes. European respiratory review : an official journal of the European Respiratory Society 29:. <https://doi.org/10.1183/16000617.0181-2019>

Heller A. (2011) Clinical procedures to avoid the “dark halo” in restorations with direct composite resins (Introducing the concept of destructive interference in restorative dentistry). Dental update 38:304–312. <https://doi.org/10.12968/denu.2011.38.5.304>

Hermann NV, Darvann TA, Kreiborg S (2020) Delayed maturation and reduced crown width of the permanent first mandibular molar in all subgroups of cleft lip and palate. Orthodontics & craniofacial research

Hernández Juyol M, Boj Quesada JR, Espasa E, Peretz B (2018) Prevalencija molarno-incizivne hipomineralizacije u skupini panjolske kolske djece. Acta Stomatologica Croatica, 2018, vol 52, num 1, p 4-11

Hernández Juyol M, Muñoz S, López F, et al (2014) Prevalencia de la hipomineralización incisivo molar en una muestra de 772 escolares de la provincia de Barcelona. Odontología Pediátrica, 2014, vol 22, num 2, p 115-125

Hernandez M, Boj J, Espasa E (2016) Do we really know the prevalence of MIH? Journal of Clinical Pediatric Dentistry 40:259–263

Hernandez M, Boj J, Espasa E, et al (2018) Molar-incisor hypomineralization: positive correlation with atopic dermatitis and food allergies. Journal of Clinical Pediatric Dentistry 42:344–348

HERNÁNDEZ M, BOJ J, ESPASA E, et al (2020) La dermatitis atópica como nuevo factor etiológico en el origen de la hipomineralización incisivo-molar. ODONTOL PEDIÁTR 28:14–24

Hernández M, Boj J-R, Espasa E, Peretz B (2018) Prevalencija molarno-incizivne hipomineralizacije u skupini španjolske školske djece. Acta stomatologica Croatica 52:4–11

Hernandez M., Droz D., Phulpin B., Mansuy L. (2017) Use of new targeted cancer therapies in children: effects on dental development and risk of jaw osteonecrosis: a review. Journal of Oral Pathology and Medicine 46:321–326. <https://doi.org/10.1111/jop.12516>

Hernández M, Planells P, Martínez E, et al (2020) Microbiology of molar-incisor hypomineralization lesions. A pilot study. Journal of oral microbiology 12:1766166. <https://doi.org/10.1080/20002297.2020.1766166>

Hernandez M, Pochon C, Chastagner P, Droz D (2019) Long-term Adverse Effects of Acute Myeloid Leukemia Treatment on Odontogenesis in a Child. International Journal of Clinical Pediatric Dentistry 12:243

Hertzberg J., Nakisbendi L., Needleman H.L., Pober B. (1994) Williams syndrome--oral presentation of 45 cases. Pediatric dentistry 16:262–267

Heywood BR, Eanes ED (1987) An ultrastructural study of calcium phosphate formation in multilamellar liposome suspensions. Calcified tissue international 41:192–201

Hickel R, Voss A (1989) Comparative studies on fissure sealing: composite versus Cermet cement. Deutsche zahnarztliche zeitschrift 44:472‐474

Hicks J., Metry D.W., Barrish J., Levy M. (2001) Uncombable hair (cheveux incoiffables, pili trianguli et canaliculi) syndrome: Brief review and role of scanning electron microscopy in diagnosis. Ultrastructural Pathology 25:99–103. <https://doi.org/10.1080/01913120117514>

Hilgert LA, Leal SC (2016) Resin Infiltration: A Microinvasive Treatment for Carious and Hypomineralised Enamel Lesions. In: Evidence-Based Caries Prevention. Springer, pp 123–141

Hill I.D., Fasano A., Guandalini S., et al (2016) NASPGHAN clinical report on the diagnosis and treatment of gluten-related disorders. Journal of Pediatric Gastroenterology and Nutrition 63:156–165. <https://doi.org/10.1097/MPG.0000000000001216>

Hiller KA, Wilfart G, Schmalz G (1998) Developmental enamel defects in children with different fluoride supplementation--a follow-up study. Caries research 32:405–411

Hillson S., Antoine D. (2011) The mechanisms that produce the defects of enamel hypoplasia. American Journal of Physical Anthropology 144:163. <https://doi.org/10.1002/ajpa.21502>

Hillson S, Bond S (1997) Relationship of enamel hypoplasia to the pattern of tooth crown growth: a discussion. American journal of physical anthropology 104:89–103

Hillson SW (1992) Dental enamel growth, perikymata and hypoplasia in ancient tooth crowns. Journal of the Royal Society of Medicine 85:460–466

Himi M, Fujimaki T, Yokoyama T, et al (2009) A case of oculodentodigital dysplasia syndrome with novel GJA1 gene mutation. Japanese journal of ophthalmology 53:541–545. <https://doi.org/10.1007/s10384-009-0711-6>

Hirai T, Yamashita M, Yoshikuni M, et al (1992) Cyclin B in fish oocytes: its cDNA and amino acid sequences, appearance during maturation, and induction of p34cdc2 activation. Molecular reproduction and development 33:131–140

Hiraishi N, Yiu CKY, King NM (2008) Effect of acid etching time on bond strength of an etch-and-rinse adhesive to primary tooth dentine affected by amelogenesis imperfecta. International journal of paediatric dentistry 18:224–230. <https://doi.org/10.1111/j.1365-263X.2007.00909.x>

Hirasaki S., Murakami K., Mizushima T., et al (2012) Successful treatment of sepsis caused by Staphylococcus lugdunensis in an adult with 22q11.2 deletion syndrome. Internal Medicine 51:377–380. <https://doi.org/10.2169/internalmedicine.51.6257>

Hirasuna K, Fried D, Darling CL (2008) Near-infrared imaging of developmental defects in dental enamel. Journal of biomedical optics 13:044011. <https://doi.org/10.1117/1.2956374>

Hiremath H, Yakub SS, Metgud S, et al (2007) Invasive cervical resorption: a case report. Journal of endodontics 33:999–1003

Hirschfeld Z, Bab I, Tamari I, Sela J (1982) Primary mineralization of dentin in rats after pulp capping with calcium-hydroxide. Journal of oral pathology 11:426–433

Hirschfelder U., Iserhardt N. (2003) Kieferorthopadische Versorgung von Lippen-Kiefer-Gaumenspalten. 3. Versorgung nach Abschluss der PrimarbehandlungOrthodontic management of cheilognatho-palatoschisis. 3. Further care following completion of primary treatment. Padiatrische Praxis 64:137–154

HITCHIN AD, NAYLOR MN (1964) ACUTE MAXILLITIS OF INFANCY. LATE SEQUELAE OF THREE CASES, INCLUDING A RHINOLITH CONTAINING A TOOTH AND A COMPOUND COMPOSITE ODONTOME. Oral surgery, oral medicine, and oral pathology 18:423–431

Hlusko LJ, Do N, Mahaney MC (2007) Genetic correlations between mandibular molar cusp areas in baboons. American journal of physical anthropology 132:445–454

Hoac B., Murshed M., Mckee M.D., et al (2018) Ablation of osteopontin in osteomalacic hyp mice partially rescues the deficient mineralization without correcting hypophosphatemia. Journal of Bone and Mineral Research 33:1

Hocevar L., Kovac J., Podkrajsek K.T., et al (2020) The possible influence of genetic aetiological factors on molar-incisor hypomineralisation. Archives of oral biology 118:104848. <https://doi.org/10.1016/j.archoralbio.2020.104848>

Hočevar L, Kovač J, Podkrajšek KT, et al (2020a) Dataset on amelogenesis-related genes variants (ENAM and ENAM interacting genes) and on human leukocyte antigen alleles (DQ2 and DQ8) distribution in children with and without molar-incisor hypomineralisation (MIH). Data in Brief 106224

Hočevar L, Kovač J, Podkrajšek KT, et al (2020b) The possible influence of genetic aetiological factors on molar–incisor hypomineralisation. Archives of Oral Biology 118:104848

Hodges DC, Wilkinson RG (1990) Effect of tooth size on the ageing and chronological distribution of enamel hypoplastic defects. American journal of human biology : the official journal of the Human Biology Council 2:553–560. <https://doi.org/10.1002/ajhb.1310020511>

Hodges SJ, Spencer RJ, Watkins SJ (2000) Unusual indelible enamel staining following fixed appliance treatment. Journal of orthodontics 27:303–306

Hoffmann T, Al-Machot E, Meyle J, et al (2016) Three-year results following regenerative periodontal surgery of advanced intrabony defects with enamel matrix derivative alone or combined with a synthetic bone graft. Clinical oral investigations 20:357‐364. <https://doi.org/10.1007/s00784-015-1522-4>

Hoffmann T, Noack B, Marzinek A, Wiedemann B (2003) Controlled clinical study of the efficacy of enamel matrix derivatives compared with barriers in the treatment of buccal Class I and II recession defects - the role of influencing parameters. Journal of dental research 82:B‐385, Abstract no: 3007

Hoffmann T, Richter S, Meyle J, et al (2006) A randomized clinical multicentre trial comparing enamel matrix derivative and membrane treatment of buccal class II furcation involvement in mandibular molars. Part III: patient factors and treatment outcome. Journal of clinical periodontology 33:575‐583. <https://doi.org/10.1111/j.1600-051X.2006.00947.x>

Hoffmann U, Neumann C, Bauer C-P, et al (2013) Respiratory diseases are associated with molar-incisor hypomineralizations

Hogan R, Goodwin M, Boothman N, et al (2018) Further opportunities for digital imaging in dental epidemiology. Journal of dentistry 74:S2–S9

Hoidal M, Grimard B, Mills M, et al (2008) Clinical evaluation of demineralized freeze-dried bone allograft with and without enamel matrix derivative for the treatment of periodontal osseous defects in humans. Journal of periodontology 79:2273‐2280. <https://doi.org/10.1902/jop.2008.080259>

Højgaard K (1980) Dentition on Bahrain, 2000 B.C. Scandinavian journal of dental research 88:467–475

Hojgaard K. (1980) Dentition on Bahrain, 2000 B.C. Scandinavian journal of dental research 88:467–475

Holan G. (2006) Long-term effect of different treatment modalities for traumatized primary incisors presenting dark coronal discoloration with no other signs of injury. Dental Traumatology 22:14–17. <https://doi.org/10.1111/j.1600-9657.2006.00346.x>

Holan G, Eidelman E, Fuks A (2005) Long-term evaluation of pulpotomy in primary molars using mineral trioxide aggregate or formocresol. Pediatric dentistry 27:129‐136

Holan G., Topf J., Fuks A.B. (1992) Effect of root canal infection and treatment of traumatized primary incisors on their permanent successors. Endodontics & dental traumatology 8:12–15

Holla G., Ramakrishna Y., Holla A., Munshi A.K. (2014) Solitary median maxillary central incisor syndrome associated with unique cleft palate: a rare case report. General dentistry 62:

Hollender L, Koch G (1976) Effect of local application of fluoride on initial demineralization of buccal surface of maxillary incisors. Clinical assessment from colour slides. Svensk tandlakare tidskrift Swedish dental journal 69:1–5

Holloway PJ, Ellwood RP (1997) The prevalence, causes and cosmetic importance of dental fluorosis in the United Kingdom: a review. Community dental health 14:148–155

Holm AK, Andersson R (1982) Enamel mineralization disturbances in 12-year-old children with known early exposure to fluorides. Community dentistry and oral epidemiology 10:335–339

Holm A.K., Arvidsson S. (1974) Oral health in preschool Swedish children. I. Three year old children. 25:81–98

Holt R, Morris C, Winter G, Downer M (1994) Enamel opacities and dental caries in children who used a low fluoride toothpaste between 2 and 5 years of age. International dental journal 44:331‐341

Holt RD, Winter GB, Fox B, Askew R (1990) Enamel opacities in children whose mothers took part in a dental health education scheme. Community dentistry and oral epidemiology 18:74–76

Holt VP, Earp DP (2000) Adhesive solutions: report of a case using multiple adhesive techniques in the management of enamel hypoplasia. Dental update 27:153

Holt V.P., Earp D.P. (2000) Adhesive solutions: report of a case using multiple adhesive techniques in the management of enamel hypoplasia. Dental update 27:153

Holtgrave EA, Hopfenmüller W, Ammar S (2001) Tablet fluoridation influences the calcification of primary tooth pulp. Journal of orofacial orthopedics = Fortschritte der Kieferorthopadie : Organ/official journal Deutsche Gesellschaft fur Kieferorthopadie 62:22–35

Holzhausen M, Gonçalves D, Corrêa F de OB, et al (2003) A case of Zimmermann-Laband syndrome with supernumerary teeth. Journal of periodontology 74:1225–1230

Homma S, Koido S, Sagawa Y, et al (2009) Antigenic stimulation with cytochrome P450 2J expressed in mouse hepatocellular carcinoma cells regulates host anti-tumour immunity. Clinical and experimental immunology 156:344–352. <https://doi.org/10.1111/j.1365-2249.2009.03900.x>

Homma S., Komita H., Nagasaki E., et al (2007) Mechanism of antitumor effect on mouse hepatocellular carcinoma by intratumoral injection of OK-432, a streptococcal preparation. Cancer Immunology, Immunotherapy 56:1265–1274. <https://doi.org/10.1007/s00262-006-0277-9>

Honarmand M., Farad Mollashahi L., Shirzaiy M., Abbasi H. (2012) Epidemiology of dental fluorosis in 7-10 years old students attending to community dentistry center of Zahedan. Iranian Journal of Epidemiology 7:66–72

Hong L, Levy SM, Warren JJ, et al (2005) Association of amoxicillin use during early childhood with developmental tooth enamel defects. Archives of pediatrics & adolescent medicine 159:943–948

Hong L, Levy SM, Warren JJ, Broffitt B (2011) Amoxicillin use during early childhood and fluorosis of later developing tooth zones. Journal of public health dentistry 71:229–235

Hoods-Moonsammy VJ, Mothopi MM, Taruvingira AK, et al (2012) Prosthodontic management of patients with amelogenesis imperfecta. SADJ : journal of the South African Dental Association = tydskrif van die Suid-Afrikaanse Tandheelkundige Vereniging 67:409–412

Hoppenreijs TJ, Voorsmit RA, Freihofer HP, van ’t Hof MA (1998) Open bite deformity in amelogenesis imperfecta. Part 2: Le Fort I osteotomies and treatment results. Journal of cranio-maxillo-facial surgery : official publication of the European Association for Cranio-Maxillo-Facial Surgery 26:286–293

Hørberg M, Lauesen SR, Daugaard-Jensen J, Kjær I (2015) Linear scleroderma en coup de sabre including abnormal dental development. European archives of paediatric dentistry : official journal of the European Academy of Paediatric Dentistry 16:227–231. <https://doi.org/10.1007/s40368-014-0148-6>

Horowitz AM (1998) Response to Weinstein: public health issues in early childhood caries. Community dentistry and oral epidemiology 26:

Horowitz HS (1989) Fluoride and enamel defects. Advances in dental research 3:143–146

Horowitz H.S. (1986) Indexes for measuring dental fluorosis. Journal of public health dentistry 46:179–183

Horowitz RA, Gautam DK, Karol S, Kumari B (2014) Periodontal management and restoration of an amelogenesis imperfecta patient: a case report. Compendium of continuing education in dentistry (Jamesburg, NJ : 1995) 35:e6

Hosoya Y., Shiraishi T., Odatsu T., et al (2010) Effects of specular component and polishing on color of resin composites. Journal of oral science 52:599–607. <https://doi.org/10.2334/josnusd.52.599>

Houari S, Babajko S, Loiodice S, et al (2018) Micro-dissection of enamel organ from mandibular incisor of rats exposed to environmental toxicants. JoVE (Journal of Visualized Experiments) e57081

Houari S., Loiodice S., Jedeon K., et al (2016) Expression of steroid receptors in ameloblasts during amelogenesis in rat incisors. Frontiers in Physiology 7:. <https://doi.org/10.3389/fphys.2016.00503>

Houwink B, Wagg B (1979) Effect of fluoride dentifrice usage during infancy upon enamel mottling of the permanent teeth. Caries research 13:

Hovey L.R., Jones A.A., Cochran D.L., et al (2006) Application of periodontal tissue engineering using enamel matrix derivative and a human fibroblast-derived demal substitute to stimulate periodontal wound healing in class III furcation defects. Journal of Periodontology 77:790–799. <https://doi.org/10.1902/jop.2006.030264>

Howe LC, Palmer RM (1991) Periodontal and restorative treatment in a patient with familial gingival fibromatosis: a case report. Quintessence international (Berlin, Germany : 1985) 22:871–872

Hoyte T, Kowlessar A, Ali A, Bearn D (2020) Cross-Sectional survey to ascertain the prevalence of Molar Incisor Hypo-mineralization in the Trinidad and Tobago population. American Journal of Biomedical Science & Research 7:204–207

Hsiao S.Y., Fukao T., Koro M., et al (1989) A case of Prader-Willi syndrome. Shoni shikagaku zasshi The Japanese journal of pedodontics 27:700–707

Hsieh M.-F., Chen C.-H. (2012) Review: Delivery of pharmaceutical agents to treat acne vulgaris: Current status and perspectives. Journal of Medical and Biological Engineering 32:215–224. <https://doi.org/10.5405/jmbe.901>

Hsieh SG-S, Hibbert S, Shaw P, et al (2011) Association of cyclophosphamide use with dental developmental defects and salivary gland dysfunction in recipients of childhood antineoplastic therapy. Cancer 117:2219–2227. <https://doi.org/10.1002/cncr.25704>

Hu T., Ye G., Fan X.-P., et al (2018) Platelet-rich plasma combined with demineralized freeze-dried bone allografts for periodontal regeneration in the treatment of periodontal defects: a meta-analysis. Shanghai kou qiang yi xue = Shanghai journal of stomatology 27:546–553

Hua R., Li C., Gong P., et al (2012) Cerebrospinal fluid biochemistry reflects effects of therapeutic hypothermia after cardiac arrest in a porcine model. American Journal of Emergency Medicine 30:1420–1428. <https://doi.org/10.1016/j.ajem.2011.10.022>

Huang L-G, Chen G (2016) A histological and radiographic study of pulpal calcification in periodontally involved teeth in a Taiwanese population. Journal of dental sciences 11:405–410. <https://doi.org/10.1016/j.jds.2016.05.001>

Huang R.-Y., Tai W.-C., Chang P.-C., Ho M.-H. (2020) Combination of a biomolecule-aided biphasic cryogel scaffold with a barrier membrane adhering PDGF-encapsulated nanofibers to promote periodontal regeneration. Journal of periodontal research 55:529–538. <https://doi.org/10.1111/jre.12740>

Huang S., Li C., Yang X., et al (2019) Effect of inguinal hernia on the thickness and blood flow of spermatic cord in boys. World Journal of Pediatric Surgery 2:. <https://doi.org/10.1136/wjps-2018-000030>

Huang W-J, Lee H-J, Chen H-L, et al (2015) Hispidulin, a constituent of Clerodendrum inerme that remitted motor tics, alleviated methamphetamine-induced hyperlocomotion without motor impairment in mice. Journal of ethnopharmacology 166:18–22. <https://doi.org/10.1016/j.jep.2015.03.001>

Huang X, Xu X, Bringas P, et al (2010) Smad4-Shh-Nfic signaling cascade-mediated epithelial-mesenchymal interaction is crucial in regulating tooth root development. Journal of bone and mineral research : the official journal of the American Society for Bone and Mineral Research 25:1167–1178. <https://doi.org/10.1359/jbmr.091103>

Hubbard A, Guatelli-Steinberg D, Sciulli PW (2009) Under restrictive conditions, can the widths of linear enamel hypoplasias be used as relative indicators of stress episode duration? American journal of physical anthropology 138:177–189. <https://doi.org/10.1002/ajpa.20917>

Hubbard MJ (2018) Molar hypomineralization: What is the US experience? The Journal of the American Dental Association 149:329–330

Hubbard MJ, Mangum JE, Perez VA, et al (2017) Molar Hypomineralisation: A Call to Arms for Enamel Researchers. Frontiers in physiology 8:546. <https://doi.org/10.3389/fphys.2017.00546>

Huber M.A., Terezhalmy G.T. (2003) The head and neck radiation oncology patient. Quintessence International 34:693–717

Huebner S., Nelson M., Martinez B. (2012) Chronic urticaria as possible manifestation of autoimmune polyendocrinopathy-candidiasis-ectodermal dystrophy (APECED). Journal of Allergy and Clinical Immunology 129:. <https://doi.org/10.1016/j.jaci.2011.12.091>

Hug G., Mueller W.A., Steinle C.J. (1982) Teeth in pseudohypoparathyroidism: response to treatment with 1,25-dihydroxyvitamin D or vitamin D. Pediatric Research 16:

Hulsmann M (1997) Root canal treatment as a treatment modality for temporary tooth retention in adolescent patients. The Journal of clinical pediatric dentistry 21:109–115

Hunter L, Stone D (1997) Supraoccluding cobalt-chrome onlays in the management of amelogenesis imperfecta in children: a 2-year case report. Quintessence international (Berlin, Germany : 1985) 28:15–19

Husebye E.S., Perheentupa J., Rautemaa R., Kampe O. (2009) Clinical manifestations and management of patients with autoimmune polyendocrine syndrome type i. Journal of Internal Medicine 265:514–529. <https://doi.org/10.1111/j.1365-2796.2009.02090.x>

Hussein AS, Ghanim AM, Abu-Hassan MI, Manton DJ (2014) Knowledge, management and perceived barriers to treatment of molar-incisor hypomineralisation in general dental practitioners and dental nurses in Malaysia. European archives of paediatric dentistry : official journal of the European Academy of Paediatric Dentistry 15:301–307. <https://doi.org/10.1007/s40368-014-0115-2>

Hyder Z., Clayton-Smith J., Beale V., O’Connor R. (2017) Genitourinary malformations: An under-recognized feature of ectrodactyly, ectodermal dysplasia and cleft lip/palate syndrome. Clinical Dysmorphology 26:78–82. <https://doi.org/10.1097/MCD.0000000000000172>

Hypomineralization AMCMI Amoxicillin and Other Antibiotics May Be Reason for More Hypoplasia

Hypomineralization MI (2016) Molar incisor hypomineralization. The journal of contemporary dental practice 17:609–13

Hysi D, Kuscu OO, Droboniku E, et al (2016) Prevalence and aetiology of Molar-Incisor Hypomineralisation among children aged 8-10 years in Tirana, Albania. European journal of paediatric dentistry 17:75–79

Hystead E., Diez-Gonzalez F., Schoenfuss T.C. (2013) The effect of sodium reduction with and without potassium chloride on the survival of Listeria monocytogenes in Cheddar cheese. Journal of dairy science 96:6172–6185. <https://doi.org/10.3168/jds.2013-6675>

Hytonen M.K., Arumilli M., Hundi S., et al (2016) Molecular Characterization of Three Canine Models of Human Rare Bone Diseases: Caffey, van den Ende-Gupta, and Raine Syndromes. PLoS Genetics 12:. <https://doi.org/10.1371/journal.pgen.1006037>

Hytonen M.K., Arumilli M., Lohi H., et al (2019) Canine models of human amelogenesis imperfecta: identification of novel recessive ENAM and ACP4 variants. Human Genetics. <https://doi.org/10.1007/s00439-019-01997-8>

Ibarra-Santana C, Ruiz-Rodríguez M del S, Fonseca-Leal M del P, et al (2007) Enamel hypoplasia in children with renal disease in a fluoridated area. The Journal of clinical pediatric dentistry 31:274–278

Ibiyemi O, Zohoori FV, Valentine RA, et al (2018) Prevalence and extent of enamel defects in the permanent teeth of 8-year-old Nigerian children. Community dentistry and oral epidemiology 46:54–62. <https://doi.org/10.1111/cdoe.12328>

Ichijo T, Yamashita Y, Terashima T (1993) Observations on structural features and characteristics of biological apatite crystals. 8. Observation on fusion of human enamel crystals. The Bulletin of Tokyo Medical and Dental University 40:207–216

Ichijo T., Yamashita Y., Terashima T. (1993a) Observations on structural features and characteristics of biological apatite crystals. 6. Observation on lattice imperfection of human tooth and bone crystals. I. The Bulletin of Tokyo Medical and Dental University 40:147–165

Ichijo T., Yamashita Y., Terashima T. (1993b) Observations on structural features and characteristics of biological apatite crystals. 7. Observation on lattice imperfection of human tooth and bone crystals II. The Bulletin of Tokyo Medical and Dental University 40:193–205

Ide F, Mishima K, Kikuchi K, et al (2011) Development and growth of adenomatoid odontogenic tumor related to formation and eruption of teeth. Head and neck pathology 5:123–132. <https://doi.org/10.1007/s12105-011-0253-3>

Ide F, Shimoyama T, Horie N, Kaneko T (1999) Primary intraosseous carcinoma of the mandible with probable origin from reduced enamel epithelium. Journal of oral pathology & medicine : official publication of the International Association of Oral Pathologists and the American Academy of Oral Pathology 28:420–422

Ide M., Matsumoto E., Ohmori I. (1989) A histological observation of the primary teeth with enamel hypoplasia resulting from perinatal disturbance. Shoni shikagaku zasshi The Japanese journal of pedodontics 27:864–875

Ide T (2006) [A case of combined periodontal and prosthetic treatment for enamel hypocalcification]. Nihon Hotetsu Shika Gakkai zasshi 50:284–287

Idon PI, Enabulele JE (2018) Prevalence, severity, and request for treatment of dental fluorosis among adults in an endemic region of Northern Nigeria. European journal of dentistry 12:184–190. <https://doi.org/10.4103/ejd.ejd_260_17>

Igarashi R, Sahara T, Shimizu-Ishiura M, Sasaki T (2003) Porcine enamel matrix derivative enhances the formation of reparative dentine and dentine bridges during wound healing of amputated rat molars. Journal of electron microscopy 52:227–236

Ikeda S, Sekine A, Baba T, et al (2016) Administration of nintedanib after discontinuation for acute exacerbation of idiopathic pulmonary fibrosis: a case report. BMC pulmonary medicine 16:38. <https://doi.org/10.1186/s12890-016-0201-9>

Ikeue R., Sato T., Nakamura-Takahashi A., et al (2018) Bone-Targeted Alkaline Phosphatase Treatment of Mandibular Bone and Teeth in Lethal Hypophosphatasia via an scAAV8 Vector. Molecular Therapy - Methods and Clinical Development 10:361–370. <https://doi.org/10.1016/j.omtm.2018.08.004>

Ilgenstein I, Zitzmann N, Bühler J, et al (2015) Influence of proximal box elevation on the marginal quality and fracture behavior of root-filled molars restored with CAD/CAM ceramic or composite onlays. Clinical oral investigations 19:1021‐1028. <https://doi.org/10.1007/s00784-014-1325-z>

Ilian H, Tanya B, Dobromira S, et al (2017) Collaboration among dental technicians and dentists concerning the choice of relining material and method-a survey. Научни трудове на Съюза на учените–Пловдив Серия Г: Медицина, фармация и дентална медицина 21:

Incici E, Matuliene G, Hüsler J, et al (2009) Cumulative costs for the prosthetic reconstructions and maintenance in young adult patients with birth defects affecting the formation of teeth. Clinical oral implants research 20:715–721. <https://doi.org/10.1111/j.1600-0501.2009.01711.x>

Incisiva HM Molar incisor hypomineralization: Analysis of asymmetry of lesions

Inciso-Molar H Oral health-related quality of life in Colombian children with Molar-Incisor Hypomineralization

Innes N, Borrie F, Bearn D, et al (2013) Should I eXtract Every Six dental trial (SIXES): study protocol for a randomized controlled trial. Trials 14:59

Innes N, Ricketts D, Chong L, et al (2015) Preformed crowns for decayed primary molar teeth. Cochrane Database of Systematic Reviews. <https://doi.org/10.1002/14651858.CD005512.pub3>

Innes NP, Borrie FR, Bearn DR, et al SIXES Dental Trial; Should I extract Every Six? International Journal of Paediatric Dentistry 22:250–257

Intini G., Bobek L.A., Buhite R.J., Andreana S. (2008) A comparative analysis of bone formation induced by human demineralized freeze-dried bone and enamel matrix derivative in rat calvaria critical-size bone defects. Journal of Periodontology 79:1217–1224. <https://doi.org/10.1902/jop.2008.070435>

Ioannou S., Henneberg M. (2016) Dental characteristics of clinically diagnosed cases of congenital syphilis in the United States of America prior to 1910. American Journal of Physical Anthropology 159:181–182. <https://doi.org/10.1002/ajpa.22955>

Iório PA (1974) [Restoration of hypoplastic and pigmented teeth using adhesive compounds]. Revista brasileira de odontologia 31:236–239

Iorio P.A. (1974) Recomposicao de dentes hipoplasicos e pigmentados com o emprego dos compostos adesivosRestoration of hypoplastic and pigmented teeth using adhesive compounds. Revista brasileira de odontologia 31:236–239

Iorio-Siciliano V, Andreuccetti G, Blasi A, et al (2014) Clinical outcomes following regenerative therapy of non-contained intrabony defects using a deproteinized bovine bone mineral combined with either enamel matrix derivative or collagen membrane. Journal of periodontology 85:1342‐1350. <https://doi.org/10.1902/jop.2014.130420>

Iorio-Siciliano V, Blasi A, Nuzzolo P, et al Treatment of Periodontal Intrabony Defects Using Enamel Matrix Derivative: Surgical Reentry After an Observation Period of at Least 5 Years. The International journal of periodontics & restorative dentistry 39:537–543. <https://doi.org/10.11607/prd.4148>

Iqbal MK (2007) Clinical and scanning electron microscopic features of invasive cervical resorption in a maxillary molar. Oral surgery, oral medicine, oral pathology, oral radiology, and endodontics 103:e49

IRCT138706241191N2 (2008) vital pulp therapy in primary teeth. http://www.who.int/trialsearch/Trial2.aspx?TrialID=IRCT138706241191N2

IRCT138811082016N3 (2010) Comparison the effect of pretreatment with lbuprofen, Indomethacin, and placebo on postoperative pain of mandibular molars with irreversible pulpitis. http://www.who.int/trialsearch/Trial2.aspx?TrialID=IRCT138811082016N3

IRCT138902203893N2 (2016) Comparative evaluation of the success rate of applying formocresol, 3Mixtatin and Mineral trioxide aggregatein pulpotomy of primary molars. http://www.who.int/trialsearch/Trial2.aspx?TrialID=IRCT138902203893N2

IRCT201102195861N1 (2011) Clinical and radiographic evaluation of bone powder with membrane and bone powder with connective tissue in the treatment of bone loss of bifurcation molars. http://www.who.int/trialsearch/Trial2.aspx?TrialID=IRCT201102195861N1

IRCT201103256105N1 (2011) Effect of Meridol and Kids Irsha mouth rinses on the amount of plaque accumulation in children. http://www.who.int/trialsearch/Trial2.aspx?TrialID=IRCT201103256105N1

IRCT201110221861N3 (2012) Fluoride Varnish versus Oral Hygiene Instructions in Control of Incipient carious Lesions. http://www.who.int/trialsearch/Trial2.aspx?TrialID=IRCT201110221861N3

IRCT201111288242N1 (2012) Evaluation of ethanol wet bonding. http://www.who.int/trialsearch/Trial2.aspx?TrialID=IRCT201111288242N1

IRCT201201231760N15 (2012) The effect of plasma rich growth factor in treatment of molar class II furcation involvement. http://www.who.int/trialsearch/Trial2.aspx?TrialID=IRCT201201231760N15

IRCT201202209085N1 (2012) Effect of laser on prevention of caries on teeth. http://www.who.int/trialsearch/Trial2.aspx?TrialID=IRCT201202209085N1

IRCT201601202016N6 (2016) Effect of concentration of irrigants on endodontic postoperative pain. http://www.who.int/trialsearch/Trial2.aspx?TrialID=IRCT201601202016N6

IRCT2016011911888N2 (2016) The effect of propolis and nanohydroxyapatite in treatment of dentinhypersensivity. http://www.who.int/trialsearch/Trial2.aspx?TrialID=IRCT2016011911888N2

IRCT20171013036745N2 (2018) The effect of Intraosseous injection in reducing pain and anxiety in children with hypomineralized Mandibular Molar. http://www.who.int/trialsearch/Trial2.aspx?TrialID=IRCT20171013036745N2

Iriei M., Homma S., Komita H., et al (2004) Inhibition of spontaneous development of liver tumors by inoculation with dendritic cells loaded with hepatocellular carcinoma cells in C3H/HeNCRJ mice. International Journal of Cancer 111:238–245. <https://doi.org/10.1002/ijc.20247>

Irigoyen-Camacho ME, Villanueva-Gutierrez T, Castano-Seiquer A, et al (2020) Evaluating the changes in molar incisor hypomineralization prevalence: A comparison of two cross-sectional studies in two elementary schools in Mexico City between 2008 and 2017. Clinical and experimental dental research 6:82–89. <https://doi.org/10.1002/cre2.252>

Isben RL (1972) Conservative restorations of decalcified enamel with an adhesive composite. Case report. Journal - Southern California Dental Association 40:926–927

Isehed C, Holmlund A, Renvert S, et al (2016) Effectiveness of enamel matrix derivative on the clinical and microbiological outcomes following surgical regenerative treatment of peri-implantitis. A randomized controlled trial. Journal of clinical periodontology 43:863‐873. <https://doi.org/10.1111/jcpe.12583>

Ishikawa M, Satoh K, Miyashin M (1990) [A clinical study of traumatic injuries to deciduous teeth. (3). The influence on their permanent successors]. Shoni shikagaku zasshi The Japanese journal of pedodontics 28:397–406

Ismail AI (1998) The role of early dietary habits in dental caries development. Special care in dentistry : official publication of the American Association of Hospital Dentists, the Academy of Dentistry for the Handicapped, and the American Society for Geriatric Dentistry 18:40–45

Ismail J.Y., Zaki H.S. (1990) Osseointegration in maxillofacial prosthetics. Dental clinics of North America 34:327–341

Ismail OS, Weber DF (1988) Light and scanning electron microscopic observations of the canalicular system in human cellular cementum. The Anatomical record 222:121–127

ISRCTN14189134 (2019) Treatment of periodontitis using a surgical regenerative therapy. http://www.who.int/trialsearch/Trial2.aspx?TrialID=ISRCTN14189134

ISRCTN18931678 (2008) Clinical trial for the treatment of pulmonary alveolar proteinosis by inhalation of recombinant human granulocyte-macrophage colony stimulating factor (GMCSF). http://www.who.int/trialsearch/Trial2.aspx?TrialID=ISRCTN18931678

Ito N., Suzuki E., Kawakami C., Inoue F. (2013) The state of dental visit and oral health care in childhood cancer survivors. Pediatric Blood and Cancer 60:184. <https://doi.org/10.1002/pbc.24719>

Ivanovic D., Malcic Ivanisevic A., Brzovic Rajic V. (2019) Mikroinvazivna terapija hipomineralizirane cakline primjenom infiltracijske smoleMicroinvasive therapy of hipomineralized enamel by application of infiltration resin. Acta Stomatologica Croatica 53:282

Ivanović M, Živojinović V, Marković D, Šindolić M (2006) Mogućnosti terapije hipomineralizovanih prvih stalnih molara i inciziva. Serbian Dental Journal/Stomatološki Glasnik Srbije 53:

Ivanovic M., Zivojinovic V., Sindolic M., Markovic D. (2007) Molar incisor hypomineralisation in the first permanent teeth. Srpski arhiv za celokupno lekarstvo 135:472–477

Ivanović M, Živojinović V, Šindolić M, Marković D (2007a) Hipomineralizacija na prvim stalnim molarima i incizivima

Ivanović M, Živojinović V, Šindolić M, Marković D (2007b) Molar incisor hypomineralisation. Srpski arhiv za celokupno lekarstvo 135:472–477

Ives R (2014) An unusual double supernumerary maxillary mesiodens in a Middle Iron Age skeleton from South Uist, Western Isles, Scotland. Archives of oral biology 59:625–630. <https://doi.org/10.1016/j.archoralbio.2014.03.009>

Iwamoto M., Shapiro I.M., Yagami K., et al (1993) Retinoic acid induces rapid mineralization and expression of mineralization-related genes in chondrocytes. Experimental Cell Research 207:413–420. <https://doi.org/10.1006/excr.1993.1209>

Izaguirre MCH, Perleche DMA (2019) Factores genéticos asociados a la hipomineralización incisivo-molar. Revisión de literatura. Revista Científica Odontológica 7:148–156

Jabbari F., Reiser E., Skoog V., et al (2015) Optimization of dental status improves long-term outcome after alveolar bone grafting in unilateral cleft lip and palate. Cleft Palate-Craniofacial Journal 52:210–218. <https://doi.org/10.1597/13-118>

Jaber L, Mascrès C, Donohue WB (1991) Electron microscope characteristics of dentin repair after hydroxylapatite direct pulp capping in rats. Journal of oral pathology & medicine : official publication of the International Association of Oral Pathologists and the American Academy of Oral Pathology 20:502–508

Jaber L, Mascrès C, Donohue WB (1992) Reaction of the dental pulp to hydroxyapatite. Oral surgery, oral medicine, and oral pathology 73:92–98

Jacob J, Bartholmai BJ, Egashira R, et al (2017a) Chronic hypersensitivity pneumonitis: identification of key prognostic determinants using automated CT analysis. BMC pulmonary medicine 17:81. <https://doi.org/10.1186/s12890-017-0418-2>

Jacob J, Bartholmai BJ, Rajagopalan S, et al (2017b) Mortality prediction in idiopathic pulmonary fibrosis: evaluation of computer-based CT analysis with conventional severity measures. The European respiratory journal 49:. <https://doi.org/10.1183/13993003.01011-2016>

Jafari A, Mohebbi S, Khami M, et al (2012) Radiographic evaluation of third molar development in 5- to 25 year olds in tehran, iran. Journal of dentistry (Tehran, Iran) 9:107–115

Jain MC, Dixit UmB (2018) Misdiagnosis: How Uncommonly Common is it? Journal of Clinical & Diagnostic Research 12:

Jain P, Patni P, Hiremath H, Jain N (2014) Successful removal of a 16 mm long pulp stone using ultrasonic tips from maxillary left first molar and its endodontic management. Journal of conservative dentistry : JCD 17:92–95. <https://doi.org/10.4103/0972-0707.124170>

Jairam LS, Dhananjaya G (2019) Molar Incisor Hypominearlisation: An Overview. Journal of Dental and Orofacial Research 15:89–94

Jaiswal R, Deo V (2013) Evaluation of the effectiveness of enamel matrix derivative, bone grafts, and membrane in the treatment of mandibular Class II furcation defects. International journal of periodontics & restorative dentistry 33:e58‐64. <https://doi.org/10.11607/prd.1428>

Jalali R, Guy F, Ghazanfari S, et al (2017) Mineralization-defects are comparable in fluorotic impacted human teeth and fluorotic mouse incisors. Archives of oral biology 83:214–221. <https://doi.org/10.1016/j.archoralbio.2017.07.018>

Jälevik B (2010) Prevalence and diagnosis of molar-incisor-hypomineralisation (MIH): a systematic review. European Archives of Paediatric Dentistry 11:59–64

Jalevik B. (2001) Enamel hypomineralization in permanent first molars. A clinical, histo-morphological and biochemical study. Swedish dental journal Supplement 1–86

Jälevik B, Dietz W, Norén JG (2005) Scanning electron micrograph analysis of hypomineralized enamel in permanent first molars. International journal of paediatric dentistry 15:233–240

Jalevik B., Fasth A., Dahllof G. (2002) Dental development after successful treatment of infantile osteopetrosis with bone marrow transplantation. Bone Marrow Transplantation 29:537–540. <https://doi.org/10.1038/sj/bmt/1703416>

Jalevik B., Klingberg G., Barregard L., Noren J.G. (2001) The prevalence of demarcated opacities in permanent first molars in a group of Swedish children. Acta odontologica Scandinavica 59:255–260

Jälevik B, Klingberg GA (2002) Dental treatment, dental fear and behaviour management problems in children with severe enamel hypomineralization of their permanent first molars. International journal of paediatric dentistry 12:24–32

Jälevik B, Norén JG (2000) Enamel hypomineralization of permanent first molars: a morphological study and survey of possible aetiological factors. International journal of paediatric dentistry 10:278–289

Jälevik B, Norén JG, Klingberg G, Barregård L (2001) Etiologic factors influencing the prevalence of demarcated opacities in permanent first molars in a group of Swedish children. European journal of oral sciences 109:230–234

Jälevik B, Szigyarto-Matei A, Robertson A (2018) The prevalence of developmental defects of enamel, a prospective cohort study of adolescents in Western Sweden: a Barn I TAnadvarden (BITA, children in dental care) study. European Archives of Paediatric Dentistry 19:187–195

Jälevik B, Szigyarto-Matei A, Robertson A (2019) Difficulties in identifying developmental defects of the enamel: a BITA study. European Archives of Paediatric Dentistry 20:481–488

Jalili IK (2010) Cone-rod dystrophy and amelogenesis imperfecta (Jalili syndrome): phenotypes and environs. Eye (London, England) 24:1659–1668. <https://doi.org/10.1038/eye.2010.103>

James P, Harding M, Beecher T, et al (2018) Fluoride And Caring for Children’s Teeth (FACCT): Clinical Fieldwork Protocol. HRB Open Research 1:

Jańczuk Z, Opalko K, Domzalska E, Lisiecka K (1984) [Enamel mineralization disorders in children undergoing fluoride prevention for caries. Clinical studies]. Czasopismo stomatologiczne 37:639–645

Janczuk Z., Opalko K., Domzalska E., Lisiecka K. (1984) Zaburzenia mineralizacji szkliwa u dzieci poddanych profilaktyce fluorkowej prochnicy. Badanie klinizneEnamel mineralization disorders in children undergoing fluoride prevention for caries. Clinical studies. Czasopismo stomatologiczne 37:639–645

Jankovic S., Davidovic B., Lecic J., Ivanovic M. (2014) Rasprostranjenost i karakteristike hipomineralizacije na kutnjacima i sekuticimaDistribution and characteristics of molar-incisor hypomineralization. Vojnosanitetski Pregled 71:730–734. <https://doi.org/10.2298/VSP1408730J>

Janković S, Ivanović M, Davidović B, Lečić J (2013) Aetiological factors of molar incisor hypomineralization. Stomatološki glasnik Srbije 60:69–75

Janković S, Ivanović M, Davidović B, Lecić J (2014) Distribution and characteristics of molar-incisor hypomineralization. Vojnosanitetski pregled 71:730–734

Jankowska K., Kaczmarek U. (2012) Stan uzebienia, przyzebia i higieny jamy ustnej u chorych na mozgowe porazenie dziecieceDental status, periodontal condition and oral hygiene of patients suffering from cerebral palsy. Dental and Medical Problems 49:495–501

Jans Muñoz A, Díaz Meléndez J, Vergara González C, Zaror Sánchez C (2011) Frecuencia y severidad de la hipomineralización molar incisal en pacientes atendidos en las clínicas odontológicas de la Universidad de La Frontera, Chile. International journal of odontostomatology 5:133–140

Jasulaityte L, Veerkamp JS, Weerheijm KL (2007) Molar incisor hypomineralization: review and prevalence data from the study of primary school children in Kaunas/Lithuania. European archives of paediatric dentistry : official journal of the European Academy of Paediatric Dentistry 8:87–94

Jasulaityte L, Weerheijm KL, Veerkamp JS (2008) Prevalence of molar-incisor-hypomineralisation among children participating in the Dutch National Epidemiological Survey (2003). European archives of paediatric dentistry : official journal of the European Academy of Paediatric Dentistry 9:218–223

Jayam C, Bandlapalli A, Patel N, Choudhary RSK (2014) Chronological hypoplasia: aesthetic management. BMJ case reports 2014:. <https://doi.org/10.1136/bcr-2013-202892>

Jayam C, Choudhary P, Venkataraghavan K, et al (2013) Linear Enamel Hypoplasia: Case Report. Journal of Advanced Oral Research 4:12–15

Jedeon K., Berdal A., Babajko A. (2016a) Impact of three endocrine disruptors, Bisphenol A, Genistein and Vinclozolin on female rat enamel. Bulletin du Groupement international pour la recherche scientifique en stomatologie & odontologie 53:

Jedeon K., Berdal A., Babajko S., et al (2014) Enamel hypomineralization due to endocrine disruptors. Connective Tissue Research 55:43–47. <https://doi.org/10.3109/03008207.2014.923857>

Jedeon K, Berdal A, Babajko S (2015) The tooth, target organ of Bisphenol A, could be used as a biomarker of exposure to this agent. Sources, Risks of Environmental Exposure and Human Health Effects, eds YG, and A Bisphenol (New York, NY: Nova Science Publishers) 205–225

Jedeon K, De la Dure-Molla M, Brookes SJ, et al (2013) Enamel defects reflect perinatal exposure to bisphenol A. The American journal of pathology 183:108–118

Jedeon K, Houari S, Loiodice S, et al (2016a) Chronic Exposure to Bisphenol A Exacerbates Dental Fluorosis in Growing Rats. Journal of bone and mineral research : the official journal of the American Society for Bone and Mineral Research 31:1955–1966. <https://doi.org/10.1002/jbmr.2879>

Jedeon K, Loiodice S, Houari S, et al (2016b) Systemic enamel pathologies may be due to anti-androgenic effects of some endocrine disruptors. BioScientifica

Jedeon K, Loiodice S, Marciano C, et al (2014a) Estrogen and bisphenol A affect male rat enamel formation and promote ameloblast proliferation. Endocrinology 155:3365–3375

Jedeon K., Loiodice S., Salhi K., et al (2016b) Androgen receptor involvement in rat amelogenesis: An additional way for endocrine-disrupting chemicals to affect enamel synthesis. Endocrinology 157:4287–4296. <https://doi.org/10.1210/en.2016-1342>

Jedeon K, Loiodice S, Salhi K, et al (2016c) Androgen receptor involvement in rat amelogenesis: an additional way for endocrine-disrupting chemicals to affect enamel synthesis. Endocrinology 157:4287–4296

Jedeon K, Marciano C, Loiodice S, et al (2014b) Enamel hypomineralization due to endocrine disruptors. Connective tissue research 55:43–47

Jekl V., Hauptman K., Knotek Z. (2017) Evidence-Based Advances in Rodent Medicine. Veterinary Clinics of North America - Exotic Animal Practice 20:805–816. <https://doi.org/10.1016/j.cvex.2017.04.012>

Jelinek E (1975) [Restoration of anterior teeth with Palakav]. Acta stomatologica Croatica 9:56–60

Jelinek E. (1975) Palakav u rekonstrukciji traumom ostecenih zubiRestoration of anterior teeth with Palakav. Acta stomatologica Croatica 9:56–60

Jenkins JF, St Germain HA (2019) Unintentional removal of a developing permanent premolar during primary molar extraction: a 10-year case report. General dentistry 67:50–53

Jenny L.L. (2012) The spatial distribution of skeletal stress indicators in a 4th century Romano-British sample: A study using ArcGIS. American Journal of Physical Anthropology 147:174. <https://doi.org/10.1002/ajpa.22033>

Jentsch H, Purschwitz R (2008) A clinical study evaluating the treatment of supra-alveolar-type defects with access flap surgery with and without an enamel matrix protein derivative: a pilot study. Journal of clinical periodontology 35:713‐718. <https://doi.org/10.1111/j.1600-051X.2008.01253.x>

Jepsen S, Heinz B, Jepsen K, et al (2004) A randomized clinical trial comparing enamel matrix derivative and membrane treatment of buccal Class II furcation involvement in mandibular molars. Part I: study design and results for primary outcomes. Journal of periodontology 75:1150‐1160. <https://doi.org/10.1902/jop.2004.75.8.1150>

Jepsen S, Topoll H, Rengers H, et al (2008) Clinical outcomes after treatment of intra-bony defects with an EMD/synthetic bone graft or EMD alone: a multicentre randomized-controlled clinical trial. Journal of clinical periodontology 35:420‐428. <https://doi.org/10.1111/j.1600-051X.2008.01217.x>

Jeremias F (2010) Hipomineralização Molar-Incisivo: prevalência, severidade e etiologia em escolares de Araraquara

Jeremias F (2013) Avaliação genética da hipomineralização molar-incisivo

Jeremias F, da Costa Silva CM, de Souza JF, et al (2010) Hipomineralización de incisivos y molares: aspectos clínicos de La severidad. Acta odontológica venezolana 48:23–24

Jeremias F, Koruyucu M, Küchler EC, et al (2013) Genes expressed in dental enamel development are associated with molar-incisor hypomineralization. Archives of oral biology 58:1434–1442

Jeremiasa JF de SF, Ângela CM da CS, dos Santos CCZL, Cordeiroc PR de CL (2011) HIPOMINERALIZACIÓN INCISIVO Y MOLAR: DIAGNÓSTICO DIFERENCIAL. Acta Odontológica Venezolana 49:

Jewell R, Sarkar A, Jones R, et al (2017) Atypical osteogenesis imperfecta caused by a 17q21. 33 deletion involving COL1A1. Clinical Dysmorphology

Jiang S, Liu T, Wu G, et al (2020) BMP2-Functionalized Biomimetic Calcium Phosphate Graft Promotes Alveolar Defect Healing During Orthodontic Tooth Movement in Beagle Dogs. Frontiers in bioengineering and biotechnology 8:517. <https://doi.org/10.3389/fbioe.2020.00517>

Jihene Z, Imene J, Badiaa J (2018) Extraction of Decayed and Dilapidated First Permanent Molars in Mixed Dentition and Spontaneous Space Closure: A Case Report

Jogendra S.S.A., Bandi S., Akkla S.G., Chinta M. (2015) Oral rehabilitation of a child with goldenhar syndrome. Journal of Young Pharmacists 7:276–280. <https://doi.org/10.5530/jyp.2015.3.21>

Johansen M (2015) Hemostasis and endothelial damage during sepsis. Danish medical journal 62:

Johar U., Jain D., Edge C.J. (2014) Neonatal teeth associated with epidermolysis bullosa: A case report. British Journal of Oral and Maxillofacial Surgery 52:. <https://doi.org/10.1016/j.bjoms.2014.07.221>

Johnsen DC (1984) Dental caries patterns in preschool children. Dental clinics of North America 28:3–20

Johnsen D.C., Dixon M. (1984) Dental caries of primary incisors in children with cleft lip and palate. Cleft Palate Journal 21:104–109

Johnsen DC, Schultz DW, Schubot DB, Easley MW (1984) Caries patterns in Head Start children in a fluoridated community. Journal of public health dentistry 44:61–66

Johnson CG, Sayegh FS, O’Toole TJ (1978) Acid-etch repair of hereditary type 4 enamel hypoplasia. Journal of the American Dental Association (1939) 97:223–226

Johnson CM, Makai GEH (2019) A Systematic Review of Perioperative Opioid Management for Minimally Invasive Hysterectomy. Journal of minimally invasive gynecology 26:233–243. <https://doi.org/10.1016/j.jmig.2018.08.024>

Johnson L, Ganss B, Wang A, et al (2017) V-ATPases Containing a3 Subunit Play a Direct Role in Enamel Development in Mice. Journal of cellular biochemistry 118:3328–3340. <https://doi.org/10.1002/jcb.25986>

Johnston WM (2014) Review of translucency determinations and applications to dental materials. Journal of esthetic and restorative dentistry : official publication of the American Academy of Esthetic Dentistry . [et al] 26:217–223. <https://doi.org/10.1111/jerd.12112>

Joho JP, Marechaux SC (1980) Amelogenesis imperfecta: treatment of case. ASDC journal of dentistry for children 47:266–268

Jongsma A.C. (2011) Proefschriften 25 jaar na dato 17. Molaarcrypten in de onderkaak[Permanent molar crypts in the human mandible]. Nederlands tijdschrift voor tandheelkunde 118:387–391

Jordan RA, Bodechtel C, Hertrampf K, et al (2014) The Fifth German Oral Health Study (Fünfte Deutsche Mundgesundheitsstudie, DMS V)–rationale, design, and methods. BMC oral health 14:161

Jordan RE, Suzuki M, Gwinnett AJ, Hunter JK (1977) Restoration of fractured and hypoplastic incisors by the acid etch resin technique: a three-year report. Journal of the American Dental Association (1939) 95:795–803

Jorgenson R.J., Levin L.S., McKusick V.A. (1974) Heritable oral handicaps. Dental Clinics of North America 18:579–594

Joseph M (2008) Endodontic treatment in three taurodontic teeth associated with 48,XXXY Klinefelter syndrome: a review and case report. Oral surgery, oral medicine, oral pathology, oral radiology, and endodontics 105:670–677. <https://doi.org/10.1016/j.tripleo.2007.11.015>

Joshi N, Hamdan AM, Fakhouri WD (2014) Skeletal malocclusion: a developmental disorder with a life-long morbidity. Journal of clinical medicine research 6:399–408. <https://doi.org/10.14740/jocmr1905w>

Jośko-Ochojska J, Rygiel K, Postek-Stefańska L (2019) Diseases of the oral cavity in light of the newest epigenetic research: Possible implications for stomatology. Advances in clinical and experimental medicine : official organ Wroclaw Medical University 28:397–406. <https://doi.org/10.17219/acem/76060>

JPRN-UMIN000012590 (2014) The retrospective study of treatment by thrombomodulin for acute exacerbations of idiopathic pulmonary fibrosis, acute respiratory distress sydmrome. http://www.who.int/trialsearch/Trial2.aspx?TrialID=JPRN-UMIN000012590

JPRN-UMIN000019684 (2015) Study of Clinical evaluation of beauty sealant. http://www.who.int/trialsearch/Trial2.aspx?TrialID=JPRN-UMIN000019684

JPRN-UMIN000021109 (2016) Preventive effect of Sivelestat Na Hydrate on postoperative acute exacerbation of idiopathic interstitial pneumonia in the patient with lung cancer. http://www.who.int/trialsearch/Trial2.aspx?TrialID=JPRN-UMIN000021109

JPRN-UMIN000027743 (2017) Effect of Autogenous Cortical Bone Particulate in Conjunction With Fibroblast Growth Factor in the Treatment of Periodontal Intraosseous Defects. http://www.who.int/trialsearch/Trial2.aspx?TrialID=JPRN-UMIN000027743

JR B (2016) Do We Really Know the Prevalence of MIH? Journal of Clinical Pediatric Dentistry 40:

Jurlina D., Kopic V., Catovic I., et al (2019) Parodontolosko regenerativno kirursko zbrinjavanje dvozidnog kostanog defekta - prikaz slucajaPeriodontal regenerative surgical care of twowalled periodontal bony defect-case report. Acta Stomatologica Croatica 53:187–188

Jurlina D, Uzarevic Z, Ivanisevic Z, et al (2020) Prevalence of Molar–Incisor Hypomineralization and Caries in Eight-Year-Old Children in Croatia. International Journal of Environmental Research and Public Health 17:6358

Kabakcieva R, Apostolova V (1989) [Study on the interrelation between dysplasias of permanent teeth and dental-jaw deformities. 1. Clinical-statistical study on the incidence and type of dental-jaw deformities among students with dysplasia of the permanent teeth]. Stomatologiia Stomatology 71:5–11

Kabakcieva R., Apostolova V. (1989) Izsledvane na vzanmovruzkata mezhdu displaziite na postoiannite zubi i zubno-cheliustnite deformatsii. 1. Kliniko-statistichesko prouchvane vurkhu chestotata i vida na subno-cheliustnite deformatsii pri uchenitsi s displazii na postoiannite zubiStudy on the interrelation between dysplasias of permanent teeth and dental-jaw deformities. 1. Clinical-statistical study on the incidence and type of dental-jaw deformities among students with dysplasia of the permanent teeth. Stomatologiia Stomatology 71:5–11

Kabakcieva R, Mihajlova E (1989) [Some clinical-laboratory indices in children with dental dysplasia of permanent teeth]. Stomatologiia Stomatology 71:10–13

Kabakcieva R., Mihajlova E. (1989) Niakoi klnikolaboratorni pokazateli pri detsa sus zubna displaziia na postoiannite zubiSome clinical-laboratory indices in children with dental dysplasia of permanent teeth. Stomatologiia Stomatology 71:10–13

Kaczmarek U., Jaworski A. (2014) Hipomineralizacja trzonowcowo-siekaczowa - Etiologia, czestosc wystepowania, obraz kliniczny i leczenie - Przeglad pismiennictwaMolar-incisor hypomineralisation - Etiology, prevalence, clinical picture and treatment - Review. Dental and Medical Problems 51:165–171

Kaczmarek U, Wrzyszcz-Kowalczyk A, Jankowska K, et al (2020) Oral health conditions in children with idiopathic nephrotic syndrome: a cross-sectional study. BMC oral health 20:213. <https://doi.org/10.1186/s12903-020-01197-1>

Kaczor M.P., Pawlik D., Wojcik R., et al (2012) Cost of chronic hepatitis C (HCV) in Poland-health care professionals survey. Value in Health 15:. <https://doi.org/10.1016/j.jval.2012.08.778>

Kadanakuppe S., Bhat P.K. (2013) Oral health status and treatment needs of Iruligas at Ramanagara District, Karnataka, India. The West Indian medical journal 62:73–80

Kagerer P, Grupe G (2001) Age-at-death diagnosis and determination of life-history parameters by incremental lines in human dental cementum as an identification aid. Forensic science international 118:75–82

Kahle P., Ludolphy C., Kierdorf H., Kierdorf U. (2018) Dental anomalies and lesions in Eastern Atlantic harbor seals, Phoca vitulina vitulina (Carnivora, Phocidae), from the German North Sea. PLoS ONE 13:. <https://doi.org/10.1371/journal.pone.0204079>

Kaipatur N.R., Murshed M., McKee M.D. (2008) Matrix Gla protein inhibition of tooth mineralization. Journal of dental research 87:839–844. <https://doi.org/10.1177/154405910808700907>

Kakaounaki E, Tahmassebi J, Fayle S (2006) Further dental treatment needs of children receiving exodontia under general anaesthesia at a teaching hospital in the UK. International Journal of Paediatric Dentistry 16:263–269

Kalkani M, Balmer RC, Homer RM, et al (2016) Molar incisor hypomineralisation: experience and perceived challenges among dentists specialising in paediatric dentistry and a group of general dental practitioners in the UK. European archives of paediatric dentistry : official journal of the European Academy of Paediatric Dentistry 17:81–88. <https://doi.org/10.1007/s40368-015-0209-5>

Kalra A, Mohan MS, Gowda EM (2015) Comparison of shear bond strength of two porcelain repair systems after different surface treatment. Contemporary clinical dentistry 6:196–200. <https://doi.org/10.4103/0976-237X.156045>

Kamata T., Kamezawa H., Yokozuka S. (1989) Stress analysis at the metal-enamel junction of the anterior adhesive bridge on non-prepared teeth with three dimensional photoelastic experiment. Shigaku Odontology; journal of Nippon Dental College 77:516–530

Kamble VD, Parkhedkar RD (2013) Multidisciplinary approach for restoring function and esthetics in a patient with amelogenesis imperfecta: a clinical report. Journal of clinical and diagnostic research : JCDR 7:3083–3085. <https://doi.org/10.7860/JCDR/2013/6665.3860>

Kameli S, Moradi-Kor N, Tafaroji R, et al (2019) Effects of Amoxicillin on the Structure and Mineralization of Dental Enamel and Dentin in Wistar Rats. Frontiers in Dentistry 16:130

KAMEN S, LAPOOK S (1965) RESTORATIVE TREATMENT FOR A MENTAL RETARDATE WITH ATYPICAL AMELOGENESIS IMPERFECTA. Journal of dentistry for children (Chicago, Ill) 32:189–193

Kamishima N, Ikeda T, Sano H (2005) Color and translucency of resin composites for layering techniques. Dental materials journal 24:428–432

Kamiya H, Panlaqui OM, Izumi S, Sozu T (2018) Systematic review and meta-analysis of prognostic factors for idiopathic inflammatory myopathy-associated interstitial lung disease. BMJ open 8:e023998. <https://doi.org/10.1136/bmjopen-2018-023998>

Kamp AA, Johnson BE (1987) Dental management of hypoplastic Amelogenesis imperfecta with a simplified acid etch composite resin technique. Journal (Indiana Dental Association) 66:11–13

Kanagaratnam S, Schluter P, Durward C, et al (2009) Enamel defects and dental caries in 9-year-old children living in fluoridated and nonfluoridated areas of Auckland, New Zealand. Community dentistry and oral epidemiology 37:250–259. <https://doi.org/10.1111/j.1600-0528.2009.00465.x>

Kanchan T, Machado M, Rao A, et al (2015) Enamel hypoplasia and its role in identification of individuals: A review of literature. Indian journal of dentistry 6:99–102. <https://doi.org/10.4103/0975-962X.155887>

Kandalgaonkar SD, Gharat LA, Tupsakhare SD, Gabhane MH (2013) Invasive cervical resorption: a review. Journal of international oral health : JIOH 5:124–130

Kandan P.M., Menaga V., Kumar R.R.R. (2011) Oral health in pregnancy (Guidelines to gynaecologists, general physicians & oral health care providers). Journal of the Pakistan Medical Association 61:1009–1014

Kang B.C., Farman A.G., Scarfe W.C., Goldsmith L.J. (1996) Observer differentiation of proximal enamel mechanical defects versus natural proximal dental caries with computed dental radiography. Oral surgery, oral medicine, oral pathology, oral radiology, and endodontics 82:459–465

Kanji J., Chopra S., Anderson J., ODonnell M. (2009) Dramatic onset of acquired red cell spherocytosis in a febrile diabetic patient: Clues from the blood film. International Journal of Antimicrobial Agents 34:

Kanthathas K., Willmot D.R., Benson P.E. (2005) Differentiation of developmental and post-orthodontic white lesions using image analysis. European Journal of Orthodontics 27:167–172. <https://doi.org/10.1093/ejo/cjh084>

Kao RT, Nares S, Reynolds MA (2015) Periodontal regeneration - intrabony defects: a systematic review from the AAP Regeneration Workshop. Journal of periodontology 86:. <https://doi.org/10.1902/jop.2015.130685>

Kaplan B, Marx SG (1967) Restoration of the maxillary canine teeth of a dog with enamel and dentin hypoplasia. Journal of the American Veterinary Medical Association 150:603–607

Kaplova E., Krejci P., Kramerova L., et al (2013) Vyvojove poruchy zubu a jejich diagnostika pomoci rentgenovych snimku. Lekar a Technika 43:23–27

Karadag R, Rapuano CJ, Hammersmith KM, Nagra PK (2020) Causes of congenital corneal opacities and their management in a tertiary care center. Arquivos brasileiros de oftalmologia 83:98–102. <https://doi.org/10.5935/0004-2749.20200023>

Karadayi B, Kaya A, Kolusayın MO, et al (2012) Radiological age estimation: based on third molar mineralization and eruption in Turkish children and young adults. International journal of legal medicine 126:933–942. <https://doi.org/10.1007/s00414-012-0773-8>

Karapanou V (2005) Endodontic management of an impacted premolar. The Journal of clinical pediatric dentistry 29:293–298

Karapataki S., Hugoson A., Kugelberg C.F. (2000) Healing following GTR treatment of bone defects distal to mandibular 2nd molars after surgical removal of impacted 3rd molars. Journal of clinical periodontology 27:325–332

Karataş MS, Sönmez IŞ (2013) Developmental disturbances of a maxillary central incisor due to trauma to its predecessor: a case report. Medical principles and practice : international journal of the Kuwait University, Health Science Centre 22:590–592. <https://doi.org/10.1159/000350500>

Karlsson V., Dankiewicz J., Nielsen N., et al (2015) Association of gender to outcome after out-of-hospital cardiac arrest - a report from the International Cardiac Arrest Registry. Critical Care 19:. <https://doi.org/10.1186/s13054-015-0904-y>

Kasaj A., Gortan-Kasaj A., Briseno-Marroquin B., Willershausen B. (2009) Treatment of severe localized periodontal destruction associated with a cemental tear: a case report and review of the literature. General dentistry 57:9

Kasbekar N. (2006) Tigecycline: A new glycylcycline antimicrobial agent. American Journal of Health-System Pharmacy 63:1235–1243. <https://doi.org/10.2146/ajhp050487>

Kaste S.C., Hudson M.M., Goodman P., et al (2009) Impact of radiation and chemotherapy on risk of dental abnormalities: A report from the childhood cancer survivor study. Cancer 115:5817–5827. <https://doi.org/10.1002/cncr.24670>

Kastovsky J, Linhartova PB, Musilova K, et al (2017) Lack of Association between BMP2/DLX3 Gene Polymorphisms and Dental Caries in Primary and Permanent Dentitions. Caries Research 51:590–595

Katon J.G., Callegari L.S., Bossick A.S., et al (2020) Association of Depression and Post-Traumatic Stress Disorder with Receipt of Minimally Invasive Hysterectomy for Uterine Fibroids: Findings from the U.S. Department of Veterans Affairs. Women’s Health Issues. <https://doi.org/10.1016/j.whi.2020.06.005>

Katsu Y., Nagahama Y., Yamashita M. (1999) Translational regulation of cyclin B mRNA by 17alpha,20beta-dihydroxy-4-pregnen-3-one (maturation-inducing hormone) during oocyte maturation in a teleost fish, the goldfish (Carassius auratus). Molecular and Cellular Endocrinology 158:79–85. <https://doi.org/10.1016/S0303-7207%2899%2900177-X>

Katsuta O, Hoshino N, Takeda M, et al (2003) A spontaneous mutation: amelogenesis imperfecta with cysts in rats. Toxicologic pathology 31:411–416

Katz C.R.T. (2012) Integrated approach to outpatient dental treatment of a patient with cerebral palsy: A case report. Special Care in Dentistry 32:210–217. <https://doi.org/10.1111/j.1754-4505.2012.00267.x>

Kaur R, Karadwal A, Sharma D, et al Esthetic and functional rehabilitation in a case of amelogenesis imperfecta

Kawai K, Tsuchitani Y (1994) Comparison of two methods for measuring generalized wear of dental materials. The Journal of Osaka University Dental School 34:1–8

Kawar N, Alrayyes S, Aljewari H (2018a) Sickle cell disease: An overview of orofacial and dental manifestations. Disease-a-month : DM 64:290–295. <https://doi.org/10.1016/j.disamonth.2017.12.004>

Kawar N, Alrayyes S, Yang B, Aljewari H (2018b) Oral health management considerations for patients with sickle cell disease. Disease-a-month : DM 64:296–301. <https://doi.org/10.1016/j.disamonth.2017.12.005>

Kayano T., Ochiai S., Kiyono K., et al (1989) Effects of Er:YAG laser irradiation on human extracted teeth. Kokubyo Gakkai zasshi The Journal of the Stomatological Society, Japan 56:381–392. <https://doi.org/10.5357/koubyou.56.381>

KCT0004164 (2019) Adjunctive use of enamel matrix derivatives to porcine derived xenograft for the treatment of one-wall intrabony defects: two-year longitudinal results of a randomized controlled clinical trial. http://www.who.int/trialsearch/Trial2.aspx?TrialID=KCT0004164

Keels MA (2019) Personalized dental caries management in children. Dental Clinics 63:621–629

Keenan AV (2014) No trial evidence for restorative interventions in children and adolescents with amelogenesis imperfecta. Evidence-based dentistry 15:45. <https://doi.org/10.1038/sj.ebd.6401023>

Keitel W, Potter G, Diemert D, et al (2019) A phase 1 study of the safety, reactogenicity, and immunogenicity of a Schistosoma mansoni vaccine with or without glucopyranosyl lipid A aqueous formulation (GLA-AF) in healthy adults from a non-endemic area. Vaccine 37:6500‐6509. <https://doi.org/10.1016/j.vaccine.2019.08.075>

Keith Jr. J.D., Caputo C., Petrungaro P., et al (2006) Clinical and histologic evaluation of a mineralized block allograft: Results from the developmental period (2001-2004). International Journal of Periodontics and Restorative Dentistry 26:321–327

Keles A, Pamukcu B, Işik F, et al (2001) Improving quality of life with a team approach: a case report. The International journal of adult orthodontics and orthognathic surgery 16:293–299

Kellerhoff N-M, Lussi A (2004) Die Molaren-Inzisiven-Hypomineralisation. Schweiz Monatsschr Zahnmed 114:243–249

Kellerhoff N.M., Lussi A. (2004) Die “Molaren-Inzisiven-Hypomineralisation”"Molar-incisor hypomineralization". Schweizer Monatsschrift fur Zahnmedizin = Revue mensuelle suisse d’odonto-stomatologie = Rivista mensile svizzera di odontologia e stomatologia / SSO 114:243–253

Kelley J (2008) Identification of a single birth cohort in Kenyapithecus kizili and the nature of sympatry between K. kizili and Griphopithecus alpani at Paşalar. Journal of human evolution 54:530–537. <https://doi.org/10.1016/j.jhevol.2007.08.005>

Kelly EC, Winick-Ng J, McClure JA, et al (2019) Hysterectomy in Ontario: A Population-Based Study of Outcomes and Complications in Minimally Invasive Compared with Abdominal Approaches. Journal of obstetrics and gynaecology Canada : JOGC = Journal d’obstetrique et gynecologie du Canada : JOGC 41:1168–1176. <https://doi.org/10.1016/j.jogc.2018.10.026>

Kemoli A (2008) Prevalence of molar incisor hypomineralisation in six to eight year-olds in two rural divisions in Kenya. East African medical journal 85:514–520

Kesmas S, Swasdison S, Yodsanga S, et al (2010) Esthetic alveolar ridge preservation with calcium phosphate and collagen membrane: preliminary report. Oral surgery, oral medicine, oral pathology, oral radiology, and endodontics 110:e24. <https://doi.org/10.1016/j.tripleo.2010.06.006>

Kettunen P, Furmanek T, Chaulagain R, et al (2011) Developmentally regulated expression of intracellular Fgf11-13, hormone-like Fgf15 and canonical Fgf16, -17 and -20 mRNAs in the developing mouse molar tooth. Acta odontologica Scandinavica 69:360–366. <https://doi.org/10.3109/00016357.2011.568968>

Keys T How To Anaesthetise A MIH Affected Molar

Khafagy Y, Abd Elfattah A, Moneir W, Salem E (2018) Leukocyte- and platelet-rich fibrin: a new graft material in endoscopic repair of spontaneous CSF leaks. European archives of oto-rhino-laryngology 1‐8. <https://doi.org/10.1007/s00405-018-5048-7>

Khalid F, Holguin F (2019) Idiopathic Hypereosinophilic Syndrome in an Elderly Female: A Case Report. The American journal of case reports 20:381–384. <https://doi.org/10.12659/AJCR.912747>

Khalil E, Ayed N, Musa A, et al (2005) Dichotomy of protective cellular immune responses to human visceral leishmaniasis. Clinical and experimental immunology 140:349‐353. <https://doi.org/10.1111/j.1365-2249.2005.02768.x>

Khan H. (2005) Evaluation of two different indices using photographic method of assessment of enamel defects (opacities). Journal of Postgraduate Medical Institute 19:149–156

Khan S, Ayub K (2020) Technique Tips. Dental Update 47:536–537

Khanna R, Khanna R, Pardhe N, et al (2016) Pure titanium membrane (Ultra – Ti®) in the treatment of periodontal osseous defects: a split-mouth comparative study. Journal of clinical and diagnostic research 10:ZC47‐ZC51. <https://doi.org/10.7860/JCDR/2016/18333.8487>

Khashayar G, Dozic A, Kleverlaan CJ, et al (2014) The influence of varying layer thicknesses on the color predictability of two different composite layering concepts. Dental materials : official publication of the Academy of Dental Materials 30:493–498. <https://doi.org/10.1016/j.dental.2014.02.002>

Khatri S, Madan K, Srinivasan S, Acharya S (2019) Retention of moisture-tolerant fluoride-releasing sealant and amorphous calcium phosphate-containing sealant in 6-9-year-old children: a randomized controlled trial. Journal of the Indian Society of Pedodontics and Preventive Dentistry 37:92‐98. <https://doi.org/10.4103/JISPPD.JISPPD_173_18>

Kher MS, Rao A (2019a) Contemporary Treatment Techniques in Pediatric Dentistry. Springer

Kher MS, Rao A (2019b) The Posterior Preformed Metal Crown (Stainless Steel Crown). In: Contemporary Treatment Techniques in Pediatric Dentistry. Springer, pp 99–116

Khodaeian N, Sabouhi M, Ataei E (2012) An interdisciplinary approach for rehabilitating a patient with amelogenesis imperfecta: a case report. Case reports in dentistry 2012:432108. <https://doi.org/10.1155/2012/432108>

Khodaeian N, Sobouhi M, Ataei E (2013) A review of amelogenesis imperfecta and its treatment planning. مجله دانشکده دندانپزشکی اصفهان 695–705

Khorashadi L, Wu CC, Betancourt SL, Carter BW (2015) Idiopathic pulmonary haemosiderosis: spectrum of thoracic imaging findings in the adult patient. Clinical radiology 70:459–465. <https://doi.org/10.1016/j.crad.2014.11.007>

Khurana D., Panayiotopoulos A., Mamkin A., et al (2013) Autoimmune hypoparathyroidism as the only clinical manifestation of autoimmune polyglandular syndrome type 1 (APS-1 ): A case report. Hormone Research in Paediatrics 80:212

Kiener P., Oetterli M., Mericske-Stern R. (2003) Prothetische Rehabilitation bei Ektodermaler Dysplasie. Ein FallberichtProsthetic rehabilitation in ectodermal dysplasia. Case report. Schweizer Monatsschrift fur Zahnmedizin = Revue mensuelle suisse d’odonto-stomatologie = Rivista mensile svizzera di odontologia e stomatologia / SSO 113:1076–1089

Kierdorf H, Filevych O, Lutz W, Kierdorf U (2016a) Dental Defects as a Potential Indicator of Chronic Malnutrition in a Population of Fallow Deer (Dama dama) from Northwestern Germany. Anatomical record (Hoboken, NJ : 2007) 299:1409–1423. <https://doi.org/10.1002/ar.23459>

Kierdorf H., Kierdorf U., Boyde A. (1997) A quantitative backscattered electron imaging study of hypomineralization and hypoplasia in fluorosed dental enamel of deer. Annals of Anatomy 179:405–412. <https://doi.org/10.1016/S0940-9602(97)80032-5>

Kierdorf H., Kierdorf U., Richards A., Josephsen K. (2004) Fluoride-induced alterations of enamel structure: An experimental study in the miniature pig. Anatomy and Embryology 207:463–474. <https://doi.org/10.1007/s00429-003-0368-8>

Kierdorf H, Kierdorf U, Richards A, Sedlacek F (2000) Disturbed enamel formation in wild boars (Sus scrofa L.) from fluoride polluted areas in Central Europe. The Anatomical record 259:12–24

Kierdorf H, Witzel C, Upex B, et al (2012) Enamel hypoplasia in molars of sheep and goats, and its relationship to the pattern of tooth crown growth. Journal of anatomy 220:484–495. <https://doi.org/10.1111/j.1469-7580.2012.01482.x>

Kierdorf U. (1988) UNTERSUCHUNGEN ZUM NACHWEIS IMMISSIONSBEDINGTER CHRONISCHER FLUORIDINTOXIKATION BEIM REH (CAPREOLUS CAPREOLUS L.)A study on chronic fluoride intoxication in roe deer (Capreolus capreolus L.) caused by immissions. 34:192–204

Kierdorf U, Death C, Hufschmid J, et al (2016b) Developmental and Post-Eruptive Defects in Molar Enamel of Free-Ranging Eastern Grey Kangaroos (Macropus giganteus) Exposed to High Environmental Levels of Fluoride. PloS one 11:e0147427. <https://doi.org/10.1371/journal.pone.0147427>

Kierdorf U, Kierdorf H, Fejerskov O (1993) Fluoride-induced developmental changes in enamel and dentine of European roe deer (Capreolus capreolus L.) as a result of environmental pollution. Archives of oral biology 38:1071–1081

Kierdorf U, Kierdorf H, Sedlacek F, Fejerskov O (1996) Structural changes in fluorosed dental enamel of red deer (Cervus elaphus L.) from a region with severe environmental pollution by fluorides. Journal of anatomy 188:

Kieswetter L., Walters T.D., Lara-Corrales I., et al (2019) Dieulafoy lesions and PHACE syndrome. Pediatric Dermatology 36:902–905. <https://doi.org/10.1111/pde.13922>

Kilinc G., Bulut G., Ertugrul F., et al (2019) Kanser tedavisi goren cocuklarda uzun sure sonra gorulen dis anomalileriLong-term dental anomalies after pediatric cancer treatment in children. Turkish Journal of Hematology 36:155–161. <https://doi.org/10.4274/tjh.galenos.2018.2018.0248>

Kilpatrick N (2009) New developments in understanding development defects of enamel: optimizing clinical outcomes. Journal of orthodontics 36:277–282. <https://doi.org/10.1179/14653120723310>

Kim MJ, Song J-S, Kim Y-J, et al (2020) Clinical Considerations for Dental Management of Children with Molar-Root Incisor Malformations. Journal of Clinical Pediatric Dentistry 44:55–59

Kim S, Kim E-Y, Jeong T-S, Kim J-W (2011) 2. International journal of paediatric dentistry 21:241–248. <https://doi.org/10.1111/j.1365-263X.2011.01126.x>

Kim T, Jeong I, Lee D, et al (2016) Prevalence and etiology of molar incisor hypomineralization in children aged 8-9 years. J Korean Acad Pediatr Dent 43:410–8

Kim T-H, Bae C-H, Jang E-H, et al (2012) Col1a1-cre mediated activation of β-catenin leads to aberrant dento-alveolar complex formation. Anatomy & cell biology 45:193–202. <https://doi.org/10.5115/acb.2012.45.3.193>

Kim Y.R., Yudina A., Figueiredo J., et al (2005) Detection of early antiangiogenic effects in human colon adenocarcinoma xenografts: In vivo changes of tumor blood volume in response to experimental VEGFR tyrosine kinase inhibitor. Cancer Research 65:9253–9260. <https://doi.org/10.1158/0008-5472.CAN-03-2619>

Kimoto S, Suga H, Yamaguchi M, et al (2003) Hypoplasia of primary and permanent teeth following osteitis and the implications of delayed diagnosis of a neonatal maxillary primary molar. International journal of paediatric dentistry 13:35–40

Kinaston RL, Roberts GL, Buckley HR, Oxenham M (2016) A bioarchaeological analysis of oral and physiological health on the south coast of New Guinea. American journal of physical anthropology 160:414–426. <https://doi.org/10.1002/ajpa.22978>

King NM (1982) Acrylic labial veneers for the restoration of hypoplastic teeth. Quintessence international, dental digest 13:1187–1192

King NM, Rule DC (1980) Restoration of hypoplastic teeth--a simplified acid etch technique. Journal of dentistry 8:81–84

King NM, Wei SH (1986) Developmental defects of enamel: a study of 12-year-olds in Hong Kong. Journal of the American Dental Association (1939) 112:835–839

King T, Humphrey LT, Hillson S (2005) Linear enamel hypoplasias as indicators of systemic physiological stress: evidence from two known age-at-death and sex populations from postmedieval London. American journal of physical anthropology 128:547–559

Kinumatsu T., Umehara K., Nagano K., Saito A. (2014) Periodontal therapy for severe chronic periodontitis with periodontal regeneration and different types of prosthesis. The Bulletin of Tokyo Dental College 55:217–224. <https://doi.org/10.2209/tdcpublication.55.217>

Kinzer GA (2010) COMMENTARY. A multidisciplinary approach to the functional and esthetic rehabilitation of amelogenesis imperfecta and open bite deformity: a case report. Journal of esthetic and restorative dentistry : official publication of the American Academy of Esthetic Dentistry . [et al] 22:294–296

Kirschneck C, Proff P (2020) Extraction of MIH-Affected Molars and Orthodontic Space Closure. In: Molar Incisor Hypomineralization. Springer, pp 187–196

Kirstein J, Douglas W, Thakur M, et al (2018) Immunogenicity of the CYD tetravalent dengue vaccine using an accelerated schedule: randomised phase II study in US adults. BMC infectious diseases 18:475. <https://doi.org/10.1186/s12879-018-3389-x>

Kirthiga M, Poornima P, Praveen R, et al (2015) Prevalence and severity of molar incisor hypomineralization in children aged 11-16 years of a city in Karnataka, Davangere. Journal of the Indian Society of Pedodontics and Preventive Dentistry 33:213–217. <https://doi.org/10.4103/0970-4388.160366>

Kirzioglu Z, Ulu KG, Sezer MT, Yüksel S (2009) The relationship of amelogenesis imperfecta and nephrocalcinosis syndrome. Medicina oral, patologia oral y cirugia bucal 14:e579

Kishaba T (2015) Practical management of Idiopathic Pulmonary Fibrosis. Sarcoidosis, vasculitis, and diffuse lung diseases : official journal of WASOG 32:90–98

Kishaba T (2019) Evaluation and management of Idiopathic Pulmonary Fibrosis. Respiratory investigation 57:300–311. <https://doi.org/10.1016/j.resinv.2019.02.003>

Kishaba T, Nei Y, Momose M, et al (2018) Clinical Characteristics Based on the New Criteria of Acute Exacerbation in Patients with Idiopathic Pulmonary Fibrosis. The Eurasian journal of medicine 50:6–10. <https://doi.org/10.5152/eurasianjmed.2018.17330>

Kishaba T, Shimaoka Y, Fukuyama H, et al (2015) Clinical characteristics of idiopathic pulmonary fibrosis patients with gender, age, and physiology staging at Okinawa Chubu Hospital. Journal of thoracic disease 7:843–849. <https://doi.org/10.3978/j.issn.2072-1439.2015.04.54>

Kiss A, Cucchiarini M, Menger MD, et al (2014) Enamel matrix derivative inhibits proteoglycan production and articular cartilage repair, delays the restoration of the subchondral bone and induces changes of the synovial membrane in a lapine osteochondral defect model in vivo. Journal of tissue engineering and regenerative medicine 8:41–49. <https://doi.org/10.1002/term.1495>

Kitamura M, Akamatsu M, Kawanami M, et al (2016) Randomized Placebo-Controlled and Controlled Non-Inferiority Phase III Trials Comparing Trafermin, a Recombinant Human Fibroblast Growth Factor 2, and Enamel Matrix Derivative in Periodontal Regeneration in Intrabony Defects. Journal of bone and mineral research 31:806‐814. <https://doi.org/10.1002/jbmr.2738>

Kitaoka T, Tajima T, Nagasaki K, et al (2017) Safety and efficacy of treatment with asfotase alfa in patients with hypophosphatasia: results from a Japanese clinical trial. Clinical endocrinology (no pagination): <https://doi.org/10.1111/cen.13343>

Kılınç G, Çetin M, Köse B, Ellidokuz H (2019) Prevalence, aetiology, and treatment of molar incisor hypomineralization in children living in Izmir City (Turkey). International journal of paediatric dentistry 29:775–782. <https://doi.org/10.1111/ipd.12508>

Kjær I, Steiniche K, Kortegaard U, et al (2012) Preeruptive intracoronal resorption observed in 13 patients. American journal of orthodontics and dentofacial orthopedics : official publication of the American Association of Orthodontists, its constituent societies, and the American Board of Orthodontics 142:129–132. <https://doi.org/10.1016/j.ajodo.2011.03.026>

Klaus K., Ruf S., Glanz T., et al (2020) Comparison of Quantitative light-induced fluorescence-digital (QLF-D) images and images of disclosed plaque for planimetric quantification of dental plaque in multibracket appliance patients. Scientific reports 10:4478. <https://doi.org/10.1038/s41598-020-61454-9>

Klein BR, Brown EN, Casden RS (2016) Preoperative macular spectral-domain optical coherence tomography in patients considering advanced-technology intraocular lenses for cataract surgery. Journal of cataract and refractive surgery 42:537–541. <https://doi.org/10.1016/j.jcrs.2016.01.036>

KLEIN IP, GONÇALVES MR, MARTINS MD, CARRARD VC (2015) Hiv-Infection Disclosed by Oral and Cutaneous Histoplasmosis. Oral Surgery, Oral Medicine, Oral Pathology and Oral Radiology 120:e80

Klepacki FH (1984) Orthodontic appliance

Klingberg G Utvärdering av tandvårderfarenhet och behandlingsresultat i en grupp 18-åringar med gravt mineraliseringsstörda sexårständer| Application

Klink A, Groten M, Huettig F (2018) Complete rehabilitation of compromised full dentitions with adhesively bonded all-ceramic single-tooth restorations: Long-term outcome in patients with and without amelogenesis imperfecta. Journal of dentistry 70:51–58. <https://doi.org/10.1016/j.jdent.2017.12.011>

Knake E (1989) [Individually manufactured ceramic facets. Case report]. Stomatologie der DDR 39:769–770

Knapp R (2019) The impact of dental caries and its treatment under general anaesthetic on the everyday lives of children and their families

Knezević A, Tarle Z, Pandurić V (2006) Esthetic reconstruction of teeth in patient with dentinogenesis imperfecta--a case report. Collegium antropologicum 30:231–234

Kobayashi E, Fujioka-Kobayashi M, Saulacic N, et al (2019) Effect of enamel matrix derivative liquid in combination with a natural bone mineral on new bone formation in a rabbit GBR model. Clinical oral implants research 30:542–549. <https://doi.org/10.1111/clr.13440>

Koch F (2020a) Therapie der verminderten Schmelzmineralisation. Der Freie Zahnarzt 64:70–71

Koch F, Meyer N, Valdec S, et al (2020) Development and application of a 3D periodontal in vitro model for the evaluation of fibrillar biomaterials. BMC oral health 20:148. <https://doi.org/10.1186/s12903-020-01124-4>

Koch G (2003) Prevalence of enamel mineralisation disturbances in an area with 1-1.2 ppm F in drinking water. Review and summary of a report published in Sweden in 1981. European journal of paediatric dentistry 4:127–128

Koch G (2020b) MIH: An Introduction. In: Molar Incisor Hypomineralization. Springer, pp 3–10

Koch M.J., Buhrer R., Pioch T., Scharer K. (1999) Enamel hypoplasia of primary teeth in chronic renal failure. Pediatric Nephrology 13:68–72. <https://doi.org/10.1007/s004670050566>

Kodaka T., Mori R., Takiguchi R., Higashi S. (1995) The structural patterns and mineralization values of prismless enamel, a case of mild enamel hypoplasia. The Bulletin of Tokyo Dental College 36:33–42

Kodaka T, Ohohara Y, Debari K (1992) Scanning electron microscopy and energy-dispersive X-ray microanalysis studies of early dental calculus on resin plates exposed to human oral cavities. Scanning microscopy 6:475

Koehne T., Jeschke A., Petermann F., et al (2016) Rsk2, the Kinase Mutated in Coffin-Lowry Syndrome, Controls Cementum Formation. Journal of dental research 95:752–760. <https://doi.org/10.1177/0022034516634329>

Koehne T., Zustin J., Amling M., Friedrich R.E. (2020) Radiological and Histopathological Features of Internal Tooth Resorption. In vivo (Athens, Greece) 34:1875–1882. <https://doi.org/10.21873/invivo.11983>

Kogon S.L. (1986) The prevalence, location and conformation of palato-radicular grooves in maxillary incisors. Journal of periodontology 57:231–234. <https://doi.org/10.1902/jop.1986.57.4.231>

Koh C, Bates E, Broughton E, et al (2010) Genetic integration of molar cusp size variation in baboons. American journal of physical anthropology 142:246–260. <https://doi.org/10.1002/ajpa.21221>

Koh M.Y., Ohtsuki C., Miyazaki T. (2011) Modification of polyglutamic acid with silanol groups and calcium salts to induce calcification in a simulated body fluid. Journal of biomaterials applications 25:581–594. <https://doi.org/10.1177/0885328209357111>

Kohlboeck G, Heitmueller D, Neumann C, et al (2013) Is there a relationship between hyperactivity/inattention symptoms and poor oral health? Results from the GINIplus and LISAplus study. Clinical oral investigations 17:1329–1338. <https://doi.org/10.1007/s00784-012-0829-7>

Koker SA, Gözmen S, Oymak Y, et al (2017) Idiopathic Pulmonary Hemosiderosis Mimicking Iron Deficiency Anemia: A Delayed Diagnosis? Hematology reports 9:7048. <https://doi.org/10.4081/hr.2017.7048>

Kolb M, Bondue B, Pesci A, et al (2018) Acute exacerbations of progressive-fibrosing interstitial lung diseases. European respiratory review : an official journal of the European Respiratory Society 27:. <https://doi.org/10.1183/16000617.0071-2018>

Koleoso DCU, Shaba OP, Isiekwe MC (2004) Prevalence of intrinsic tooth discolouration among 11-16 year-old Nigerians. Odonto-stomatologie tropicale = Tropical dental journal 27:35–39

Komita H., Homma S., Ohno T., et al (2006) Interferon-gamma produced by interleukin-12-activated tumor infiltrating CD8+T cells directly induces apoptosis of mouse hepatocellular carcinoma. Journal of Hepatology 45:662–672. <https://doi.org/10.1016/j.jhep.2006.05.018>

Komiya-Ito A, Tomita S, Kinumatsu T, et al (2013) Longitudinal supportive periodontal therapy for severe chronic periodontitis with furcation involvement: a 12-year follow-up report. The Bulletin of Tokyo Dental College 54:243–250

Kondoh Y, Cottin V, Brown KK (2017) Recent lessons learned in the management of acute exacerbation of idiopathic pulmonary fibrosis. European respiratory review : an official journal of the European Respiratory Society 26:. <https://doi.org/10.1183/16000617.0050-2017>

Koning SW, Ellerbroek PM, Leenen LPH (2015) Indoor fire in a nursing home: evaluation of the medical response to a mass casualty incident based on a standardized protocol. European journal of trauma and emergency surgery : official publication of the European Trauma Society 41:167–178. <https://doi.org/10.1007/s00068-014-0446-z>

Konis AB (1993) Treatment of enamel hypoplasia in young adults. The New York state dental journal 59:38–40

Konstantinova D., Arnautska H. (2014) Orthodontic-prosthetic approach in thetreatment of complex clinical cases. Journal of IMAB - Annual Proceeding (Scientific Papers) 20:469–472. <https://doi.org/10.5272/jimab.2014201.469>

Kontham UR, Tiku AM, Damle SG, Kalaskar RR (2005) Apexogenesis of a symptomatic mandibular first permanent molar with calcium hydroxide pulpotomy. Quintessence international (Berlin, Germany : 1985) 36:653–657

Koop R, Merheb J, Quirynen M (2012) Periodontal regeneration with enamel matrix derivative in reconstructive periodontal therapy: a systematic review. Journal of periodontology 83:707–720. <https://doi.org/10.1902/jop.2011.110266>

Kopperud SE, Pedersen CG, Espelid I (2016) Treatment decisions on Molar-Incisor Hypomineralization (MIH) by Norwegian dentists - a questionnaire study. BMC oral health 17:3. <https://doi.org/10.1186/s12903-016-0237-5>

Kopperud SE, Pedersen CG, Espelid I (2017) Treatment decisions on Molar-Incisor Hypomineralization (MIH) by Norwegian dentists–a questionnaire study. BMC oral health 17:1–7

Korbmacher HM, Lemke R, Kahl-Nieke B (2007) Progressive pre-eruptive crown resorption in autosomal recessive generalized hypoplastic amelogenesis imperfecta. Oral surgery, oral medicine, oral pathology, oral radiology, and endodontics 104:540–544

Korolenkova M.V., Starikova N.V., Ageeva L.V. (2016) Risk factors for teeth aplasia and hypoplasia in cleft lip and palate children. Stomatologiia 95:59–62. <https://doi.org/10.17116/stomat201695159-62>

Korolenkova MV, Starikova NV, Udalova NV (2019) The role of external aetiological factors in dental anomalies in non-syndromic cleft lip and palate patients. European archives of paediatric dentistry : official journal of the European Academy of Paediatric Dentistry 20:105–111. <https://doi.org/10.1007/s40368-018-0397-x>

Koruyucu M, Kasimoğlu Y, Seymen F, et al (2018a) Rethinking isolated cleft lip and palate as a syndrome. Oral surgery, oral medicine, oral pathology and oral radiology 125:307–312

Koruyucu M, Seymen F, Gencay G, et al (2018b) Nephrocalcinosis in Amelogenesis Imperfecta Caused by the FAM20A Mutation. Nephron 139:189–196. <https://doi.org/10.1159/000486607>

Kostoulas I, Kourtis S, Andritsakis D, Doukoudakis A (2005) Functional and esthetic rehabilitation in amelogenesis imperfecta with all-ceramic restorations: a case report. Quintessence international (Berlin, Germany : 1985) 36:329–338

Kostrzewska M., Toporowska-Kowalska E., Kudzin J., Wasowska-Krolikowska K. (2007) Obraz kliniczny celiakii rozpoznanej po 4 roku zycia w materiale wlasnym kliniki alergologii, gastroenterologii i zywienia dzieci uniwersytetu medycznego w LodziClinical manifestation of celiac disease diagnosed in children of 4 years and above in self-study. Przeglad Pediatryczny 37:237–243

Kotilainen J., Holtta P., Mikkonen T., et al (1995) Craniofacial and dental characteristics of Silver-Russell syndrome. American Journal of Medical Genetics 56:229–236. <https://doi.org/10.1002/ajmg.1320560223>

Kotsomitis N., Freer T.J. (1997) Inherited dental anomalies and abnormalities. ASDC journal of dentistry for children 64:405–408

Koudstaal M.J., Wolvius E.B., van der Wal K.G.H., et al (2009) Stability, tipping and relapse of bone-borne versus tooth-borne surgically assisted rapid maxillary expansion; a prospective randomized patient trial. International Journal of Oral and Maxillofacial Surgery 38:308–315. <https://doi.org/10.1016/j.ijom.2009.02.012>

Kraemer N (2011) Molar Incisor Hypomineralization: Etiology, Classification, Treatment and Limits. GEORG THIEME VERLAG KG RUDIGERSTR 14, D-70469 STUTTGART, GERMANY, pp 193–193

Krahel J.A., Baran A., Flisiak I. (2016) Skorne manifestacje w przebiegu autoimmunologicznego zespolu niedoczynnosci wielogruczolowej typu 1 - Opis przypadku i przeglad pismiennictwaCutaneous manifestations of autoimmune polyglandular syndrome type 1 - Case report and literature review. Przeglad Dermatologiczny 103:354–361. <https://doi.org/10.5114/dr.2016.62885>

Kralick AE, Loring Burgess M, Glowacka H, et al (2017) A radiographic study of permanent molar development in wild Virunga mountain gorillas of known chronological age from Rwanda. American journal of physical anthropology 163:129–147. <https://doi.org/10.1002/ajpa.23192>

Krämer N, Bui Khac N-HN, Lücker S, et al (2018) Bonding strategies for MIH-affected enamel and dentin. Dental materials : official publication of the Academy of Dental Materials 34:331–340. <https://doi.org/10.1016/j.dental.2017.11.015>

Krämer N, Frankenberger R (2005) Clinical performance of bonded leucite-reinforced glass ceramic inlays and onlays after eight years. Dental materials 21:262‐271. <https://doi.org/10.1016/j.dental.2004.03.009>

Krasuska-Slawinska E., Brozyna A., Dembowska-Baginska B., Olczak-Kowalczyk D. (2016) Antineoplastic chemotherapy and congenital tooth abnormalities in children and adolescents. Wspolczesna Onkologia 20:394–401. <https://doi.org/10.5114/wo.2016.64602>

Krejci I., Lutz F., Sener B., Jenss J. (1991) Rontgenopazitat von zahnfarbenen Inlaymaterialien und KompositzementenThe x-ray opacity of tooth-coloring inlay materials and composite cements. Schweizer Monatsschrift fur Zahnmedizin = Revue mensuelle suisse d’odonto-stomatologie = Rivista mensile svizzera di odontologia e stomatologia / SSO 101:299–304

Krieger O, Matuliene G, Hüsler J, et al (2009) Failures and complications in patients with birth defects restored with fixed dental prostheses and single crowns on teeth and/or implants. Clinical oral implants research 20:809–816. <https://doi.org/10.1111/j.1600-0501.2009.01720.x>

Krishnan R, Ramesh M, Chalakkal P (2015) Prevalence and characteristics of MIH in school children residing in an endemic fluorosis area of India: an epidemiological study. European Archives of Paediatric Dentistry 16:455–460

KRISHNAN R, WADEI MMA, QAHTHANI MTA, et al (2020) Assessment of Enamel Permeability Using Scanning Electron Microscopy in Permanent Teeth with and without Molar Incisor Hypomineralisation-An In Vivo Study. Journal of Clinical & Diagnostic Research 14:

Kroll RG, Stone JH (1976) The exfoliating hypoplastic tooth. A case report: with a low bow to the poetic muse. The New York state dental journal 42:544–545

Krungkasem C, Ohira T, Yang W-J, et al (2002) Identification of two distinct molt-inhibiting hormone-related peptides from the giant tiger prawn Penaeus monodon. Marine biotechnology (New York, NY) 4:132–140

Kubota M, Chiba M, Obinata M, et al (2004) Establishment of Periodontal Ligament Cell Lines from Temperature-Sensitive Simian Virus 40 Large T-antigen Transgenic Rats. Cytotechnology 44:55–65. <https://doi.org/10.1023/B:CYTO.0000043412.08814.80>

Küçükcan T, Ziyal T, Sennaroglu L, et al (2013) Are Muslim Democrats a Threat to Secularism and Freedom of Religion? The Turkish Case. The Future of Religious Freedom: Global Challenges 270–289

Kucukesmen C., Edotan Y. (2012) One-year evaluation of class-i composite restorations in fluorosed and non-fluorosed permanent teeth. Fluoride 45:161–162

Kuderewska S, Stawiecka M, Milewska R, et al (2019) Hipomineralizacja trzonowcowo-siekaczowa (MIH)–etiologia, obraz kliniczny, leczenie. Nowa Stomatologia

Kuga T, Sasaki M, Mikami T, et al (2016) FAM83H and casein kinase I regulate the organization of the keratin cytoskeleton and formation of desmosomes. Scientific reports 6:26557. <https://doi.org/10.1038/srep26557>

Kühnisch J Ätiologie und Therapie der Molaren-Inzisiven-Hypomineralisation

Kuhnisch J, Dietz W, Stosser L, et al (2007) Effects of dental probing on occlusal surfaces--a scanning electron microscopy evaluation. Caries research 41:43‐48. <https://doi.org/10.1159/000096104>

Kühnisch J, Kabary L, Malyk Y, et al (2018) Relationship between caries experience and demarcated hypomineralised lesions (including MIH) in the permanent dentition of 15-year-olds. Clinical oral investigations 22:2013–2019

Kuhnisch J., Mach D., Thiering E., et al (2014) Respiratory diseases are associated with molar-incisor hypomineralizations. Swiss dental journal 124:286–293

Kühnisch J, Thiering E, Heinrich-Weltzien R, et al (2017) Fluoride/vitamin D tablet supplementation in infants-effects on dental health after 10 years. Clinical oral investigations 21:2283–2290. <https://doi.org/10.1007/s00784-016-2021-y>

Kühnisch J, Thiering E, Kratzsch J, et al (2015) Elevated serum 25 (OH)-vitamin D levels are negatively correlated with molar-incisor hypomineralization. Journal of dental research 94:381–387

Kuijpers MAR, Loomans B (2015) [Combined orthodontic and restorative treatment]. Nederlands tijdschrift voor tandheelkunde 122:575–581. <https://doi.org/10.5177/ntvt.2015.11.15190>

Kuklik HH, Cruz ITSA, Celli A, et al (2020) MOLAR INCISOR HYPOMINERALIZATION AND CELIAC DISEASE. Arquivos de gastroenterologia 57:167–171. <https://doi.org/10.1590/S0004-2803.202000000-31>

Kulas A, Illge C, Bekes K, et al (2016) Structural color changes in permanent enamel of patients with cleft lip and palate: a case-control study. Journal of orofacial orthopedics = Fortschritte der Kieferorthopadie : Organ/official journal Deutsche Gesellschaft fur Kieferorthopadie 77:45–51. <https://doi.org/10.1007/s00056-015-0007-z>

Kulczyk T. (2006) Evaluation of the results of periodontal treatment by means of digital subtraction of radiographic images. Advances in medical sciences 126–129

Kulkarni M., Agrawal T., Kheur S. (2011) Tooth agenesis: Newer concept. Journal of Clinical Pediatric Dentistry 36:65–70. <https://doi.org/10.17796/jcpd.36.1.p74362q544210p33>

Kulkarni RS, Pimpale SK, Powar SN (2016) Interdisciplinary approach to complete-mouth rehabilitation: a clinical report. General dentistry 64:e10

Kulkarni R.S., Pimpale S.K., Powar S.N. (2016) Interdisciplinary approach to complete-mouth rehabilitation: a clinical report. General dentistry 64:

Kumar H, Palamara J, Burrow MF, Manton DJ (2012) Resin infiltration-taking the first steps to filling the holes in cheese molars. Annals of the Royal Australasian College of Dental Surgeons 21:120–123

Kumar H, Palamara JEA, Burrow MF, Manton DJ (2017a) An investigation into the effect of a resin infiltrant on the micromechanical properties of hypomineralised enamel. International journal of paediatric dentistry 27:399–411. <https://doi.org/10.1111/ipd.12272>

Kumar S, Gupta S (2009) The restoration of function and esthetics of a patient with amelogenesis imperfecta using a combination of orthodontic and prosthodontic treatment: a case report. The journal of contemporary dental practice 10:E079

Kumar S, Mathur RM, Chandra S, Jaiswal JN (1990) Pulp calcifications in primary teeth. The Journal of pedodontics 14:93–96

Kumar S, Pai D, Saran R (2017b) Oral Health Characteristics and Dental Rehabilitation of Children with Global Developmental Delay. Case reports in dentistry 2017:5486327. <https://doi.org/10.1155/2017/5486327>

Kumari A., Kumari J., Sharma S., Singh E. (2012) Genesis of axenfeld rieger syndrome: A review. International Journal of Pharmaceutical Sciences Review and Research 14:64–68

Kumari M., Sharma A., Jagannadham M.V. (2012) Religiosin B, a milk-clotting serine protease from Ficus religiosa. Food Chemistry 131:1295–1303. <https://doi.org/10.1016/j.foodchem.2011.09.122>

Kumazawa K., Shintani S., Sawada T., Yanagisawa T. (2011) Analysis of effects of amoxicillin on rat incisor. Connective Tissue Research 52:79. <https://doi.org/10.3109/03008207.2010.531333>

Kumazawa K., Shintani S., Sawada T., Yanagisawa T. (2012) Effect of single-dose amoxicillin on rat incisor odontogenesis: A morphological study. Clinical Oral Investigations 16:835–842. <https://doi.org/10.1007/s00784-011-0581-4>

Künzel PW, Padrón FS (1974) [Relation between the frequency and intensity of enamel white spots caused by dental fluorosis and the fluoride concentration in Cuban drinking water]. Revista cubana de estomatologia 11:165–174

Kunzel P.W., Padron F.S. (1974) Relacion entre la frecuencia e intensidad de las manchas blancas del esmalte, causadas por la fluorosis dental y la concentracion de fluor en le agua potable de CubaRelation between the frequency and intensity of enamel white spots caused by dental fluorosis and the fluoride concentration in Cuban drinking water. Revista cubana de estomatologia 11:165–174

Kurbad A, Reichel K (2005) CAD/CAM-manufactured restorations made of lithium disilicate glass ceramics. International journal of computerized dentistry 8:337–348

Kurji ZA, Sigal MJ, Andrews P, Titley K (2011) A retrospective study of a modified 1-minute formocresol pulpotomy technique part 2: effect on exfoliation times and successors. Pediatric dentistry 33:139–143

Kuroda S., Wazen R., Sellin K., et al (2011) Ameloblastin is not implicated in bone remodelling and repair. European Cells and Materials 22:56–67. <https://doi.org/10.22203/eCM.v022a05>

Kurtiş B, Unsal B, Cetiner D, et al (2002) Effect of polylactide/glycolide (PLGA) membranes loaded with metronidazole on periodontal regeneration following guided tissue regeneration in dogs. Journal of periodontology 73:694–700

Kuru B, Yilmaz S, Argin K, Noyan U (2006) Enamel matrix derivative alone or in combination with a bioactive glass in wide intrabony defects. Clinical oral investigations 10:227‐234. <https://doi.org/10.1007/s00784-006-0052-5>

Kusku O.O., Caglar E., Sandalli N. (2008) The prevalence and aetiology of molar-incisor hypomineralisation in a group of children in Istanbul. European journal of paediatric dentistry : official journal of European Academy of Paediatric Dentistry 9:139–144

Kuykendall KL (1996) Dental development in chimpanzees (Pan troglodytes): the timing of tooth calcification stages. American journal of physical anthropology 99:135–157

Kwok-Tung L, King NM (2006) The restorative management of amelogenesis imperfecta in the mixed dentition. The Journal of clinical pediatric dentistry 31:130–135

Kwon M, Shin J, Kim J, Kim S (2016) An epidemiological study on the dental treatment needs of adolescents in Yangsan. J Korean Acad Pediatr Dent 43:354–364

Kwon SJ, Lawson NC, McLaren EE, et al (2018) Comparison of the mechanical properties of translucent zirconia and lithium disilicate. The Journal of prosthetic dentistry 120:132–137. <https://doi.org/10.1016/j.prosdent.2017.08.004>

Kyle B., Larsen C.S., Schepartz L.A. (2011) Reconstructing health at Apollonia, Albania: Impacts of Corinthian colonization. American Journal of Physical Anthropology 144:194. <https://doi.org/10.1002/ajpa.21502>

La Monaca G, Pranno N, Vozza I, et al (2019) Sequelae in permanent teeth after traumatic injuries to primary dentition. Minerva stomatologica 68:332–340. <https://doi.org/10.23736/S0026-4970.19.04297-3>

Laaksovirta S., Laurila M., Isotalo T., et al (2002) Rabbit muscle and urethral in situ biocompatibility properties of the self-reinforced L-lactide-glycolic acid copolymer 80:20 spiral stent. Journal of Urology 167:1527–1531. <https://doi.org/10.1016/S0022-5347(05)65357-8>

Lachowski KM, Botta SB, Lascala CA, et al (2013) Study of the radio-opacity of base and liner dental materials using a digital radiography system. Dento maxillo facial radiology 42:20120153. <https://doi.org/10.1259/dmfr.20120153>

Ladhani S., Shingadia D., Aibara R.J., Riordan F.A.I. (2007) Imported malaria in children: a review of clinical studies. Lancet Infectious Diseases 7:349–357. <https://doi.org/10.1016/S1473-3099%2807%2970110-X>

Laffranchi Z., Manasse G.C., Salzani L., Milella M. (2019) Patterns of funerary variability, diet, and developmental stress in a Celtic population from NE Italy (3rd-1st c BC). PLoS ONE 14:. <https://doi.org/10.1371/journal.pone.0214372>

Lafzi A, Farahani RM, Tubbs RS, et al (2007) Enamel matrix derivative Emdogain as an adjuvant for a laterally-positioned flap in the treatment of gingival recession: an electron microscopic appraisal. Folia morphologica 66:100–103

Lagarde A, Kerebel B, Le Cabellec MT (1989) [Anomalies of enamel formation in subjects with maxillary clefts]. Bulletin du Groupement international pour la recherche scientifique en stomatologie & odontologie 32:191–197

Lagarde A., Kerebel B., Le Cabellec M.T. (1989) Anomalies de formation de l’email chez les sujets presentant des fentes maxillairesAnomalies of enamel formation in subjects with maxillary clefts. Bulletin du Groupement international pour la recherche scientifique en stomatologie & odontologie 32:191–197

Lagarde M, Vennat E, Attal J-P, Dursun E (2020) Strategies to optimize bonding of adhesive materials to molar-incisor hypomineralization-affected enamel: A systematic review. International journal of paediatric dentistry 30:405–420

Lago JD (2017) Incidência da hipomineralização molar-incisivo em Araraquara e análise de fatores associados

Lago M, Mozzaquatro LR, Rodrigues C, et al (2017) Influence of Bleaching Agents on Color and Translucency of Aged Resin Composites. Journal of esthetic and restorative dentistry : official publication of the American Academy of Esthetic Dentistry . [et al] 29:368–377. <https://doi.org/10.1111/jerd.12261>

Lahdesmaki T., Poyhonen H., Peltola V., et al (2016) Safety of high dose oral and intravenous doxycycline in treatment of pediatric central nervous system infections. European Journal of Pediatrics 175:1460. <https://doi.org/10.1007/s00431-016-2785-8>

Lai PY, Seow WK, Tudehope DI, Rogers Y (1997) Enamel hypoplasia and dental caries in very-low birthweight children: a case-controlled, longitudinal study. Pediatric dentistry 19:42–49

Laish-Farkash A., Matetzky S., Kassem S., et al (2007) Therapeutic hypothermia for comatose survivors after cardiac arrest. Israel Medical Association Journal 9:252–256

Laisi S., Ess A., Sahlberg C., et al (2009) Amoxicillin may cause molar incisor hypomineralization. Journal of dental research 88:132–136

Lalau CV (2015) Hipomineralização Molar-Incisivo: Uma revisão de literatura

Lam A, David D, Townsend G, Anderson P (2010) Van der Woude syndrome: dentofacial features and implications for clinical practice. Australian dental journal 55:51–58

Lam WYH, Ho EHT, Pow EHN (2014) Rehabilitation of molar-incisor hypomineralization (MIH) complicated with localized tooth surface loss: a case report. Quintessence international (Berlin, Germany : 1985) 45:377–379. <https://doi.org/10.3290/j.qi.a31540>

Lamarca G de A, Vettore MV, Monteiro da Silva AM (2018) The influence of stress and anxiety on the expectation, perception and memory of dental pain in schoolchildren. Dentistry journal 6:60

Lamb DJ (1976) The treatment of amelogenesis imperfecta. The Journal of prosthetic dentistry 36:286–291

Lambrechts P, Mattar D, De Munck J, et al (2002) Air-abrasion enamel microsurgery to treat enamel white spot lesions of traumatic origin. Journal of esthetic and restorative dentistry : official publication of the American Academy of Esthetic Dentistry . [et al] 14:167–187

Laouina S., Al Bouzidi A., Amezian R., et al (2015) A rare association - Amelogenesis imperfecta, platispondyly and bicytopenia: A case report. Journal of Medical Case Reports 9:. <https://doi.org/10.1186/s13256-015-0724-3>

Large JF, Hasmun N, Lawson JA, et al (2020) What children say and clinicians hear: accounts relating to incisor hypomineralisation of cosmetic concern. European archives of paediatric dentistry : official journal of the European Academy of Paediatric Dentistry 21:185–191. <https://doi.org/10.1007/s40368-019-00465-1>

Larmas M., Hietala E.L., Simila S., Pajari U. (1991) Oral manifestations of familial hypophosphatemic rickets after phosphate supplement therapy: a review of the literature and report of case. ASDC journal of dentistry for children 58:328–334

Larsen MJ, Fejerskov O (1989) Chemical and structural challenges in remineralization of dental enamel lesions. Scandinavian journal of dental research 97:285–296

Larsen T.H., Jemec G.B.E. (2003) Acne: Comparing hormonal approaches to antibiotics and isotretinoin. Expert Opinion on Pharmacotherapy 4:1097–1103. <https://doi.org/10.1517/14656566.4.7.1097>

Latz T, Schwarz F, Sculean A, Becker J (2003) Treatment of intraossary defects with an Er: YAG laser in combination with an enamel matrix protein. Parodontologie 14:430

Laugel-Haushalter V., Schaefer E., Stoetzel C., et al (2019) A new SLC10A7 homozygous missense mutation responsible for a milder phenotype of skeletal dysplasia with amelogenesis imperfecta. Frontiers in Genetics 10:. <https://doi.org/10.3389/fgene.2019.00504>

Lauritano D., Petruzzi M. (2012) Decayed, missing and filled teeth index and dental anomalies in long-term survivors leukaemic children: A prospective controlled study. Medicina Oral, Patologia Oral y Cirugia Bucal 17:. <https://doi.org/10.4317/medoral.17955>

Laverty DP, Thomas BM (2016a) The Prosthodontic Pathway for Patients with Anomalies Affecting Tooth Structure. Dental update 43:356

Laverty DP, Thomas MBM (2016b) The restorative management of microdontia. British dental journal 221:160–166. <https://doi.org/10.1038/sj.bdj.2016.595>

Law V, Seow WK (2006) A longitudinal controlled study of factors associated with mutans streptococci infection and caries lesion initiation in children 21 to 72 months old. Pediatric dentistry 28:58–65

Lawson J, Warren JJ, Levy SM, et al (2008) Relative esthetic importance of orthodontic and color abnormalities. The Angle orthodontist 78:889–894. <https://doi.org/10.2319/080207-361.1>

Lazor R, Bonetti A, Nicod LP (2010) [Acute exacerbations of idiopathic pulmonary fibrosis]. Revue medicale suisse 6:2228

LE BAS V (2006) Molar-incisor hypomineralisation “MIH”. About 6 clinical cases.

Le Cabec A., Tafforeau P., Smith T.M., et al (2014) Dental development of the Australopithecus sediba juvenile MH1 determined from synchrotron virtual paleohistology. American Journal of Physical Anthropology 153:166. <https://doi.org/10.1002/ajpa/22488>

Le Cabec A., Tafforeau P., Tang N. (2015) Accessing developmental information of fossil hominin teeth using new synchrotron microtomography-based visualization techniques of dental surfaces and interfaces. PLoS ONE 10:. <https://doi.org/10.1371/journal.pone.0123019>

Le MH, Nakano Y, Abduweli Uyghurturk D, et al (2017) Fluoride Alters Klk4 Expression in Maturation Ameloblasts through Androgen and Progesterone Receptor Signaling. Frontiers in physiology 8:925. <https://doi.org/10.3389/fphys.2017.00925>

Leal SC, Oliveira TRM, Ribeiro APD (2017) Do parents and children perceive molar–incisor hypomineralization as an oral health problem? International journal of paediatric dentistry 27:372–379

Lee E, Kim Y, Kim H, Nam S (2015) Abberant Root Morphology in the Permanent First Molars. J Korean Acad Pediatr Dent 42:172–179

Lee E.-J., Lee I.-J., Kim W.W., et al (2014) Inhibition of IL-17A suppresses enhanced-tumor growth in low dose pre-irradiated tumor beds. PLoS ONE 9:. <https://doi.org/10.1371/journal.pone.0106423>

Lee H-S, Kim S-H, Kim S-O, et al (2014) A new type of dental anomaly: molar-incisor malformation (MIM). Oral surgery, oral medicine, oral pathology and oral radiology 118:101. <https://doi.org/10.1016/j.oooo.2014.03.014>

Lee I.P., Lucier G.W. (1976) The potentiation of barbiturate induced narcosis by procarbazine. Journal of Pharmacology and Experimental Therapeutics 196:586–593

Lee J, Kim YH, Kang JY, et al (2019) Korean Guidelines for Diagnosis and Management of Interstitial Lung Diseases: Part 3. Idiopathic Nonspecific Interstitial Pneumonia. Tuberculosis and respiratory diseases 82:277–284. <https://doi.org/10.4046/trd.2018.0092>

Lee J, Messer L (2010) Intake of sweet drinks and sweet treats versus reported and observed caries experience. european archives of Paediatric Dentistry 11:5–17

Lee J.-H., Kim D.-H., Jeong S.-N. (2020) Adjunctive use of enamel matrix derivatives to porcine-derived xenograft for the treatment of one-wall intrabony defects: Two-year longitudinal results of a randomized controlled clinical trial. Journal of Periodontology 91:880–889. <https://doi.org/10.1002/JPER.19-0432>

Lee J.S., Fitzgibbon E.J., Chen Y.R., et al (2012) Clinical guidelines for the management of craniofacial fibrous dysplasia. Orphanet Journal of Rare Diseases 7:. <https://doi.org/10.1186/1750-1172-7-S1-S2>

Lee K-E, Lee S-K, Jung S-E, et al (2011a) A novel mutation in the AMELX gene and multiple crown resorptions. European journal of oral sciences 119:. <https://doi.org/10.1111/j.1600-0722.2011.00858.x>

Lee KJ, Watson RD (2002) Antipeptide antibodies for detecting crab (Callinectes sapidus) molt-inhibiting hormone. Peptides 23:853–862

Lee K.J., Watson R.D. (2002) Antipeptide antibodies for detecting crab (Callinectes sapidus) molt-inhibiting hormone. Peptides 23:853–862. <https://doi.org/10.1016/S0196-9781%2802%2900011-6>

Lee R.Z., Veale D.J. (2002) Management of spondyloarthropathy: New pharmacological treatment options. Drugs 62:2349–2359. <https://doi.org/10.2165/00003495-200262160-00003>

Lee SG, Mykles DL (2006) Proteomics and signal transduction in the crustacean molting gland. Integrative and comparative biology 46:965–977. <https://doi.org/10.1093/icb/icl047>

Lee S-K, Lee K-E, Hwang Y-H, et al (2011b) Identification of the DSPP mutation in a new kindred and phenotype-genotype correlation. Oral diseases 17:314–319. <https://doi.org/10.1111/j.1601-0825.2010.01760.x>

Leevailoj C, Lawanrattanakul S, Mahatumarat K Amelogenesis Imperfecta: Case Study. Operative dentistry 42:457–469. <https://doi.org/10.2341/13-256-S>

Lehrhaupt N.B., Brownstein C.N., Deasy M.J. (1997) Osseous repair of a lateral periodontal cyst. Journal of periodontology 68:608–611

Leite G.A.S., Sawan R.M.M., Teofilo J.M., et al (2011) Exposure to lead exacerbates dental fluorosis. Archives of Oral Biology 56:695–702. <https://doi.org/10.1016/j.archoralbio.2010.12.011>

Leite G.M.F., Lana J.P., Manzi F.R., et al (2014) Anatomic variations and lesions of the mandibular canal detected by cone beam computed tomography. Surgical and Radiologic Anatomy 36:795–804. <https://doi.org/10.1007/s00276-013-1247-5>

Leknes K, Amarante E, Price D, et al (2005) Coronally positioned flap procedures with or without a biodegradable membrane in the treatment of human gingival recession. A 6-year follow-up study. Journal of clinical periodontology 32:518‐529. <https://doi.org/10.1111/j.1600-051X.2005.00706.x>

Leknes K, Andersen K, Bøe O, et al (2009) Enamel matrix derivative versus bioactive ceramic filler in the treatment of intrabony defects: 12-month results. Journal of periodontology 80:219‐227. <https://doi.org/10.1902/jop.2009.080236>

Leknes KN, Lie T, Selvig KA (1996) Cemental tear: a risk factor in periodontal attachment loss. Journal of periodontology 67:583–588

Lekovic V., Camargo P.M., Weinlaender M., et al (2000) A comparison between enamel matrix proteins used alone or in combination with bovine porous bone mineral in the treatment of intrabony periodontal defects in humans. Journal of periodontology 71:1110–1116

Lekovic V., Camargo P.M., Weinlaender M., et al (2001) Combination use of bovine porous bone mineral, enamel matrix proteins, and a bioabsorbable membrane in intrabony periodontal defects in humans. Journal of periodontology 72:583–589

Lekovic V, Carnargo P, Weinlainder M, Nedic M (2001) The use of enamel derived matrix and natural bone mineral in the treatment of interproximal periodontal defects. Journal of dental research 80:1214, Abstract no: 105

Lempesi E, Pandis N, Fleming PS, Mavragani M (2014) A comparison of apical root resorption after orthodontic treatment with surgical exposure and traction of maxillary impacted canines versus that without impactions. European journal of orthodontics 36:690–697

Lena K, Marianne K (2017) Ozone treatment on dentin hypersensitivity surfaces–a pilot study. The Open Dentistry Journal 11:65

Leppaniemi A., Lukinmaa P.L., Alaluusua S. (2001) Nonfluoride hypomineralizations in the permanent first molars and their impact on the treatment need. Caries research 35:36–40

Letourneau Y., Perusse R., Buithieu H. (2001) Oral manifestations of Ehlers-Danlos syndrome. Journal (Canadian Dental Association) 67:330–334

Leung VW, Low B, Yang Y, Botelho MG (2018) Oral Rehabilitation of Young Adult with Amelogenesis Imperfecta. The journal of contemporary dental practice 19:599–604

Leverd C, Laumaille M, Jayet M, Delfosse C ionomers: the material. Dear readers 41

Levey EB, Stashinko E, Clegg NJ, Delgado MR (2010) Management of children with holoprosencephaly. American journal of medical genetics Part C, Seminars in medical genetics 154:. <https://doi.org/10.1002/ajmg.c.30254>

Levin LS (1975) Letter: “Confusing” article. Journal of the American Dental Association (1939) 90:912–913

Levin LS (1981) The dentition in the osteogenesis imperfecta syndromes. Clinical orthopaedics and related research 64–74

Levine R.S., Beal J.F., Fleming C.M. (1989) A photographically recorded assessment of enamel hypoplasia in fluoridated and non-fluoridated areas in England. British dental journal 166:249–252

Levy BP, Reeve CM, Kierland RR (1969) The oral aspects of epidermolysis bullosa dystrophica: a case report. Journal of periodontology 40:431–434

Levy SM, Warren JJ, Broffitt B, Nielsen B (2005) Factors associated with parents’ esthetic perceptions of children’s mixed dentition fluorosis and demarcated opacities. Pediatric dentistry 27:486–492

Lewandowska A (1971) [Prosthetic management of cases of congenital enamel hypoplasia]. Protetyka stomatologiczna 21:271–275

Lewandowski K.C., Brzozowska M., Lewinski A., et al (2019) Metformin paradoxically worsens insulin resistance in SHORT syndrome. Diabetology and Metabolic Syndrome 11:. <https://doi.org/10.1186/s13098-019-0477-z>

Lewis S (1994) Controlled comparison of the cardiovascular effects of levobunolol 0.25% ophthalmic solution and betaxolol 0.25% ophthalmic suspension. Journal of glaucoma 3:308‐314

Li J., Prinsen K., Marysael T., et al (2010a) Small molecular dual targeting chemo- and radiotherapy: An integrated approach to improve cancer treatability and curability. Annals of Oncology 21:. <https://doi.org/10.1093/annonc/mdq115>

Li J., Sun Z., Wang H., et al (2010b) Targeting radiotherapy to cancer via small necrosis avid molecules: A simpler approach to improve treatability and curability? Journal of Nuclear Medicine 51:

Li J.N., Nijhawan R.I., Srivastava D. (2019) Cutaneous Surgery in Patients Who Are Pregnant or Breastfeeding. Dermatologic Clinics 37:307–317. <https://doi.org/10.1016/j.det.2019.03.002>

Li M-Y (2012) Contemporary Approach to Dental Caries. BoD–Books on Demand

Li RW (1999) Adhesive solutions: report of a case using multiple adhesive techniques in the management of enamel hypoplasia. Dental update 26:277

Li R.W. (1999) Adhesive solutions: report of a case using multiple adhesive techniques in the management of enamel hypoplasia. Dental update 26:277–287

Li S.M., Zou J., Wang Z., et al (2003) Quantitative assessment of enamel hypomineralization by KaVo DIAGNOdent at different sites on first permanent molars of children in China. Pediatric dentistry 25:485–490

Li W, Xiao L, Hu J (2012) The use of enamel matrix derivative alone versus in combination with bone grafts to treat patients with periodontal intrabony defects: a meta-analysis. Journal of the American Dental Association (1939) 143:e46

Li X, Venugopalan SR, Cao H, et al (2014) A model for the molecular underpinnings of tooth defects in Axenfeld-Rieger syndrome. Human molecular genetics 23:194–208. <https://doi.org/10.1093/hmg/ddt411>

Li X, Zhang J, Niu R, et al (2019a) Effect of fluoride exposure on anxiety- and depression-like behavior in mouse. Chemosphere 215:454–460. <https://doi.org/10.1016/j.chemosphere.2018.10.070>

Li Y, Decker S, Yuan Z-A, et al (2005) Effects of sodium fluoride on the actin cytoskeleton of murine ameloblasts. Archives of oral biology 50:681–688

Li Y, Konicki WS, Wright JT, et al (2013) Mouse genetic background influences the dental phenotype. Cells, tissues, organs 198:448–456. <https://doi.org/10.1159/000360157>

Li Y, Navia JM, Bian JY (1996) Caries experience in deciduous dentition of rural Chinese children 3-5 years old in relation to the presence or absence of enamel hypoplasia. Caries research 30:8–15

Li Y., Pugach M.K., Kuehl M.A., et al (2011) Dental enamel structure is altered by expression of dominant negative RhoA in ameloblasts. Cells Tissues Organs 194:227–231. <https://doi.org/10.1159/000324559>

Li Y, Wu Y, Wang C, et al (2019b) Incidence of pediatric metachronous contralateral inguinal hernia and the relationship with contralateral patent processus vaginalis. Surgical endoscopy 33:1087–1090. <https://doi.org/10.1007/s00464-018-6359-x>

Li Y.-H., Zhang C.-L., Zhang X.-Y., et al (2015) Effects of mild induced hypothermia on hippocampal connexin 43 and glutamate transporter 1 expression following traumatic brain injury in rats. Molecular Medicine Reports 11:1991–1996. <https://doi.org/10.3892/mmr.2014.2928>

Liao Y-H, Lee H-J, Huang W-J, et al (2016) Hispidulin alleviated methamphetamine-induced hyperlocomotion by acting at α6 subunit-containing GABAA receptors in the cerebellum. Psychopharmacology 233:3187–3199. <https://doi.org/10.1007/s00213-016-4365-z>

Licht W.S., Leveton E.E. (1980) Overdentures for treatment of severe attrition. The Journal of prosthetic dentistry 43:497–500

Liebenberg WH (1994) Enamel and gingival abrasion: a case report illustrating the combined esthetic treatment of fluorosis and melanoplakia. Journal (Canadian Dental Association) 60:701

Light EI, Rakow B, Fraze RL (1975) An esthetic transitional treatment for amelogenesis imperfecta: report of two cases. Journal of the American Dental Association (1939) 90:166–170

Lignon G., Beres F., Quentric M., et al (2017) FAM20A gene mutation: Amelogenesis or ectopic mineralization? Frontiers in Physiology 8:. <https://doi.org/10.3389/fphys.2017.00267>

Lim H-C, Lee J-S, Jung U-W, Choi S-H (2016) Bone Regenerative Potential of Enamel Matrix Protein in the Circumferential Defect Around a Dental Implant. Implant dentistry 25:179–185. <https://doi.org/10.1097/ID.0000000000000383>
[truncated: 412,574 more chars]
